# Synthesis of *cis*-thiiranes as diastereoselective access to epoxide congeners via 4π-electrocyclization of thiocarbonyl ylides

**DOI:** 10.1038/s41467-022-32499-3

**Published:** 2022-08-16

**Authors:** Su-min Song, Jaeseong Jin, Jun-Ho Choi, Won-jin Chung

**Affiliations:** Department of Chemistry, Gwangju Institute of Science and Technology, Gwangju, 61005 Republic of Korea

**Keywords:** Synthetic chemistry methodology, Stereochemistry

## Abstract

Organochalcogen heterocycles are ubiquitously present and widely utilized in various fields. Among them, oxirane has been extensively studied, and all of the stereoisomeric forms are readily available. In contrast, synthetic studies on thiirane were rarely reported, and thus the useful sulfur-congener of oxirane has been difficult to access in a stereodefined form. In this research, a general stereoselective synthesis of *cis*-thiiranes is accomplished by taking advantage of stereospecific electrocyclization of *trans*-thiocarbonyl ylides, which are generated in situ from readily available *E*,*E*-aldazine *N*-oxides upon treatment with Lawesson’s reagent. This newly developed practical method provides a variety of *cis*−1,2-diarylthiiranes as essentially single diastereomers in high yields under mild reaction conditions. The intermediacy of *trans*-thiocarbonyl yilde is confirmed by mechanistic experiments, and the excellent stereocontrol is rationalized by DFT calculation.

## Abstract (web summary)

Thiirane, the sulfur congener of oxirane, has been hard to access in a stereodefined form. Here, the authors accomplished a highly stereoselective synthesis of *cis*-diarylthiiranes through the stereospecific sulfurization of *E,E*-aldazine N-oxides.

## Introduction

**Fig. 1:**
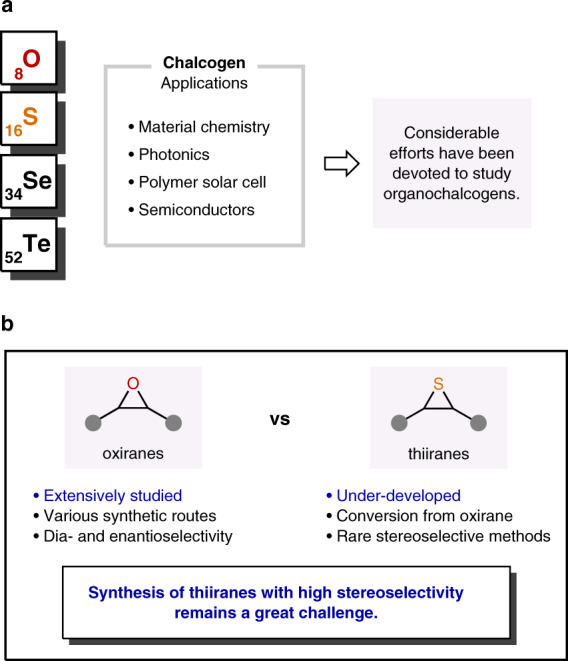
Thiiranes as synthetic targets. **a** Importance of organochalcogens. **b** Comparison between oxiranes and thiiranes.

The chalcogens, elements in group 16 of the periodic table, are widely utilized in various fields (Fig. 1a). In particular, non-covalent interaction between chalcogen atoms and electron donors, so-called chalcogen bonding plays important roles in materials chemistry^1–3^ as well as noncovalent organocatalysis^1,4,5^. Moreover, the combination with other electropositive elements composes chalcogenides, which have numerous applications such as chalcogenide glasses^6–8^, polymer solar cells^9^, wide band gap semiconductors^10^, and electrochemical sensors^11^. Furthermore, chalcogens are often found in natural products and pharmacologically active compounds. Among them, three-membered heterocycles containing a chalcogen are ubiquitous subunits, and significant efforts have been dedicated to the development of synthetic methods for oxiranes^12,13^. The sulfur congeners, thiiranes are also useful synthetic precursors that can be transformed into an array of functional groups via ring-opening or desulfurization^14–20^. In addition, versatile sulfur-containing polymers can be produced via ring-expansion polymerizations or copolymerizations^21–25^. Moreover, thiiranes have been examined as analogs of biologically active oxiranes^26–28^, and several derivatives showed inhibitory activity against gelatinases^29,30^. However, despite the apparent utility, synthetic methods for thiiranes are not well established compared to oxiranes (Fig. 1b). Indeed, the stereoselective construction of thiiranes remains a great challenge. For example, whereas all of the stereoisomers of stilbene oxide can be easily prepared and are even commercially available, the related stilbene sulfides are typically produced through stereospecific conversion of the corresponding stilbene oxides^31^. Clearly, it is highly desirable to develop a general and practical stereoselective synthesis of thiiranes.

**Fig. 2:**
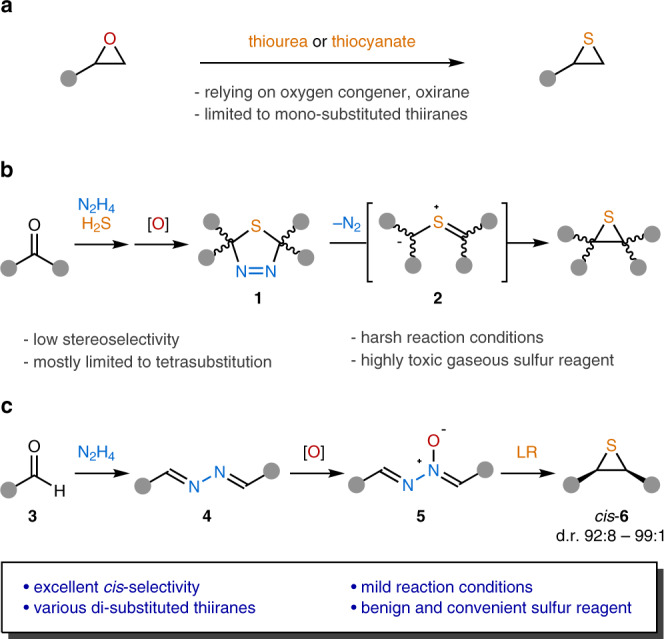
Approach to stereoselective synthesis of thiiranes. **a** Common method for thiirane synthesis. **b** Previous approach to thiiranes via the intermediacy of thiocarbonyl ylides. **c** This work: Diastereoselective synthesis of *cis*-thiiranes from aldazine *N*-oxides. LR Lawesson’s reagent, d.r. diastereomeric ratio.

Thiirane synthesis has been generally accomplished via the conversion from oxirane analogs with thiourea or thiocyanate as a sulfurating reagent (Fig. 2a)^32–38^. Consequently, the stereostructure of thiiranes necessarily relies on the configuration of oxiranes. In a special case, it is possible to introduce a stereochemical element during the chalcogen substitution step via a kinetic resolution process as recently demonstrated by the List group utilizing the chiral phosphoric acid catalysis^39,40^. However, only terminal thiiranes can be prepared by this strategy. Alternatively, the episulfidation of alkenes has been investigated^15^, but this seemingly straightforward epoxidation-like approach is applicable only to a limited range of cyclic alkenes. Otherwise, stereoselective synthesis of thiiranes has rarely been developed^41–45^, and most of the reported examples have serious drawbacks such as low stereoselectivity, narrow substrate scope, and/or the requirement of highly toxic reagents. Among these precedents, a unique synthetic sequence of the Barton–Kellogg reaction is noteworthy (Fig. 2b)^46–53^. The Kellogg group developed a sequential process involving non-stereoselective sulfurization of azines followed by dehydrogenation to furnish thiadiazolines (**1**) as a mixture of diastereomers^48^. Interestingly, the isolated *trans-* and *cis-***1** were stereospecifically transformed to the corresponding *cis-* and *trans-*thiiranes, respectively. The stereochemical outcome was rationalized by 4π-electrocyclization of the putative thiocarbonyl ylides (**2**), which were derived from **1** via thermal extrusion of dinitrogen gas. Unfortunately, despite the intriguing stereochemical properties, this method has not received much attention because of the use of a highly toxic and inconvenient gaseous sulfurating reagent, H_2_S. Moreover, the formation of **1** requires harsh and toxic oxidants such as Pb(OAc)_2_ or diethyl azodicarboxylate (DEAD). The most critical drawback is the lack of stereoselectivity in the sulfurization step, which diminishes the practicality of this method quite substantially. Our group has been interested in the unusual reactivity of electrophilically activated 1,2-diazines and related organonitrogen compounds^54,55^. While investigating the reactions of aldazine *N*-oxides (**5**) with various electrophiles, we serendipitously observed the highly diastereoselective formation of *cis*-disubstituted thiiranes (**6**) upon treatment with Lawesson’s reagent (LR) (Fig. 2c).

Herein, we report a general synthetic method for *cis-*diarylthiiranes through the stereospecific sulfurization of aldazine *N-*oxides. Our newly discovered process resembles the Barton–Kellogg reaction but exhibits several distinct advantages. Because azine is pre-oxidized under relatively mild conditions^56–58^ prior to the sulfurization, harsh oxidation conditions are avoided. In addition, a more convenient and less toxic sulfurating reagent, LR, is utilized. Most importantly, *cis*-thiiranes are produced with an excellent level of diastereoselectivity.

## Results and discussion

Aldazine *N-*oxides (**5**) were readily prepared by a two-step sequence involving double condensation of aldehydes with hydrazine^59,60^ and the following *N-*oxidation (see Section 2.1 of the Supplementary Information for details).^56–58^ All of **5** were obtained with *E*,*E*-configuration^59,60^ and are bench-stable solids that can be stored for months without decomposition. On the other hand, it was challenging to access unsymmetrical substrates because of the disproportionation during both C = N condensation and *N*-oxidation, and aliphatic aldazines are unstable under the *N*-oxidation conditions. At the outset, the reaction conditions for thiirane formation were optimized with benzaldazine *N-*oxide (**5a**) and LR (Table 1). In the presence of 1.0 equiv of LR in CH_2_Cl_2_ at −10 °C, 2,3-diphenylthiirane (**6a**) was produced in 13% yield with a reasonably high 84:16 *cis*/*trans* selectivity (entry 1). Although the yield was low, the dominant formation of *cis-*isomer was encouraging. When the reaction was conducted in polar MeCN, both yield (23%) and *cis-*selectivity (94:6) were increased (entry 2). Thus, a few other polar solvents were examined. DMSO was unsuitable because it reacted vigorously with LR. Gratifyingly, the use of DMF resulted in a synthetically useful 62% yield with an enhanced 97:3 *cis-*selectivity (entry 3). The analysis of the crude mixture revealed that a small amount of DMTF was generated by thionation of DMF. Hence, DMTF was employed as a solvent in order to evaluate the potentially promoting activity, but a drastically decreased 25% yield was afforded (entry 4). Subsequently, upon a brief survey of other amide solvents, an improved 67% yield and an excellent 99:1 *cis-*selectivity were obtained in DMPU (entry 5), whereas the use of DMA was less effective (entry 6). In these experiments, a noticeable exotherm was detected during the addition of LR. It was hypothesized that the elevated internal temperature could have caused unproductive side reactions, leading to moderate chemical yields. Therefore, the reaction was carried out at a lower temperature. For this purpose, DMF was employed as a solvent because of the high melting point of DMPU. At −50 °C, an increased 71% yield was obtained with an essentially exclusive *cis-*selectivity (entry 7). To our delight, when LR was added slowly as a solution for finer temperature control, the yield was even further improved to 78% (entry 8). In this case, DMPU was used as a solvent for LR because DMF reacts with LR at room temperature^61^. With this protocol, higher and lower loadings of LR were examined, but the yields were slightly diminished in both cases (entries 9 and 10).

**Table 1:** Reaction conditions optimization^a^


Entry	A	Solvent	Temp. (°C)	Time	Yield (%)^b^	d.r.^c^
1	1.0	CH_2_Cl_2_	−10	20 min	13	84:16
2	1.0	MeCN	−10	20 min	23	94:6
3	1.0	DMF	−10	20 min	62	97:3
4	1.0	DMTF	−10	20 min	25	99:1
5	1.0	DMPU	−10	20 min	67	99:1
6	1.0	DMA	−10	20 min	59	98:2
7	1.0	DMF	−50	4 h	71	99:1
8^d^	1.0	DMF	−50	4 h	78	99:1
9^d^	1.5	DMF	−50	4 h	75	99:1
10^d^	0.5	DMF	−50	4 h	71	99:1

*DMF* *N*,*N*-dimethylformamide, *DMTF* *N*,*N*-dimethylthioformamide, *DMPU* *N*,*N*’-dimethylpropyleneurea, *DMA* *N*,*N*-dimethylacetamide.

^a^Reaction conditions: **5a** (1.0 mmol) and LR (1.0 mmol) in solvent (10.0 mL). LR was added in one-portion.

^b^Isolated yields after column chromatography.

^c^Determined by ^1^H NMR analysis of the isolated material.

^d^LR was added slowly over 1 h as a solution in DMPU.

**Fig. 3:**
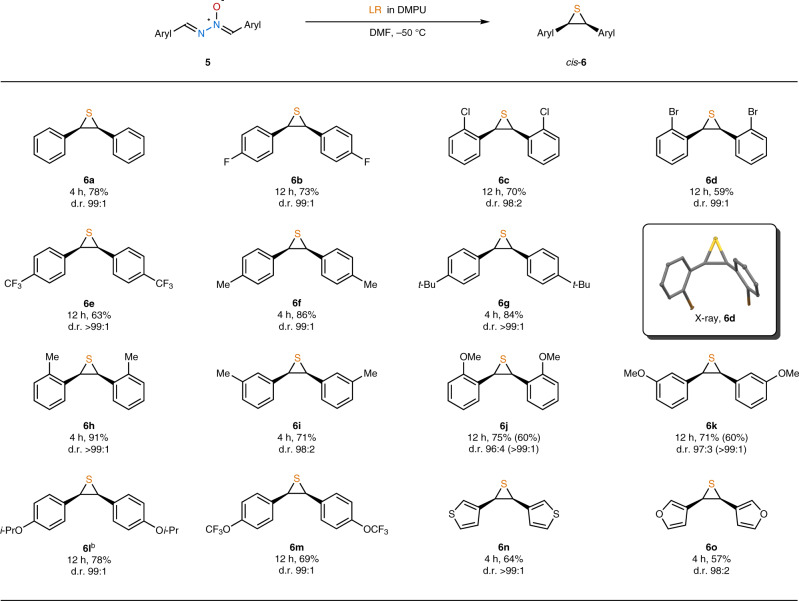
Substrate scope of LR-mediated *cis-*diarylthiirane synthesis. **a** Reaction conditions: **5** (1.0 mmol) in DMF (5.0 mL) and LR (1.0 mmol) in DMPU (5.0 mL). Isolated yields after column chromatography are given. Diastereomeric ratio was determined by ^1^H NMR analysis of the isolated material. Data after recrystallization are given in the parenthesis. **b** Contaminated by alkene (86:14).

**Fig. 4:**
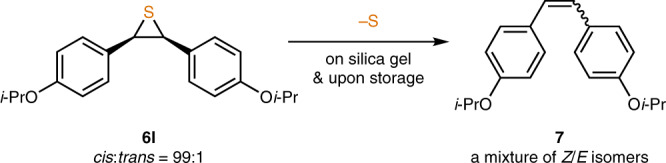
Instability of electron-rich thiirane. Non-stereospecific, spontaneous desulfurization of *p*-alkoxy-*cis*-thiirane.

With the optimal reaction conditions in hand, a wide range of 1,2-diarylaldazine *N*-oxides was surveyed (Fig. 3). Electron-withdrawing groups were well tolerated. Not only *p*-fluorine but also sterically hindering *o*-chlorine/bromine substituents could be introduced, producing **6b**–**6d** in 59–73% yields. Moreover, highly electron-deficient, *p-*trifluoromethyl-substituted thiirane **6e** was successfully afforded in 63% yield. It was possible to install electron-donating alkyl groups at any positions on the aryl rings, too. Thiiranes with *p-*methyl (**6f**), *p*-*tert*-butyl (**6g**), *o*-methyl (**6h**), or *m*-methyl (**6i**) groups were formed in 71–91% yields. Strongly electron-donating alkoxy groups could be present at the *o*-positions, again, without steric encumbrance problem, providing **6j** in still high yield and diastereoselectivity. The presence of *m*-methoxy groups was allowed to give **6k** in 71% yield, as well. Unfortunately, *p-*alkoxy substitution resulted in an unstable product. Even though the predominant formation of **6l** was observed by ^1^H NMR analysis of the crude mixture, the thiirane decomposed slowly to the corresponding alkene upon purification on silica gel. Thus, **6l** was obtained as a mixture with alkene (86:14). Moreover, the loss of sulfur atom took place spontaneously over time to increase the amount of alkene upon storage even in the fridge (Fig. 4).^14^ This desulfurization process is not stereospecific, and thus a mixture of *Z-* and *E-*alkene isomers **7** was produced from an essentially diastereopure thiirane. Such decomposition was suppressed by attenuating the electron-donating ability of the alkoxy group. Hence, **6m** containing *p*-trifluoromethoxy groups could be isolated in 69% yield. Finally, heteroaromatic substrates were examined, and both 3-thienyl and 3-furyl moieties were successfully employed to give **6n** and **6o** in 64% and 57% yields, respectively. On the other hand, the isomeric 2-thienyl and 2-furyl substrates could not be employed because the corresponding thiirane products decomposed rapidly on silica gel. Overall, *cis*-diarylthiiranes were consistently obtained in good yields with excellent diastereoselectivity regardless of the electronic and steric properties of the substituents. The *cis*-configuration of thiirane products was unambiguously established by X-ray crystallographic analysis of **6d** (Supplementary Fig. 4).

**Fig. 5:**
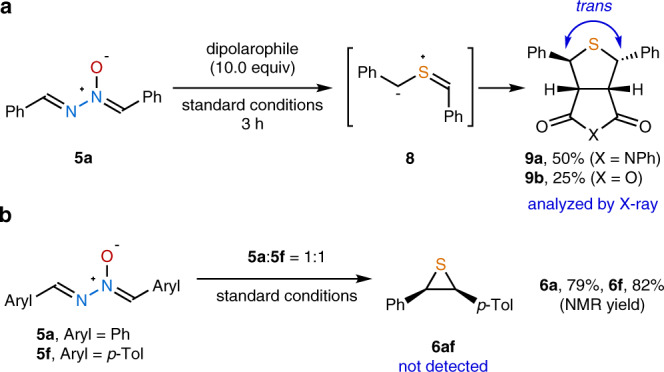
Mechanistic experiments. **a** Trapping the *trans*-thiocarbonyl ylide intermediate with dipolarophiles. **b** Crossover experiment with two different aldazine *N*-oxides.

Mechanistic experiments were carried out for the newly developed *cis-*selective thiirane synthesis (Fig. 5). On the basis of Kellogg’s study^48^, it was hypothesized that the observed *cis*-configuration of thiiranes would be originated from the intermediacy of a *trans-*thiocarbonyl ylide via stereospecific conrotatory 4π-electrocyclization. Therefore, trapping of a putative *trans-*thiocarbonyl ylide **8** was pursued, and the 1,3-dipolar cycloadducts **9a** and **9b** were successfully obtained employing *N-*phenylmaleimide and maleic anhydride (Fig. 5a)^62^. The 1,3-*trans*-configurations of these compounds were determined by single-crystal X-ray diffraction analysis (Supplementary Figs. 2 and 3), highly supporting the presence of *trans-***8**. In addition, a crossover experiment was conducted with a 1:1 mixture of **5a** and **5f** (Fig. 5b). Under the standard reaction conditions, only **6a** and **6f** were afforded, and the scrambled product **6af** was not detected. Therefore, the intermolecular reaction pathway was excluded.

**Fig. 6:**
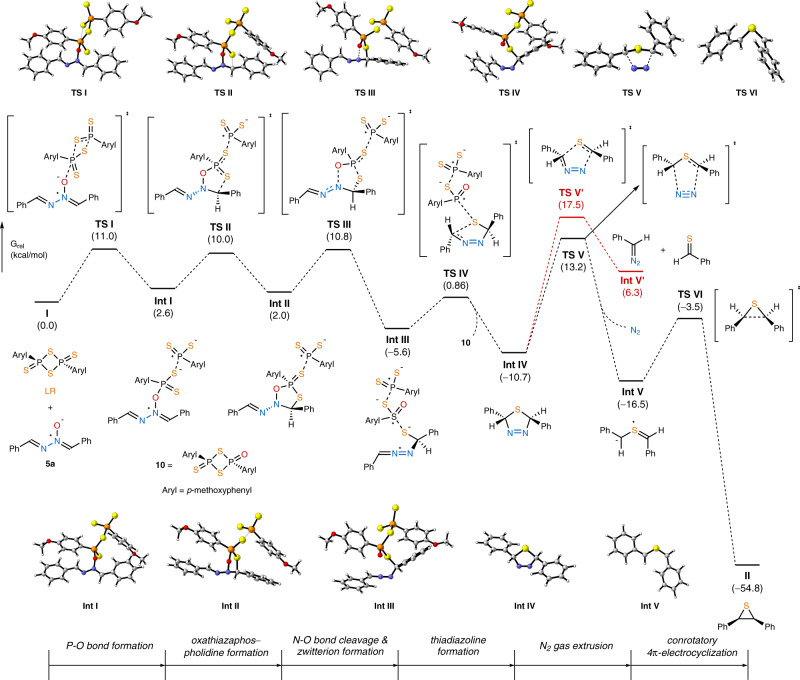
DFT calculation of the reaction mechanism via a thiocarbonyl ylide. Gibbs free energy profile at the M06-2X/6-311 + G(d,p)/PCM(DMF) level of theory.

To gain insight into the detailed reaction mechanism, a computational study was performed by the density functional theory calculation at the M06-2X/6-311 + G(d,p)/PCM(DMF) level of theory (Fig. 6, Supplementary Data 1)^63–66^. Whereas typical thionation with LR requires heating, our thiirane formation proceeds at a subzero temperature. Thus, dissociation of LR is less likely under our reaction conditions, and the dimeric form was employed in the computational analysis. The process is initiated by the coordination of aldazine *N*-oxide **5a** onto dimeric LR to generate **Int I**^67^, and the subsequent cyclization affords oxathiazaphospholidine sulfide **Int II**, completing a stepwise formal [3 + 2] cycloaddition^68^. Then, the exergonic production of zwitterion **Int III** is driven by the formation of a stable P = O bond^67^. Until here, even after cleavage of covalent bonds in the LR moiety, the electron-deficient phosphorus centers and the electron-rich sulfur anions still appear to associate through electrostatic interaction. Notably, a few calculated structures were located only when the solvent effect of DMF was considered in the computation. This result is consistent with the experimental observation. During the reaction conditions optimization, the yield of thiirane was substantially improved upon the use of DMF (Table 1, entry 3), whereas the reactions in less polar solvents gave inferior results (Table 1, entries 1, 2, and 4). It is presumed that a highly polar solvent is needed for the stabilization of multiply charge-separated transition structures and intermediates. Subsequently, a facile intramolecular nucleophilic attack of the reactive sulfur anion onto the neighboring diazenium moiety furnishes *trans-*thiadiazoline **Int IV** with concomitant release of an oxygenated LR analog **10**. The calculated pathway up to this point from **I** is composed of a series of stereospecific transformations, all of which take place at one side of **5a**. In consequence, the *E*,*E*-geometry of aldazine *N*-oxide leads to the *trans*-configuration of thiadiazoline. Then, upon a concerted extrusion of dinitrogen gas, the experimentally confirmed *trans-*thiocarbonyl ylide **Int V** is produced^69^. Alternatively, **Int IV** may split into a diazo compound and a thial (**Int V’**) via a [3 + 2] cycloreversion (red line)^50^. This potential side reaction is endergonic and reversible. Hence, the stereochemical integrity of **Int IV** may be compromised through unselective recombination. Fortunately, the activation energy of the desired pathway is 4.3 kcal/mol lower. Moreover, the intermolecular mechanism has been ruled out by the crossover experiment (Fig. 5b). Finally, **Int V** undergoes thermal conrotatory 4π-electrocyclization to give the product *cis-*thiirane **II**.

In summary, a generally applicable, highly stereoselective synthesis of *cis*-thiiranes has been realized under mild reaction conditions utilizing Lawesson’s reagent as a convenient sulfur source. The newly developed method provides access to a wide variety of *cis*-1,2-diarylthiiranes from *E*,*E*-aldazine *N-*oxides in high yields with almost exclusive diastereoselectivity. Mechanistic experiments, as well as DFT calculation, suggest a reaction mechanism composed of stereospecific transformations involving 4π-electrocyclization of a *trans*-thiocarbonyl ylide as the origin of the exquisite diastereocontrol. Through this research, the useful sulfur congener of oxirane becomes available in a stereodefined form. Further expansion of this unique reactivity is currently investigated in our laboratories.

## Methods

### A representative procedure for LR-mediated synthesis of *cis*-thiiranes from aldazine *N*-oxides

To a mixture of benzaldazine *N*-oxide (**5a**, 224 mg, 1.00 mmol) in DMF (5.0 mL) was added LR (404 mg, 1.00 mmol) as a solution in DMPU (5.0 mL) at –50 °C dropwise under Ar. The change in internal temperature should not exceed 1 °C when the LR solution was added. After 4 h, the reaction mixture was transferred to a 100 mL separatory funnel and diluted with CH_2_Cl_2_ (4 mL). The mixture was washed with water (50 mL × 3) and brine (50 mL). The combined aqueous layers were extracted with CH_2_Cl_2_ (3 mL × 6). The combined organic layers were dried over anhydrous MgSO_4_ (2 g), filtered through a glass frit, and concentrated in vacuo. The residue was purified immediately by flash column chromatography (SiO_2_, ø = 3.5 cm, *l* = 5.0 cm, hexanes, *R*_f_ = 0.20, KMnO_4_) to give *cis*-2,3-diphenylthiirane (**6a**, 165 mg, 78%, *cis*:*trans* = 99:1) as white solid. Data for **6a**: ^1^H NMR (400 MHz, CDCl_3_): δ 7.14–7.10 (m, 10H), 4.38 (s, 2H); ^13^C NMR (100 MHz, CDCl_3_): δ 135.2, 129.5, 127.8, 127.3, 44.2; HRMS (ESI): [M–H]^–^ calcd for C_14_H_11_S: 211.0587; found: 211.0586.

## Supplementary information

Supplementary Information

Description of Additional Supplementary Files

Supplementary Data 1

## Data Availability

The data supporting the findings of this study are available within this article and its Supplementary Information, which contains experimental details, characterization data, copies of NMR spectra for all new compounds, and DFT calculation data. Crystallographic data for **6d**, **9a**, and **9b** have been deposited at the Cambridge Crystallographic Data Centre (CCDC) under deposition numbers CCDC 2119467, 2119466, and 2119465, respectively. Copies of the data can be accessed free of charge via https://www.ccdc.cam.ac.uk/structures/.

## References

[1] Vogel L, Wonner P, Huber SM (2019). Chalcogen bonding: an overview. Angew. Chem. Int. Ed..

[2] Ho PC, Wang JZ, Meloni F, Vargas-Baca I (2020). Chalcogen bonding in materials chemistry. Coord. Chem. Rev..

[3] Mahmudov KT, Kopylovich MN, Guedes da Silva MFC, Pombeiro AJL (2017). Chalcogen bonding in synthesis, catalysis and design of materials. Dalton Trans..

[4] Young CM (2020). The importance of 1,5-oxygen···chalcogen interactions in enantioselective isochalcogenourea catalysis. Angew. Chem. Int. Ed..

[5] Wang W (2019). Chalcogen–chalcogen bonding catalysis enables assembly of discrete molecules. J. Am. Chem. Soc..

[6] Eggleton BJ, Luther-Davies B, Richardson K (2011). Chalcogenide photonics. Nat. Photonics.

[7] Zakery A, Elliott SR (2003). Optical properties and applications of chalcogenide glasses: a review. J. Non Cryst. Solids.

[8] Sanghera JS, Shaw LB, Aggarwal ID (2002). Applications of chalcogenide glass optical fibers. C. R. Chim..

[9] Freitas JN, Gonçalves AS, Nogueira AF (2014). A comprehensive review of the application of chalcogenide nanoparticles in polymer solar cells. Nanoscale.

[10] Woods-Robinson R (2020). Wide band gap chalcogenide semiconductors. Chem. Rev..

[11] Suresh, R., Pandiaraj, M., Sankaralingam, M. & Giribabu, K. Graphene–metal chalcogenide modified electrochemical sensor. In *Graphene-Based Electrochemical Sensors for Biomolecules* (eds Pandikumar, A. & Rameshkumar, P.) Ch. 6, 139–153 (Elsevier, 2019).

[12] Faveri GD, Ilyashenko G, Watkinson M (2011). Recent advances in catalytic asymmetric epoxidation using the environmentally benign oxidant hydrogen peroxide and its derivatives. Chem. Soc. Rev..

[13] Zhu Y, Wang Q, Cornwall RG, Shi Y (2014). Organocatalytic asymmetric epoxidation and aziridination of olefins and their synthetic applications. Chem. Rev..

[14] Sander M (1966). Thiiranes. Chem. Rev..

[15] Adam W, Bargon RM (2004). Synthesis of thiiranes by direct sulfur transfer: the challenge of developing effective sulfur donors and metal catalysts. Chem. Rev..

[16] Murphree, S. S. Three-membered heterocycles. Structure and reactivity. In *Modern Heterocyclic Chemistry* Vol. 4 (eds Alvarez-Builla, J., Vaquero, J. J. & Barluenga, J.), Ch. 2, 11–162 (Wiley, 2011).

[17] Rogers E, Araki H, Batory LA, Mclnnis CE, Njardarson JT (2007). Highly selective copper-catalyzed ring expansion of vinyl thiiranes: application to synthesis of biotin and the heterocyclic core of plavix. J. Am. Chem. Soc..

[18] Iranpoor N, Firouzabadi H, Jafari AA (2003). Conversion of thiiranes to β-chlorothioacetates catalyzed with CoCl_2_. Synth. Commun..

[19] Sauve AA, Groves JT (2002). Synthesis of trithiolanes and tetrathianes from thiiranes catalyzed by ruthenium salen nitrosyl complexes. J. Am. Chem. Soc..

[20] Xu J (2020). Recent synthesis of thietanes. Beilstein J. Org. Chem..

[21] Chao J-Y (2022). Controlled disassembly of elemental sulfur: an approach to the precise synthesis of polydisulfides. Angew. Chem. Int. Ed..

[22] Kudo H (2020). Living ring-expansion polymerization of thiirane with cyclic monocarbamothioates. Macromolecules.

[23] Takahashi A, Tsunoda S, Yuzaki R, Kameyama A (2020). Thioacyl-transfer ring-expansion polymerization of thiiranes based on a cyclic dithiocarbamate initiator. Macromolecules.

[24] Takahashi A, Yuzaki R, Ishida Y, Kameyama A (2019). Controlled ring-expansion polymerization of thiiranes based on cyclic aromatic thiourethane initiator. J. Polym. Sci. Part A: Polym. Chem..

[25] Schuetz J-H, Sandbrink L, Vana P (2013). Insights into the ring-expansion polymerization of thiiranes with 2,4-thiazolidinedione. Macromol. Chem. Phys..

[26] Schramm F, Müller A, Hammer H, Paschke A, Schüürmann G (2011). Epoxide and thiirane toxicity in vitro with the ciliates tetrahymena puriformis: structural alerts indicating excess toxicity. Environ. Sci. Technol..

[27] Gao M (2015). Acceleration of diabetic wound healing using a novel protease–anti-protease combination therapy. Proc. Natl Acad. Sci. USA.

[28] Lee M (2005). Synthesis of chiral 2-(4-phenoxyphenylsulfonylmethyl)thiiranes as selective gelatinase inhibitors. Org. Lett..

[29] Fabre B (2014). New clicked thiirane derivatives as gelatinase inhibitors: the relevance of the P1’ segment. RSC Adv..

[30] Lee M (2012). Structure–activity relationship for thiirane-based gelatinase inhibitors. ACS Med. Chem. Lett..

[31] Ketcham R, Shah VP (1963). *cis-* and *trans-*stilbene sulfides. J. Org. Chem..

[32] Akhlaghinia B, Rahimizadeh M, Eshghi H, Zhaleh S, Rezazadeh S (2012). Green synthesis of thiiranes from oxiranes under solvent- and catalyst-free conditions. J. Sulfur Chem..

[33] Zeynizadeh B, Baradarani MM, Eisavi R (2011). A practical and eco-friendly method for conversion of epoxides to thiiranes with immobilized thiourea on CaCo_3_. Phosphorus Sulfur Silicon Relat. Elem..

[34] Wu L, Wang Y, Yan F, Yang C (2010). Facile conversion of epoxides to thiiranes with ammonium thiocyanate catalyzed with etidronic acid. Bull. Korean Chem. Soc..

[35] Zeynizadeh B, Yeghaneh S (2009). A green protocol for solvent-free conversion of epoxides to thiiranes with dowex-50WX8–supported thiourea. Phosphorous Sulfur Silicon Relat Elem.

[36] Yadav JS (2008). Iodine as a mild, efficient, and cost-effective catalyst for the synthesis of thiiranes from oxiranes. Monatch. Chem..

[37] Bandgar BP, Patil AV, Kamble VT, Totre JV (2007). An efficient synthesis of thiiranes from oxiranes using fluoroboric acid adsorbed on silica gel (HBF_4_–SiO_2_) as a catalyst under mild conditions in the absence of solvent. J. Mol. Catal. A Chem..

[38] Yadav JS, Reddy BVS, Baishya G (2003). Indium tribromide: a novel and highly efficient reagent for the conversion of oxiranes to thiiranes. Synlett.

[39] Liao S, Leutzsch M, Monaco MR, List B (2016). Catalytic enantioselective conversion of epoxides to thiiranes. J. Am. Chem. Soc..

[40] Duan M (2022). Chiral phosphoric acid catalyzed conversion of epoxides into thiiranes: mechanism, stereochemical model, and new catalyst design. Angew. Chem. Int. Ed..

[41] Lin X (2022). Asymmetric catalytic (2+1) cycloaddition of thioketones to synthesize tetrasubstituted thiiranes. Angew. Chem. Int. Ed..

[42] Schmidt TA, Sparr C (2021). Catalyst-controlled stereoselective Barton–Kellogg olefination. Angew. Chem. Int. Ed..

[43] Cano I (2012). *N*-(Diazoacetyl)oxazolidin-2-thiones as sulfur-donor reagents: asymmetric synthesis of thiiranes from aldehydes. Angew. Chem. Int. Ed..

[44] Zhou C, Fu C, Ma S (2007). Highly selective thiiranation of 1,2-allenyl sulfones with Br_2_ and Na_2_S_2_O_3_: mechanism and asymmetric synthesis of alkylidenethiiranes. Angew. Chem. Int. Ed..

[45] Collazo LR, Guziec FS (1993). Stereoselective synthesis of unhindered olefins by 2-fold extrusion reactions. J. Org. Chem..

[46] Kellogg RM (1976). The molecules R_2_CXCR_2_ including azomethine, carbonyl and thiocarbonyl ylides. their syntheses, properties and reactions. Tetrahedron.

[47] Barton, D. H. R. & Willis, B. J. Olefin synthesis by two-fold extrusion processes. Part I Preliminary experiments. *J. Chem. Soc. Perkin Trans.* **1**, 305–310 (1972).

[48] Buter J, Wassenaar S, Kellogg RM (1972). Thiocarbonyl ylides. Generation, properties, and reactions. J. Org. Chem..

[49] Mlostoń G, Pipiak P, Heimgartner H (2016). Diradical reaction mechanisms in [3+2]-cycloadditions of hetaryl thioketones with alkyl- or trimethylsilyl-substituted diazomethanes. Beilstein J. Org. Chem..

[50] Mlostoń G, Jasiński R, Kula K, Heimgartner H (2020). A DFT study on the Barton–Kellogg reaction—the molecular mechanism of the formation of thiiranes in the reaction between diphenyldiazomethane and diaryl thioketones. Eur. J. Org. Chem..

[51] Kowalski MK, Obijalska E, Mlostoń G, Heimgartner H (2017). Generation and reactions of thiocarbonyl *S*-(2,2,2-trifluoroethanides). Synthesis of trifluoromethylated 1,3-dithiolanes, thiiranes and alkenes. J. Fluor. Chem..

[52] Shermolovich YG (2021). Reaction of *N,N-*disubstituted polyfluoroalkanethioamides with diazomethane: entry to new thiirane derivatives. Eur. J. Org. Chem..

[53] Mlostoń G, Heimgartner H (2020). Reactions for thiocarbonyl compounds with electrophilic and nucleophilic carbenes as well as with their metal complexes. J. Sulfur Chem..

[54] Im JK (2021). *N*‐Chlorinative ring contraction of 1,4‐dimethoxyphthalazines via a bicyclization/ring opening mechanism. Synthesis.

[55] Im JK, Yang B, Jeong I, Choi J-H, Chung W-j (2020). *N*-Chlorination-Induced, oxidative ring contraction of 1,4-dimethoxyphthalazines. Tetrahedron Lett..

[56] Williams WM, Dolbier WR (1969). Thermal and photochemical rearrangements of azine oxides I. A novel pyrolytic decomposition to nitrile. J. Org. Chem..

[57] Chuang KV, Xu C, Reisman SE (2016). A 15-Step synthesis of (+)-ryanodol. Science.

[58] Soldaini G, Cardona F, Goti A (2007). Catalytic oxidation of imines based on methyltrioxorhenium/urea hydrogen peroxide: a mild and easy chemo- and regioselective entry to nitrones. Org. Lett..

[59] Safari J, Gandomi-Ravandi S (2014). Structure, synthesis and application of azines: a historical perspective. RSC Adv..

[60] Karmakar R, Choudhury CR, Batten SR, Mitra S (2007). Two new copper(II) complexes with the shortest (N–N) diazine based rigid ligand: example of unusual tridentate coordination mode. J. Mol. Struct..

[61] Iashin V (2020). Metal-free C–H borylation of *N-*heteroarenes by boron trifluoride. Chem. Eur. J..

[62] Huisgen R (1984). Recent developments of the chemistry of thiocarbonyl ylides. Bull. Soc. Chim. Belg..

[63] Zhao Y, Truhlar DG (2008). Density functionals with broad applicability in chemistry. Acc. Chem. Res..

[64] Zhao Y, Truhlar DG (2008). The M06 suite of density functionals for main group thermochemistry, thermochemical kinetics, noncovalent interactions, excited states, and transition elements: two new functionals and systematic testing of four M06-class functionals and 12 other functionals. Theor. Chem. Acc..

[65] Frisch, M. J. et al. *Gaussian 16, Revision C.01* (Gaussian, Inc., Wallingford, CT, 2016).

[66] Legault, C. Y. *CYLview20* (Université de Sherbrooke, 2020).

[67] Legnani L (2016). Computational mechanistic study of thionation of carbonyl compounds with Lawesson’s reagent. J. Org. Chem..

[68] Dubau-Assibat N, Baceiredo A, Bertrand G (1995). Lawesson’s reagent: an efficient 1,3-dipole trapping agent. J. Org. Chem..

[69] Burns JM, Clark T, Williams CM (2021). Comprehensive computational investigation of the Barton–Kellogg reaction for both alkyl and aryl systems. J. Org. Chem..

