# Supplementary material for: Synthesis of cis-thiiranes as diastereoselective access to epoxide congeners via 4π-electrocyclization of thiocarbonyl ylides
Source: Nat Commun. 2022 Aug 16;13:4818. doi: 10.1038/s41467-022-32499-3 (PMC9381720; doi:10.1038/s41467-022-32499-3)
Supplement: Supplementary file 1 — Supplementary Information [file 41467_2022_32499_MOESM1_ESM.pdf]

Synthesis of *cis*-Thiiranes as Diastereoselective Access to Epoxide Congeners via 4π-Electrocyclization of Thiocarbonyl Ylides

Su-min Song, Jaeseong Jin, Jun-Ho Choi,* and Won-jin Chung*

Email: junhochoi@gist.ac.kr; wjchung@gist.ac.kr

Department of Chemistry, Gwangju Institute of Science and Technology, Gwangju 61005, Republic of Korea.

Supplementary Information

1. Supplementary Notes ··· *S2*

2. Supplementary Methods ··· *S2*

2.1. Preparation of Aldazines and Aldazine *N*-Oxides ··· *S2*

2.2. LR-Mediated Synthesis of *cis*-Thiiranes from Aldazine *N*-Oxides ··· *S13*

2.3. Mechanistic Experiments ··· *S19*

2.3.1. Trapping *trans*-Thiocarbonyl Ylide with Dipolarophiles ··· *S19*

2.3.2. Crossover Experiment with Benzaldazine and Tolualdazine *N*-Oxides ··· *S20*

2.4. Computational Study ··· *S21*

3. Supplementary Figures ··· *S23*

3.1. X-ray Crystallographic Data ··· *S23*

3.2. NMR Spectra ··· *S27*

4. Supplementary References ··· *S128*

1. Supplementary Notes

All reactions were performed in oven-dried (140 °C) or flame-dried glassware under an atmosphere of dry argon unless otherwise noted. Tetrahydrofuran (THF), diethyl ether (Et_2O), dichloromethane (CH_2Cl_2), acetonitrile (MeCN), and *N,N*-dimethylformamide (DMF) were dried by percolation through a column packed with neutral alumina and a column packed with Q5 reactant, a supported copper catalyst for scavenging oxygen, under a positive pressure of argon. *N,N*-Dimethylacetamide (DMA) and *N,N'*-dimethylpropyleneurea (DMPU) were distilled over CaH_2 under reduced pressure and stored over 3 Å molecular sieves. *N,N*-Dimethylthioformamide (DMTF, Aldrich, 98%), EtOH (Fisher, HPLC grade), and MeOH (Fisher, HPLC grade) were dried with 3 Å molecular sieves. Aldehydes were distilled under reduced pressure. Benzaldazine (*i*-PrOH), *N*-phenylmaleimide (cyclohexane), and maleic anhydride ($CHCl_3$ and hexanes) were recrystallized. Hydrazine monohydrate (Alfa, >98%), urea hydrogen peroxide (UHP, Alfa, 97%), trifluoroacetic anhydride (TFAA, Acros, >99%), Lawesson's reagent (LR, Alfa, 97%), $MeReO_3$ (Acros, 98% and Aldrich, Re 71.0-71.6%), and Na_2CO_3 (Daejung, Extra pure) were used without further purification. Solvents for workup and chromatography were hexanes (Duksan, Extra Pure), ethyl acetate (EtOAc, Duksan, Extra Pure), CH_2Cl_2 (Duksan, Extra Pure), Et_2O (Daejung, Extra Pure), pentane (Daejung, Extra Pure), and EtOH (Fisher, HPLC grade). Quenching solutions and drying reagents for workup were $MgSO_4$ (Duksan, Extra pure), K_2CO_3 (Duksan, Extra pure), and NaCl (Daejung, Extra pure).

^{1}H, ^{13}C, and ^{19}F NMR spectra were recorded on a JEOL JCX-400 spectrometer (400 MHz, ^{1}H; 100MHz, ^{13}C; 376MHz, ^{19}F). Spectra were referenced to residual chloroform (7.26 ppm, ^{1}H; 77.16 ppm, ^{13}C), dimethylsulfoxide (2.50 ppm, ^{1}H; 39.52 ppm, ^{13}C), and hexafluorobenzene (–164.9 ppm, ^{19}F). Chemical shifts were reported in ppm, and multiplicities are indicated by s (singlet), d (doublet), t (triplet), q (quartet), sep (septet), m (multiplet), and bs (broad singlet). Coupling constants, *J*, are reported in Hertz. Kugelrohr distillation was carried out using Büchi B585 glass oven with Büchi bulb-to-bulb distillation apparatus, and air bath temperatures (ABT) are reported. Filtration and column chromatography were performed using Merck 230-400 mesh silica gel. Analytical TLC was conducted on Merck silica gel 60 F_{254} TLC plates. Visualization was accomplished with UV (254 nm) as well as potassium permanganate ($KMnO_4$) and *p*-anisaldehyde staining solutions. ESI-HRMS was performed on a Bruker Impact II quadrupole-time-of-flight (Q-TOF) spectrometer at GIST Central Research Facilities (GCRF). EI-HRMS was performed on a JEOL JMS-700 MStation mass spectrometer at Korea Basic Science Institute (KBSI), Daegu Center. Data are reported in the form of *m/z*.

2. Supplementary Methods

2.1. Preparation of Aldazines and Aldazine *N*-Oxides

General procedure for preparation of aldazines[1]

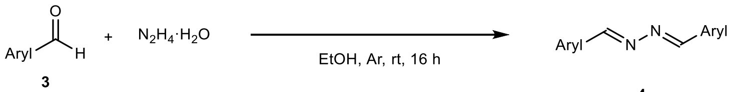

3
(1.0 equiv)
(0.5 equiv)
4

To a stirred solution of aldehyde (**3**, 10.0 mmol) in EtOH (20.0 mL) was added hydrazine monohydrate (245 µL, 5.0 mmol) at rt under Ar. After 16 h, the reaction mixture was concentrated *in vacuo.* The residue was purified by recrystallization to afford aldazine (**4**) as yellow crystals.

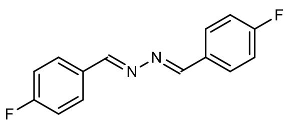

JSJ-14-012: recrystallization in EtOH, 1.14 g (94%).

Data for 4,4'-difluorobenzaldazine (**4b**):

^{1}H NMR: (400 MHz, $CDCl_3$)

δ 8.62 (s, 2H), 7.87–7.82 (m, 4H), 7.17–7.12 (m, 4H).

^{13}C NMR: (100 MHz, $CDCl_3$)

δ 164.8 (d, *J* = 252.1), 161.0, 130.7 (d, *J* = 8.6), 130.5 (d, *J* = 2.9), 116.2 (d, *J* = 22.0).

^{19}F NMR: (376 MHz, $CDCl_3$)

δ –111.3.

HRMS (ESI): $[M+H]^+$ calcd for $C_{14}H_{11}F_2N_2$: 245.0890; found: 245.0886.

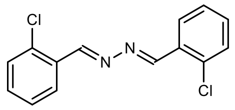

SMS-14-046: recrystallization in EtOH, 1.25 g (90%).

Data for 2,2'-dichlorobenzaldazine (**4c**):[2]

^{1}H NMR: (400 MHz, $CDCl_3$)

δ 9.09 (s, 2H), 8.24–8.21 (m, 2H), 7.45–7.33 (m, 6H).

^{13}C NMR: (100 MHz, $CDCl_3$)

δ 159.2, 136.0, 132.3, 131.6, 130.2, 128.4, 127.2.

HRMS (ESI): calcd for $C_{14}H_{11}{}^{35}Cl_2N_2$, $C_{14}H_{11}{}^{35}Cl^{37}ClN_2$, $C_{14}H_{11}{}^{37}Cl_2N_2$ $[M+H]^+$: 277.0299 (100.0%), 279.0270 (63.9%), 281.0240 (10.2%); found: 277.0296 (100.0%), 279.0268 (64.6%), 281.0239 (10.6%).

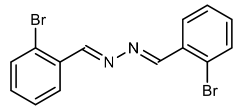

SMS-14-047: recrystallization in EtOH and 1,2-DCE, 1.68 g (92%).

Data for 2,2'-dibromobenzaldazine (**4d**):[3]

^{1}H NMR: (400 MHz, $CDCl_3$)

δ 9.04 (s, 2H), 8.23–8.20 (m, 2H), 7.65–7.62 (m, 2H), 7.42–7.38 (m, 2H), 7.34–7.30 (m, 2H).

^{13}C NMR: (100 MHz, $CDCl_3$)

δ 161.6, 133.5, 133.1, 132.6, 128.9, 127.8, 126.0.

HRMS (ESI): calcd for $C_{14}H_{11}{}^{79}Br_2N_2$, $C_{14}H_{11}{}^{79}Br^{81}BrN_2$, $C_{14}H_{11}{}^{81}Br_2N_2$ $[M+H]^+$: 364.9289 (51.4%), 366.9269 (100.0%), 368.9248 (48.6%); found: 364.9284 (51.0%), 366.9265 (100.0%), 368.9244 (49.3%).

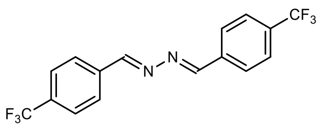

SMS-14-024: recrystallization in EtOH, 1.57 g (91%).

Data for 4,4'-bis(trifluoromethyl)benzaldazine (**4e**):[3]

^{1}H NMR:	(400 MHz, $CDCl_3$)
	δ 8.69 (s, 2H), 7.97 (d, *J* = 8.2, 4H), 7.73 (d, *J* = 8.2, 4H).
^{13}C NMR:	(100 MHz, $CDCl_3$)
	δ 161.3, 137.2, 133.0 (q, *J* = 32.6), 129.0, 125.9 (q, *J* = 3.6), 124.0 (q, *J* = 272.4).
^{19}F NMR:	(376 MHz, $CDCl_3$)
	δ –66.1.
HRMS (ESI):	$[M+H]^+$ calcd for $C_{16}H_{11}F_6N_2$: 345.0826; found: 345.0821.

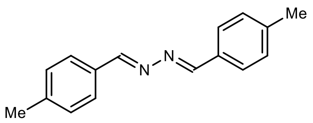

SMS-14-023: recrystallization in EtOH, 1.03 g (87%).

Data for 4,4'-dimethylbenzaldazine (**4f**):[4]

^{1}H NMR:	(400 MHz, $CDCl_3$)
	δ 8.64 (s, 2H), 7.74 (d, *J* = 8.1, 4H), 7.26 (d, *J* = 8.1, 4H), 2.41 (s, 6H).
^{13}C NMR:	(100 MHz, $CDCl_3$)
	δ 162.0, 141.8, 131.6, 129.7, 128.7, 21.8.
HRMS (ESI):	$[M+Na]^+$ calcd for $C_{16}H_{16}N_2Na$: 259.1211; found: 259.1206.

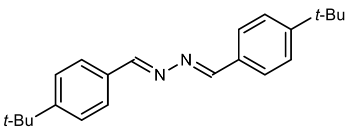

SMS-14-029: recrystallization in EtOH, 1.41 g (88%).

Data for 4,4'-di-*tert*-butylbenzaldazine (**4g**):

^{1}H NMR:	(400 MHz, $CDCl_3$)
	δ 8.65 (s, 2H), 7.79–7.76 (m, 4H), 7.48–7.46 (m, 4H), 1.35 (s, 18H).
^{13}C NMR:	(100 MHz, $CDCl_3$)
	δ 161.9, 154.8, 131.4, 128.5, 125.9, 35.2, 31.3.
HRMS (ESI):	$[M+Na]^+$ calcd for $C_{22}H_{28}N_2Na$: 343.2150; found: 343.2147.

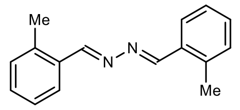

JSJ-14-007: recrystallization in EtOH, 1.05 g (89%).

Data for 2,2'-dimethylbenzaldazine (**4h**):[2]

^{1}H NMR:	(400 MHz, $CDCl_3$)
	δ 9.01 (s, 2H), 8.07–8.05 (m, 2H), 7.38–7.23 (m, 6H), 2.57 (s, 6H).
^{13}C NMR:	(100 MHz, $CDCl_3$)
	δ 160.8, 138.7, 132.3, 131.14, 131.06, 127.6, 126.4, 19.7.
HRMS (ESI):	$[M+H]^+$ calcd for $C_{16}H_{17}N_2$: 237.1392; found: 237.1384.

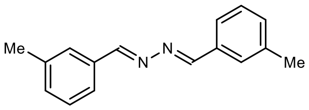

SMS-16-006: recrystallization in hexanes, 1.06 g (90%).

Data for 3,3'-dimethylbenzaldazine (**4i**):

^{1}H NMR:	(400 MHz, $CDCl_3$)
	δ 8.65 (s, 2H), 7.70 (s, 2H), 7.63–7.61 (m, 2H), 7.37–7.33 (m, 2H), 7.30–7.28 (m, 2H), 2.42 (s, 6H).
^{13}C NMR:	(100 MHz, $CDCl_3$)
	δ 162.5, 138.7, 134.1, 132.3, 128.9, 128.8, 126.3, 21.5.
HRMS (ESI):	$[M+Na]^+$ calcd for $C_{16}H_{16}N_2Na$: 259.1211; found: 259.1206.

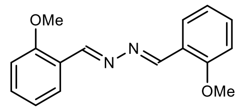

SMS-14-061: recrystallization in EtOH, 1.18 g (89%).

Data for 2,2'-dimethoxybenzaldazine (**4j**):[4]

^{1}H NMR:	(400 MHz, $CDCl_3$)
	δ 9.10 (s, 2H), 8.12 (dd, J = 7.7, 1.7, 2H), 7.42 (ddd, J = 8.4, 7.7, 1.7, 2H), 7.02 (dd, J = 7.6, 7.6, 2H), 6.94 (d, J = 8.4, 2H), 3.88 (s, 6H).
^{13}C NMR:	(100 MHz, $CDCl_3$)
	δ 159.2, 157.6, 132.5, 127.5, 123.0, 120.9, 111.3, 55.7.
HRMS (ESI):	$[M+H]^+$ calcd for $C_{16}H_{17}N_2O_2$: 269.1290; found: 269.1283.

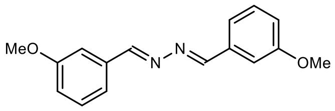

JSJ-14-081: recrystallization in hexanes and CH_2Cl_2, 1.21 g (91%).

Data for 3,3'-dimethoxybenzaldazine (**4k**):[3]

^{1}H NMR: (400 MHz, $CDCl_3$)

δ 8.64 (s, 2H), 7.46–7.45 (m, 2H), 7.38–7.36 (m, 4H), 7.05–7.00 (m, 2H), 3.88 (s, 6H).

^{13}C NMR: (100 MHz, $CDCl_3$)

δ 162.1, 160.0, 135.6, 129.9, 122.1, 118.1, 112.0, 55.5.

HRMS (ESI): $[M+H]^+$ calcd for $C_{16}H_{17}N_2O_2$: 269.1290; found: 269.1284.

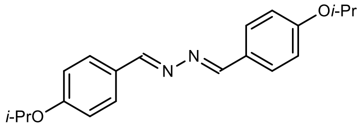

JSJ-14-016: recrystallization in EtOH, 1.50 g (92%).

Data for 4,4'-bis(isopropoxy)benzaldazine (**4l**):

^{1}H NMR: (400 MHz, $CDCl_3$)

δ 8.60 (s, 2H), 7.78–7.74 (m, 4H), 6.95–6.91 (m, 4H), 4.63 (sep, *J* = 6.1, 2H), 1.37 (d, *J* = 6.1, 12H).

^{13}C NMR: (100 MHz, $CDCl_3$)

δ 161.2, 160.6, 130.3, 126.8, 115.9, 70.1, 22.1.

HRMS (ESI): $[M+H]^+$ calcd for $C_{20}H_{25}N_2O_2$: 325.1916; found: 325.1914.

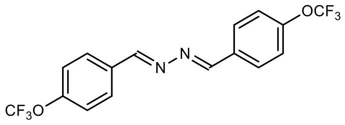

SMS-14-031: recrystallization in cold EtOH, 1.69 g (90%).

Data for 4,4'-bis(trifluoromethoxy)benzaldazine (**4m**):

^{1}H NMR: (400 MHz, $CDCl_3$)

δ 8.64 (s, 2H), 7.91–7.87 (m, 4H), 7.31–7.29 (m, 4H).

^{13}C NMR: (100 MHz, $CDCl_3$)

δ 161.0, 151.4, 132.7, 130.3, 121.2, 120.5 (q, *J* = 258.5).

^{19}F NMR: (376 MHz, $CDCl_3$)

δ –60.8.

HRMS (ESI): $[M+H]^+$ calcd for $C_{16}H_{11}F_6N_2O_2$: 377.0725; found: 377.0722.

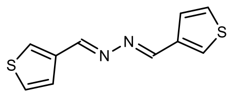

JSJ-15-018: recrystallization in EtOH, 1.01 g (92%).

Data for 3,3'-thiophenecarboxaldazine (**4n**):[2]

^{1}H NMR: (400 MHz, $CDCl_3$)

δ 8.65 (s, 2H), 7.72 (dd, *J* = 2.9, 1.1, 2H), 7.62 (dd, *J* = 5.1, 1.0, 2H), 7.37 (dd, *J* = 5.1, 2.9, 2H).

^{13}C NMR: (100 MHz, $CDCl_3$)

δ 156.4, 138.0, 129.5, 126.8, 126.0.

HRMS (ESI): $[M+H]^+$ calcd for $C_{10}H_9N_2S_2$: 221.0207; found: 221.0200.

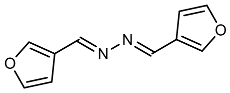

JSJ-15-037: recrystallization in EtOH, 854 mg (91%).

Data for 3,3'-furancarboxaldazine (**4o**):

^{1}H NMR: (400 MHz, $CDCl_3$)

δ 8.56 (s, 2H), 7.812–7.807 (m, 2H), 7.49–7.47 (m, 2H), 6.872–6.868 (m, 2H).

^{13}C NMR: (100 MHz, $CDCl_3$)

δ 153.8, 146.2, 144.5, 123.0, 107.9.

HRMS (ESI): $[M+H]^+$ calcd for $C_{10}H_9N_2O_2$: 189.0664; found: 189.0659.

N-Oxidation of benzaldazine with CF_3CO_3H (method A)[5]

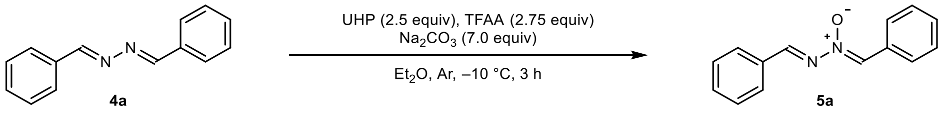

To a mixture of UHP (1.18 g, 12.5 mmol) in CH_2Cl_2 (12.5 mL) was added TFAA (1.90 mL, 13.8 mmol) at 0 °C dropwise under Ar. After 1 h, the solution was warmed to rt and stirred until the mixture turned biphasic. The upper layer (CF_3CO_3H) of the biphasic solution was directly used for *N*-oxidation. To a stirred mixture of benzaldazine (**4a**, 1.04 g, 5.00 mmol) and Na_2CO_3 (3.71 g, 35.0 mmol) in Et_2O (20.0 mL) was added the CF_3CO_3H solution at –10 °C (the ice bath was made by a mixture of ice and brine) dropwise under Ar (The change in internal temperature should not exceed 1°C when the CF_3CO_3H was added). After 3 hours, the reaction mixture was transferred to a 100 mL separatory funnel, and water and Et_2O were added to dissolve all solids. The separated organic layer was washed with 0.8 M aq. K_2CO_3 (70 mL) and water (35 mL × 3). The combined aqueous layers were extracted with Et_2O (10 mL). The combined organic layers were dried over anhydrous $MgSO_4$ (2 g), filtered, and concentrated *in vacuo*. The residue was purified by recrystallization or flash column chromatography on silica gel to give benzaldazine *N*-oxide (**5a**).

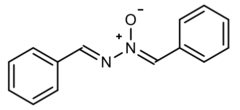

SMS-14-090 Recrystallization (hexanes) afforded **5a** (886 mg, 80%) as pale yellow crystals.

Data for benzaldazine *N*-oxide (**5a**):[6]

^{1}H NMR: (400 MHz, $CDCl_3$)

δ 9.43 (s, 1H), 8.33–8.29 (m, 2H), 7.94–7.91(m, 2H), 7.88 (s, 1H), 7.57–7.45 (m, 6H).

^{13}C NMR: (100 MHz, $CDCl_3$)

δ 156.5, 133.3, 132.6, 131.2, 131.1, 130.1, 130.0, 129.8, 129.2, 128.9.

General procedure for *N*-oxidation of aldazines with UHP and $MeReO_3$ (method B)[7]

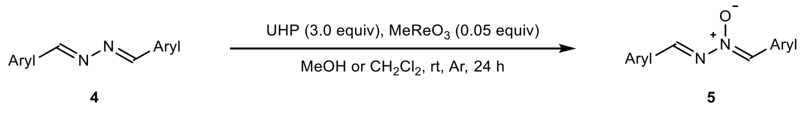

To a stirred mixture aldazine (**4**, 3.00 mmol) and UHP (847 mg, 9.00 mmol) in MeOH or CH_2Cl_2 (12.0 mL) was added $MeReO_3$ (37 mg, 0.15 mmol) at rt. After 24 hours, the reaction mixture was concentrated *in vacuo*, and the residue was filtered through a pad of silica gel (SiO_2, ϕ = 3.0 cm, l = 3.0 cm) immediately with CH_2Cl_2 to remove urea. The filtrate was concentrated *in vacuo*, and the residue was purified by recrystallization or flash column chromatography on silica gel to give aldazine *N*-oxide (**5**).

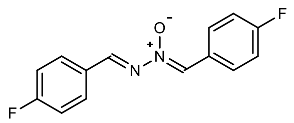

SMS-15-052: reaction in CH_2Cl_2; Flash column chromatography (SiO_2, ϕ = 4.5 cm, l = 15 cm, CH_2Cl_2/hexanes = 1/1 (CH_2Cl_2 loading), R_f = 0.30, $KMnO_4$) afforded **5b** (636 mg, 81%) as pale yellow solid.

Data for 4,4'-difluorobenzaldazine *N*-oxide (**5b**):

^{1}H NMR: (400 MHz, $CDCl_3$)

δ 9.37 (s, 1H), 8.36–8.31 (m, 2H), 7.95–7.90 (m, 2H), 7.83 (s, 1H), 7.22–7.16 (m, 4H).

^{13}C NMR: (100 MHz, $CDCl_3$)

δ 165.5 (d, J = 254.5), 164.0 (d, J = 254.2), 155.2, 132.2 (d, J = 9.0), 132.1, 131.9 (d, J = 8.4), 127.5 (d, J = 3.1), 126.4 (d, J = 3.2), 116.5 (d, J = 22.1), 116.2 (d, J = 21.9).

^{19}F NMR: (376 MHz, $CDCl_3$)

δ –108.8, –109.8.

HRMS (ESI): $[M+Na]^+$ calcd for $C_{14}H_{10}F_2N_2ONa$: 283.0659; found: 283.0650.

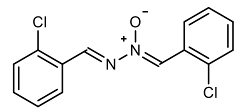

SMS-14-096: reaction in CH_2Cl_2; Flash column chromatography (SiO_2, ϕ = 3.5 cm, *l* = 15 cm, CH_2Cl_2/hexanes = 1/2, R_f = 0.32, $KMnO_4$) afforded **5c** (481 mg, 55%) as yellow solid.

Data for 2,2'-dichlorobenzaldazine *N*-oxide (**5c**):

^{1}H NMR: (400 MHz, $CDCl_3$)
δ 9.88 (s, 1H), 9.31–9.29 (m, 1H), 8.37 (s, 1H), 8.22–8.20 (m, 1H), 7.51–7.36 (m, 6H).

^{13}C NMR: (100 MHz, $CDCl_3$)
δ 154.2, 137.5, 134.6, 133.6, 131.8, 130.5, 130.1, 129.9, 129.7, 129.3, 128.9, 127.8, 127.4, 127.3.

HRMS (ESI): calcd for $C_{14}H_{10}{}^{35}Cl_2N_2ONa$, $C_{14}H_{10}{}^{35}Cl^{37}ClN_2ONa$, $C_{14}H_{10}{}^{37}Cl_2N_2ONa$ $[M+Na]^+$: 315.0068 (100.0%), 317.0038 (63.9%), 319.0009 (10.2%); found: 315.0059 (100.0%), 317.0031 (66.7%), 319.0001 (10.8%).

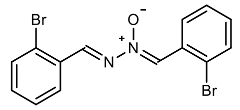

SMS-14-097: reaction in CH_2Cl_2; Flash column chromatography (SiO_2, ϕ = 3.5 cm, *l* = 15 cm, CH_2Cl_2/hexanes = 1/2, R_f = 0.28, $KMnO_4$) afforded **5d** (420 mg, 37%) as yellow solid.

Data for 2,2'-dibromobenzaldazine *N*-oxide (**5d**):

^{1}H NMR: (400 MHz, $CDCl_3$)
δ 9.84 (s, 1H), 9.31–9.28 (m, 1H), 8.35 (s, 1H), 8.22–8.20 (m, 1H), 7.69–7.67 (m, 2H), 7.50–7.35 (m, 3H), 7.32–7.28 (m, 1H).

^{13}C NMR: (100 MHz, $CDCl_3$)
δ 156.7, 133.82, 133.78, 133.4, 132.3, 132.1, 130.52, 130.48, 129.7, 129.3, 127.97, 127.94, 127.6, 125.0.

HRMS (ESI): calcd for $C_{14}H_{10}{}^{79}Br_2N_2ONa$, $C_{14}H_{10}{}^{79}Br^{81}BrN_2ONa$, $C_{14}H_{10}{}^{81}Br_2N_2ONa$ $[M+Na]^+$: 402.9058 (51.4%), 404.9037 (100.0%), 406.9017 (48.6%); found: 402.9052 (51.1%), 404.9033 (100.0%), 406.9011 (49.5%).

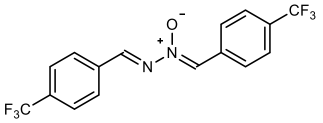

SMS-15-035: reaction in CH_2Cl_2; Flash column chromatography (SiO_2, ϕ = 4.5 cm, *l* = 15 cm, CH_2Cl_2/hexanes = 1/2, R_f = 0.31, $KMnO_4$) afforded **5e** (599 mg, 55%) as pale yellow solid.

Data for 4,4'-bis(trifluoromethyl)benzaldazine *N*-oxide (**5e**):

^{1}H NMR: (400 MHz, $CDCl_3$)
δ 9.45 (s, 1H), 8.42–8.40 (m, 2H), 8.04–8.02 (m, 2H), 7.94 (s, 1H), 7.77–7.73 (m, 4H).

^{13}C NMR: (100 MHz, $CDCl_3$)

δ 156.2, 134.25 (q, *J* = 32.8), 134.17, 133.1, 132.4 (q, *J* = 32.8), 132.3, 130.3, 129.8, 126.2 (q, *J* = 3.6), 125.8 (q, *J* = 3.6), 123.8 (q, *J* = 272.3), 123.7 (q, *J* = 272.6).

^{19}F NMR: (376 MHz, $CDCl_3$)

δ –66.2, –66.3.

HRMS (ESI): $[M+H]^+$ calcd for $C_{16}H_{11}F_6N_2O$: 361.0776; found: 361.0770.

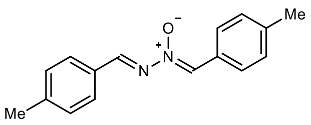

SMS-15-032: reaction in CH_2Cl_2; Flash column chromatography (SiO_2, ϕ = 4.0 cm, *l* = 15 cm, CH_2Cl_2/hexanes = 1/1, R_f = 0.26, $KMnO_4$) afforded **5f** (556 mg, 73%) as pale yellow solid.

Data for 4,4’-dimethylbenzaldazine *N*-oxide (**5f**):

^{1}H NMR: (400 MHz, $CDCl_3$)

δ 9.38 (s, 1H), 8.21–8.19 (m, 2H), 7.82–7.79 (m, 3H), 7.31–7.28 (m, 4H), 2.43 (s, 3H), 2.42 (s, 3H).

^{13}C NMR: (100 MHz, $CDCl_3$)

δ 156.0, 143.2, 141.7, 133.1, 129.9, 129.8, 129.6, 128.7, 127.5, 21.89, 21.88.

HRMS (ESI): $[M+Na]^+$ calcd for $C_{16}H_{16}N_2ONa$: 275.1160; found: 275.1155.

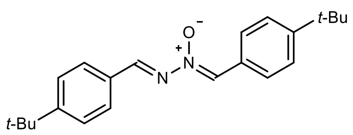

SMS-15-048: reaction in CH_2Cl_2; Flash column chromatography (SiO_2, ϕ = 3.5 cm, *l* = 15 cm, CH_2Cl_2/hexanes = 1/1, R_f = 0.37, $KMnO_4$) afforded **5g** (601 mg, 59%) as yellow solid.

Data for 4,4’-di-*tert*-butylbenzaldazine *N*-oxide (**5g**):

^{1}H NMR: (400 MHz, $CDCl_3$)

δ 9.40 (s, 1H), 8.26–8.24 (m, 2H), 7.86–7.84 (m, 2H), 7.84 (s, 1H), 7.54–7.50 (m, 4H), 1.36 (s, 18H).

^{13}C NMR: (100 MHz, $CDCl_3$)

δ 156.3, 155.9, 154.7, 133.0, 129.8, 129.7, 128.6, 127.5, 126.2, 125.9, 35.3, 35.2, 31.2 (2C).

HRMS (ESI): $[M+Na]^+$ calcd for $C_{22}H_{28}N_2ONa$: 359.2099; found: 359.2095.

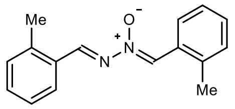

SMS-14-066: reaction in MeOH; Recrystallization (hexanes) afforded **5h** (498 mg, 66%) as yellow crystals.

Data for 2,2’-dimethylbenzaldazine *N*-oxide (**5h**):

^{1}H NMR: (400 MHz, $CDCl_3$)

δ 9.75 (s, 1H), 9.21–9.19 (m, 1H), 8.07 (s, 1H), 8.07–8.05 (m, 1H), 7.44–7.26 (m, 6H), 2.62 (s, 3H), 2.51 (s, 3H).

^{13}C NMR: (100 MHz, $CDCl_3$)

δ 154.9, 140.4, 138.1, 132.3, 131.4, 130.9, 130.6, 130.5, 129.5, 128.85, 128.79, 128.5, 126.7, 126.6, 20.1, 19.9.

HRMS (ESI): $[M+Na]^+$ calcd for $C_{16}H_{16}N_2ONa$: 275.1160; found: 275.1156.

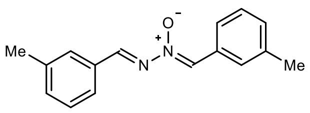

SMS-16-007: reaction in CH_2Cl_2; Flash column chromatography (SiO_2, ϕ = 3.5 cm, *l* = 15 cm, CH_2Cl_2/hexanes = 1/1, R_f = 0.22, $KMnO_4$) afforded **5i** (564 mg, 75%) as yellow solid.

Data for 3,3'-dimethylbenzaldazine *N*-oxide (**5i**):

^{1}H NMR: (400 MHz, $CDCl_3$)

δ 9.38 (s, 1H), 8.18 (s, 1H), 8.07–8.05 (m, 1H), 7.84 (s, 1H), 7.75 (s, 1H), 7.71–7.70 (m, 1H), 7.41–7.34 (m, 3H), 7.31–7.29 (m, 1H), 2.44 (s, 6H).

^{13}C NMR: (100 MHz, $CDCl_3$)

δ 156.6, 139.0, 138.6, 133.5 (2C), 132.1, 131.2, 130.3, 130.2, 130.0, 129.1, 128.8, 127.4, 127.2, 21.6, 21.4.

HRMS (ESI): $[M+Na]^+$ calcd for $C_{16}H_{16}N_2ONa$: 275.1160; found: 275.1154.

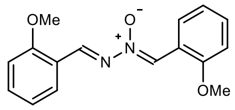

SMS-15-042: reaction in MeOH (9 mL) and CH_2Cl_2 (3 mL); Flash column chromatography (SiO_2, ϕ = 4.5 cm, *l* = 15 cm, CH_2Cl_2/hexanes = 2/1, R_f = 0.34, $KMnO_4$) and recrystallization (hexanes and 1,2-DCE) afforded **5j** (522 mg, 61%) as yellow crystals.

Data for 2,2'-dimethoxybenzaldazine *N*-oxide (**5j**):

^{1}H NMR: (400 MHz, $CDCl_3$)

δ 9.83 (s, 1H), 9.24–9.21 (m, 1H), 8.31 (s, 1H), 8.10–8.08 (m, 1H), 7.51–7.46 (m, 1H), 7.44–7.40 (m, 1H), 7.12–6.92 (m, 4H), 3.92 (s, 6H).

^{13}C NMR: (100 MHz, $CDCl_3$)

δ 160.1, 158.2, 152.0, 133.9, 132.1, 129.6, 128.4, 127.9, 121.0, 120.9, 120.1, 119.4, 111.5, 110.1, 55.7 (2C).

HRMS (ESI): $[M+Na]^+$ calcd for $C_{16}H_{16}N_2O_3Na$: 307.1059; found: 307.1055.

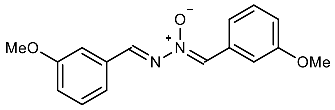

JSJ-14-092: reaction in CH_2Cl_2; Flash column chromatography (SiO_2, ϕ = 3.5 cm, *l* = 15 cm, EtOAc/hexanes = 1/6, R_f = 0.30, $KMnO_4$) and recrystallization (hexanes and CH_2Cl_2) afforded **5k** (583 mg, 68%) as orange solid.

Data for 3,3’-dimethoxybenzaldazine *N*-oxide (**5k**):

^{1}H NMR: (400 MHz, DMSO-d_6)

δ 9.39 (s, 1H), 8.18 (s, 1H), 8.10 (s, 1H), 7.90–7.88 (m, 1H), 7.59–7.56 (m, 2H), 7.50–7.43 (m, 2H), 7.21–7.18 (m, 1H), 7.12–7.09 (m, 1H), 3.84 (s, 3H), 3.82 (s, 3H).

^{13}C NMR: (100 MHz, DMSO-d_6)

δ 159.6, 159.1, 156.6, 132.2, 132.0, 131.2, 130.4, 129.8, 122.6, 122.2, 119.1, 116.9, 114.0, 113.8, 55.3, 55.2.

HRMS (ESI): $[M+Na]^+$ calcd for $C_{16}H_{16}N_2O_3Na$: 307.1059; found: 307.1054.

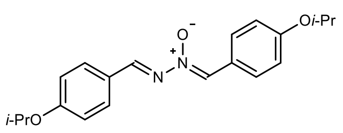

JSJ-14-086: reaction in CH_2Cl_2; Recrystallization (hexanes and CH_2Cl_2) afforded **5l** (744 mg, 73%) as yellow crystals.

Data for 4,4’-bis(isopropoxy)benzaldazine *N*-oxide (**5l**):

^{1}H NMR: (400 MHz, $CDCl_3$)

δ 9.32 (s, 1H), 8.27–8.25 (m, 2H), 7.84–7.81 (m, 2H), 7.75 (s, 1H), 6.99–6.94 (m, 4H), 4.69–4.62 (m, 2H), 1.37 (d, *J* = 6.1, 12H).

^{13}C NMR: (100 MHz, $CDCl_3$)

δ 161.6, 160.2, 154.8, 132.5, 131.7 (2C), 123.8, 122.7, 116.1, 115.9, 70.3, 70.2, 22.13, 22.10.

HRMS (ESI): $[M+Na]^+$ calcd for $C_{20}H_{24}N_2O_3Na$: 363.1685; found: 363.1681.

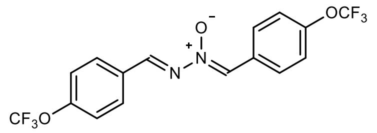

SMS-14-095: reaction in MeOH (9 mL) and CH_2Cl_2 (3 mL); Recrystallization (hexanes) afforded **5m** (900 mg, 77%) as pale yellow crystals.

Data for 4,4’-bis(trifluoromethoxy)benzaldazine *N*-oxide (**5m**):

^{1}H NMR: (400 MHz, $CDCl_3$)

δ 9.39 (s, 1H), 8.38–8.34 (m, 2H), 7.98–7.94 (m, 2H), 7.86 (s, 1H), 7.34–7.32 (m, 4H).

^{13}C NMR: (100 MHz, $CDCl_3$)

δ 155.4, 152.4, 150.7, 132.0, 131.7, 131.4, 129.5, 128.5, 121.2, 121.0, 120.51 (q, *J* = 258.4), 120.46 (q, *J* = 258.4).

^{19}F NMR: (376 MHz, $CDCl_3$)

δ –60.8.

HRMS (ESI): $[M+H]^+$ calcd for $C_{16}H_{11}F_6N_2O_3$: 393.0674; found: 393.0670.

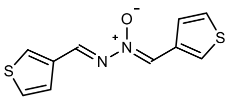

JSJ-15-024: reaction in CH_2Cl_2; Flash column chromatography (SiO_2, ϕ = 3.5 cm, *l* = 15 cm, CH_2Cl_2/hexanes = 1/1 to 2/1, R_f = 0.18 in CH_2Cl_2/hexanes = 1/1, $KMnO_4$) and recrystallization (CH_2Cl_2 and hexanes) afforded **5n** (394 mg, 56%) as yellow solid.

Data for 3,3'-dithiophenecarboxaldazine *N*-oxide (**5n**):

^{1}H NMR: (400 MHz, $CDCl_3$)
δ 9.41 (s, 1H), 8.85–8.84 (m, 1H), 7.93–7.92 (m, 1H), 7.97 (s, 1H), 7.63–7.62 (m, 1H), 7.49–7.47 (m, 1H), 7.43–7.39 (m, 2H).

^{13}C NMR: (100 MHz, $CDCl_3$)
δ 150.5, 134.7, 133.1, 131.4, 130.2, 128.3, 127.8, 127.4, 126.1, 126.0.

HRMS (ESI): $[M+H]^+$ calcd for $C_{10}H_9N_2S_2O$: 237.0156; found: 237.0151.

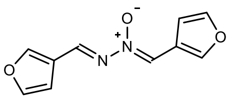

JSJ-15-051: UHP (18.0 mmol) and $MeReO_3$ (0.30 mmol), reaction in CH_2Cl_2; Flash column chromatography twice (1st: SiO_2, ϕ = 4.0 cm, *l* = 15 cm, EtOAc/hexanes = 1/6, 2nd: SiO_2, ϕ = 3.0 cm, *l* = 15 cm, EtOAc/hexanes = 1/6, R_f = 0.30, *p*-anisaldehyde) afforded **5o** (230 mg, 38%) as pale yellow solid.

Data for 3,3'-furancarboxaldazine *N*-oxide (**5o**):

^{1}H NMR: (400 MHz, $CDCl_3$)
δ 9.30 (s, 1H), 8.792–8.788 (m, 1H), 7.96–7.95 (m, 1H), 7.71 (s, 1H), 7.52–7.51 (m, 2H), 6.87–6.86 (m, 1H), 6.693–6.686 (m, 1H).

^{13}C NMR: (100 MHz, $CDCl_3$)
δ 148.6, 148.2, 146.7, 145.0, 143.6, 125.9, 120.4, 117.1, 109.9, 108.1.

HRMS (ESI): $[M+H]^+$ calcd for $C_{10}H_9N_2O_3$: 205.0613; found: 205.0608.

2.2. LR-Mediated Synthesis of *cis*-Thiiranes from Aldazine *N*-Oxides

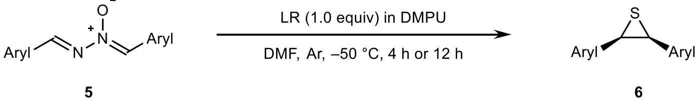

To a stirred mixture of aldazine *N*-oxide (**5**, 1.00 mmol) in DMF (5.0 mL) was added a solution of LR (404 mg, 1.00 mmol) in DMPU (5.0 mL) dropwise at –50 °C under Ar. (The change in internal temperature should not exceed 1 °C when the LR solution was added) After 4 or 12 h, the reaction mixture was transferred to a 100 mL separatory funnel and diluted with CH_2Cl_2 (4 mL). The mixture was washed with water (50 mL × 3) and brine (50 mL). The combined aqueous layers were extracted with CH_2Cl_2 (3 mL × 6). The combined organic layers were dried over anhydrous $MgSO_4$ (2 g), filtered, and concentrated *in vacuo*. The residue was purified immediately by flash column chromatography to give *cis*-thiirane (**6**) as white solid or a colorless oil. The *cis*:*trans* ratio was determined by ^{1}H NMR analysis of the benzylic protons. (When the reaction performed at –10 °C, the ice bath was made by a mixture of ice and brine.)

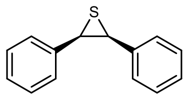

SMS-15-044, 4 h; Flash column chromatography (SiO_2, ϕ = 3.5 cm, l = 5.0 cm, hexanes, R_f = 0.20, $KMnO_4$) afforded **6a** (165 mg, 78%, *cis*:*trans* = 99:1) as white solid.

Data for *cis*-2,3-diphenylthiirane (**6a**):[8]

^{1}H NMR: (400 MHz, $CDCl_3$)
δ 7.14–7.10 (m, 10H), 4.38 (s, 2H). (3.96 (s, 0.01H) for *trans*-**6a**)

^{13}C NMR: (100 MHz, $CDCl_3$)
δ 135.2, 129.5, 127.8, 127.3, 44.2.

HRMS (ESI): $[M–H]^–$ calcd for $C_{14}H_{11}S$: 211.0587; found: 211.0586.

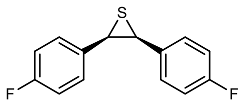

SMS-15-030, 12 h; Flash column chromatography (SiO_2, ϕ = 4.0 cm, l = 5.0 cm, hexanes, R_f = 0.32, $KMnO_4$) afforded **6b** (182 mg, 73%, *cis*:*trans* = 99:1) as white solid.

Data for *cis*-2,3-bis(4-fluorophenyl)thiirane (**6b**):

^{1}H NMR: (400 MHz, $CDCl_3$)
δ 7.10–7.05 (m, 4H), 6.85–6.79 (m, 4H), 4.31 (s, 2H). (3.87 (s, 0.02H) for *trans*-**6b**)

^{13}C NMR: (100 MHz, $CDCl_3$)
δ162.1 (d, J = 246.3), 131.0 (d, J = 7.7), 130.7 (d, J = 2.9), 114.8 (d, J = 21.1), 43.3.

^{19}F NMR: (376 MHz, $CDCl_3$)
δ –118.2.

HRMS (ESI): $[M–H]^–$ calcd for $C_{14}H_9F_2S$: 247.0399; found: 247.0397.

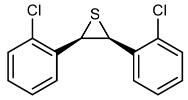

SMS-15-024, 12 h; Flash column chromatography (SiO_2, ϕ = 3.5 cm, l = 7.0 cm, hexanes, R_f = 0.46, $KMnO_4$) afforded **6c** (195 mg, 70%, *cis*:*trans* = 98:2) as white solid.

Data for *cis*-2,3-bis(2-chlorophenyl)thiirane (**6c**):

^{1}H NMR: (400 MHz, $CDCl_3$)
δ 7.22–7.19 (m, 2H),7.16–7.14 (m, 2H), 7.08–7.04 (m, 2H), 7.01–6.97 (m, 2H), 4.71 (s, 2H). (4.34 (s, 0.04H) for *trans*-**6c**)

^{13}C NMR: (100 MHz, $CDCl_3$)
δ 135.5, 132.5, 131.1, 129.0, 128.7, 126.0, 42.4.

HRMS (EI): calcd for $C_{14}H_{10}{}^{35}Cl_2S$, $C_{14}H_{10}{}^{35}Cl^{37}ClS$, $C_{14}H_{10}{}^{37}Cl_2S$ $[M]^+$: 279.9880 (100.0%), 281.9852 (69.6%), 283.9824 (13.9%); found: 279.9879 (100.0%), 281.9850 (69.0%), 283.9818 (17.7%).

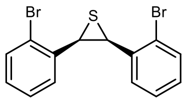

SMS-15-039, 12 h; Flash column chromatography (SiO_2, ϕ = 3.5 cm, l = 5.0 cm, hexanes, R_f = 0.40, $KMnO_4$) afforded **6d** (216 mg, 59%, *cis*:*trans* = 99:1) as white solid.

Data for *cis*-2,3-bis(2-bromophenyl)thiirane (**6d**):

^{1}H NMR: (400 MHz, $CDCl_3$)

δ 7.42–7.40 (m, 2H), 7.15–7.13 (m, 2H), 7.06–6.97 (m, 4H), 4.69 (s, 2H). (4.31(s, 0.02H) for *trans*-**6d**)

^{13}C NMR: (100 MHz, $CDCl_3$)

δ 134.1, 132.4, 131.3, 129.0, 126.6, 125.9, 44.8.

HRMS (EI): calcd for $C_{14}H_{10}{}^{79}Br_2S$, $C_{14}H_{10}{}^{79}Br^{81}BrS$, $C_{14}H_{10}{}^{81}Br_2S$ $[M]^+$: 367.8870 (49.9%), 369.8850 (100.0%), 371.8829 (52.8%); found: 367.8872 (48.8%), 369.8843 (100.0%), 371.8828 (55.7%).

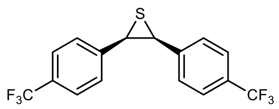

SMS-15-049, 12 h; Flash column chromatography (SiO_2, ϕ = 3.5 cm, l = 5.0 cm, hexanes, R_f = 0.21, $KMnO_4$) afforded **6e (**220 mg, 63%, *cis*:*trans* ≥ 99:1) as a colorless oil.

Data for *cis*-2,3-bis(4-trifluoromethylphenyl)thiirane (**6e**):

^{1}H NMR: (400 MHz, $CDCl_3$)

δ 7.41–7.39 (m, 4H), 7.25–7.23 (m, 4H), 4.42 (s, 2H). (3.95 (s, 0.01H) for *trans*-**6e**)

^{13}C NMR: (100 MHz, $CDCl_3$)

δ 138.8, 129.9 (q, J = 32.6), 129.8, 125.0 (q, J = 3.8), 124.1 (q, J = 271.9), 43.3.

^{19}F NMR: (376 MHz, $CDCl_3$)

δ –65.8.

HRMS (ESI): $[M–H]^-$ calcd for $C_{16}H_9F_6S$: 347.0335; found: 347.0337.

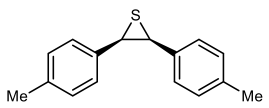

SMS-15-037, 4 h; Flash column chromatography (SiO_2, ϕ = 3.5 cm, l = 5.0 cm, hexanes, R_f = 0.29, $KMnO_4$) afforded **6f** (206 mg, 86%, *cis*:*trans* = 99:1) as white solid.

Data for *cis*-2,3-bis(4-methylphenyl)thiirane (**6f**):

^{1}H NMR: (400 MHz, $CDCl_3$)

δ 7.03–7.01 (m, 4H), 6.95–6.92 (m, 4H), 4.32 (s, 2H), 2.24 (s, 6H). (3.92 (s, 0.03H) for *trans*-**6f**)

^{13}C NMR: (100 MHz, $CDCl_3$)

δ 136.9, 132.3, 129.4, 128.5, 44.2, 21.2.

HRMS (ESI): $[M+H]^+$ calcd for $C_{16}H_{17}S$: 241.1051; found: 241.1042.

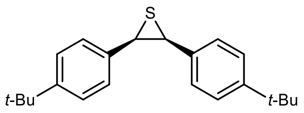

SMS-15-027, 4 h; Flash column chromatography (SiO_2, ϕ = 3.5 cm, l = 5.0 cm, hexanes, R_f = 0.24, $KMnO_4$) afforded **6g** (274 mg, 84%, *cis*:*trans* ≥ 99:1) as white solid.

Data for *cis*-2,3-bis(4-*tert*-butylphenyl)thiirane (**6g**):

^{1}H NMR: (400 MHz, $CDCl_3$)

δ 7.14–7.12 (m, 4H), 7.05–7.03 (m, 4H), 4.33 (s, 2H), 1.22 (s, 18H). (3.96 (s, 0.01H) for *trans*-**6g**)

^{13}C NMR: (100 MHz, $CDCl_3$)

δ 150.2, 132.4, 129.2, 124.7, 44.0, 34.5, 31.4.

HRMS (ESI): $[M+H]^+$ calcd for $C_{22}H_{29}S$: 325.1990; found: 325.1983.

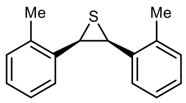

SMS-15-023, 4 h; Flash column chromatography (SiO_2, ϕ = 3.5 cm, l = 5.0 cm, hexanes, R_f = 0.24, $KMnO_4$) afforded **6h** (218 mg, 91%, *cis*:*trans* ≥ 99:1) as white solid.

Data for *cis*-2,3-bis(2-methylphenyl)thiirane (**6h**):

^{1}H NMR: (400 MHz, $CDCl_3$)

δ 7.08–6.91 (m, 8H), 4.49 (s, 2H), 2.34 (s, 6H). (4.20 (s, 0.01H) for *trans*-**6h**)

^{13}C NMR: (100 MHz, $CDCl_3$)

δ 137.1, 133.2, 129.6, 129.5, 127.3, 125.4, 42.7, 19.8.

HRMS (ESI): $[M–H]^–$ calcd for $C_{16}H_{15}S$: 239.0900; found: 239.0898.

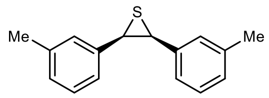

SMS-16-008, 4 h; Flash column chromatography twice (1st: SiO_2, ϕ = 3.5 cm, l = 5.0 cm, hexanes, 2nd: SiO_2, ϕ = 2.0 cm, l = 5.0 cm, hexanes, R_f = 0.21, $KMnO_4$) afforded **6i** (171 mg, 71%, *cis*:*trans* = 98:2) as a colorless oil

Data for *cis*-2,3-bis(3-methylphenyl)thiirane (**6i**):

^{1}H NMR: (400 MHz, $CDCl_3$)

δ 7.02–6.99 (m, 4H), 6.94–6.89 (m, 4H), 4.33 (s, 2H), 2.22 (s, 6H). (3.94 (s, 0.04H) for *trans*-**6i**)

^{13}C NMR: (100 MHz, $CDCl_3$)

δ 137.3, 135.2, 130.4, 128.0, 127.6, 126.5, 44.2, 21.4.

HRMS (ESI): $[M+H]^+$ calcd for $C_{16}H_{17}S$: 241.1051; found: 241.1045.

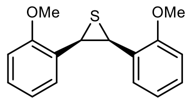

SMS-15-047, 12 h; Flash column chromatography (SiO_2, ϕ = 3.5 cm, l = 10 cm, hexanes to EtOAc/hexanes = 1/20, R_f = 0.29 in EtOAc/hexanes = 1/20, $KMnO_4$) afforded **6j** (203 mg, 75%, *cis*:*trans* = 96:4) as pale purple solid, and recrystallization (cold pentane) afforded **6j** (164 mg, 60%, *cis*:*trans* ≥ 99:1) as colorless crystals.

Data for *cis*-2,3-bis(2-methoxyphenyl)thiirane (**6j**):

^{1}H NMR: (400 MHz, $CDCl_3$)
δ 7.09–7.02 (m, 4H), 6.68–6.65 (m, 4H), 4.60 (s, 2H), 3.71 (s, 6H). (4.40 (s) for *trans*-**6j**)

^{13}C NMR: (100 MHz, $CDCl_3$)
δ 158.5, 130.5, 128.3, 124.2, 119.7, 109.6, 55.5, 40.4.

HRMS (ESI): $[M+H]^+$ calcd for $C_{16}H_{17}O_2S$: 273.0949; found: 273.0942.

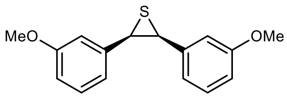

JSJ-15-014, 12 h; Flash column chromatography (SiO_2, ϕ = 4.0 cm, l = 15 cm, hexanes to EtOAc/hexanes = 1/25, R_f = 0.27 in EtOAc/hexanes = 1/25, $KMnO_4$) afforded **6k** (194 mg, 71%, *cis*:*trans* = 97:3) as a pale brown oil, and recrystallization (pentane) and flash column chromatography of the mother liquor (SiO_2, ϕ = 2.0 cm, l = 15 cm, EtOAc/hexanes = 1/25, $KMnO_4$) afforded **6k** (164 mg, 60%, *cis*:*trans* ≥ 99:1) as white solid.

Data for *cis*-2,3-bis(3-methoxyphenyl)thiirane (**6k**):

^{1}H NMR: (400 MHz, $CDCl_3$)
δ 7.08–7.04 (m, 2H), 6.82–6.79 (m, 2H), 6.70–6.67 (m, 4H), 4.35 (s, 2H), 3.63 (s, 6H). (3.88 (s) for *trans*-**6k**)

^{13}C NMR: (100 MHz, $CDCl_3$)
δ 159.1, 136.9, 128.8, 122.1, 114.6, 113.4, 55.2, 44.0.

HRMS (ESI): $[M+H]^+$ calcd for $C_{16}H_{17}O_2S$: 273.0949; found: 273.0942.

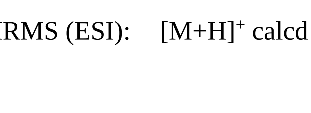

SMS-15-046, 12 h; Flash column chromatography twice (SiO_2, ϕ = 3.5 cm, l = 5.0 cm, hexanes, R_f = 0.17, $KMnO_4$) afforded **6l** (257 mg, 78%, *cis*:*trans* = 99:1, thiirane (**6l**) :alkene (**7**) = 86:14) as a yellow opaque oil.

Data for *cis*-2,3-bis(4-isopropoxyphenyl)thiirane (**6l**):

^{1}H NMR: (400 MHz, $CDCl_3$)
δ 7.03–6.99 (m, 4H), 6.67–6.63 (m, 4H), 4.44 (sep, J = 6.1, 2H), 4.28 (s, 2H), 1.27 (d, J = 6.1, 12H). (3.90 (s, 0.03H) for *trans*-**6l**)

HRMS (ESI): $[M+H]^+$ calcd for $C_{20}H_{25}O_2S$: 329.1575; found: 329.1569.

Data for 1,2-bis(4-isopropoxyphenyl)ethene (**7**):

^{1}H NMR: (400 MHz, $CDCl_3$)

trans-**7**: δ 7.42–7.38 (m, 4H), 6.91 (s, 2H), 6.88–6.85 (m, 4H), 4.59–4.53 (m, 2H), 1.35 (d, *J* = 6.1, 12H); *cis*-**7**: δ 7.20–7.18 (m, 4H), 6.75–6.73 (m, 4H), 6.42 (s, 2H), 4.55–4.49 (m, 2H), 1.33 (d, *J* = 6.1, 12H).

HRMS (ESI): $[M+H]^+$ calcd for $C_{20}H_{25}O_2$: 297.1855; found: 297.1845.

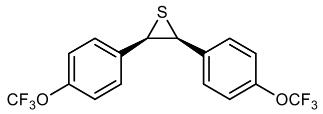

SMS-15-055: 12 mL of DMF was used. The addition of LR solution was performed at –40 °C because the reaction mixture was frozen at –50 °C. After addition of LR solution, the reaction mixture was allowed to cool to –50 °C. The reaction time was 12 h; Flash column chromatography (SiO_2, ϕ = 3.5 cm, *l* = 5.0 cm, hexanes, R_f = 0.27, $KMnO_4$) afforded **6m** (264 mg, 69%, *cis*:*trans* = 99:1) as a colorless oil.

Data for *cis*-2,3-bis(4-trifluoromethoxyphenyl)thiirane (**6m**):

^{1}H NMR: (400 MHz, $CDCl_3$)

δ 7.14–7.11 (m, 4H), 6.99–6.97 (m, 4H), 4.35 (s, 2H). (3.89 (s, 0.01H) for *trans*-**6m**)

^{13}C NMR: (100 MHz, $CDCl_3$)

δ 148.6, 133.6, 130.8, 120.5 (q, *J* = 257.2), 120.4, 43.0.

^{19}F NMR: (376 MHz, $CDCl_3$)

δ –61.2.

HRMS (ESI): $[M–H]^–$ calcd for $C_{16}H_9F_6O_2S$: 379.0233; found: 379.0228.

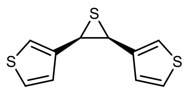

JSJ-15-035, 4 h; Flash column chromatography twice (1st: SiO_2, ϕ = 3.0 cm, *l* = 5.0 cm, hexanes, 2nd: SiO_2, ϕ = 2.5 cm, *l* = 15 cm, hexanes, R_f = 0.30, $KMnO_4$) afforded **6n** (144 mg, 64%, *cis*:*trans* ≥ 99:1) as white solid.

Data for *cis*-2,3-bis(3-thienyl)thiirane (**6n**):

^{1}H NMR: (400 MHz, $CDCl_3$)

δ 7.10 (dd, *J* = 5.0, 3.1, 2H), 7.05 (dd, *J* = 3.1, 1.3, 2H), 6.75 (dd, *J* = 5.0, 1.3, 2H), 4.27 (s, 2H). (4.08 (s, 0.01H) for *trans*-**6n**)

^{13}C NMR: (100 MHz, $CDCl_3$)

δ 137.2, 128.3, 125.3, 124.3, 39.0.

HRMS (EI): $[M]^+$ calcd for $C_{10}H_8S_3$: 223.9788; found: 223.9788.

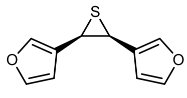

JSJ-15-055, 4 h; Flash column chromatography twice (1st: SiO_2, ϕ = 3.0 cm, *l* = 5.0 cm, hexanes, 2nd: SiO_2, ϕ = 2.0 cm, *l* = 10 cm, hexanes, R_f = 0.24, $KMnO_4$) afforded **6o** (110 mg, 57%, *cis*:*trans* = 98:2) as a colorless oil.

Data for *cis*-2,3-bis(3-furyl)thiirane (**6o**):

^{1}H NMR: (400 MHz, DMSO-d_6)

δ 7.55 (bs, 2H), 7.51–7.50 (m, 2H), 6.19–6.18 (m, 2H), 4.21 (s, 2H). (4.28 (s, 0.04H) for *trans*-**6o**)

^{13}C NMR: (100 MHz, DMSO-d_6)

δ 143.2, 142.2, 121.3, 111.2, 34.6.

HRMS (EI): $[M]^+$ calcd for $C_{10}H_8O_2S$: 192.0245; found: 192.0248.

2.3. Mechanistic Study

2.3.1. Trapping *trans*-Thiocarbonyl Ylide with Dipolarophiles

With *N*-phenylmaleimide

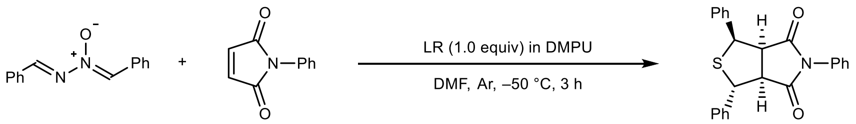

5a
(1.0 equiv) (10.0 equiv) (±)-**9a**

To a stirred mixture of benzaldazine *N*-oxide (**5a**, 224 mg, 1.00 mmol) and *N*-phenylmaleimide (1.73 g, 10.0 mmol) in DMF (5.0 mL) was added a solution of LR (404 mg, 1.00 mmol) in DMPU (5.0 mL) dropwise at –50 °C under Ar. (The change in internal temperature should not exceed 1 °C when the LR solution was added) After 3 h, the reaction mixture was transferred to a 100 mL separatory funnel and diluted with CH_2Cl_2 (4 mL). The mixture was washed with water (50 mL × 3) and brine (50 mL). The combined aqueous layers were extracted with CH_2Cl_2 (3 mL × 6). The combined organic layers were dried over anhydrous $MgSO_4$ (2 g), filtered, and concentrated *in vacuo*. The residue was purified immediately by flash column chromatography (SiO_2, ϕ = 3.5 cm, l = 10 cm, hexanes to EtOAc/hexanes = 1/2, R_f = 0.48 in EtOAc/hexanes = 1/2, $KMnO_4$). The chromatographed material was dissolved in EtOAc and filtered to remove the remaining *N*-phenylmaleimide. The insoluble material in EtOAc was recrystallized from CH_2Cl_2 and pentane to afford **9a** (190 mg, 50%) as white crystals.

Data for (1*R**,3*R**,3a*R**,6a*S**)-1,3,5-triphenyltetrahydro-4*H*-thieno[3,4-*c*]pyrrole-4,6(5*H*)-dione (**9a**): **SMS-15-053**

^{1}H NMR: (400 MHz, $CDCl_3$)

δ 7.52–7.49 (m, 2H), 7.46–7.42 (m, 4H), 7.40–7.29 (m, 7H), 7.19–7.16 (m, 2H), 5.31 (s, 1H), 5.00 (d, *J* = 8.9, 1H), 4.04 (dd, *J* = 7.6, 1.4, 1H), 3.94–3.90 (m, 1H).

^{13}C NMR: (100 MHz, $CDCl_3$)

δ 176.4, 173.4, 140.72, 135.0, 131.8, 129.3, 129.1, 128.8, 128.7 (3C), 127.9, 126.9, 126.3, 57.3, 55.3, 53.8, 53.0.

HRMS (ESI): $[M+H]^+$ calcd for $C_{24}H_{20}NO_2S$: 386.1216; found: 386.1207.

With maleic anhydride

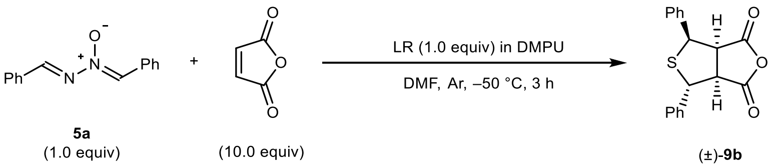

To a stirred mixture of benzaldazine *N*-oxide (**5a**, 224 mg, 1.00 mmol) and maleic anhydride (1.48 g, 10.0 mmol) in DMF (5.0 mL) was added a solution of LR (404 mg, 1.00 mmol) in DMPU (5.0 mL) dropwise at –50 °C under Ar. (The change in internal temperature should not exceed 1 °C when the LR solution was added) After 3 h, the reaction mixture was transferred to a 100 mL separatory funnel and diluted with CH_2Cl_2 (4 mL). The mixture was washed with water (50 mL × 3) and brine (50 mL). The combined aqueous layers were extracted with CH_2Cl_2 (3 mL × 6). The combined organic layers were dried over anhydrous $MgSO_4$ (2 g), filtered, and concentrated *in vacuo*. The residue was purified immediately by flash column chromatography twice (SiO_2, ϕ = 3.5 cm, l = 10 cm, EtOAc/hexanes = 1/2, R_f = 0.57, $KMnO_4$) and recrystallization (CH_2Cl_2 and pentane) to afford **9b** (78 mg, 25%) as white crystals.

Data for (3a*R**,4*R**,6*R**,6a*S**)-4,6-diphenyltetrahydro-1*H*,3*H*-thieno[3,4-*c*]furan-1,3-dione (**9b**): **SMS-15-054**

^{1}H NMR: (400 MHz, $CDCl_3$)
δ 7.46–7.40 (m, 4H), 7.39–7.31 (m, 6H), 5.20 (s, 1H), 4.91 (d, *J* = 8.9, 1H), 4.19 (dd, *J* = 7.9, 1.1, 1H), 3.93 (dd, *J* = 8.4, 8.4, 1H).

^{13}C NMR: (100 MHz, $CDCl_3$)
δ 171.9, 168.3, 139.3, 133.3, 129.3, 129.2, 128.9, 128.5, 128.2, 126.6, 57.4, 55.5, 54.0, 53.6.

HRMS (ESI): $[M+Na]^+$ calcd for $C_{18}H_{14}O_3SNa$: 333.0561; found: 333.0553.

2.3.2. Crossover Experiment with Benzaldazine and Tolualdazine *N*-Oxides (SMS-15-070)

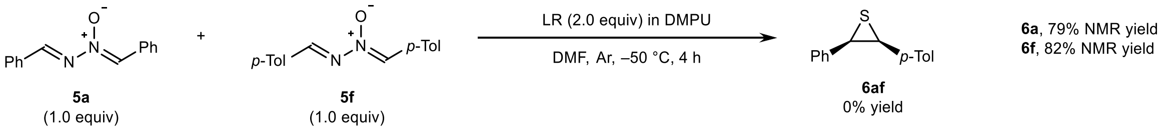

To a stirred mixture of benzaldazine *N*-oxide (**5a**, 112 mg, 0.500 mmol) and tolualdazine *N*-oxide (**5f**, 126 mg, 0.500 mmol) in DMF (5.0 mL) was added a solution of LR (404 mg, 1.00 mmol) in DMPU (5.0 mL) dropwise at –50 °C under Ar. After workup following general procedure, the residue was purified immediately by flash column chromatography (SiO_2, ϕ = 3.5 cm, l = 5.0 cm, hexanes, R_f = 0.21, $KMnO_4$) to give *cis*-thiiranes **6a** and **6f** (183 mg) as white solid. The scrambled product **6af** was not observed in ^{1}H NMR spectrum and EI-HRMS.

2.4. Computational Study

Approximate transition structures for the transformation of aldazine *N*-oxide **5a** to thiirane **6a** were obtained by the PM7 semi-empirical Hamiltonian using MOPAC 2016.[9] These saddle points were refined by DFT calculation at the M06-2X/6-311+G(d,p)/ PCM(DMF) level[10] of theory using the Gaussian 16 suite of programs.[11] These transition structures were verified by the presence of a single negative frequency as well as the Intrinsic Reaction coordinate (IRC) calculation at the same level of theory. All the reported energy values are obtained after thermal free energy correction. The 3-D illustrations were produced using CYLview 20.[12]

Reaction Progress Diagram

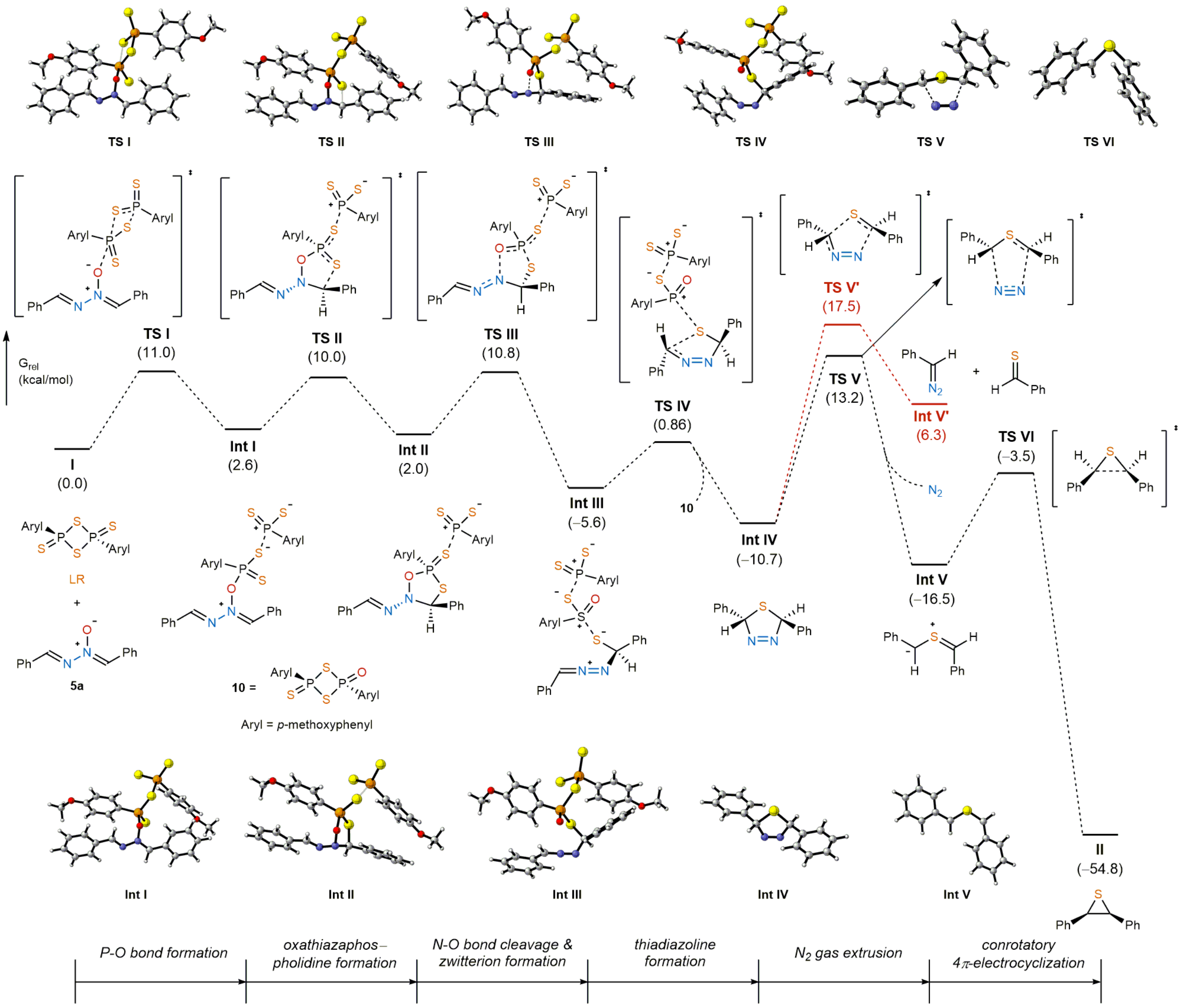

Supplementary Figure 1. DFT calculation of the reaction mechanism.

* The Cartesian coordinates are provided in a separate text file.

- computed data for **I** (**5a** + **LR**)
: total free energy = –3692.624393 Hartree
: relative free energy = 0.00 kcal/mol
: no imaginary frequency

- computed data for **TS I**
: total free energy = –3692.606887 Hartree
: relative free energy = 11.0 kcal/mol
: a single imaginary frequency at –166.2 cm^{-1}

- computed data for **Int I**
: total free energy = –3692.620196 Hartree
: relative free energy = 2.6 kcal/mol
: no imaginary frequency

- computed data for **TS II**
: total free energy = –3692.608463 Hartree
: relative free energy = 10.0 kcal/mol
: a single imaginary frequency at –235.5 cm^{-1}

- computed data for **Int II**
: total free energy = –3692.621169 Hartree
: relative free energy = 2.0 kcal/mol
: no imaginary frequency

- computed data for **TS III**
: total free energy = –3692.607183 Hartree
: relative free energy = 10.8 kcal/mol
: a single imaginary frequency at –386.5 cm^{-1}

- computed data for **Int III**
: total free energy = –3692.633254 Hartree
: relative free energy = –5.6 kcal/mol
: no imaginary frequency

- computed data for **TS IV**
: total free energy = –3692.623029 Hartree
: relative free energy = 0.86 kcal/mol
: a single imaginary frequency at –178.8 cm^{-1}

- computed data for **Int IV**

: total free energy with **10** = –3692.641371 Hartree (without **10**, –1048.074840 Hartree)

: relative free energy = –10.7 kcal/mol

: no imaginary frequency

- computed data for **TS V**

: total free energy = –1048.036869 Hartree

: relative free energy = 13.2 kcal/mol

: a single imaginary frequency at –438.7 cm^{-1}

- computed data for **TS V'**

: total free energy = –1048.029902 Hartree

: relative free energy = 17.5 kcal/mol

: a single imaginary frequency at –313.6 cm^{-1}

- computed data for **Int V**

: total free energy = –1048.084234 Hartree

: relative free energy = –16.5 kcal/mol

: no imaginary frequency

- computed data for **Int V' (diazo compound + thial)**

: total free energy = –1048.047885 Hartree

: relative free energy = 6.3 kcal/mol

: no imaginary frequency

- computed data for **TS VI**

: total free energy = –1048.063416 Hartree

: relative free energy = –3.5 kcal/mol

: a single imaginary frequency at –189.6 cm^{-1}

- computed data for **II** (**6a**)

: total free energy = –1048.145229 Hartree

: relative free energy = –54.8 kcal/mol

: no imaginary frequency

3. Supplementary Figures

3.1. X-Ray Crystallographic Data

Reflection data were collected using a Bruker APEX-II CCD-based diffractometer with graphite-monochromated MoKα radiation (λ = 0.7107 Å). The hemisphere of the reflection data was collected as ω scan frames at 0.5°/frame and an

exposure time of 5 s/frame. The cell parameters were determined and refined using the APEX2 program.[13] The data were corrected for Lorentz and polarization effects and an empirical absorption correction was applied using the SADABS program.[14] The compound structures were solved by direct methods and refined by full matrix least-squares using the SHELXTL program package[15] and Olex2[16] with anisotropic thermal parameters for all non-hydrogen atoms. The relevant data are summarized in Supplementary Table 1. CCDC 2119465 (**9b**), 2119466 (**9a**) and 2119467 (**6d**) and contains the supplementary crystallographic data for this study. These data can be obtained free of charge from The Cambridge Crystallographic Data Centre via www.ccdc.cam.ac.uk/data_request/cif.

Supplementary Table 1. Crystallographic data and parameters for compound **9b**, **9a**, and **6d.**

	**9b**	**9a**	**6d**
Identification code	Jwj03	jwj07-2	Jwj08-6
CCDC #	2119465	2119466	2119467
Empirical formula	$C_{18}H_{14}O_3S$	$C_{24}H_{19}NO_2S$	$C_{14}H_{10}Br_2S$
Formula weight	310.35	385.46	370.10
Temperature/K	100.0	100.0	100.0
Crystal system	monoclinic	monoclinic	triclinic
Space group	P21/c	$P2_1/n$	P-1
a/Å	13.1767(2)	14.914(5)	7.6556(5)
b/Å	6.40670(10)	6.253(2)	8.0061(5)
c/Å	18.5814(3)	20.847(7)	12.1350(8)
α/°	90	90	71.839(4)
β/°	110.6178(8)	103.40(2)	88.096(4)
γ/°	90	90	63.963(4)
Volume/Å^3	1468.16(4)	1891.1(11)	630.31(7)
Z	4	4	2
ρ_{calc}(g/cm^3)	1.404	1.354	1.950
μ/mm^{-1}	0.230	0.191	6.568
F(000)	648.0	808.0	360.0
Crystal size/mm^3	0.13 × 0.11 × 0.10	0.15 × 0.07 × 0.07	0.1 × 0.1 × 0.1
Radiation	MoKα (λ = 0.71073)	MoKα (λ = 0.71073)	MoKα (λ = 0.71073)
2Θ range for data collection/°	6.616 to 51.544	5.616 to 51.558	5.878 to 51.522
Index ranges	$-16 \leq h \leq 16, -7 \leq k \leq 7, -22 \leq l \leq 22$	$-18 \leq h \leq 18, -7 \leq k \leq 7, -25 \leq l \leq 25$	$-9 \leq h \leq 9, -9 \leq k \leq 9, -12 \leq l \leq 14$
Reflections collected	19259	24098	8178
Independent reflections	2803 [R_{int} = 0.0246, R_{sigma} = 0.0170]	3609 [R_{int} = 0.0546, R_{sigma} = 0.0459]	2391 [R_{int} = 0.0596, R_{sigma} = 0.0625]
Data/restraints/parameters	2803/0/199	3609/0/253	2391/108/164
Goodness-of-fit on F2	1.060	1.093	1.037
Final R indexes [I>=2σ (I)]	R_1 = 0.0314, wR_2 = 0.0809	R_1 = 0.0463, wR_2 = 0.1091	R_1 = 0.0661, wR_2 = 0.1786
Final R indexes [all data]	R_1 = 0.0353, wR_2 = 0.0839	R_1 = 0.0767, wR_2 = 0.1221	R_1 = 0.0732, wR_2 = 0.1858
Largest diff. peak/hole / e Å-3	0.34/-0.27	0.31/-0.36	3.29/-4.54

^{a} $R_1 = \sum||Fo| - |Fc||/\sum|Fo|$. ^{b} $wR_2 = \{[\sum w(Fo^2 - Fc^2)^2]/[\sum w(Fo^2)^2]\}^{1/2}$.

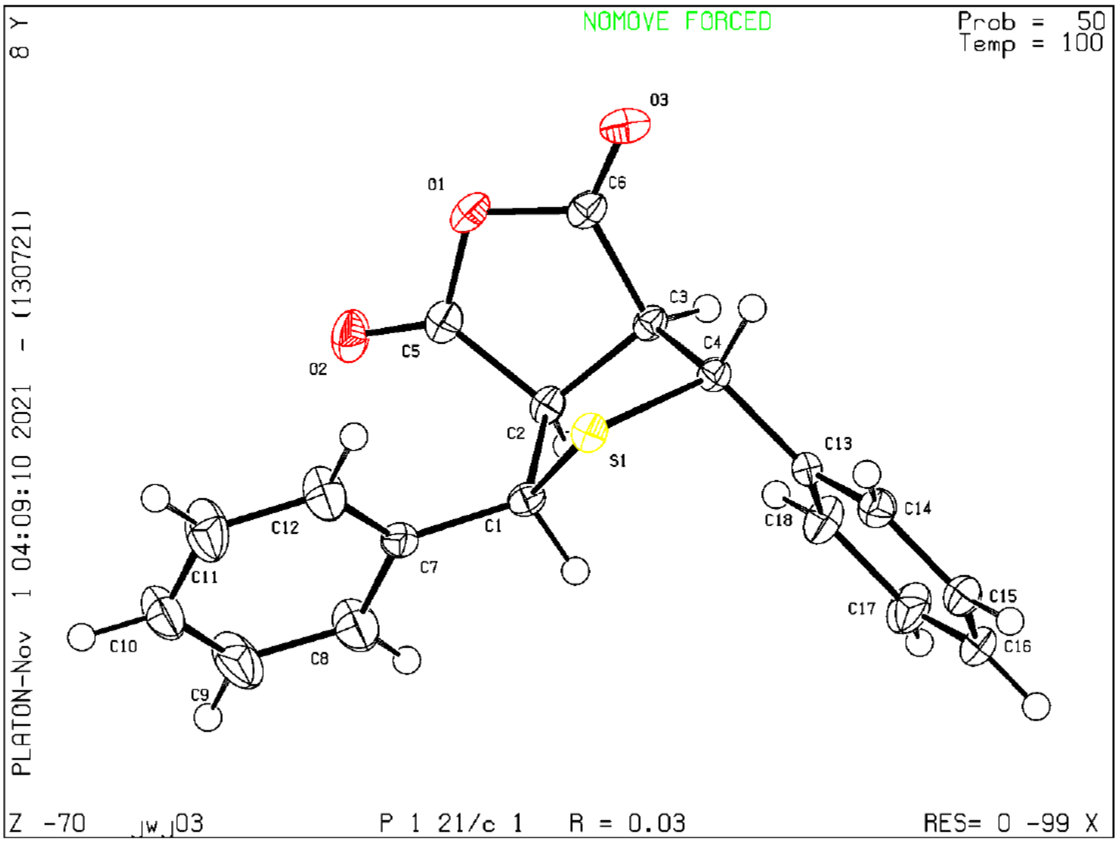

Supplementary Figure 2. ORTEP drawing of **9b**, CCDC 2119465.

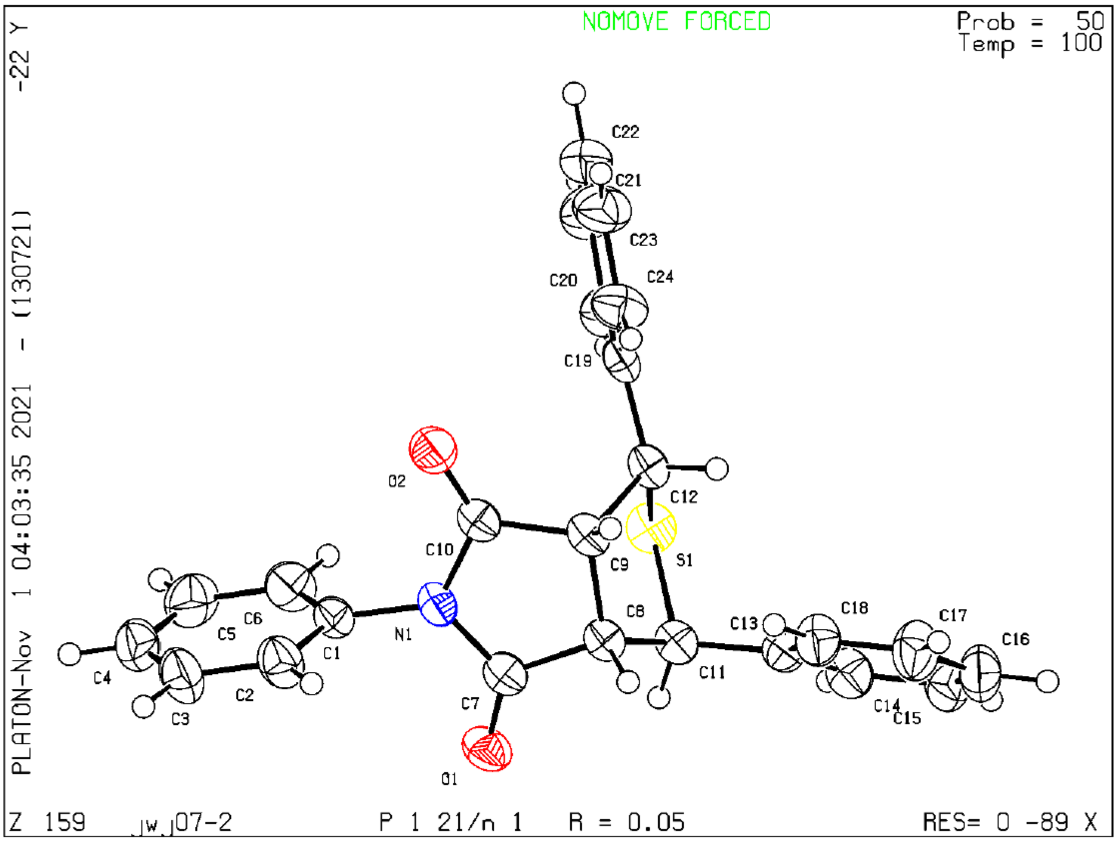

Supplementary Figure 3. ORTEP drawing of **9a**, CCDC 2119466.

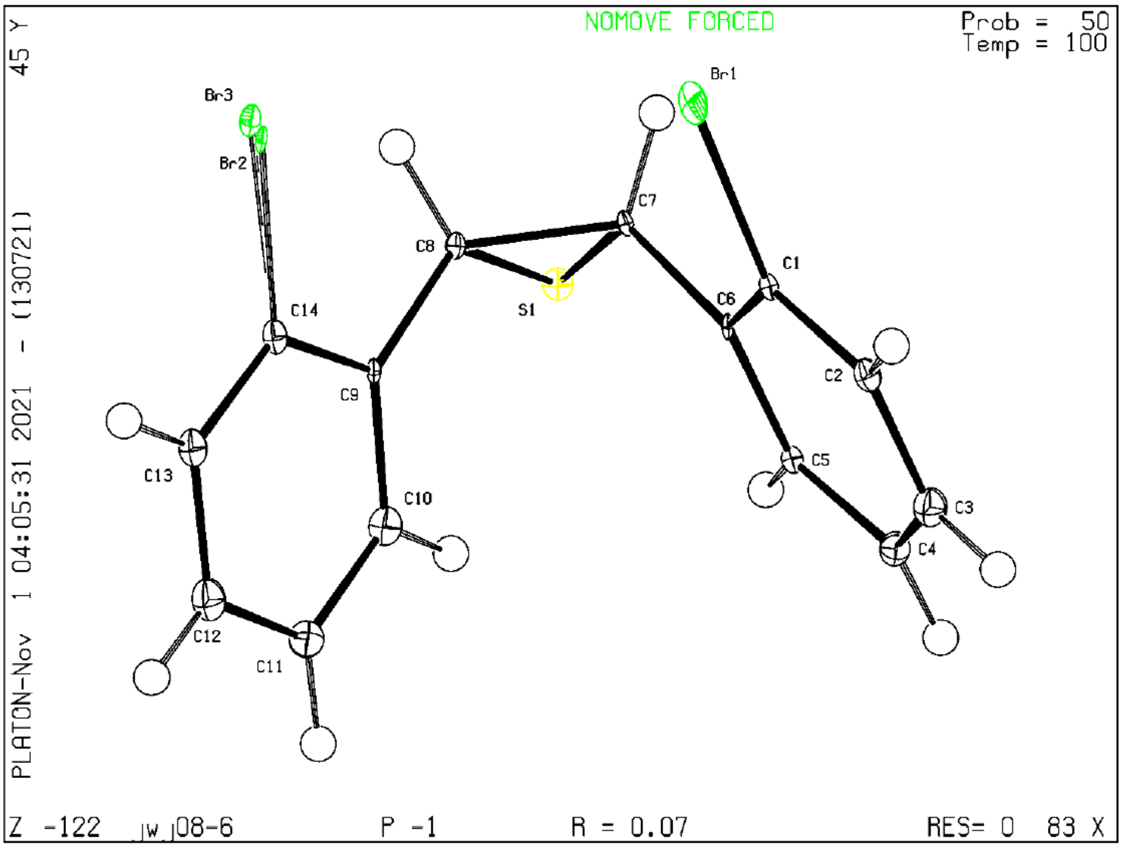

Supplementary Figure 4. ORTEP drawing of **6d**, CCDC 2119467.

CheckCif alerts for compound **6d** (CCDC 2119467).

A-level alerts

PLAT972_ALERT_2_A Check Calcd Resid. Dens. 0.62Ang From Br3 -4.53 eA-3

This is due to the subtle disorder of Br atoms.

PLAT972_ALERT_2_A Check Calcd Resid. Dens. 0.66Ang From Br2 -4.12 eA-3

This is due to the subtle disorder of Br atoms.

B-level alerts

DIFMN02_ALERT_2_B The minimum difference density is < -0.1*ZMAX*1.00
_refine_diff_density_min given = -4.540
Test value = -3.500

This is due to the subtle disorder of Br atoms.

PLAT098_ALERT_2_B Large Reported Min. (Negative) Residual Density -4.54 eA-3

This is due to the subtle disorder of Br atoms.

PLAT971_ALERT_2_B Check Calcd Resid. Dens. 0.48Ang From Br2 3.02 eA-3

This is due to the subtle disorder of Br atoms.

PLAT971_ALERT_2_B Check Calcd Resid. Dens. 0.43Ang From Br2 2.84 eA-3

This is due to the subtle disorder of Br atoms.

PLAT972_ALERT_2_B Check Calcd Resid. Dens. 0.59Ang From Br1 -2.66 eA-3

This is due to the subtle disorder of Br atoms.

PLAT972_ALERT_2_B Check Calcd Resid. Dens. 0.61Ang From Br1 -2.59 eA-3

This is due to the subtle disorder of Br atoms.

3.2. NMR Spectra

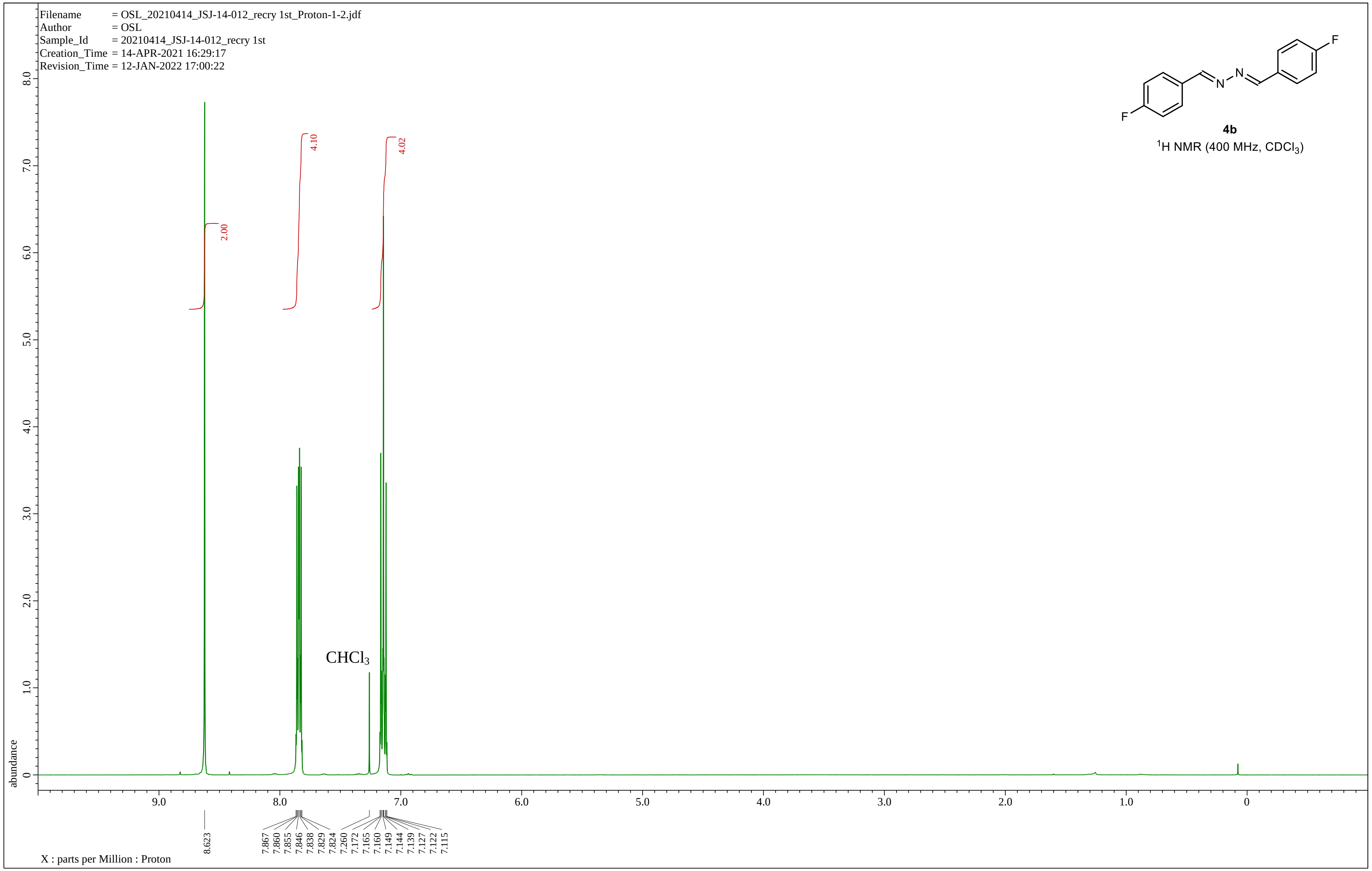

Supplementary Figure 5. ^{1}H NMR spectrum of compound **4b**, recorded at 400 MHz and 298 K in $CDCl_3$.

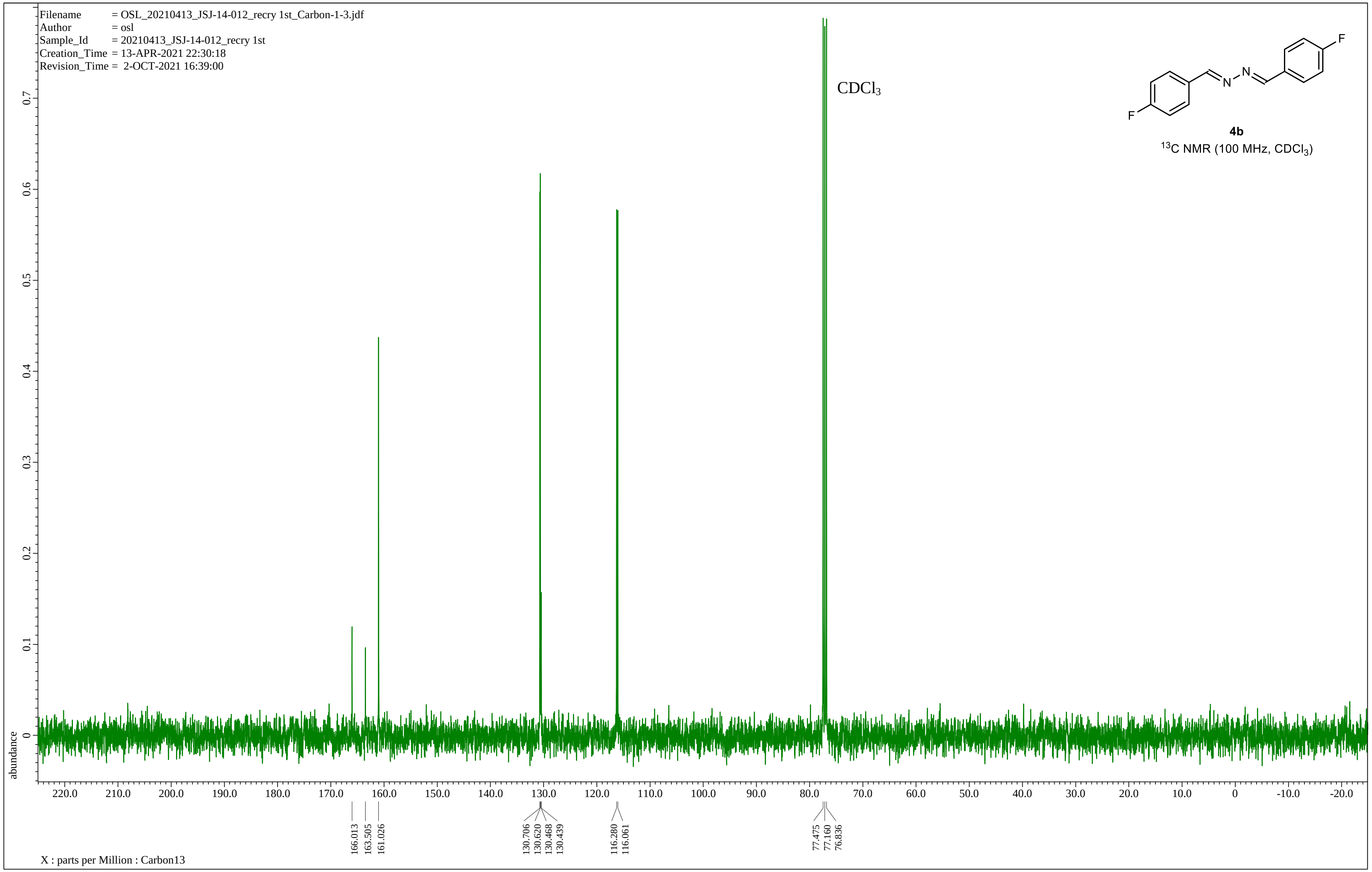

Supplementary Figure 6. ^{13}C NMR spectrum of compound **4b**, recorded at 100 MHz and 298 K in $CDCl_3$.

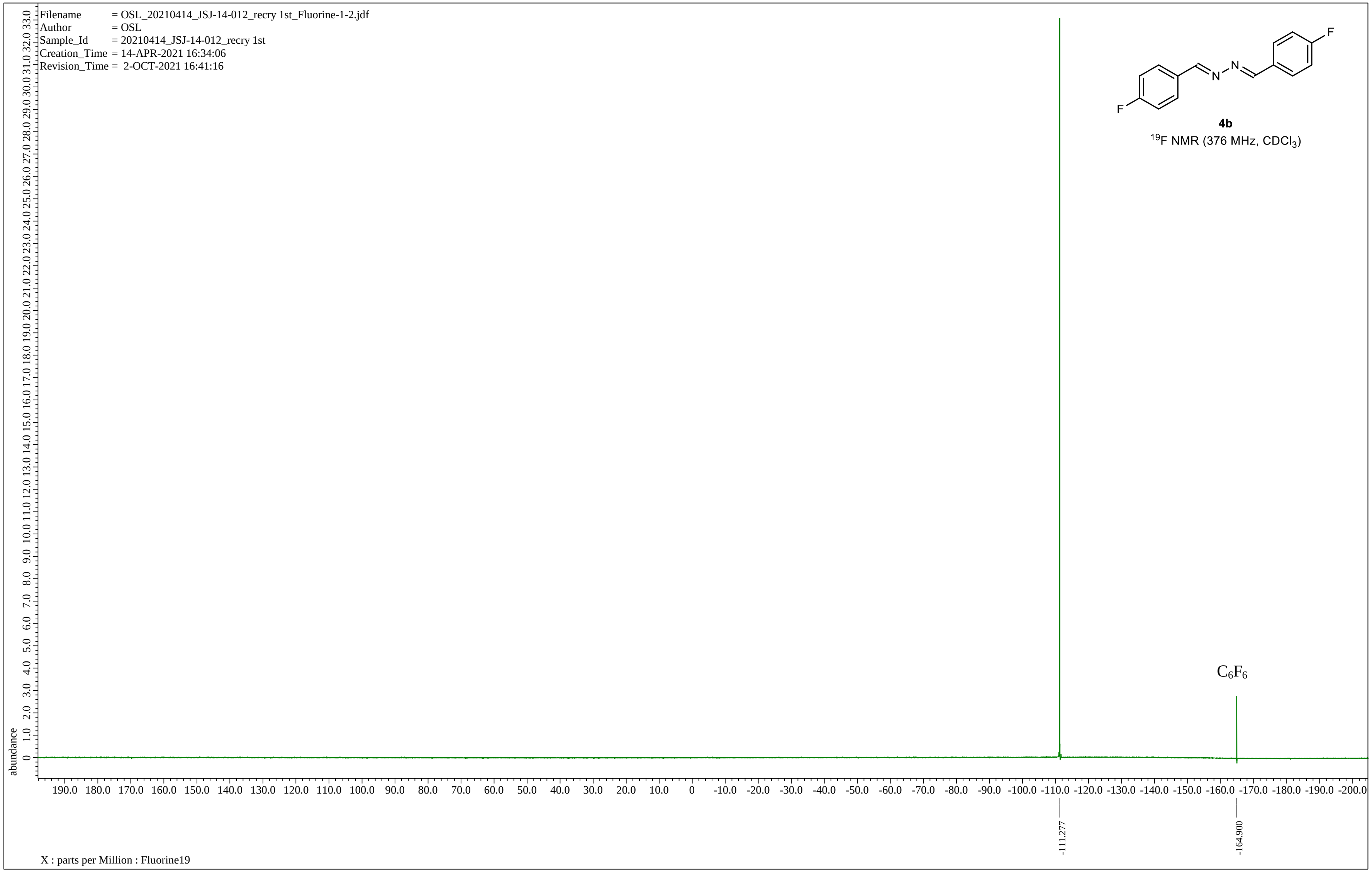

Supplementary Figure 7. ^{19}F NMR spectrum of compound **4b**, recorded at 376 MHz and 298 K in $CDCl_3$.

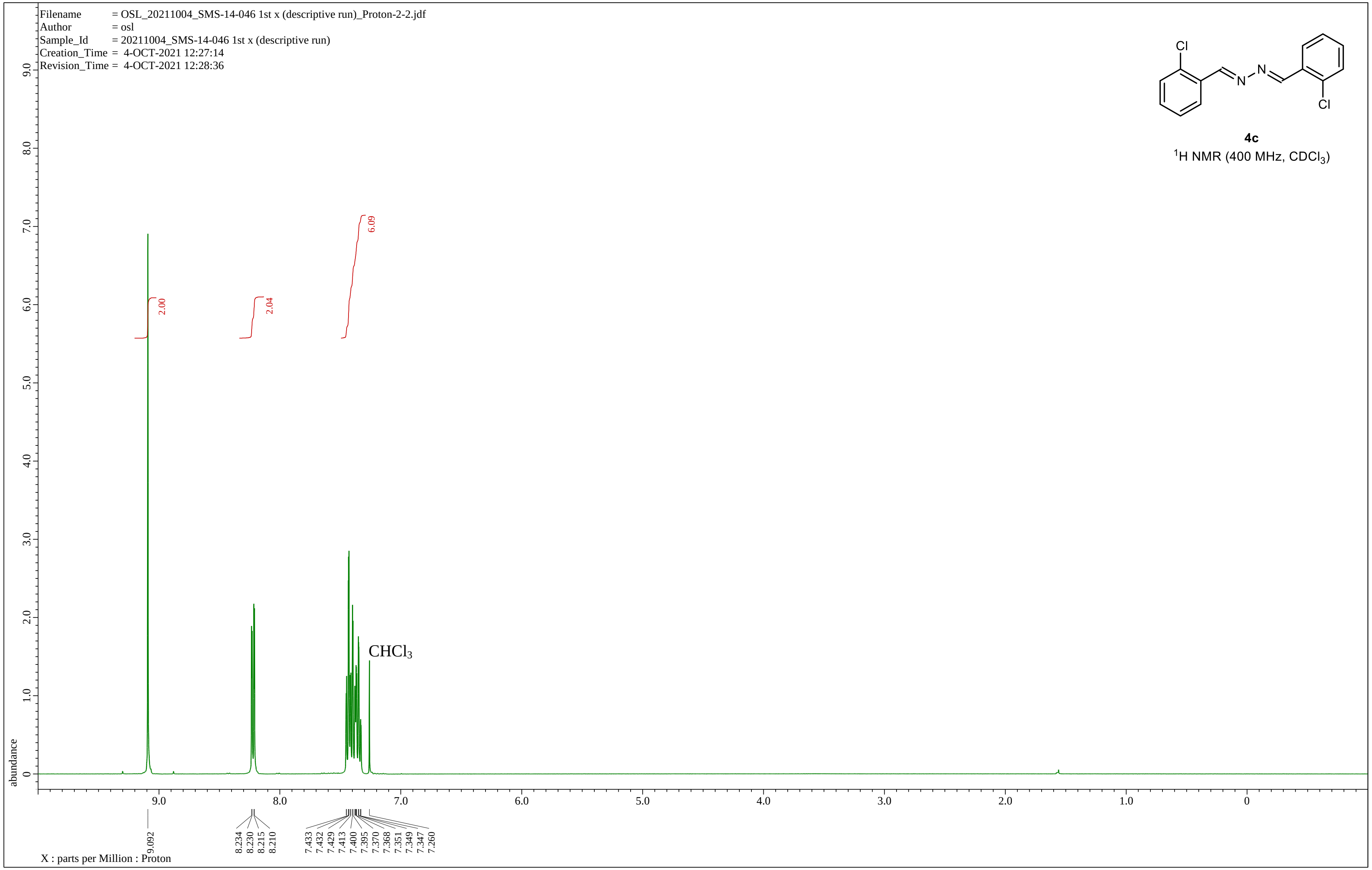

Supplementary Figure 8. ^{1}H NMR spectrum of compound **4c**, recorded at 400 MHz and 298 K in $CDCl_3$.

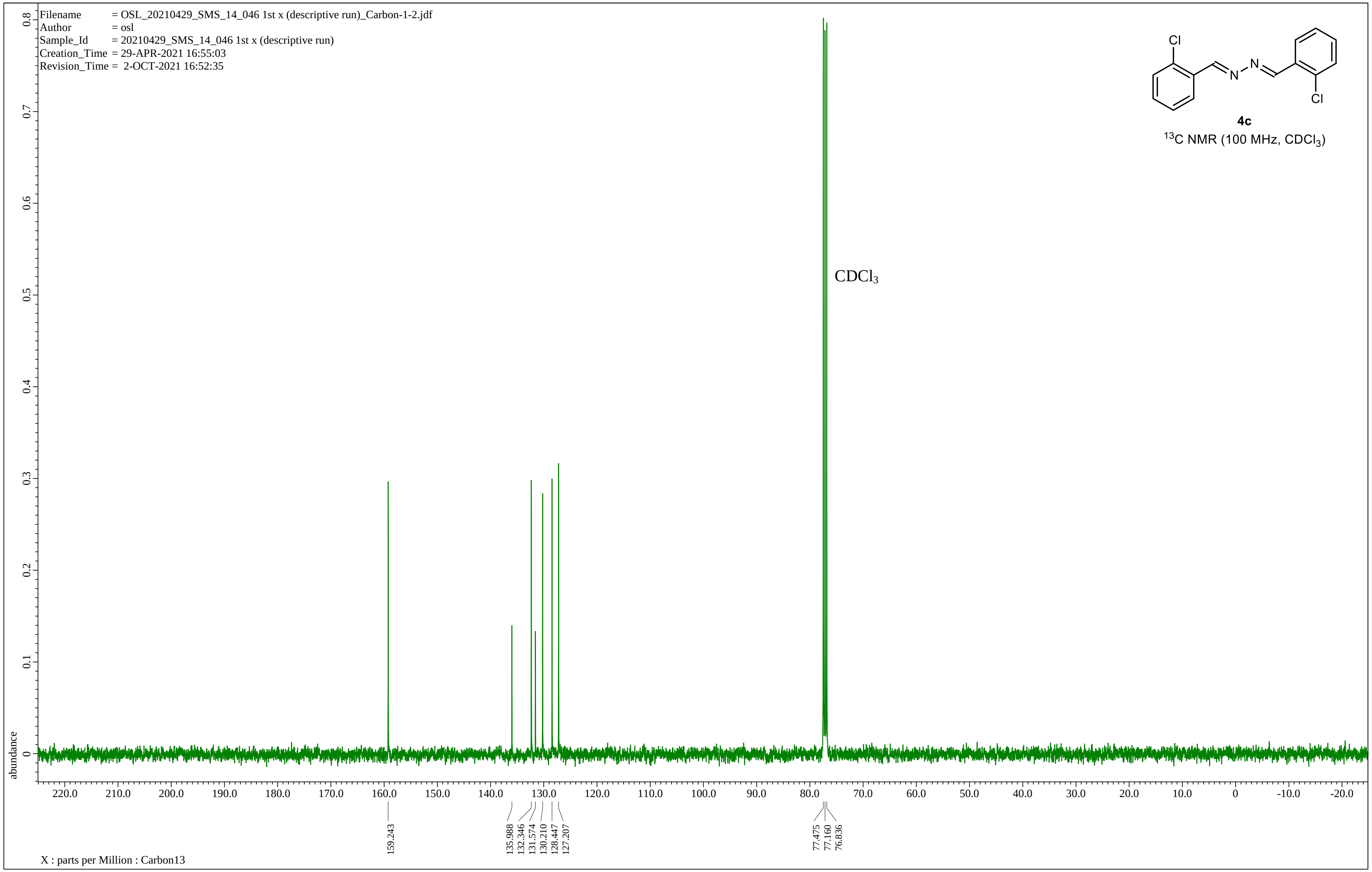

Supplementary Figure 9. ^{13}C NMR spectrum of compound **4c**, recorded at 100 MHz and 298 K in $CDCl_3$.

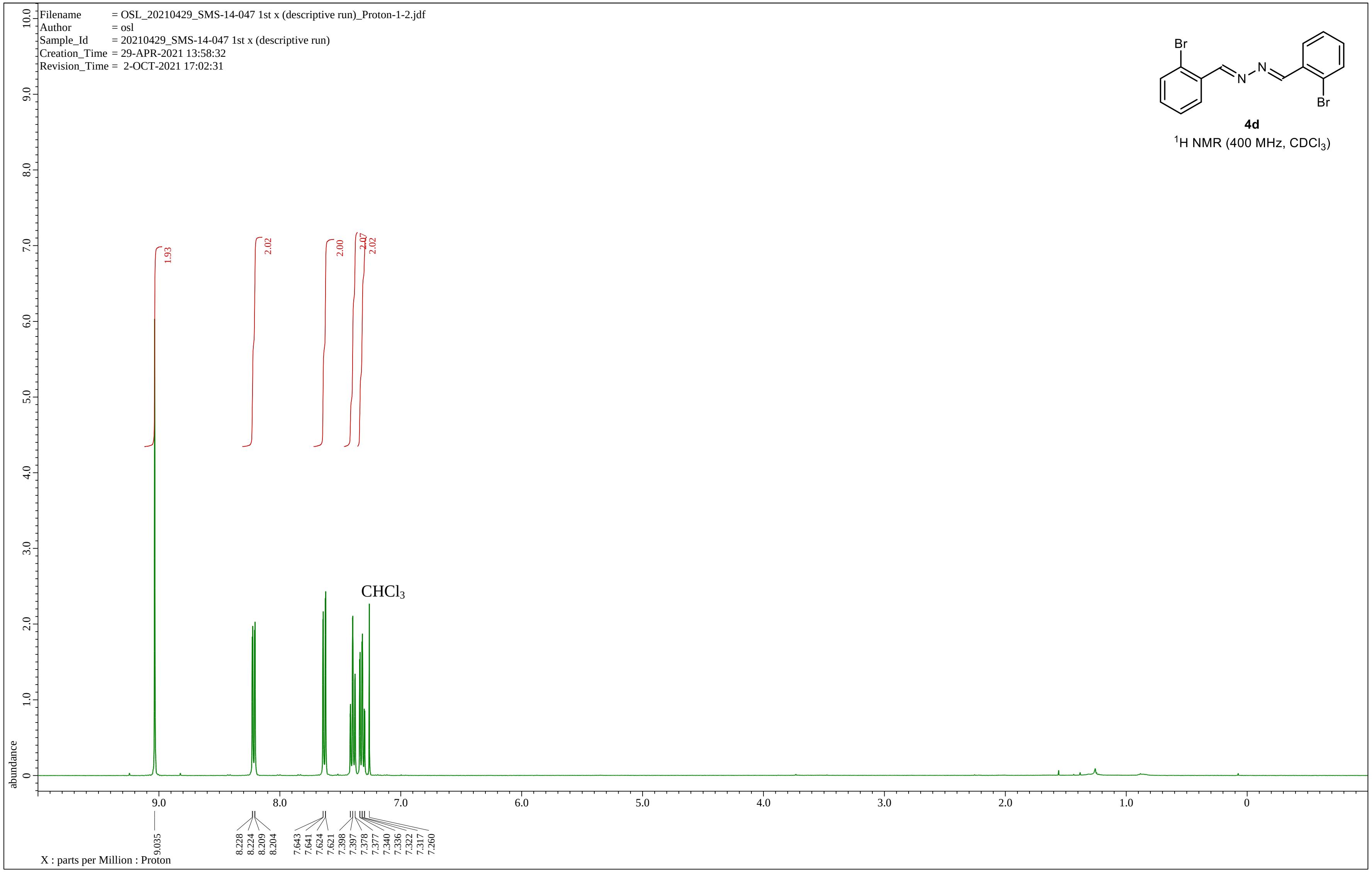

Supplementary Figure 10. ^{1}H NMR spectrum of compound **4d**, recorded at 400 MHz and 298 K in $CDCl_3$.

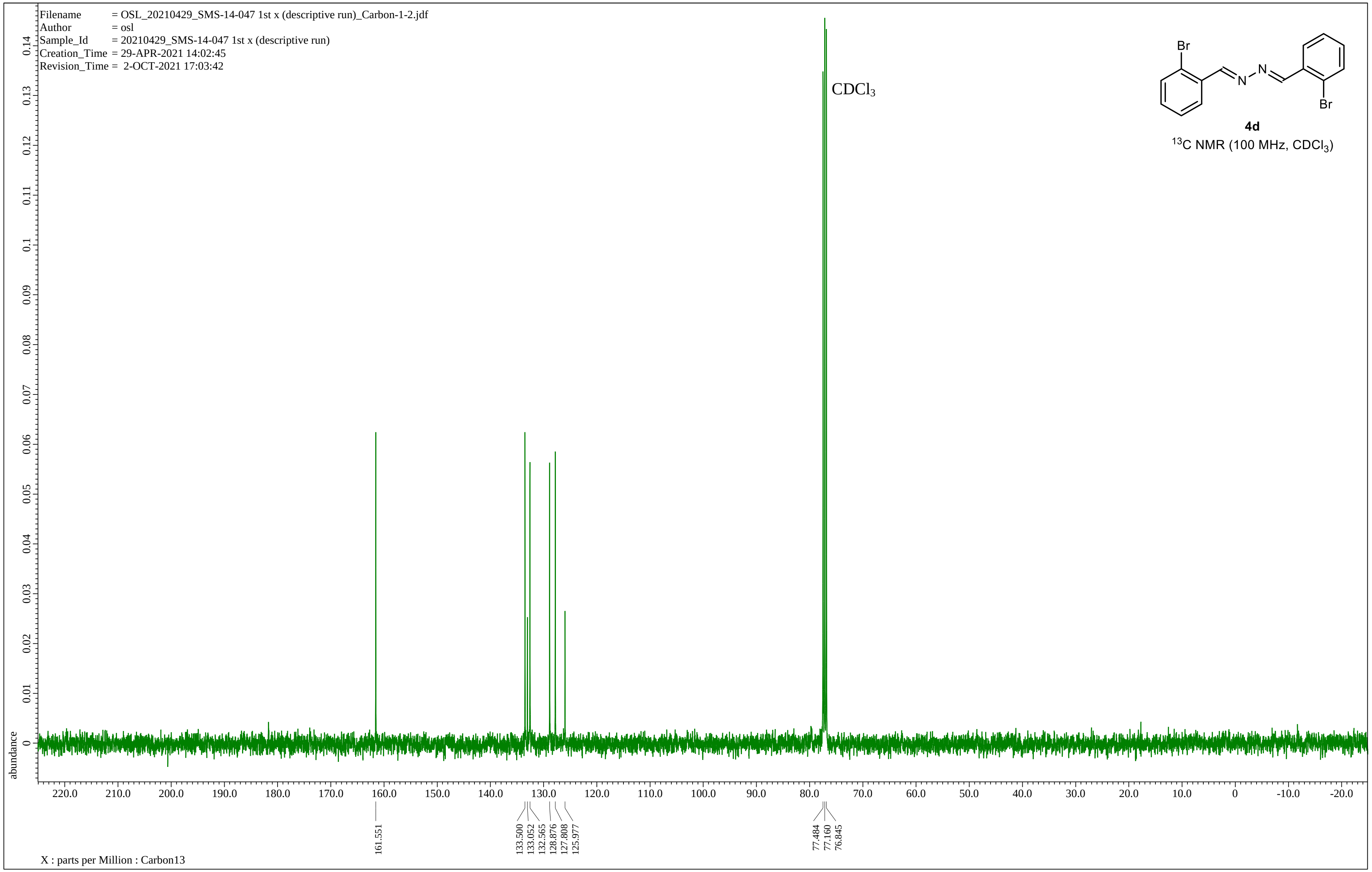

Supplementary Figure 11. ^{13}C NMR spectrum of compound **4d**, recorded at 100 MHz and 298 K in $CDCl_3$.

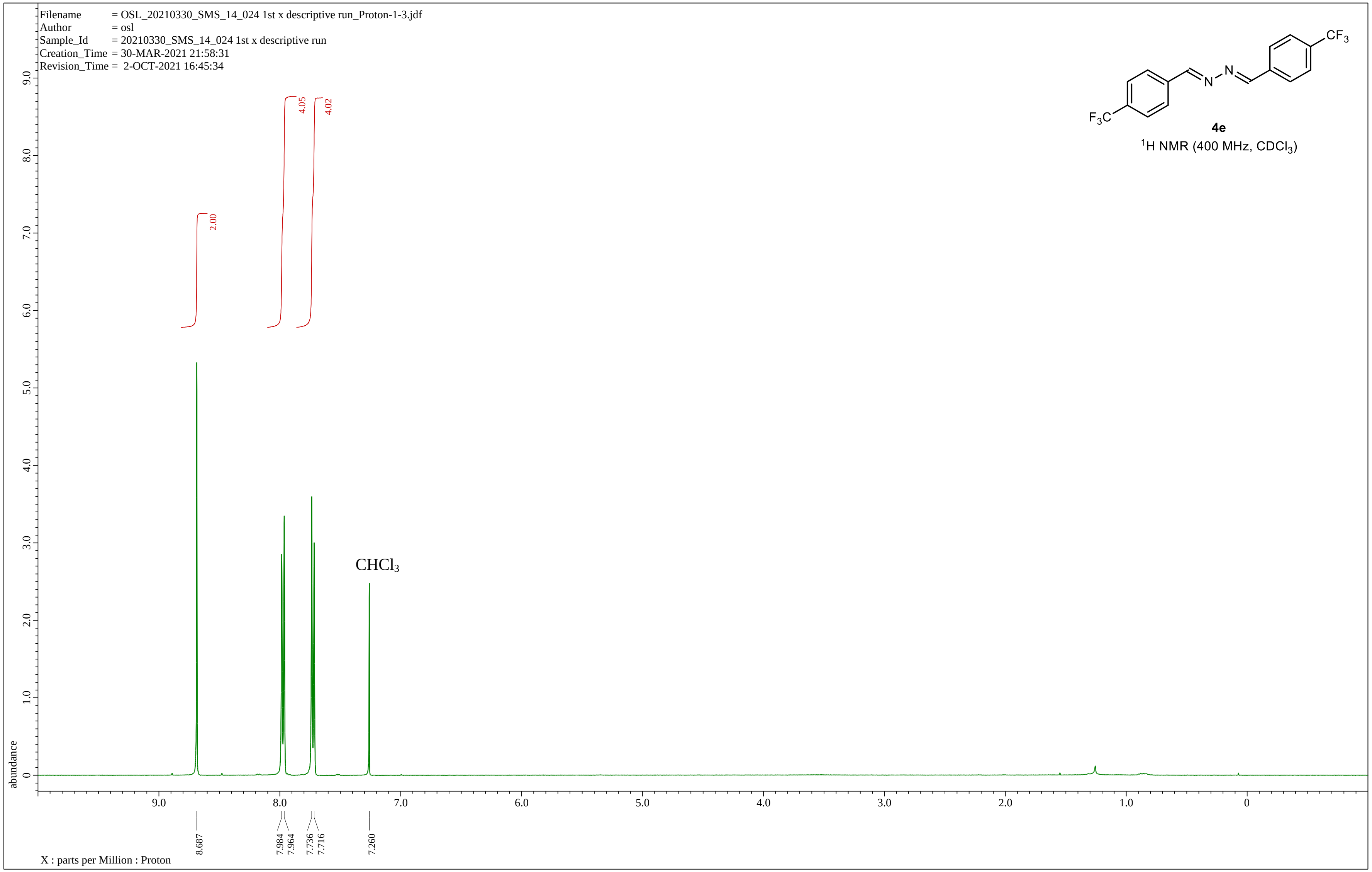

Supplementary Figure 12. ^{1}H NMR spectrum of compound **4e**, recorded at 400 MHz and 298 K in $CDCl_3$.

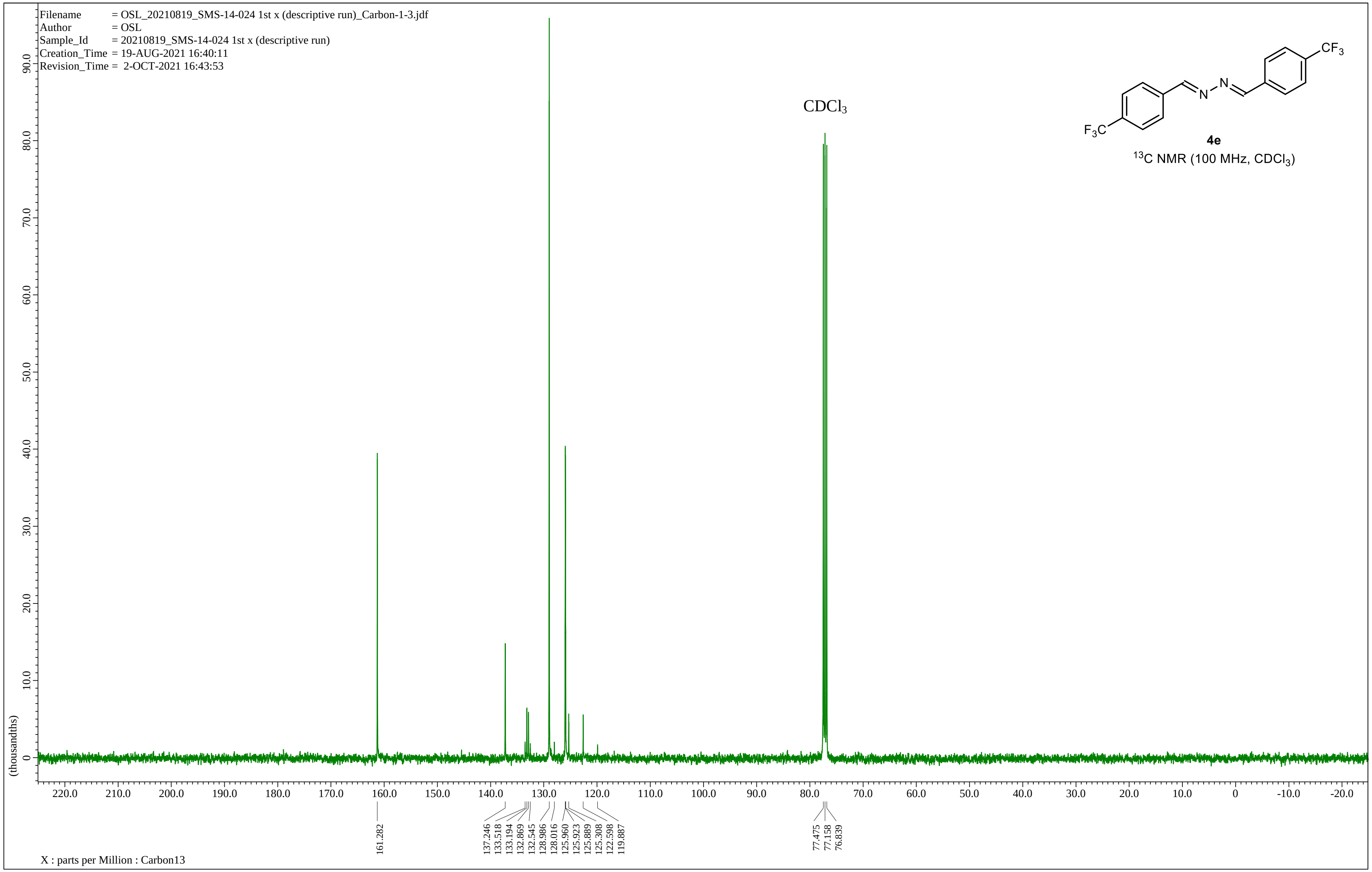

Supplementary Figure 13. ^{13}C NMR spectrum of compound **4e**, recorded at 100 MHz and 298 K in $CDCl_3$.

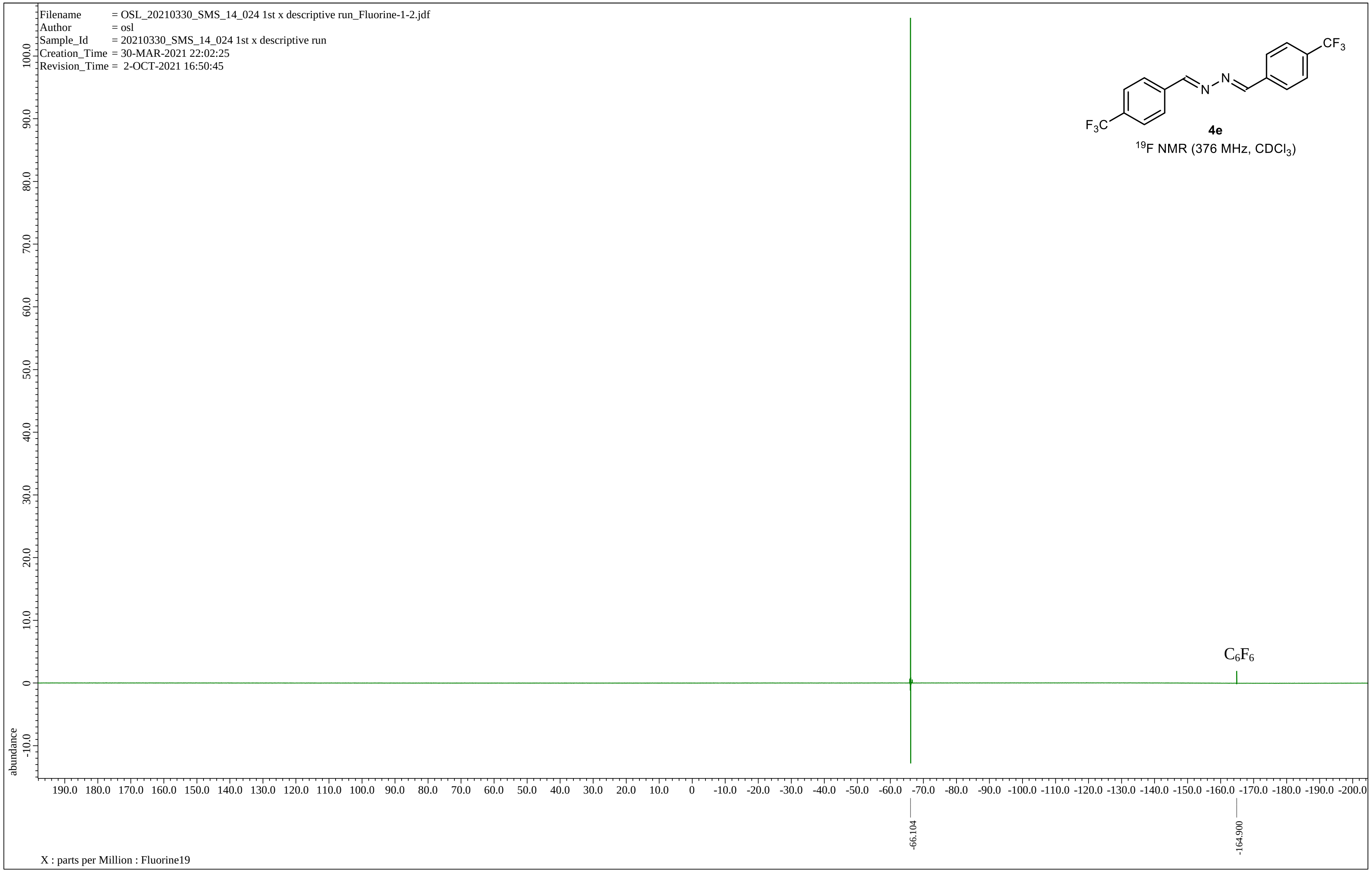

Supplementary Figure 14. ^{19}F NMR spectrum of compound **4e**, recorded at 376 MHz and 298 K in $CDCl_3$.

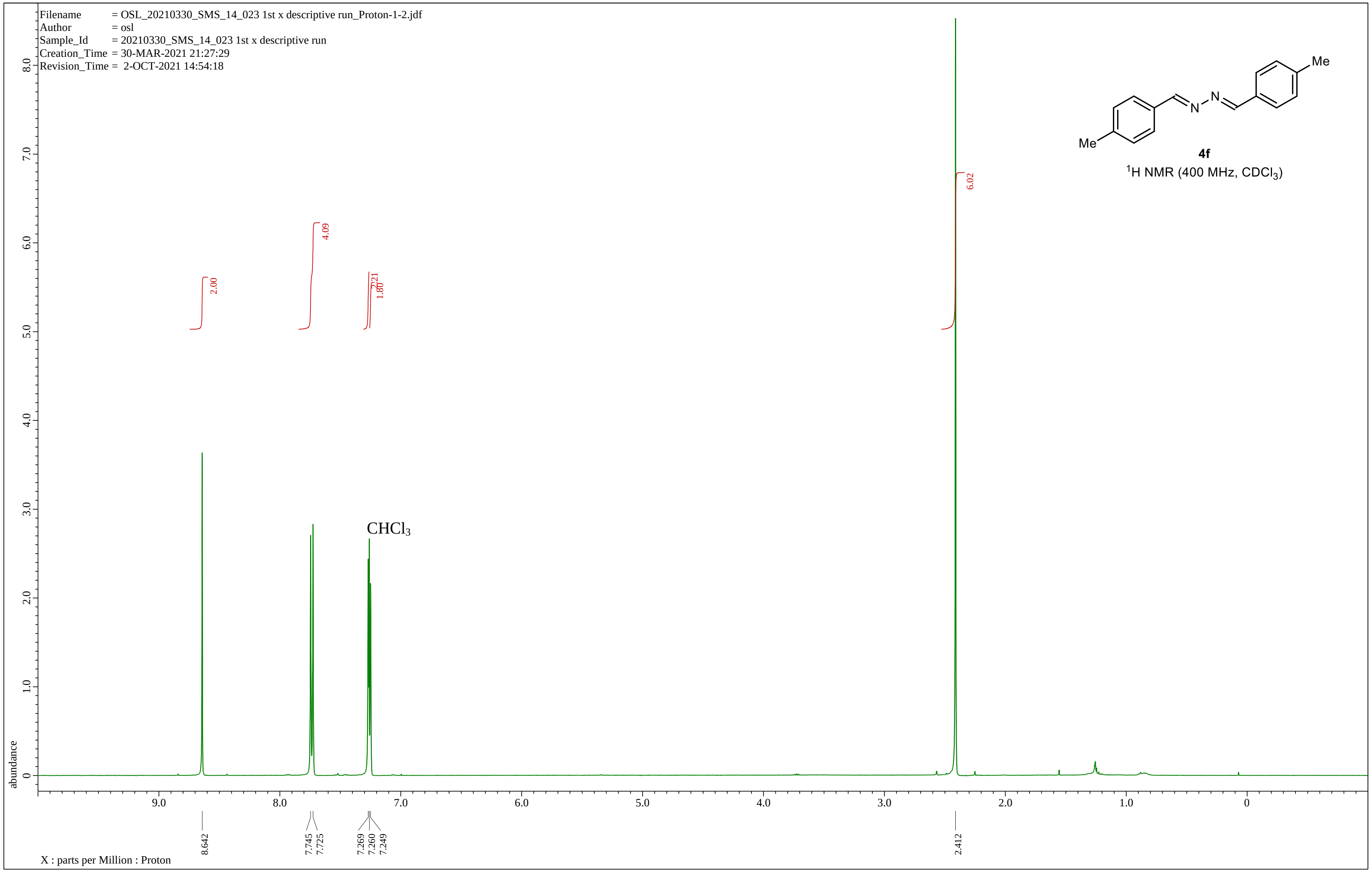

Supplementary Figure 15. ^{1}H NMR spectrum of compound **4f**, recorded at 400 MHz and 298 K in $CDCl_3$.

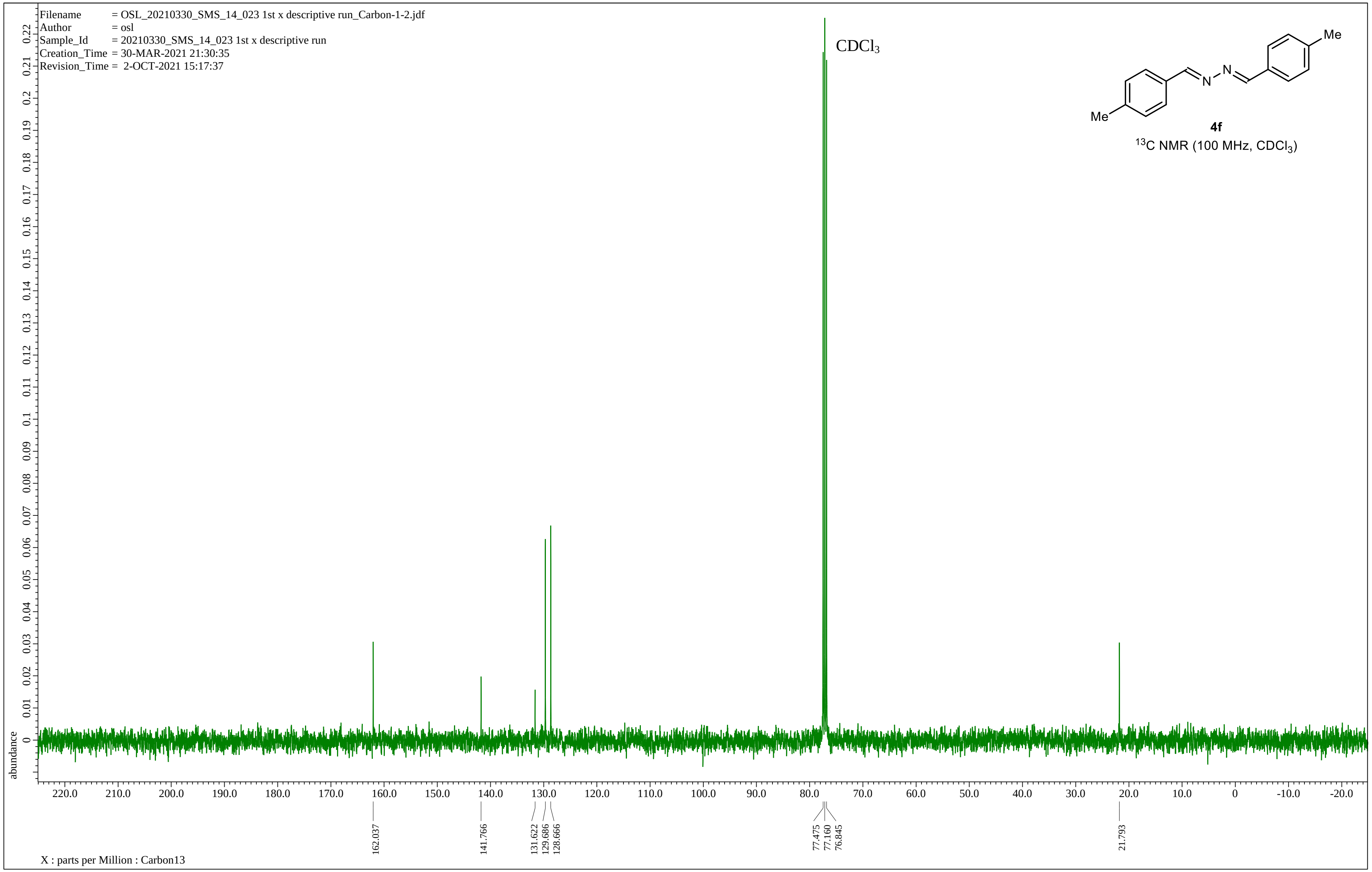

Supplementary Figure 16. ^{13}C NMR spectrum of compound **4f**, recorded at 100 MHz and 298 K in $CDCl_3$.

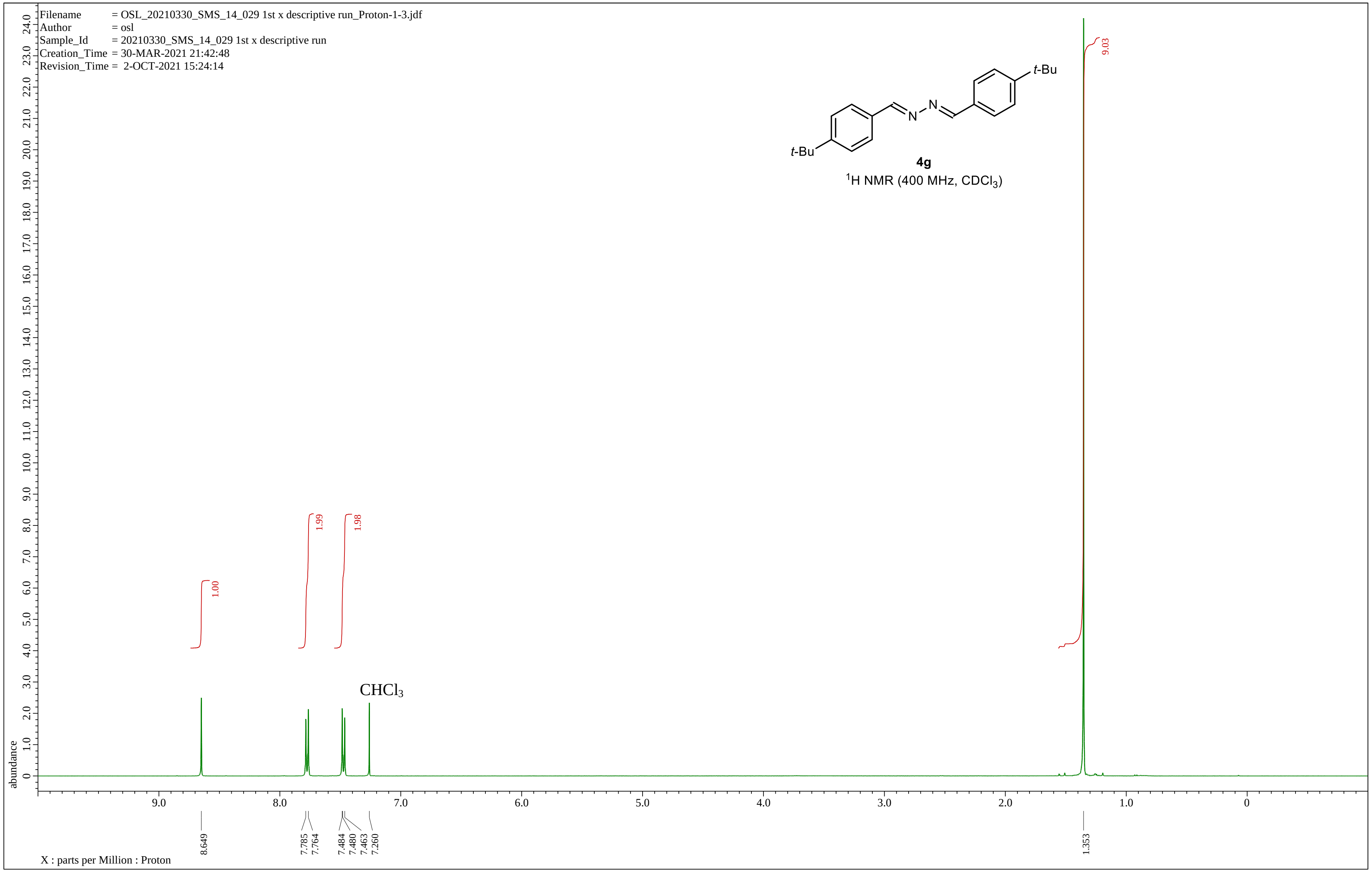

Supplementary Figure 17. ^{1}H NMR spectrum of compound **4g**, recorded at 400 MHz and 298 K in $CDCl_3$.

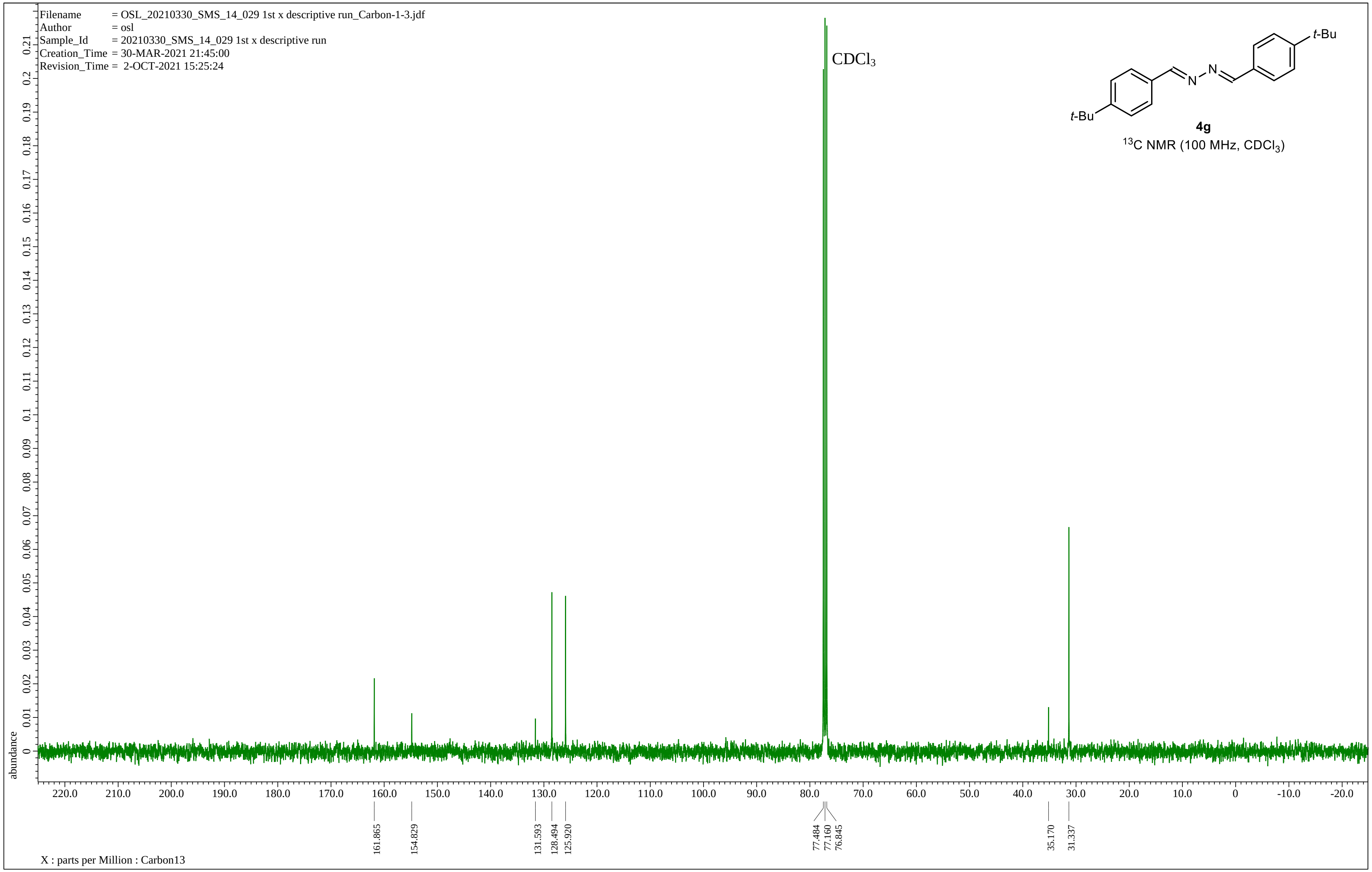

Supplementary Figure 18. ^{13}C NMR spectrum of compound **4g**, recorded at 100 MHz and 298 K in $CDCl_3$.

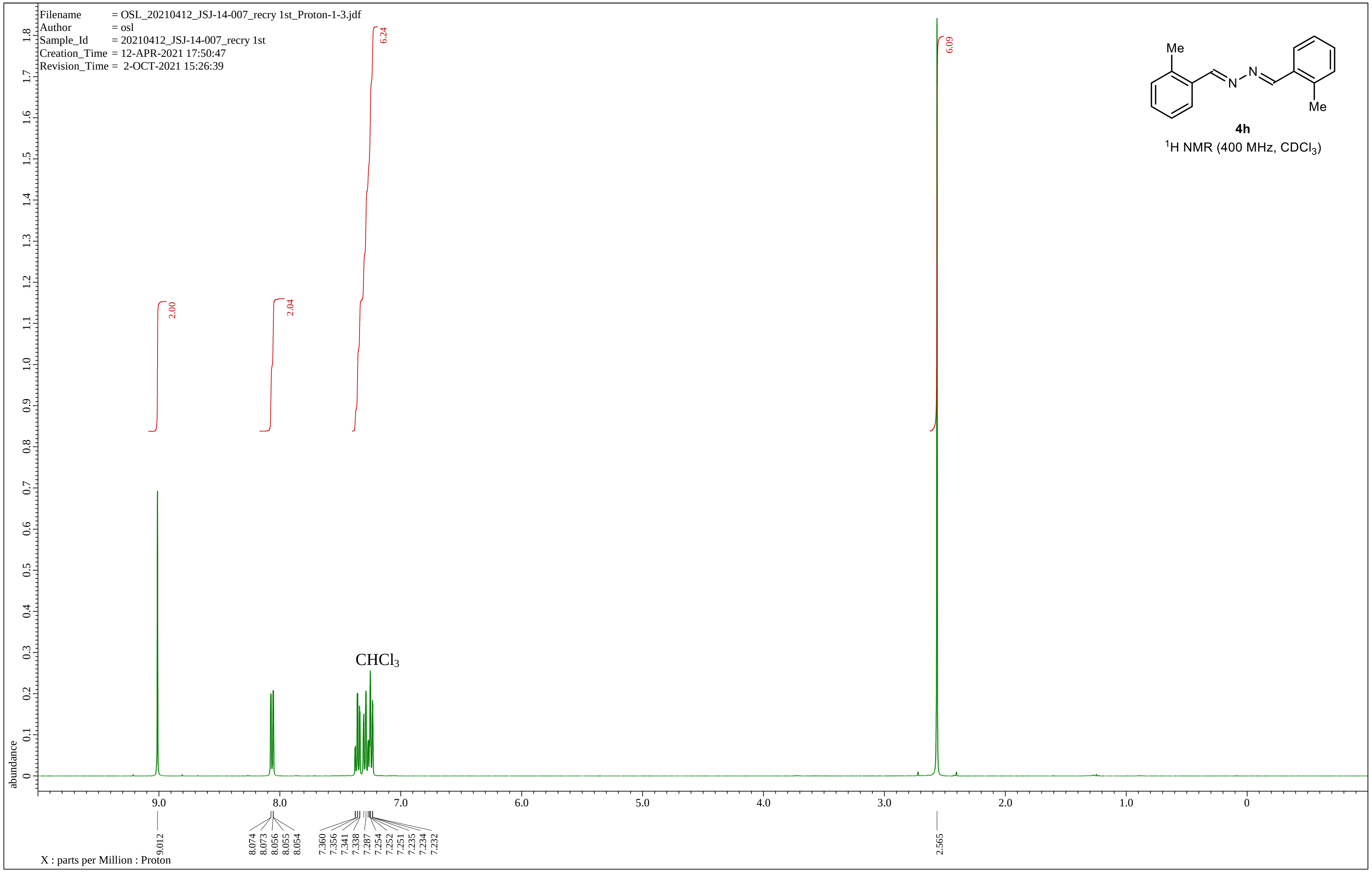

Supplementary Figure 19. ^{1}H NMR spectrum of compound **4h**, recorded at 400 MHz and 298 K in $CDCl_3$.

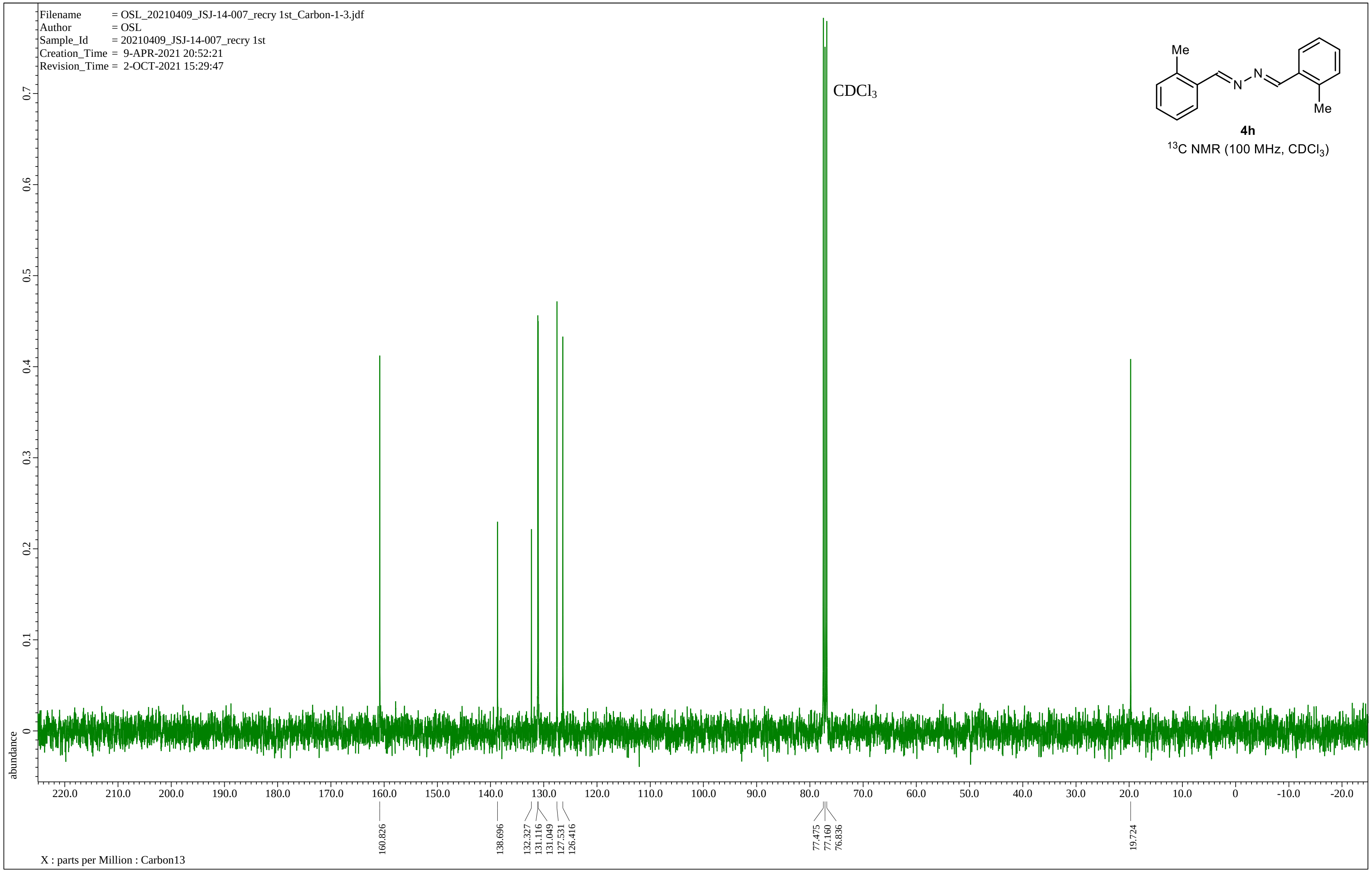

Supplementary Figure 20. ^{13}C NMR spectrum of compound **4h**, recorded at 100 MHz and 298 K in $CDCl_3$.

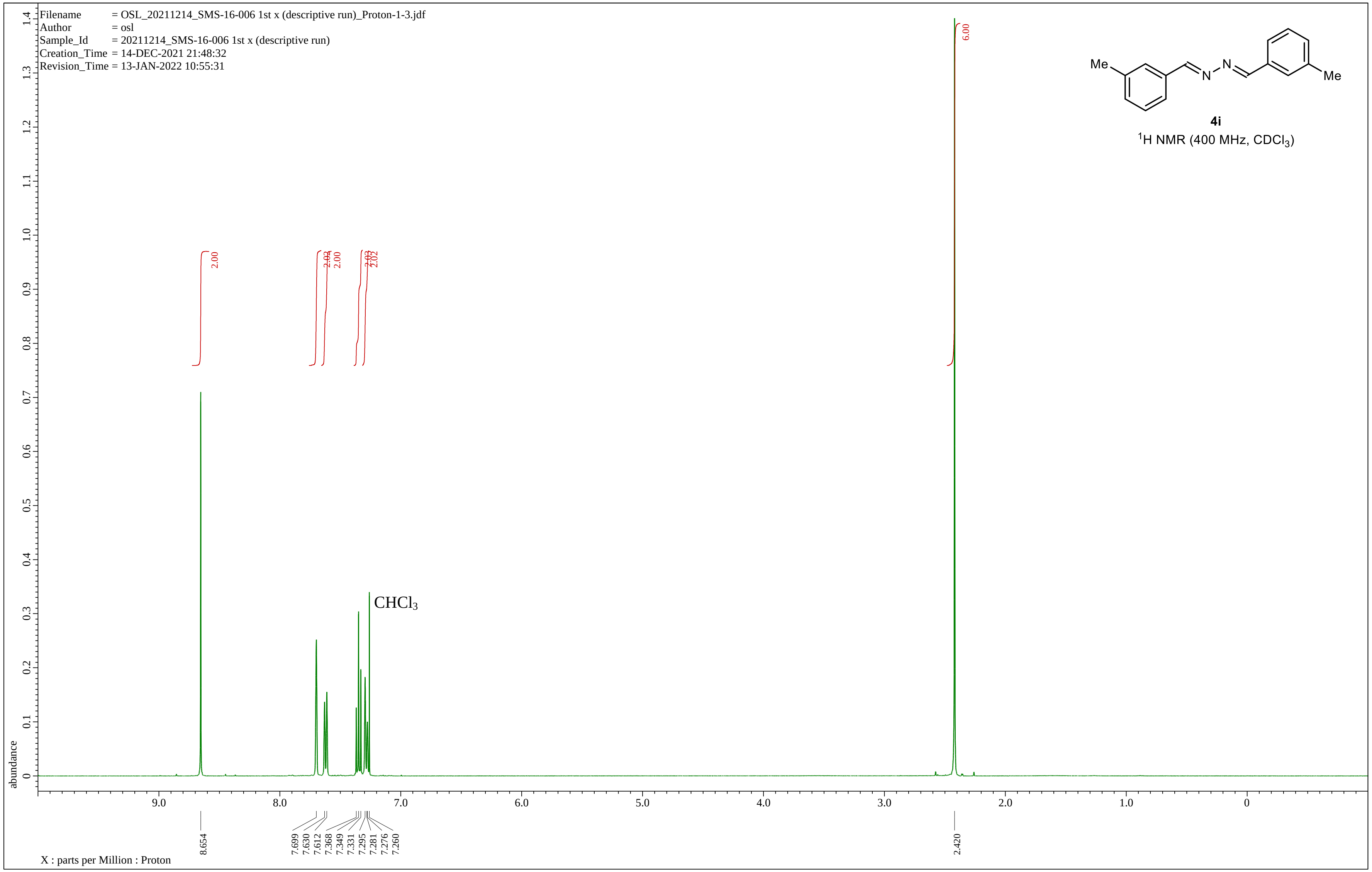

Supplementary Figure 21. ^{1}H NMR spectrum of compound **4i**, recorded at 400 MHz and 298 K in $CDCl_3$.

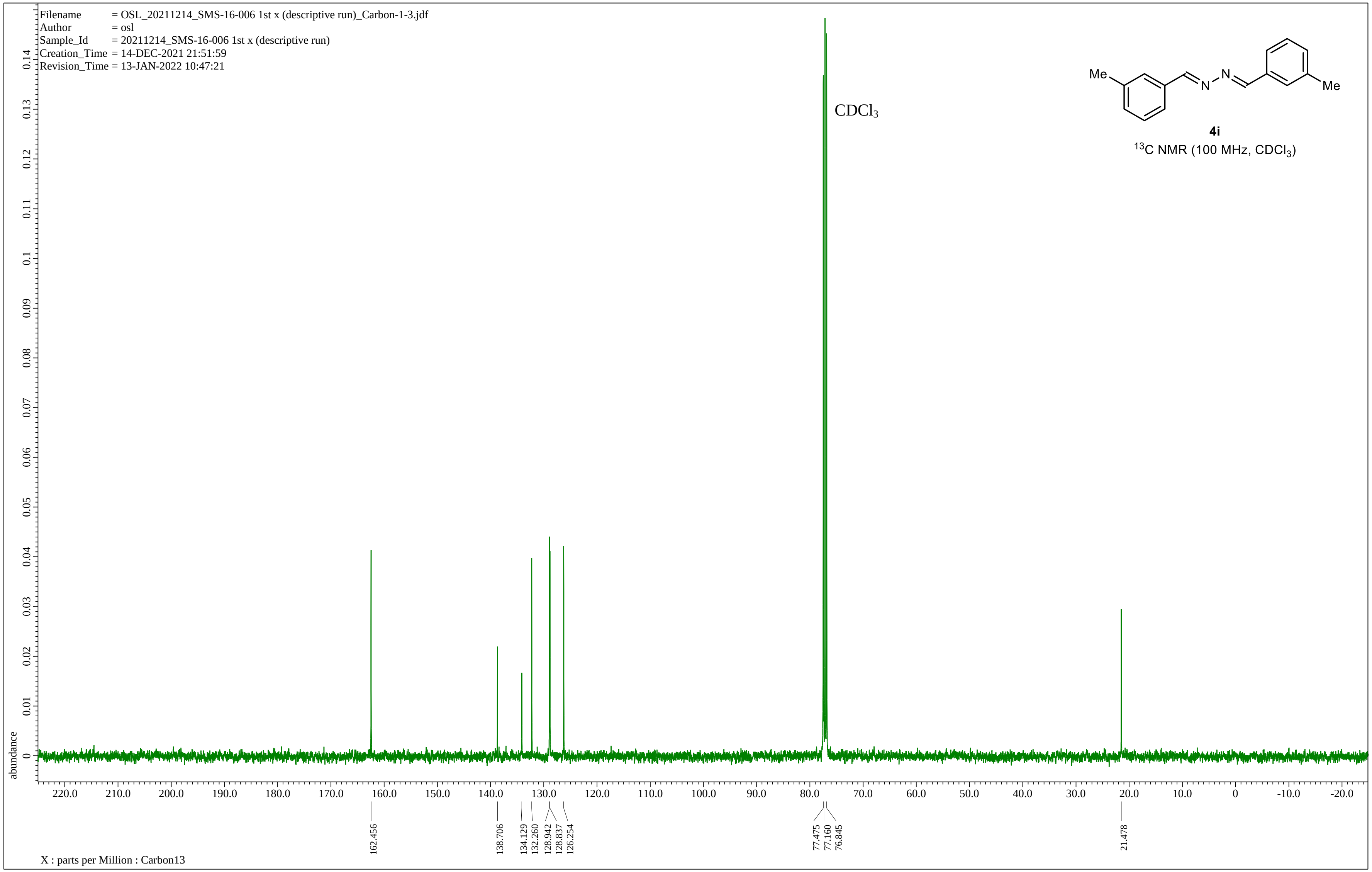

Supplementary Figure 22. ^{13}C NMR spectrum of compound **4i**, recorded at 100 MHz and 298 K in $CDCl_3$.

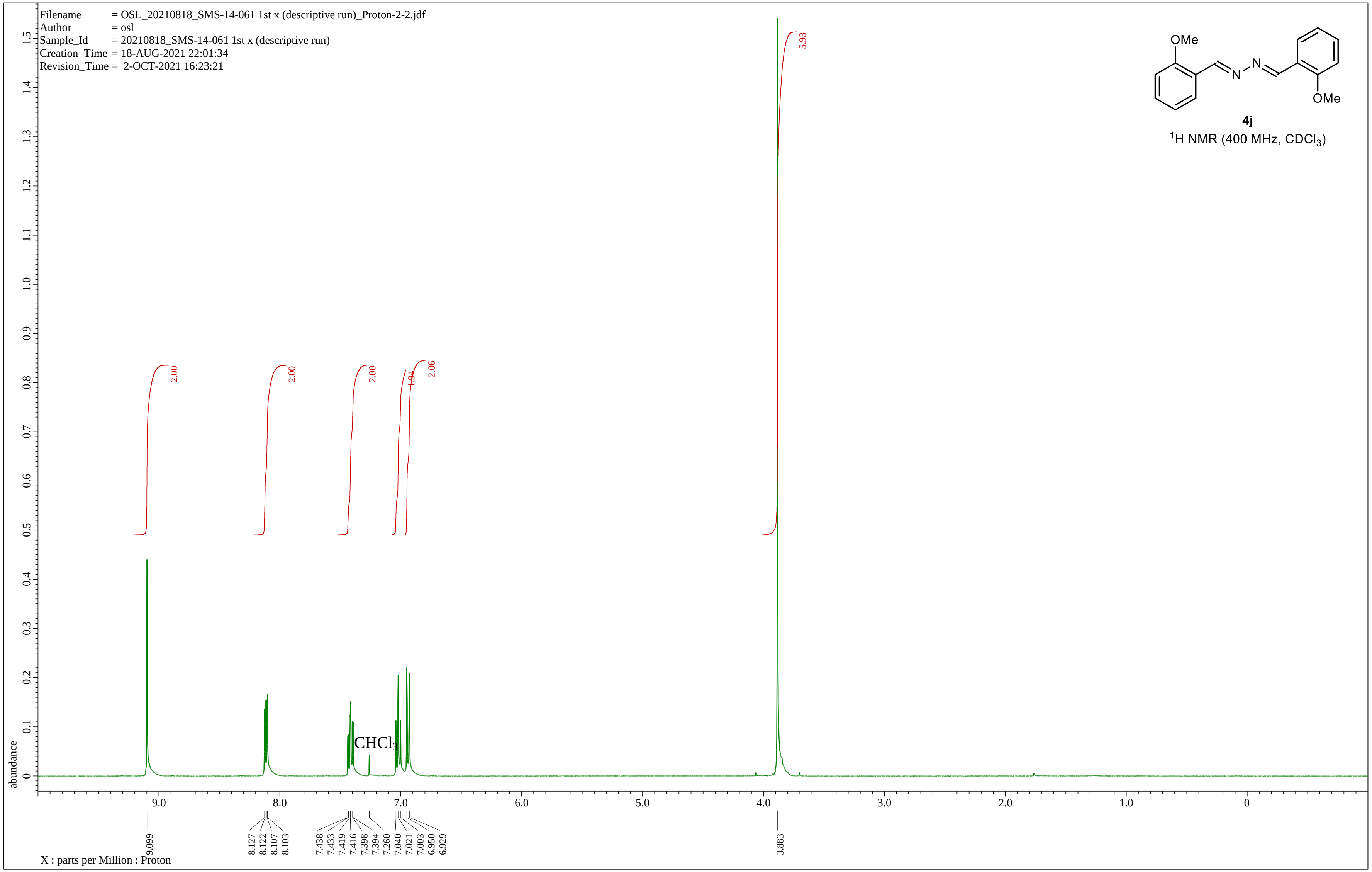

Supplementary Figure 23. ^{1}H NMR spectrum of compound **4j**, recorded at 400 MHz and 298 K in $CDCl_3$.

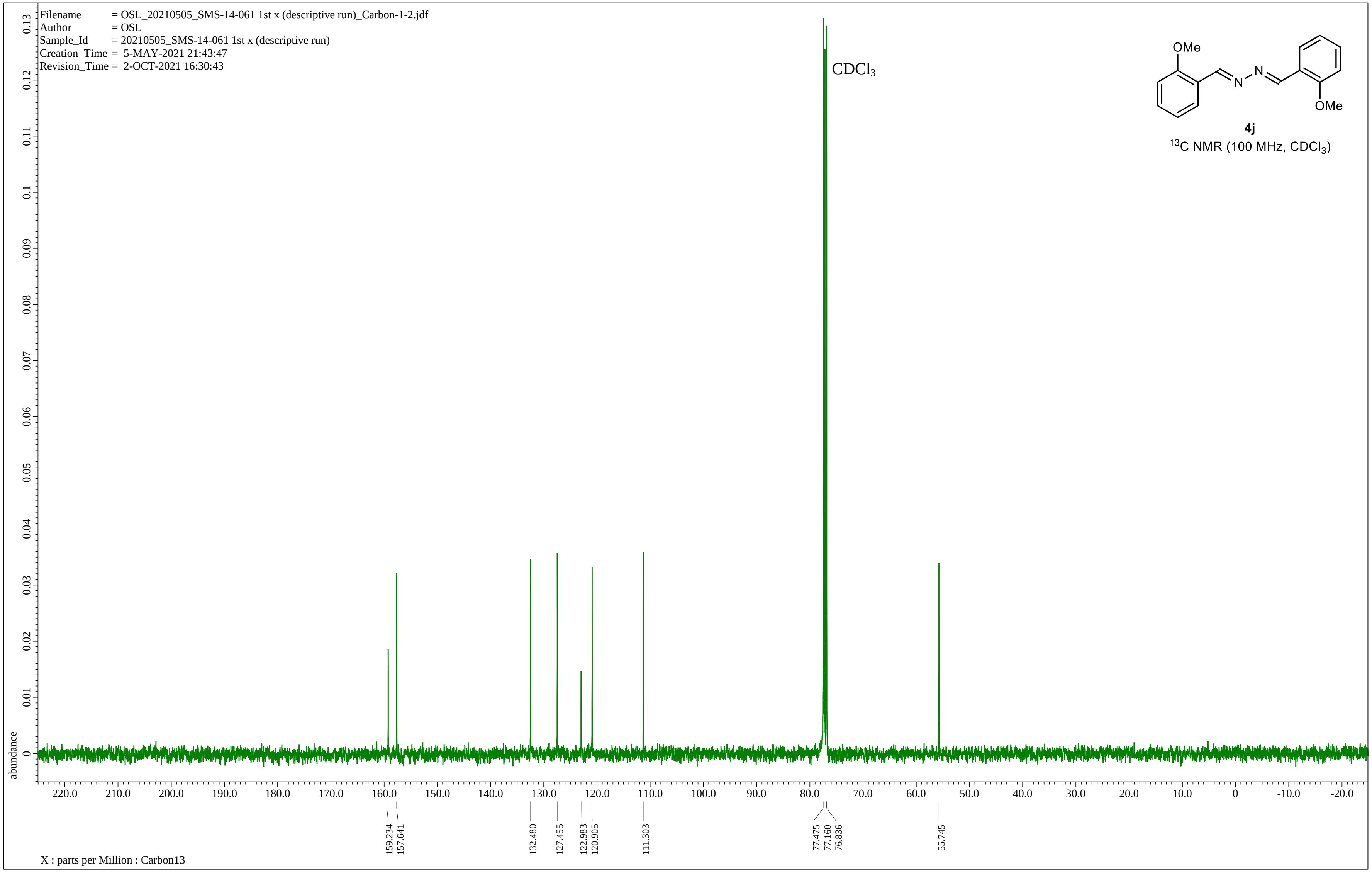

Supplementary Figure 24. ^{13}C NMR spectrum of compound **4j**, recorded at 100 MHz and 298 K in $CDCl_3$.

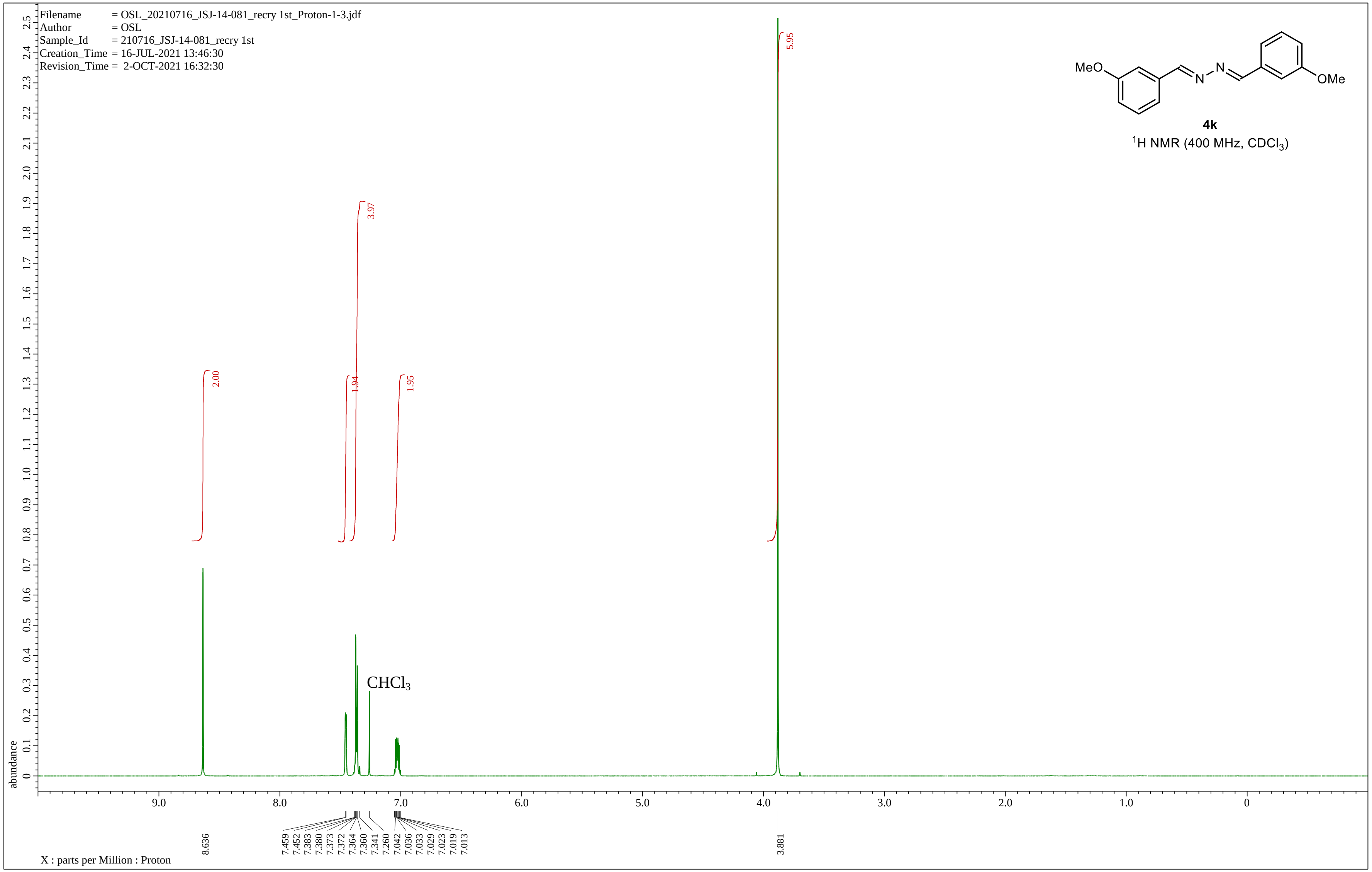

Supplementary Figure 25. ^{1}H NMR spectrum of compound **4k**, recorded at 400 MHz and 298 K in $CDCl_3$.

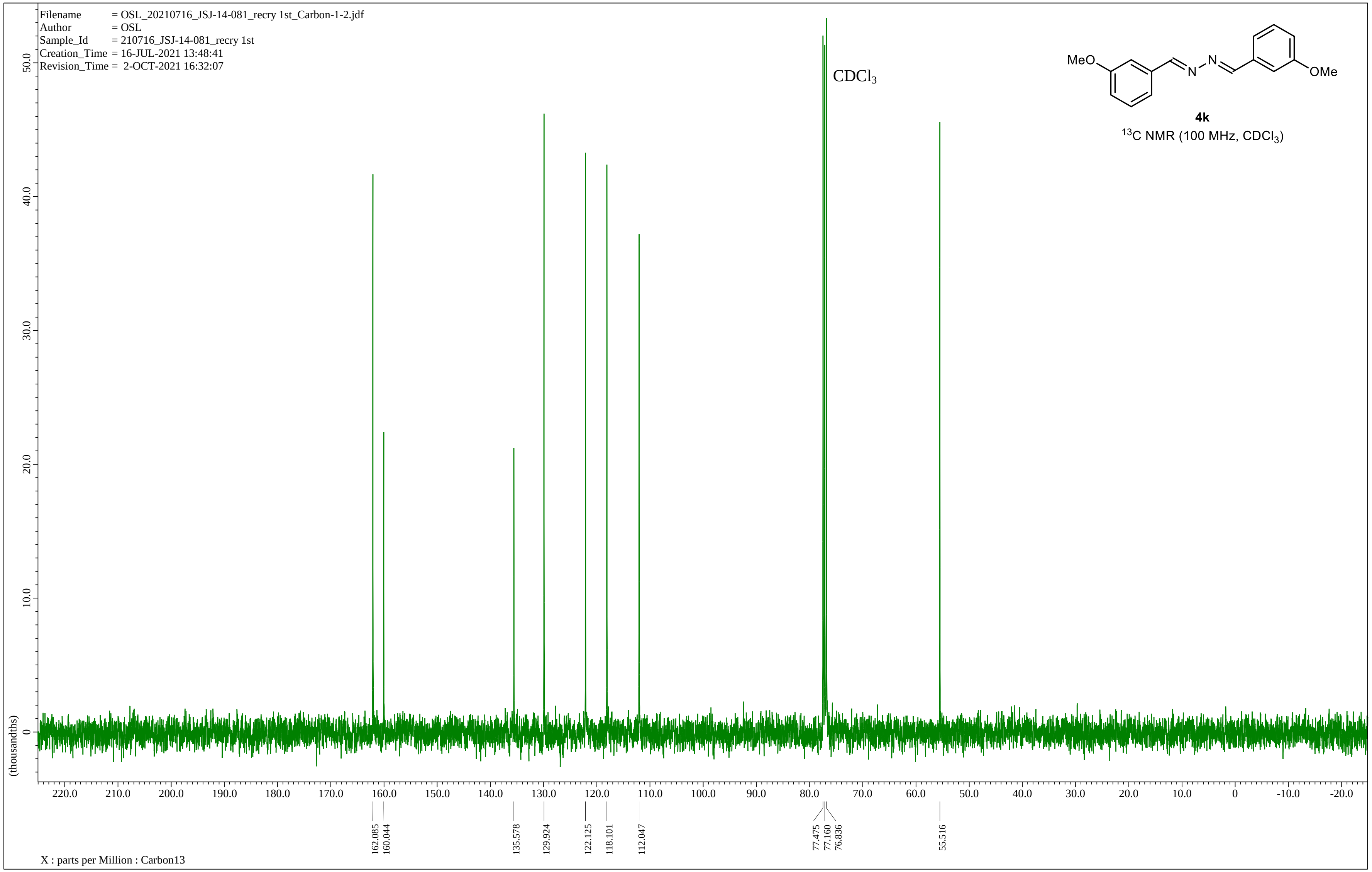

Supplementary Figure 26. ^{13}C NMR spectrum of compound **4k**, recorded at 100 MHz and 298 K in $CDCl_3$.

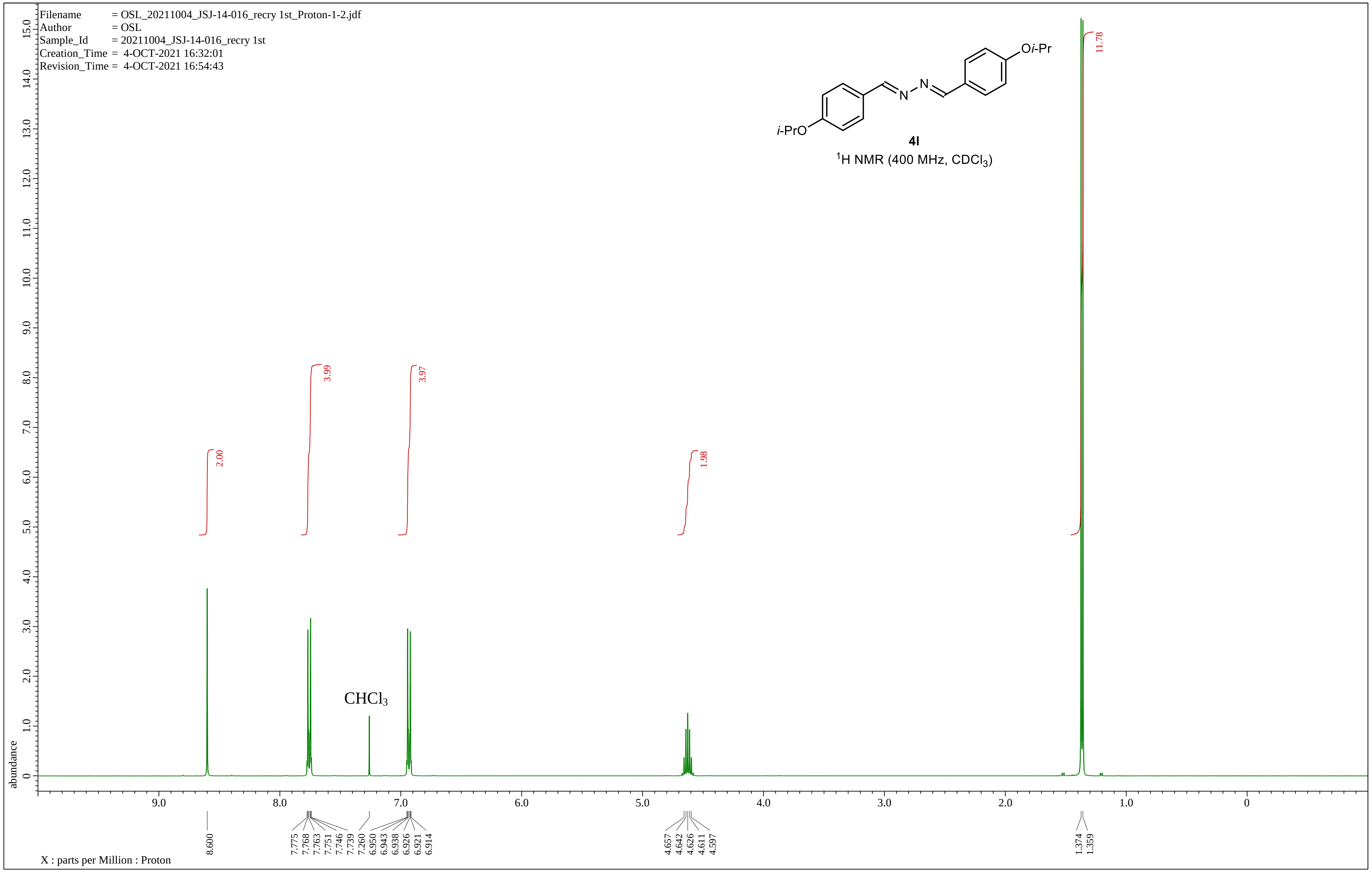

Supplementary Figure 27. ^{1}H NMR spectrum of compound **4l**, recorded at 400 MHz and 298 K in $CDCl_3$.

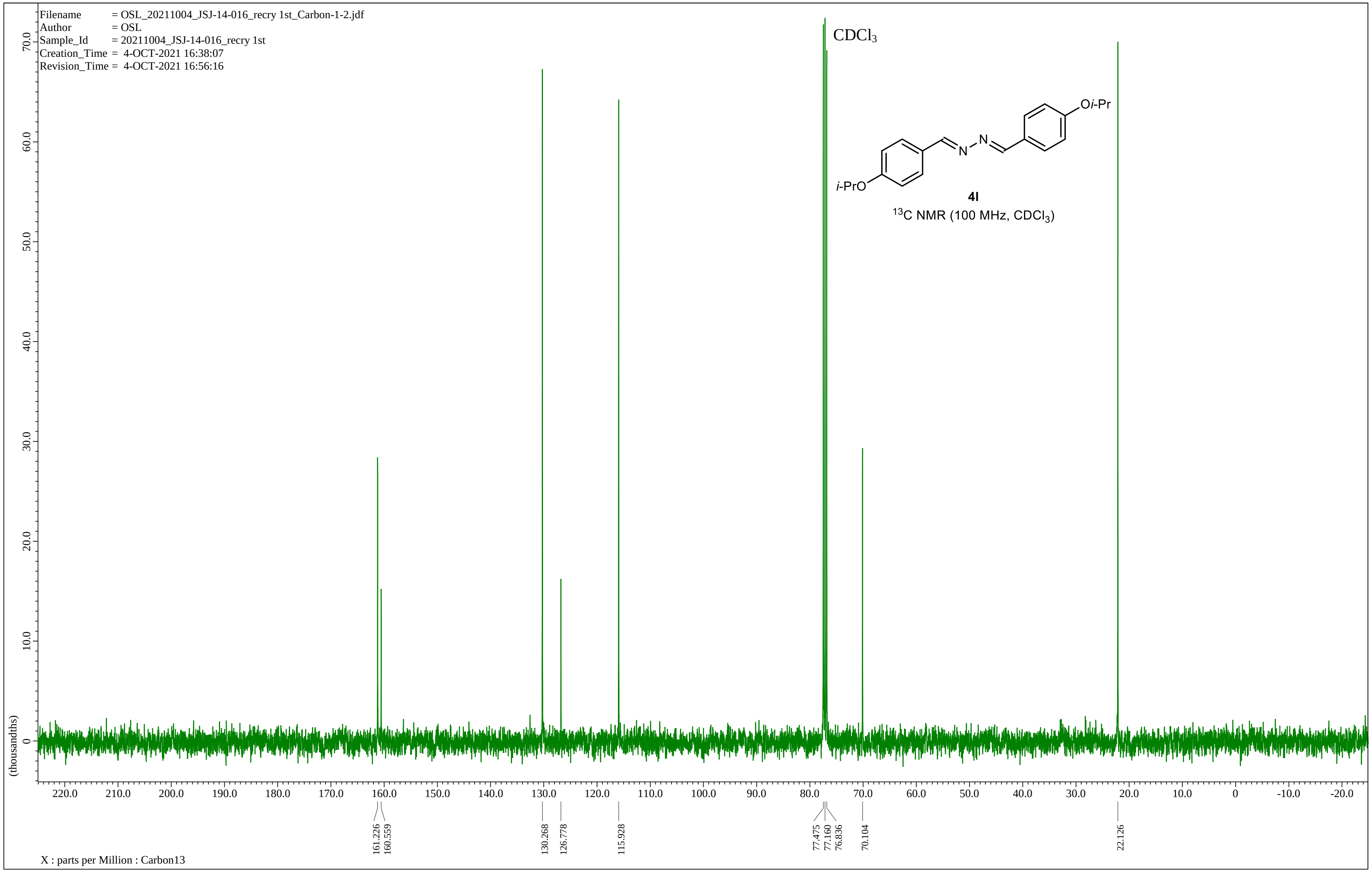

Supplementary Figure 28. ^{13}C NMR spectrum of compound **4l**, recorded at 100 MHz and 298 K in $CDCl_3$.

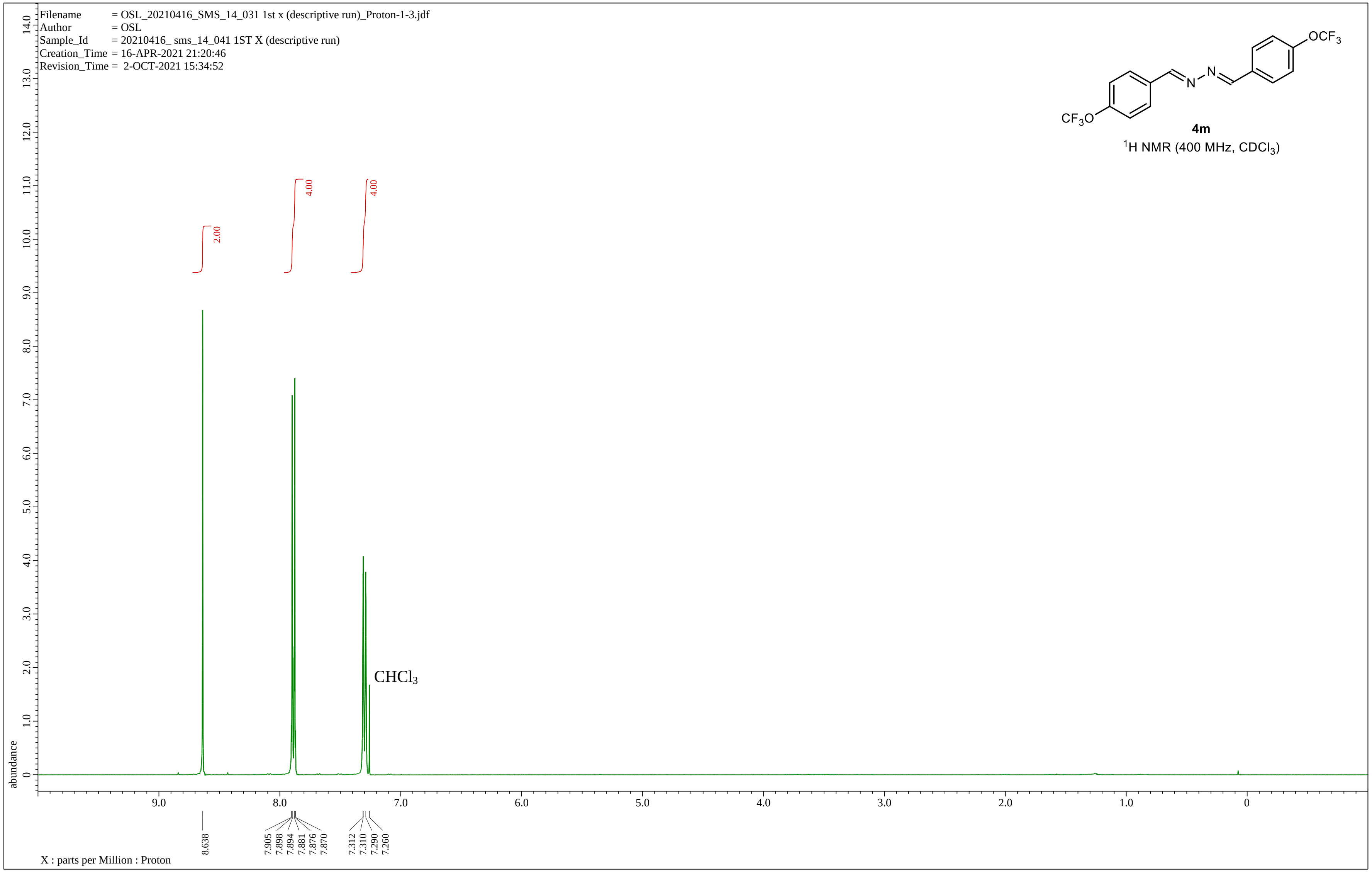

Supplementary Figure 29. ^{1}H NMR spectrum of compound **4m**, recorded at 400 MHz and 298 K in $CDCl_3$.

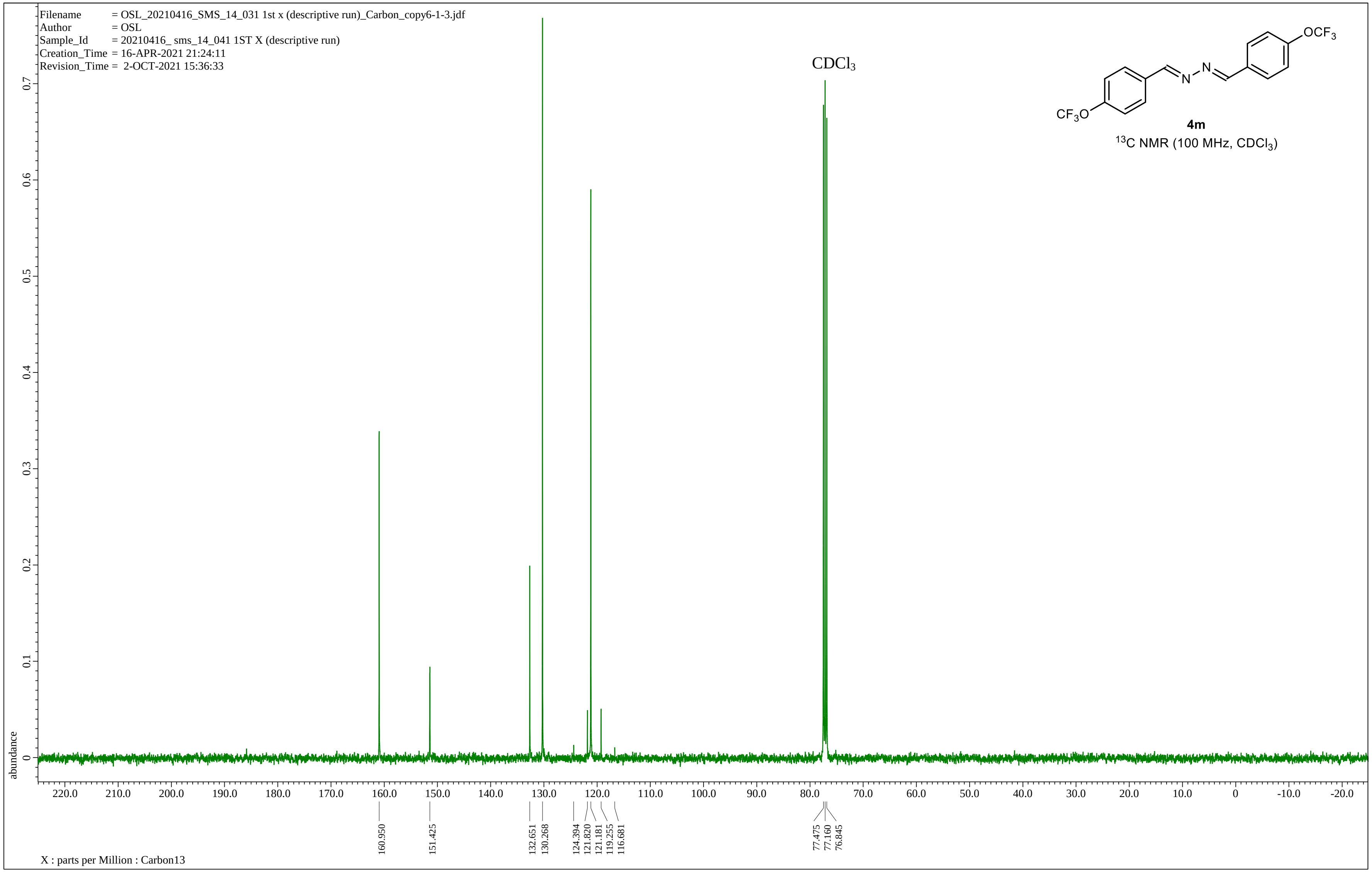

Supplementary Figure 30. ^{13}C NMR spectrum of compound **4m**, recorded at 100 MHz and 298 K in $CDCl_3$.

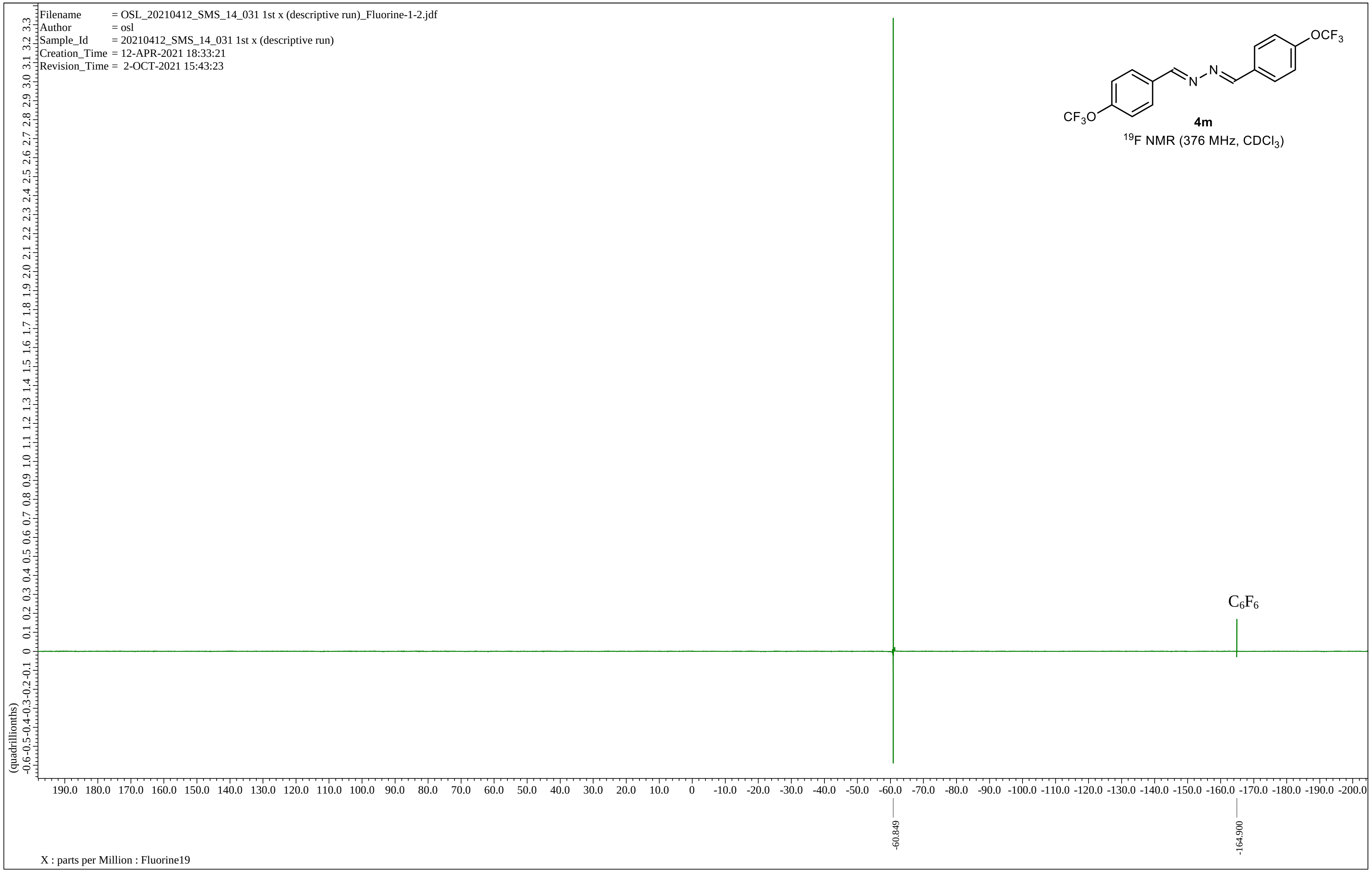

Supplementary Figure 31. ^{19}F NMR spectrum of compound **4m**, recorded at 376 MHz and 298 K in $CDCl_3$.

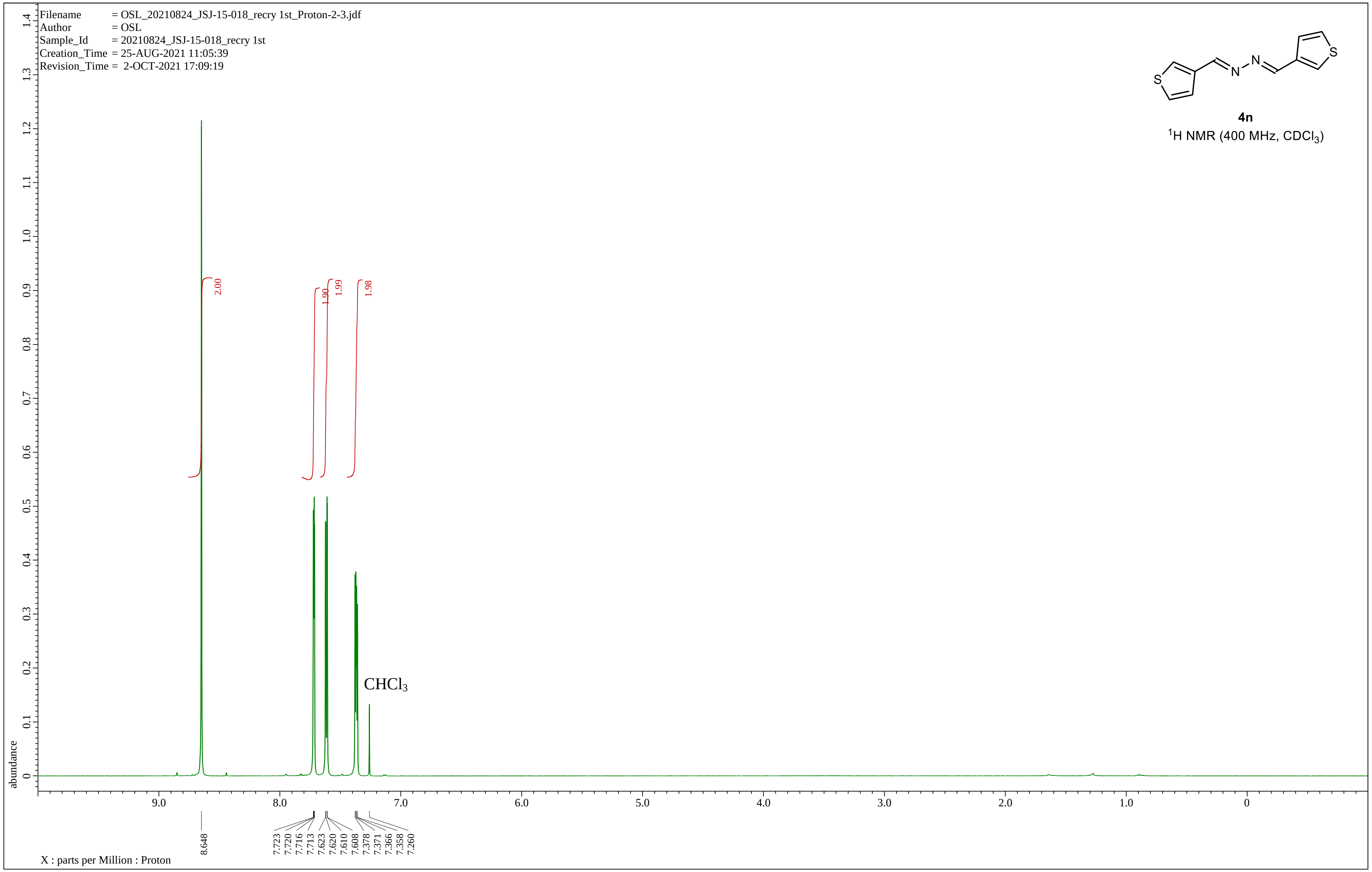

Supplementary Figure 32. ^{1}H NMR spectrum of compound **4n**, recorded at 400 MHz and 298 K in $CDCl_3$.

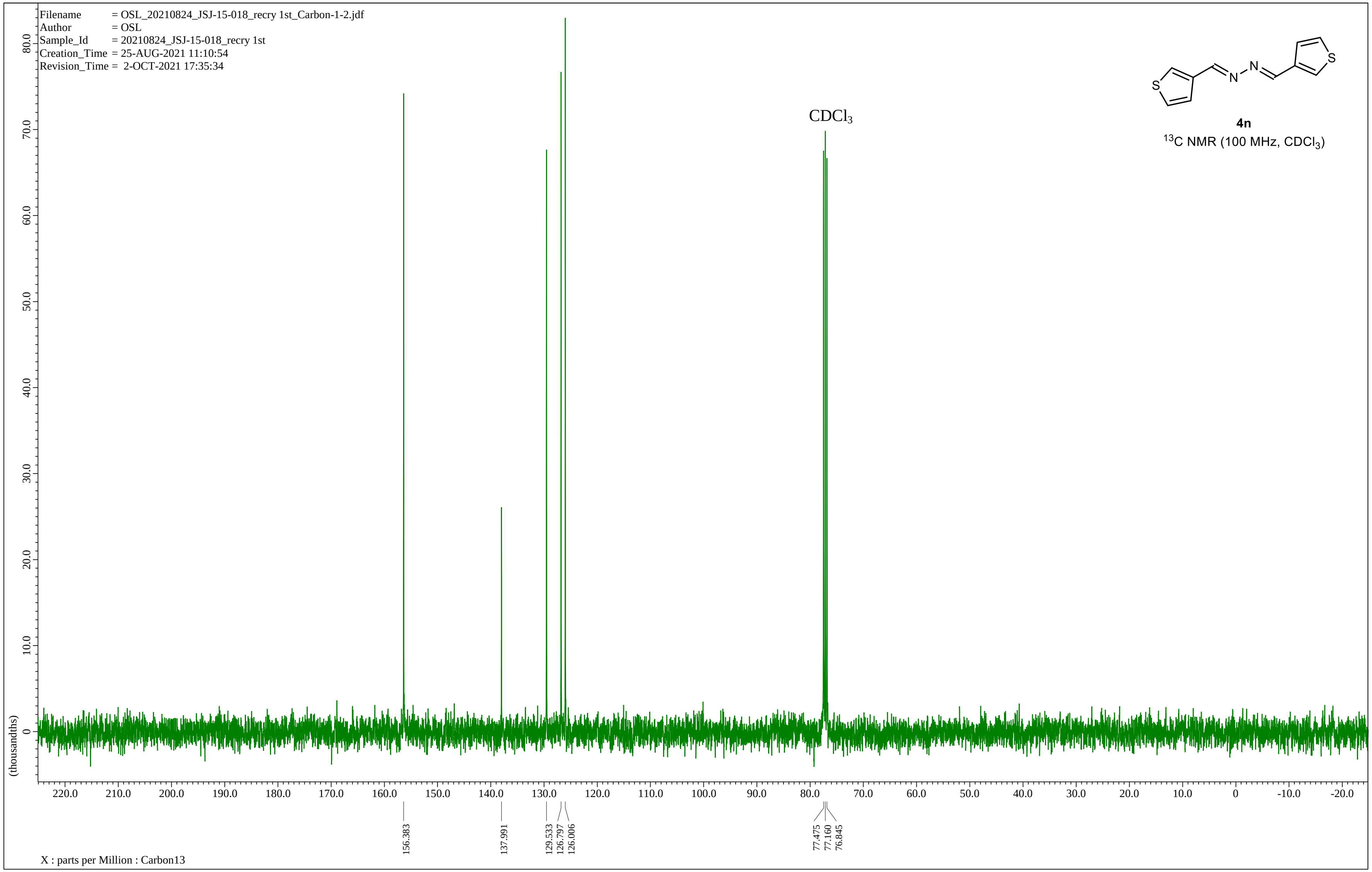

Supplementary Figure 33. ^{13}C NMR spectrum of compound **4n**, recorded at 100 MHz and 298 K in $CDCl_3$.

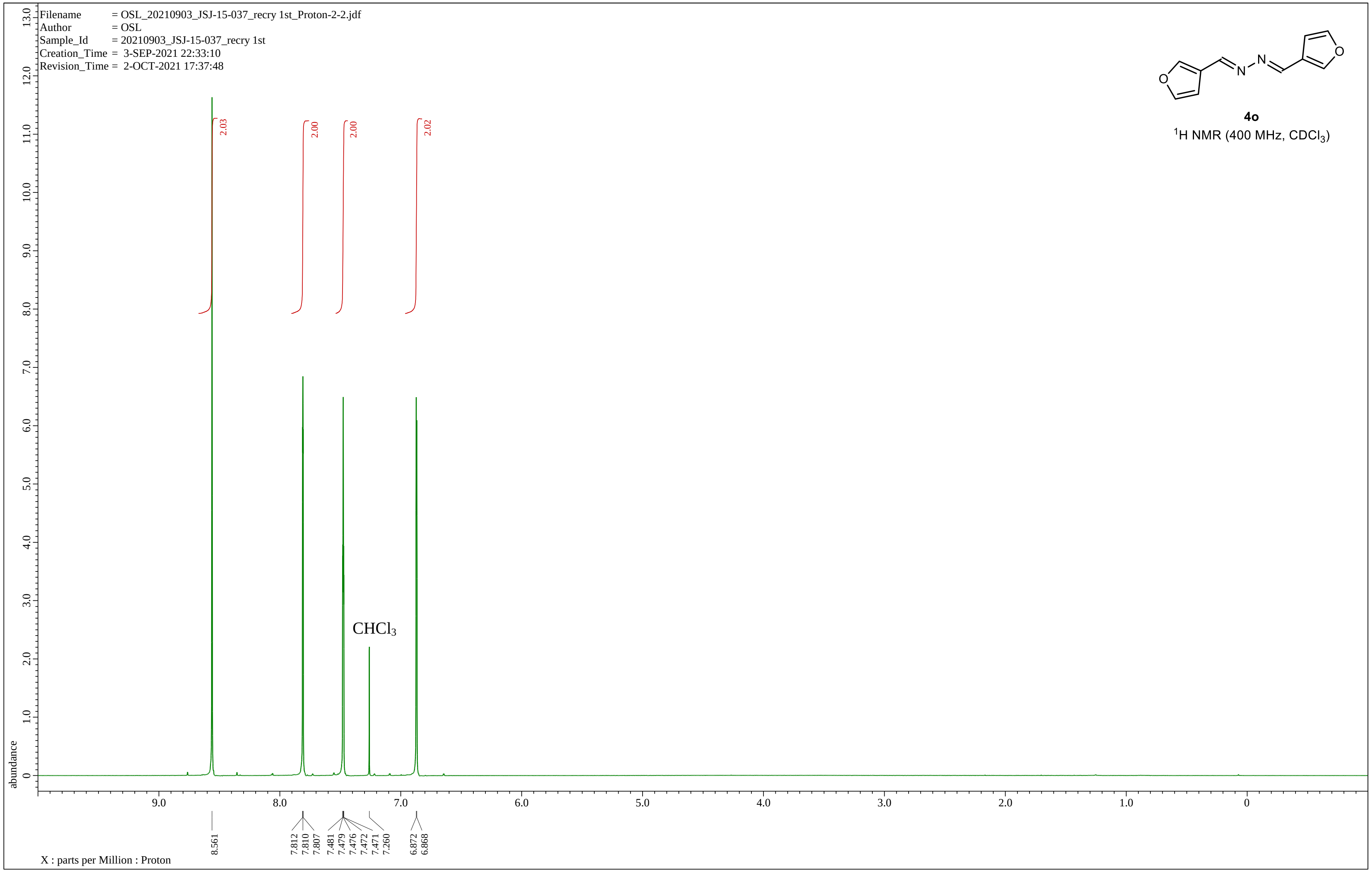

Supplementary Figure 34. ^{1}H NMR spectrum of compound **4o**, recorded at 400 MHz and 298 K in $CDCl_3$.

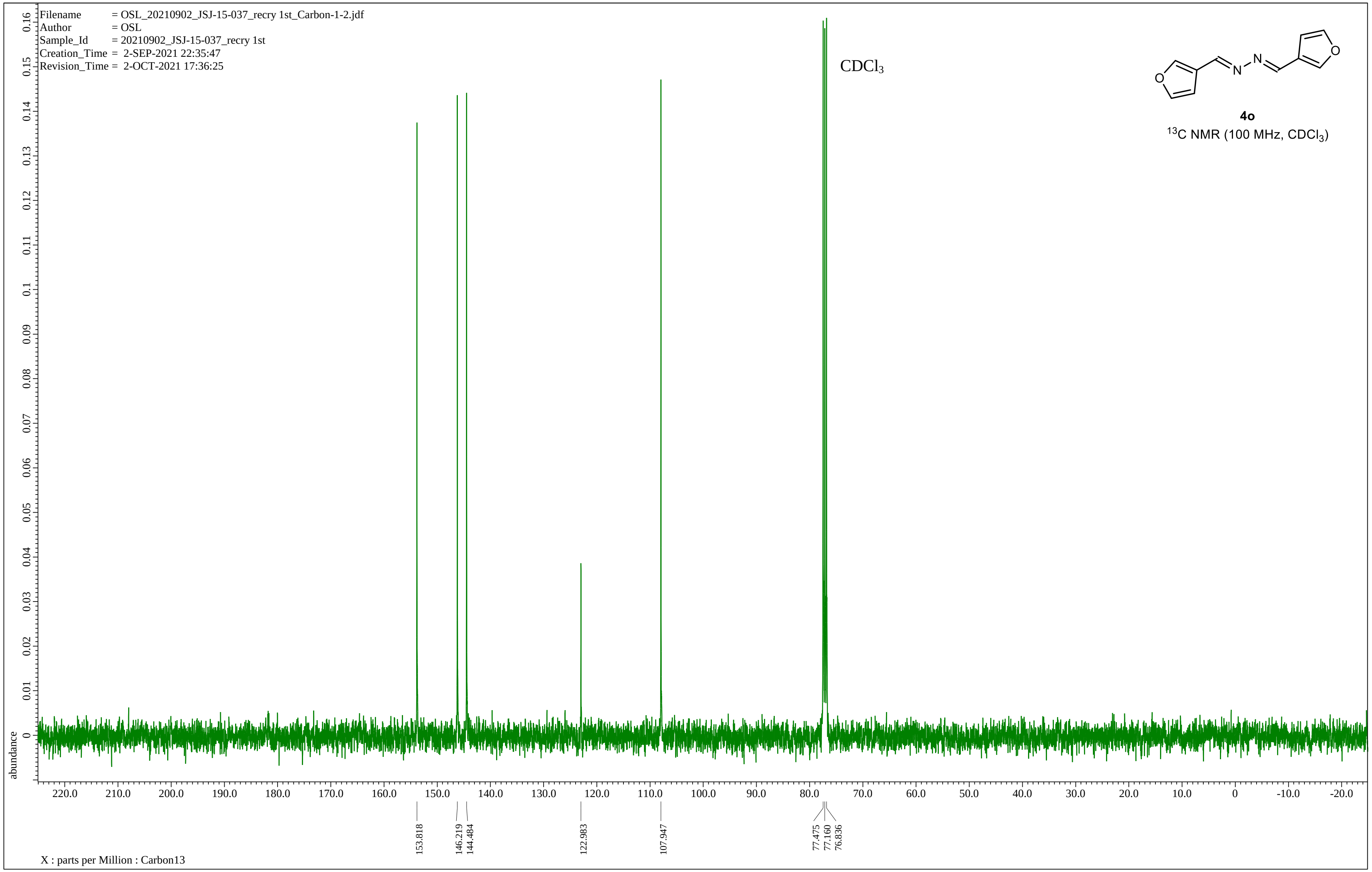

Supplementary Figure 35. ^{13}C NMR spectrum of compound **4o**, recorded at 100 MHz and 298 K in $CDCl_3$.

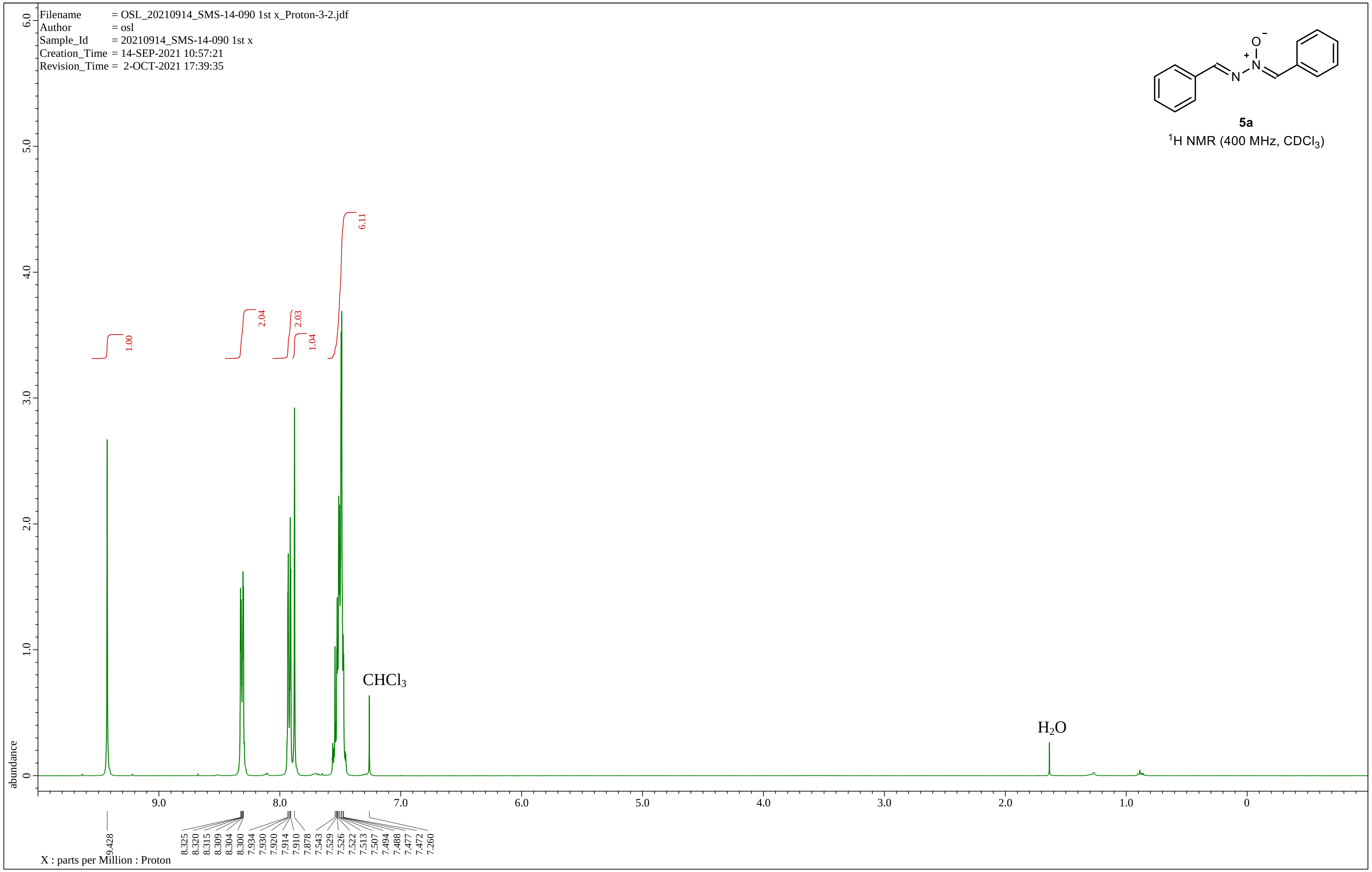

Supplementary Figure 36. ^{1}H NMR spectrum of compound **5a**, recorded at 400 MHz and 298 K in $CDCl_3$.

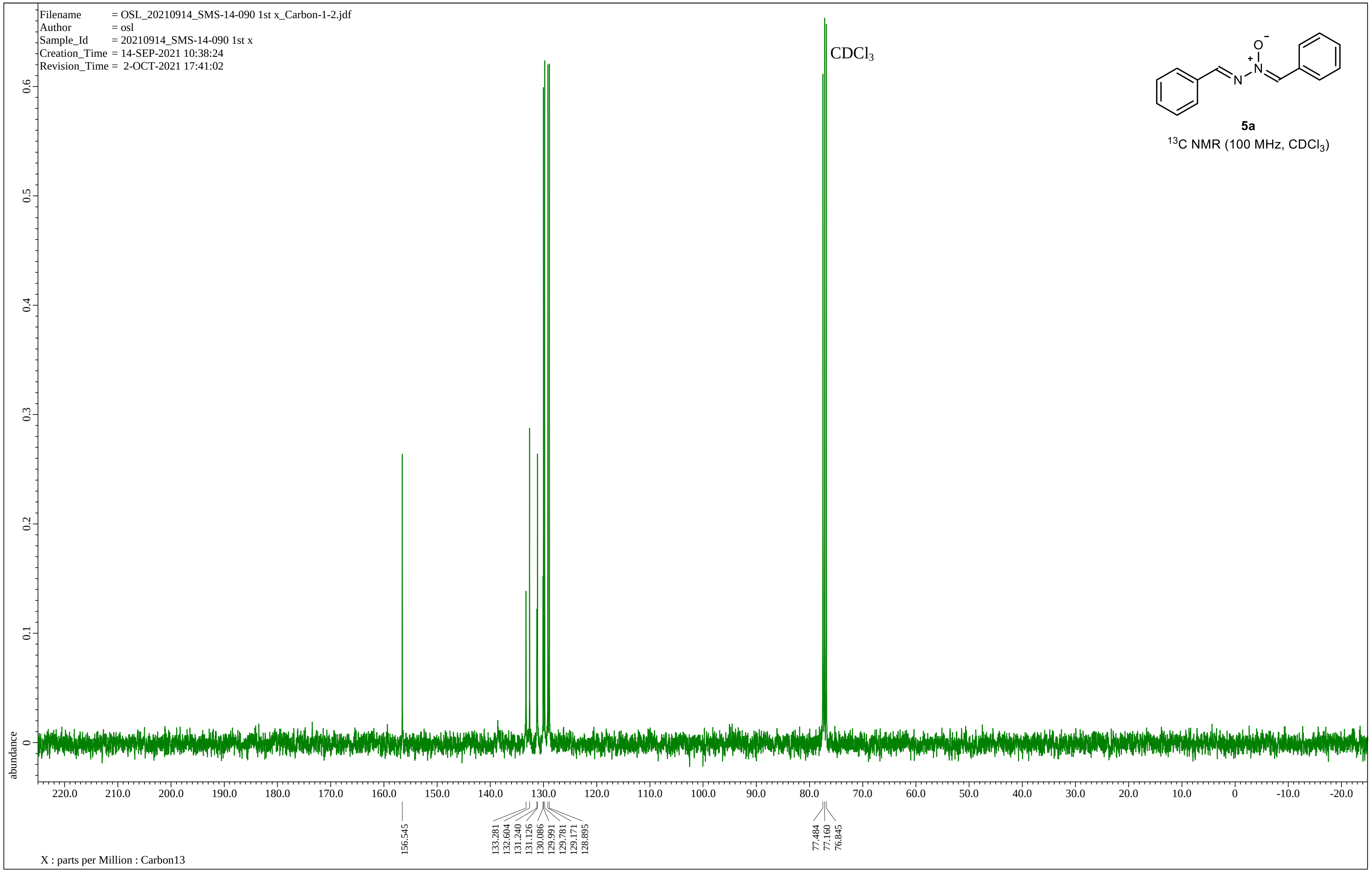

Supplementary Figure 37. ^{13}C NMR spectrum of compound **5a**, recorded at 100 MHz and 298 K in $CDCl_3$.

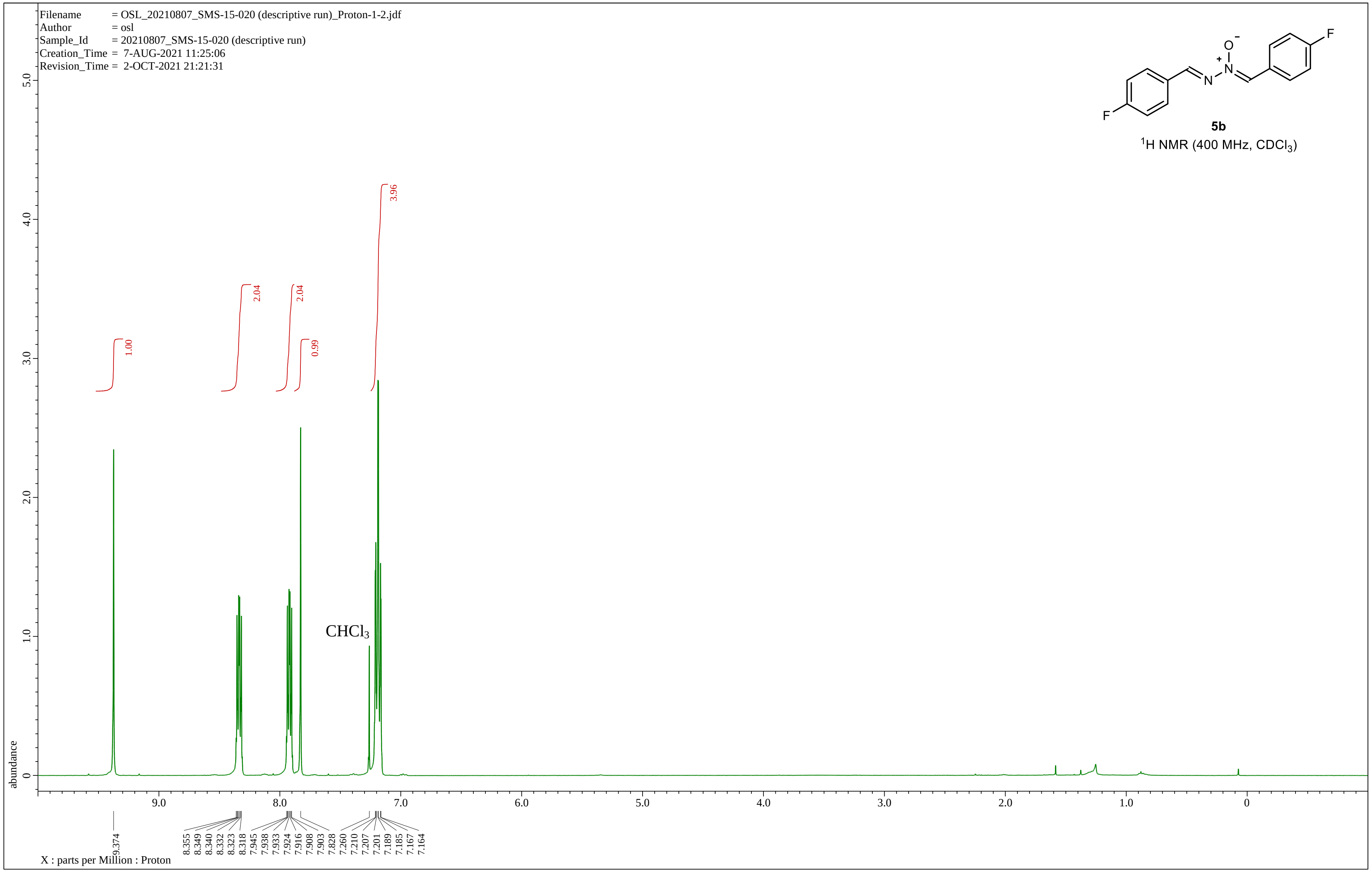

Supplementary Figure 38. ^{1}H NMR spectrum of compound **5b**, recorded at 400 MHz and 298 K in $CDCl_3$.

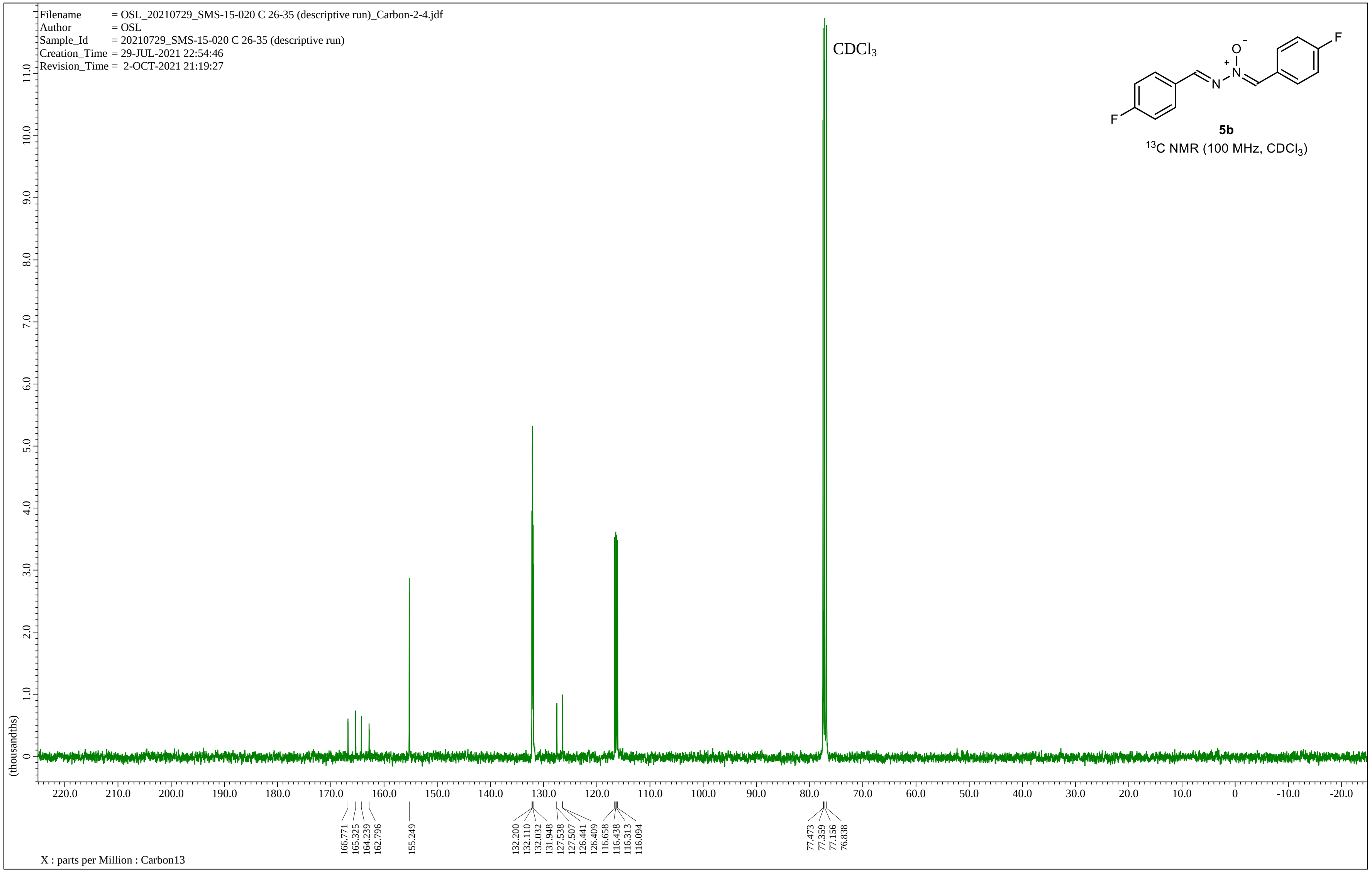

Supplementary Figure 39. ^{13}C NMR spectrum of compound **5b**, recorded at 100 MHz and 298 K in $CDCl_3$.

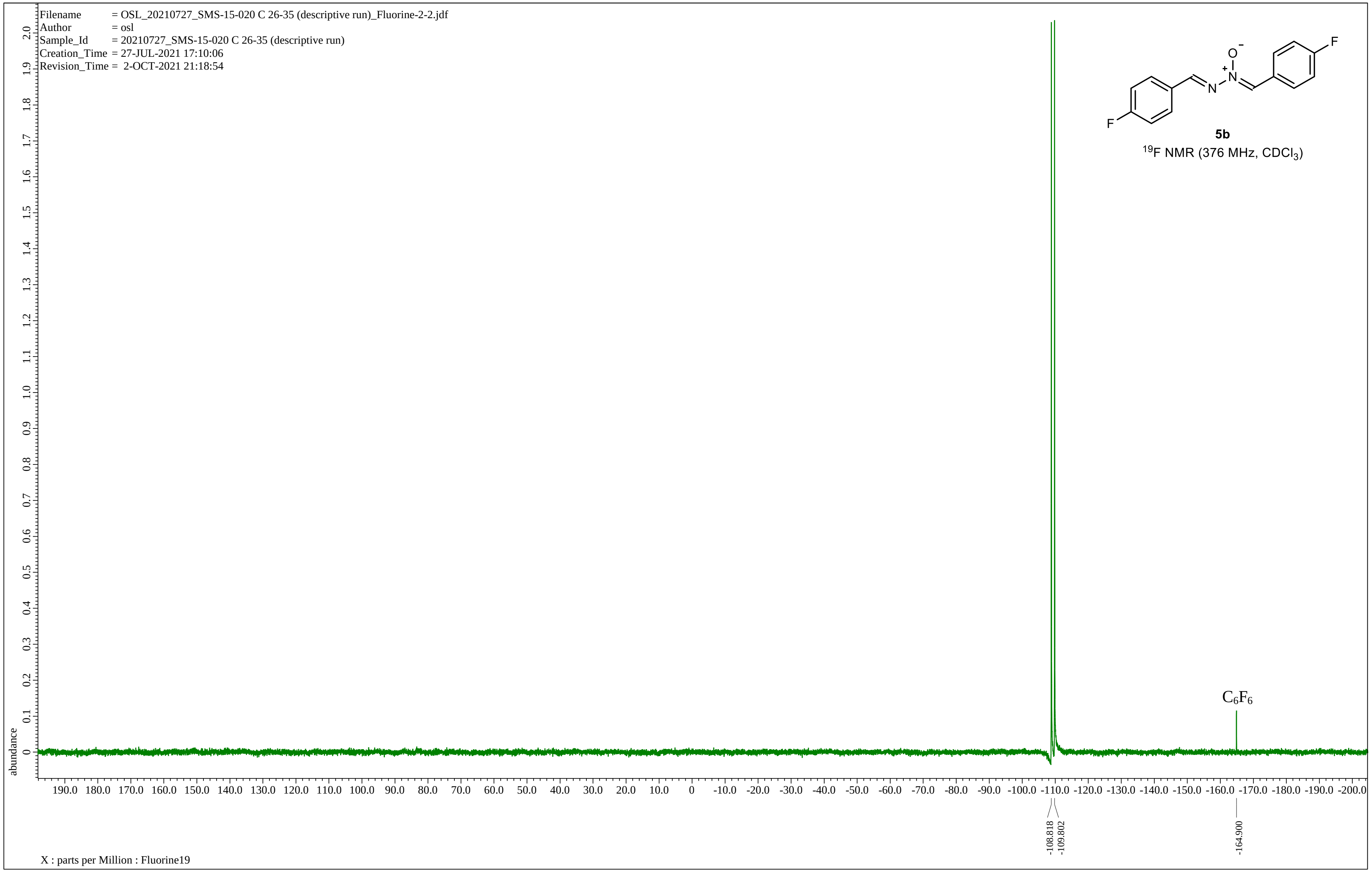

Supplementary Figure 40. ^{19}F NMR spectrum of compound **5b**, recorded at 376 MHz and 298 K in $CDCl_3$.

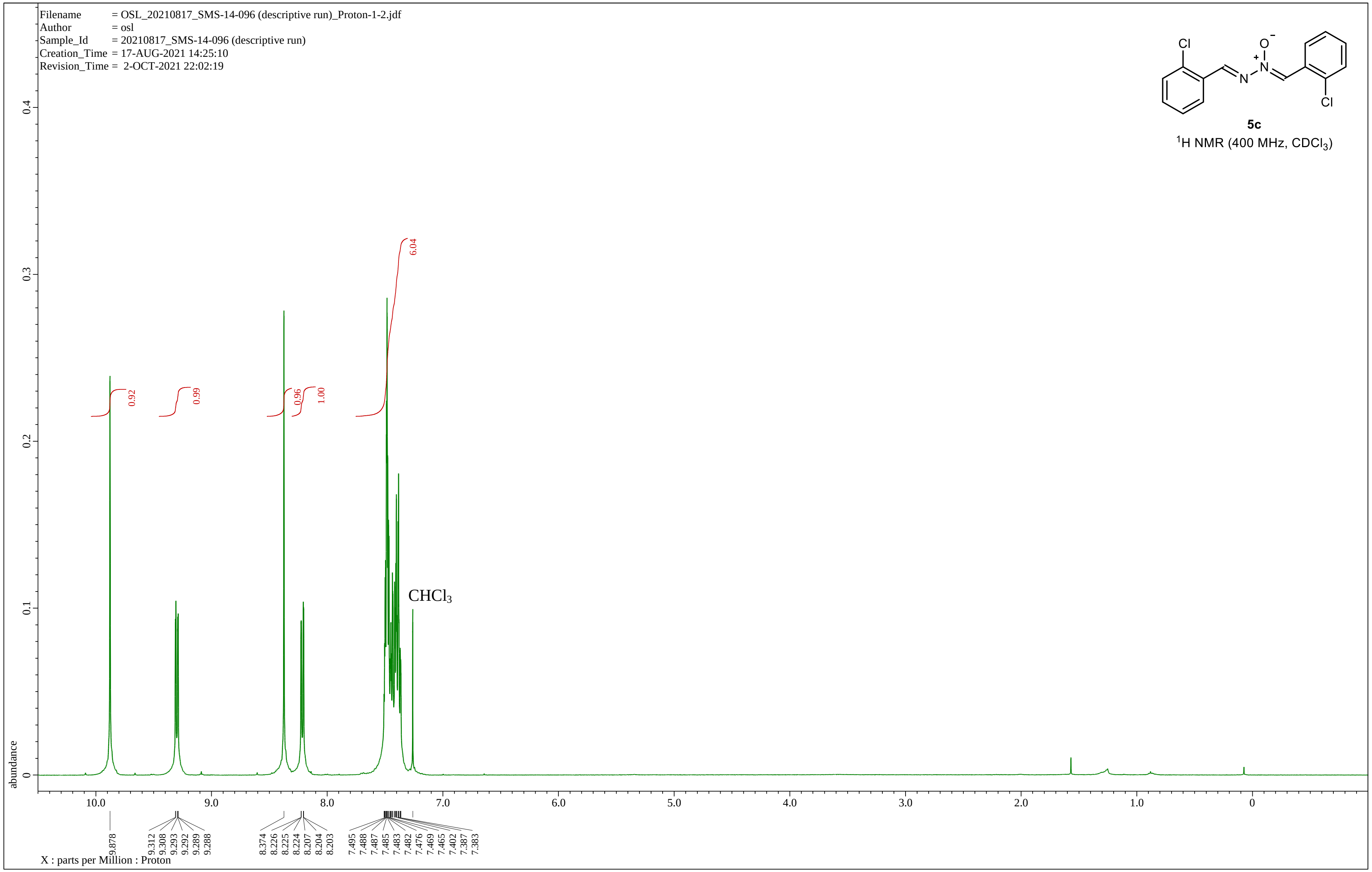

Supplementary Figure 41. ^{1}H NMR spectrum of compound **5c**, recorded at 400 MHz and 298 K in $CDCl_3$.

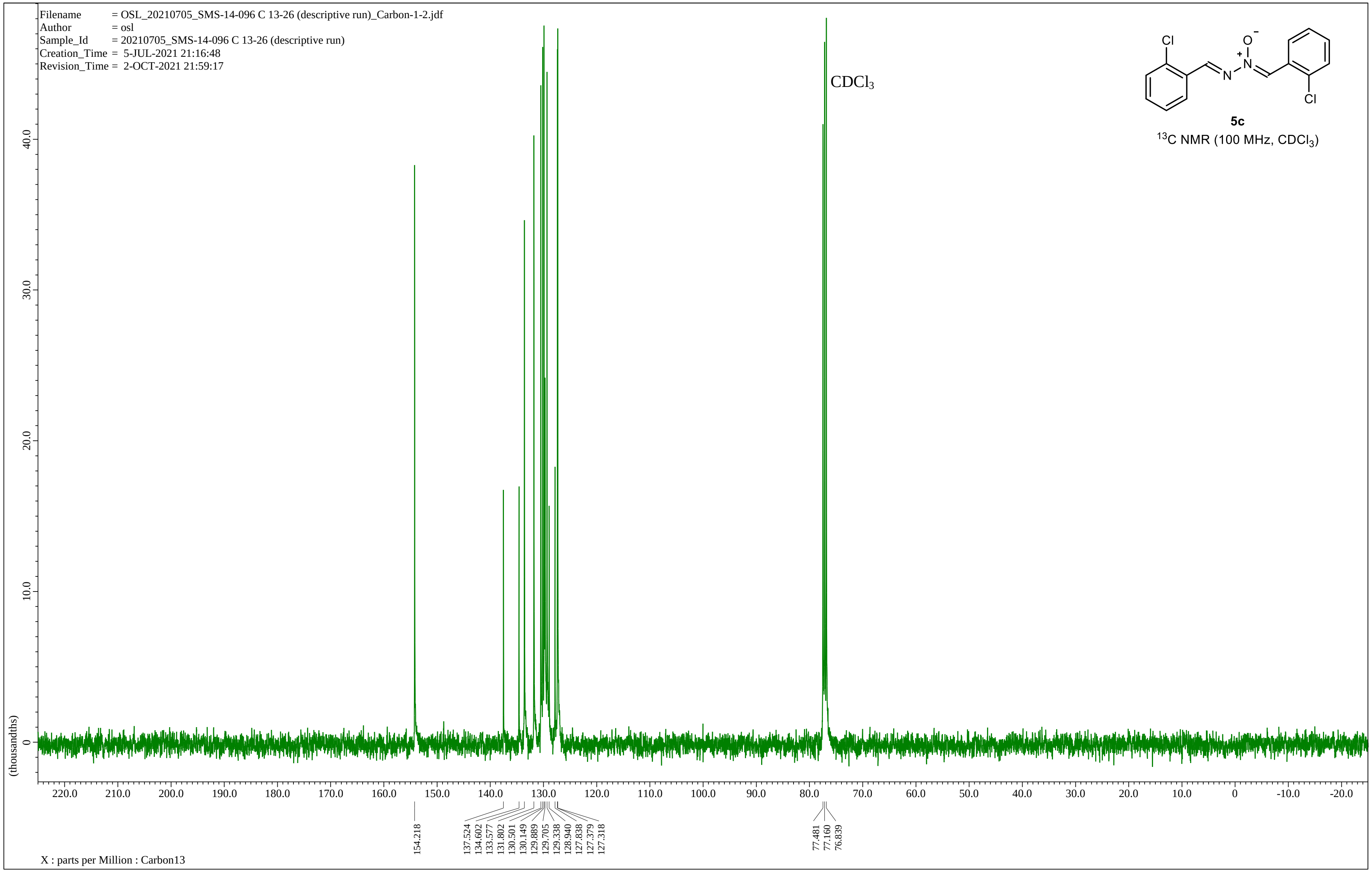

Supplementary Figure 42. ^{13}C NMR spectrum of compound **5c**, recorded at 100 MHz and 298 K in $CDCl_3$.

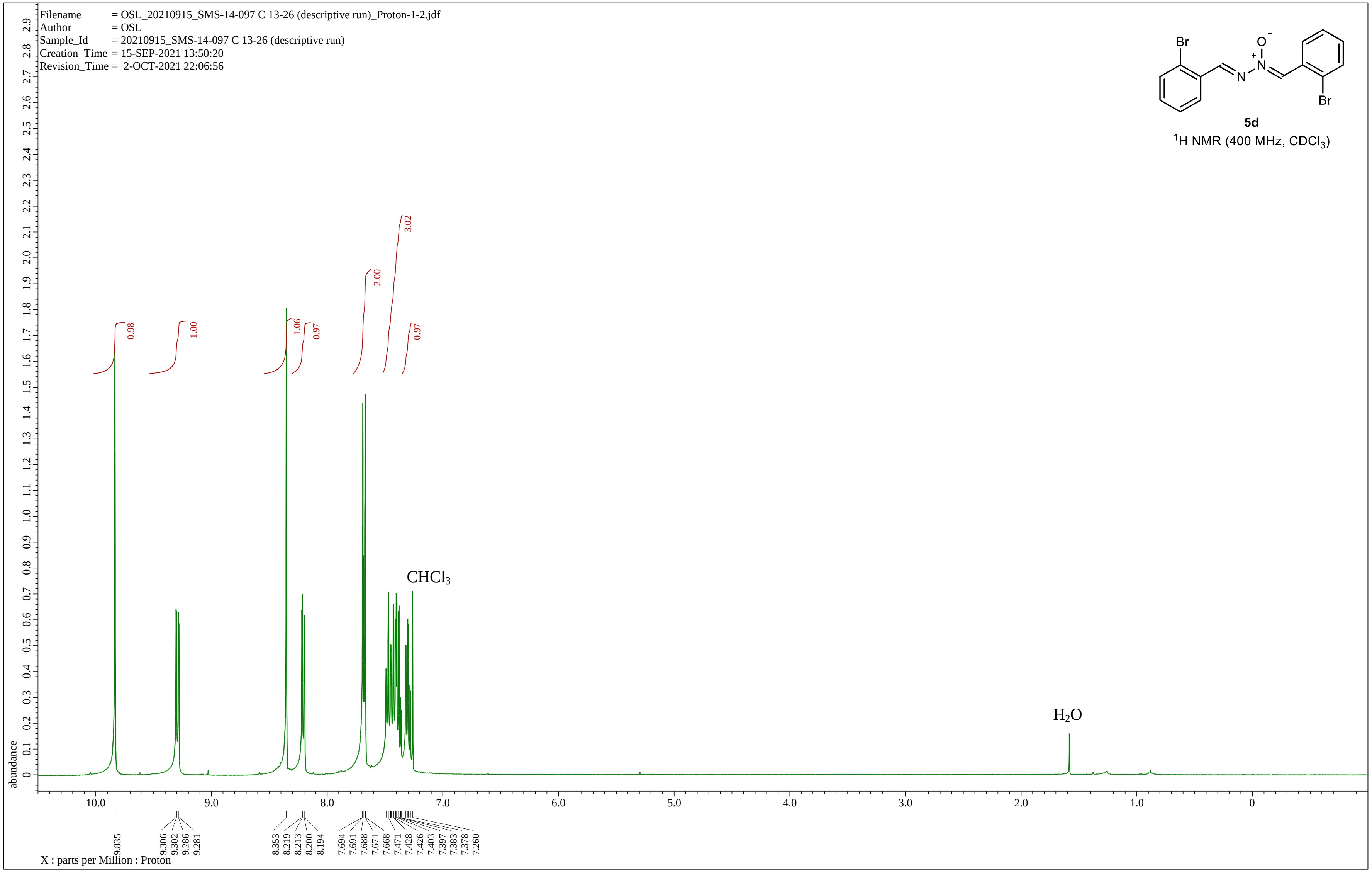

Supplementary Figure 43. ^{1}H NMR spectrum of compound **5d**, recorded at 400 MHz and 298 K in $CDCl_3$.

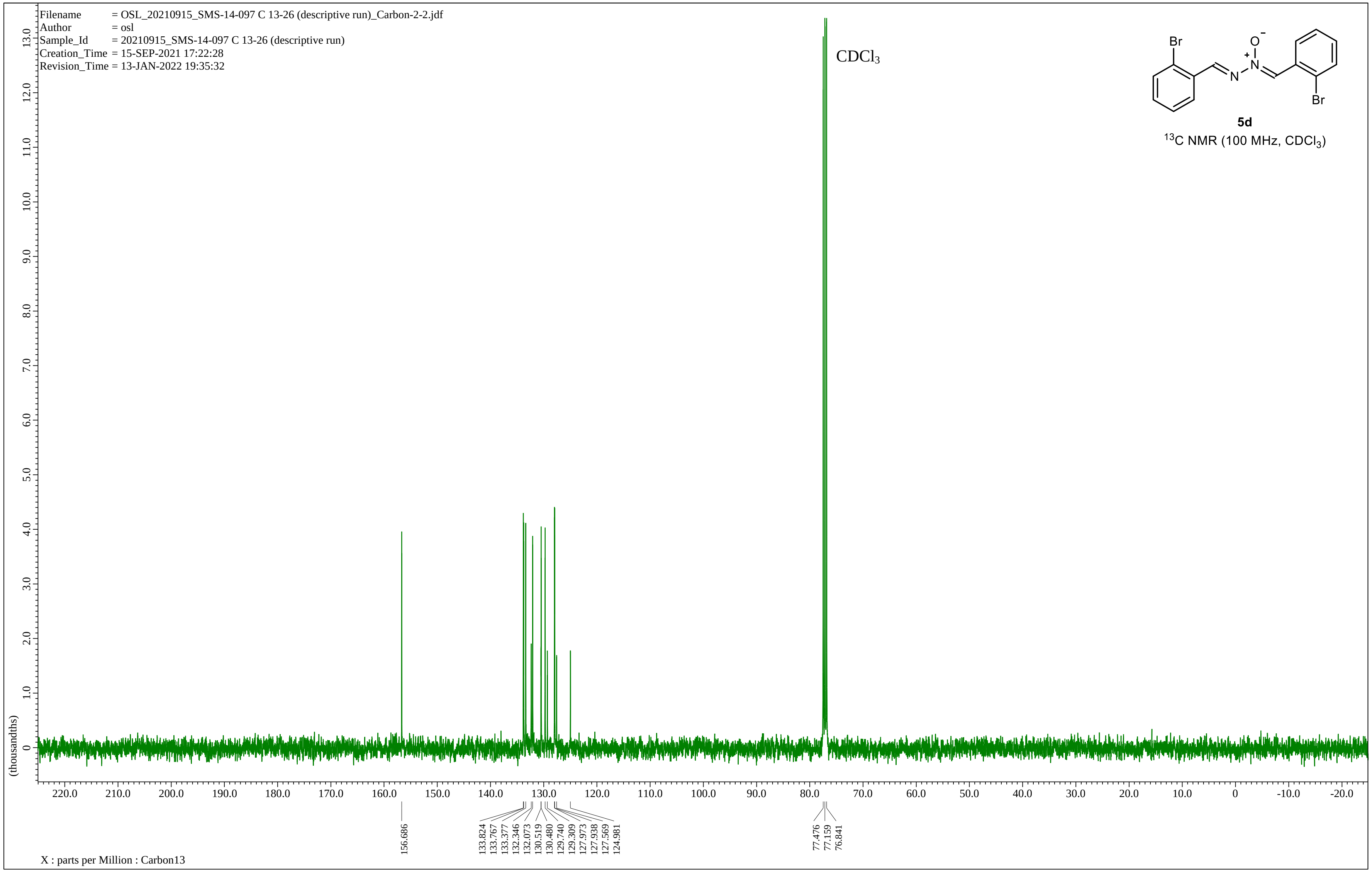

Supplementary Figure 44. ^{13}C NMR spectrum of compound **5d**, recorded at 100 MHz and 298 K in $CDCl_3$.

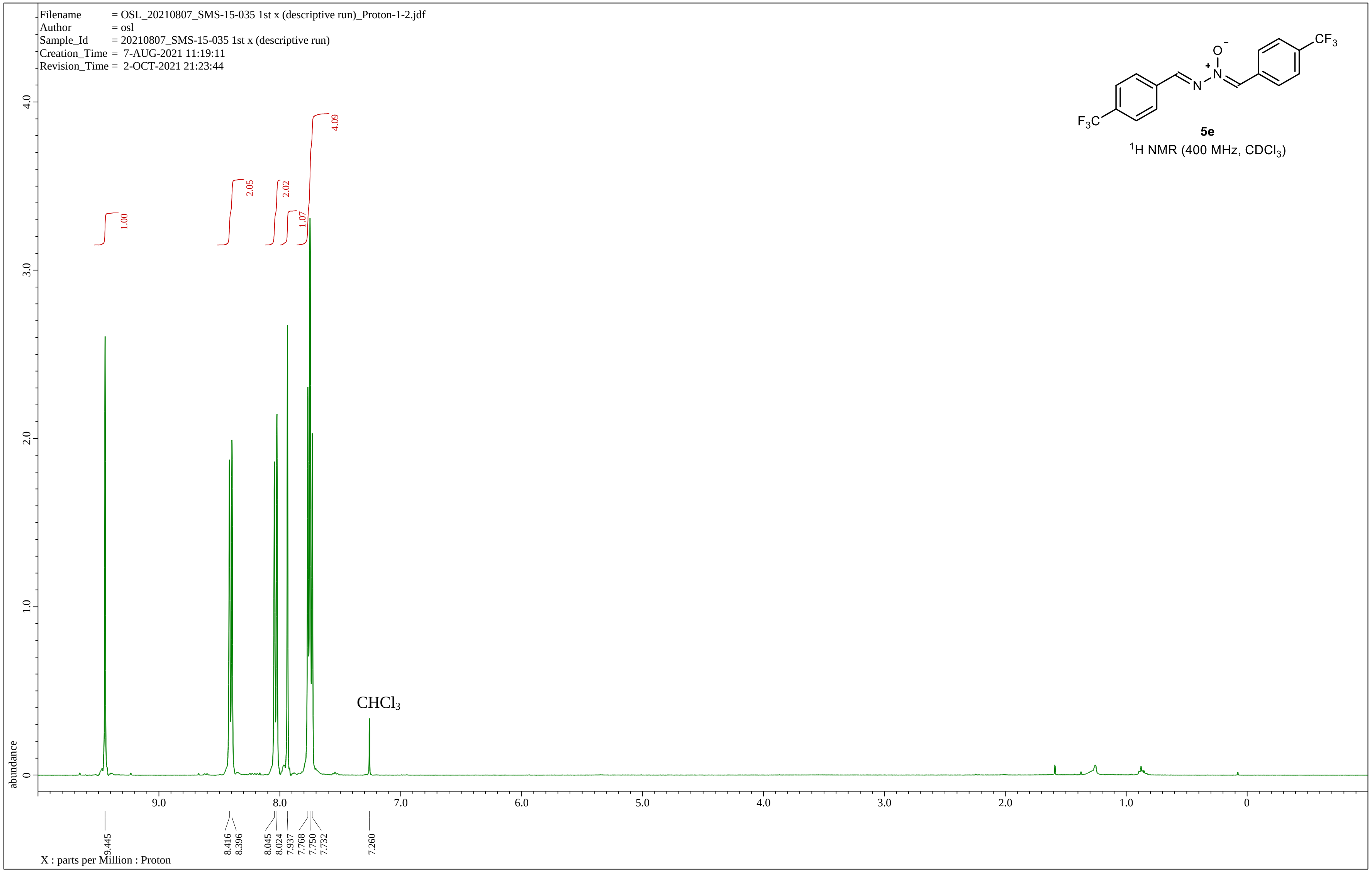

Supplementary Figure 45. ^{1}H NMR spectrum of compound **5e**, recorded at 400 MHz and 298 K in $CDCl_3$.

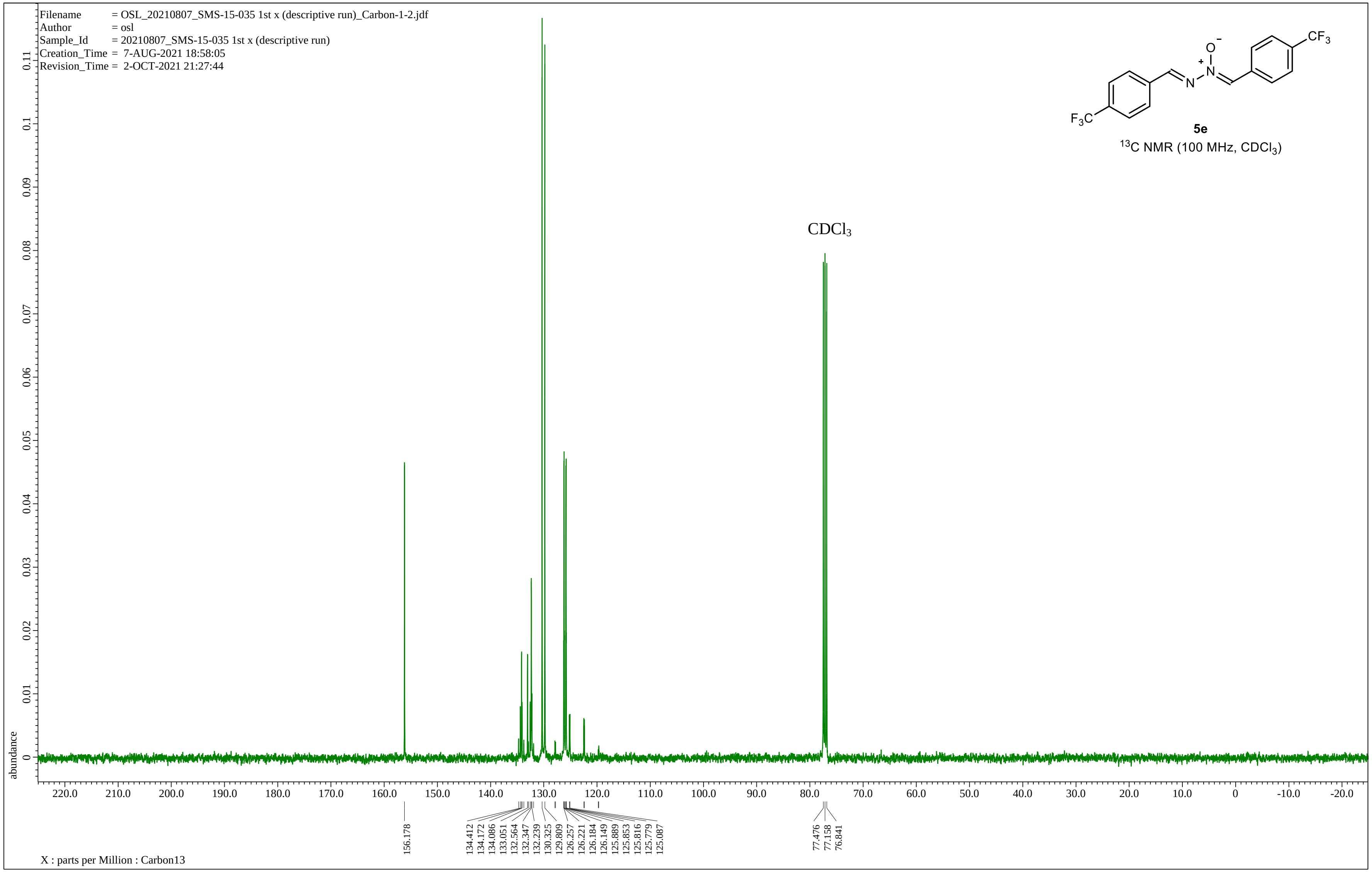

Supplementary Figure 46. ^{13}C NMR spectrum of compound **5e**, recorded at 100 MHz and 298 K in $CDCl_3$.

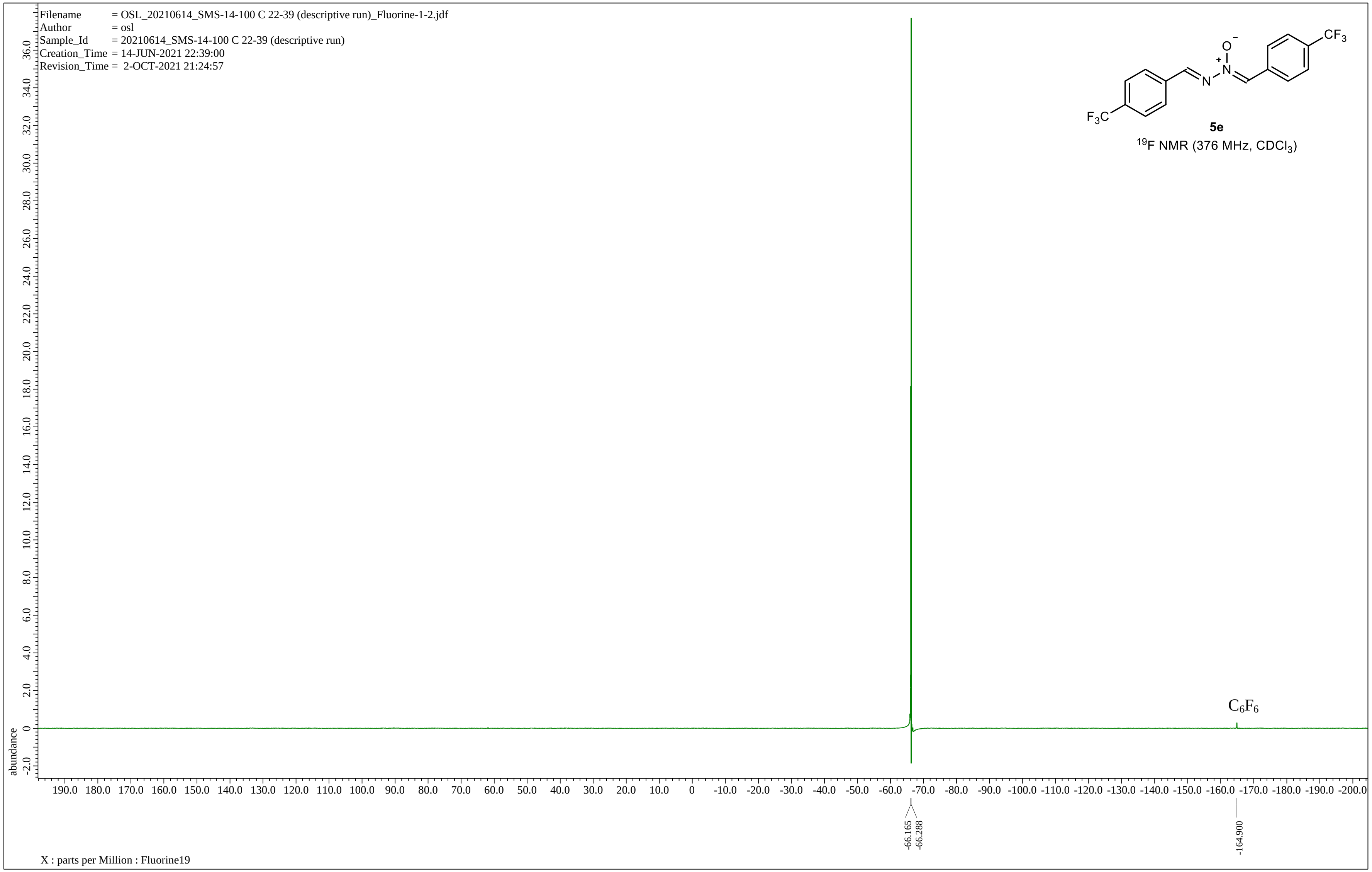

Supplementary Figure 47. ^{19}F NMR spectrum of compound **5e**, recorded at 376 MHz and 298 K in $CDCl_3$.

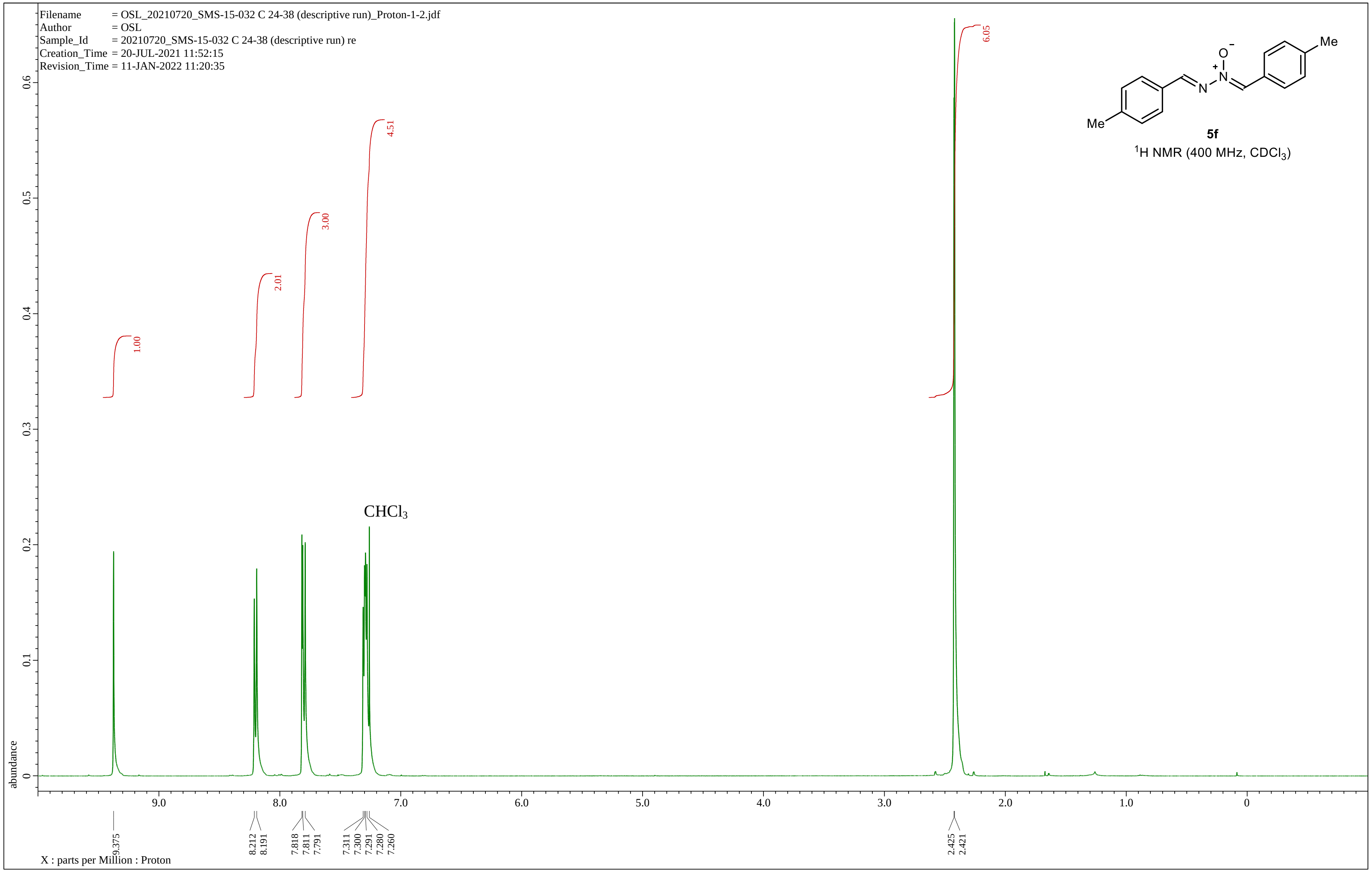

Supplementary Figure 48. ^{1}H NMR spectrum of compound **5f**, recorded at 400 MHz and 298 K in $CDCl_3$.

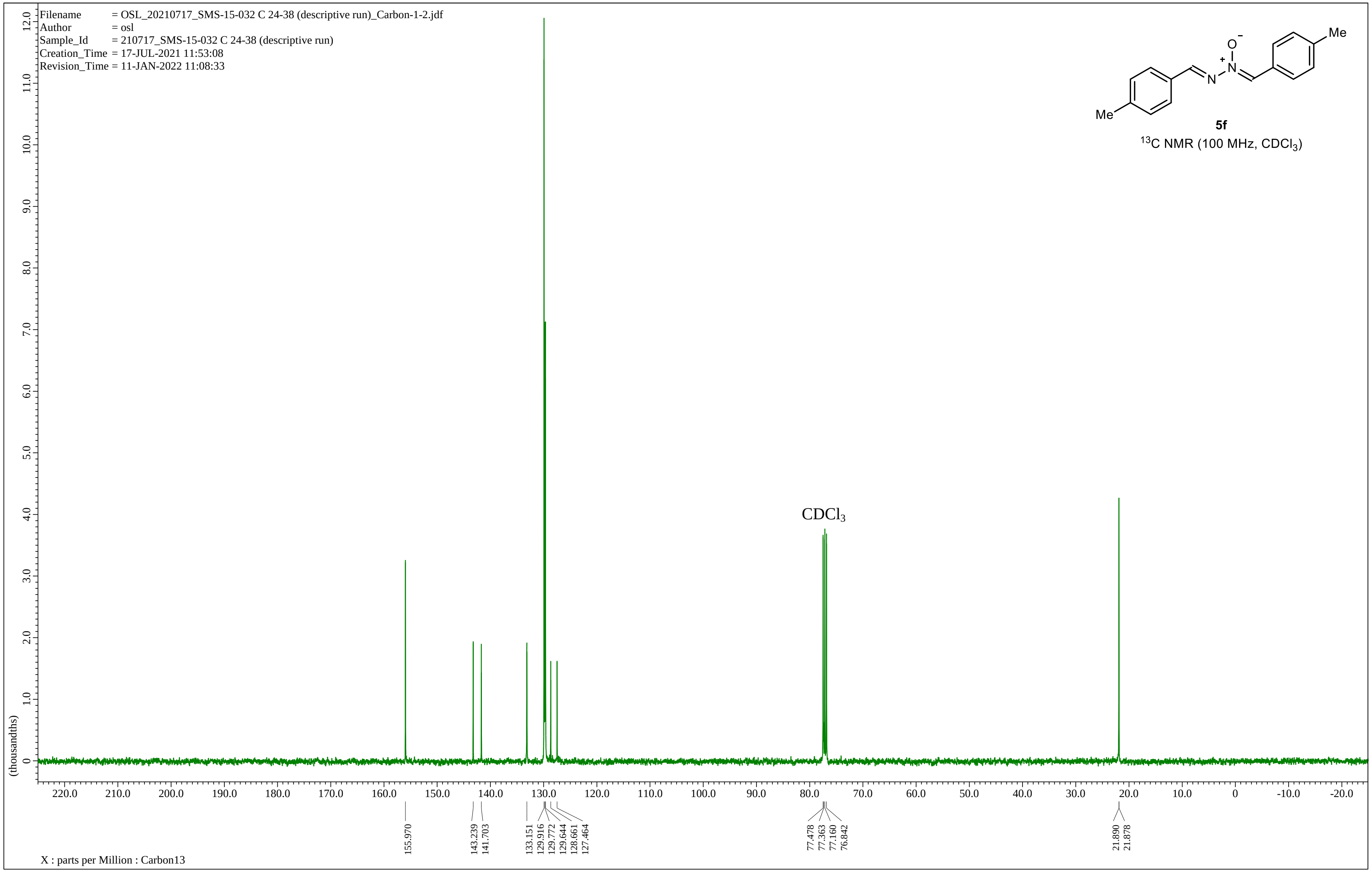

Supplementary Figure 49. ^{13}C NMR spectrum of compound **5f**, recorded at 100 MHz and 298 K in $CDCl_3$.

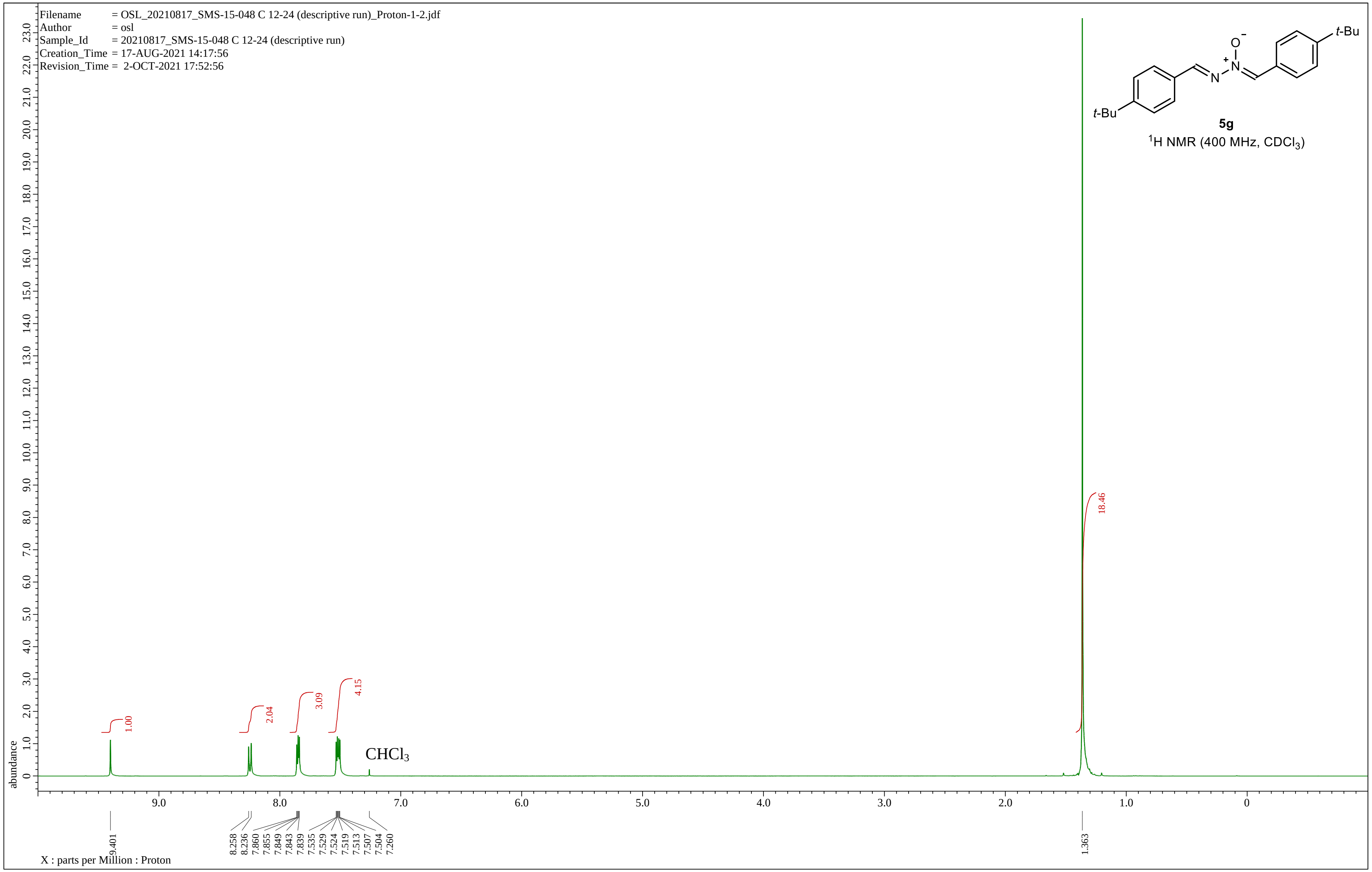

Supplementary Figure 50. ^{1}H NMR spectrum of compound **5g**, recorded at 400 MHz and 298 K in $CDCl_3$.

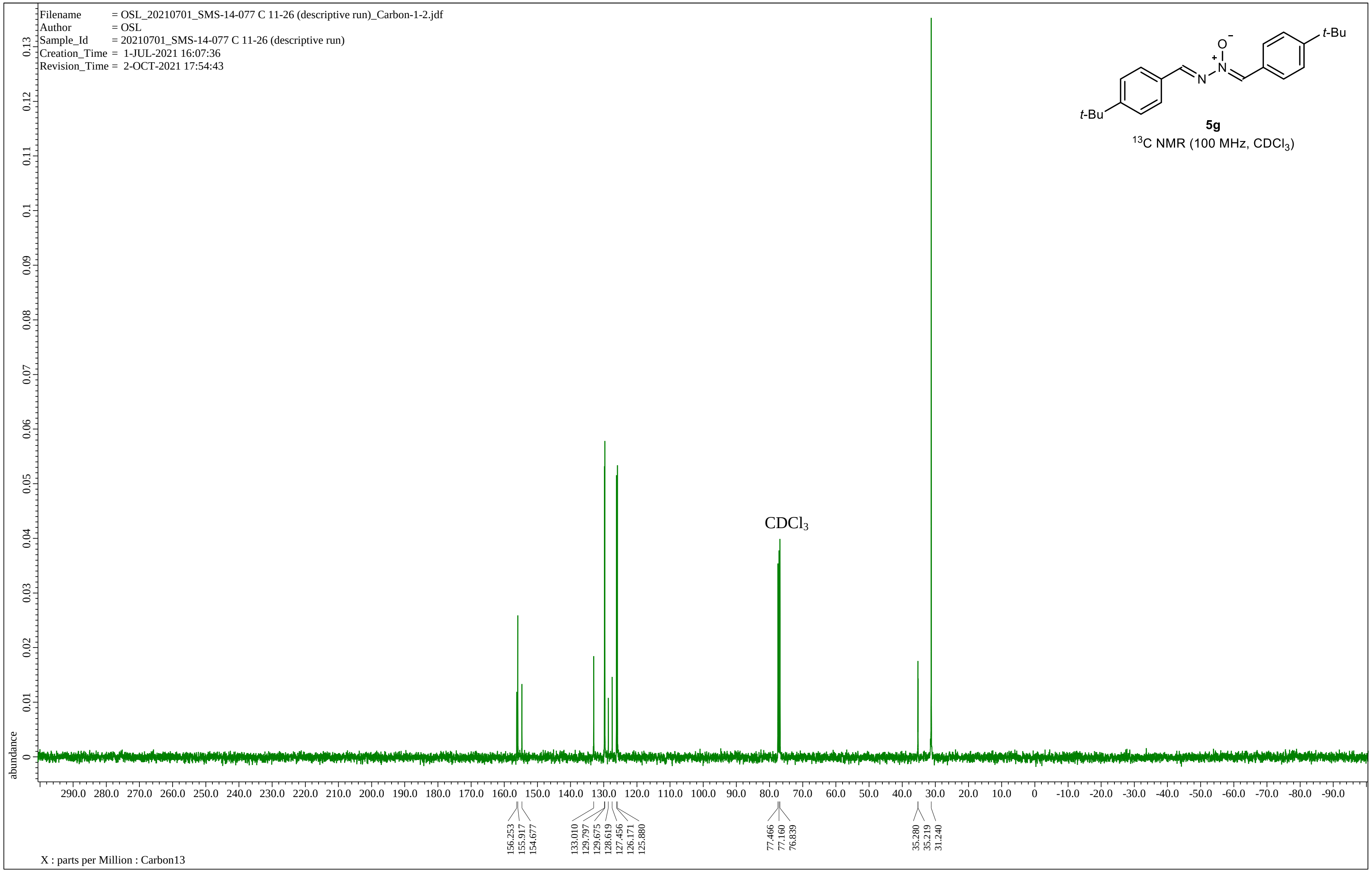

Supplementary Figure 51. ^{13}C NMR spectrum of compound **5g**, recorded at 100 MHz and 298 K in $CDCl_3$.

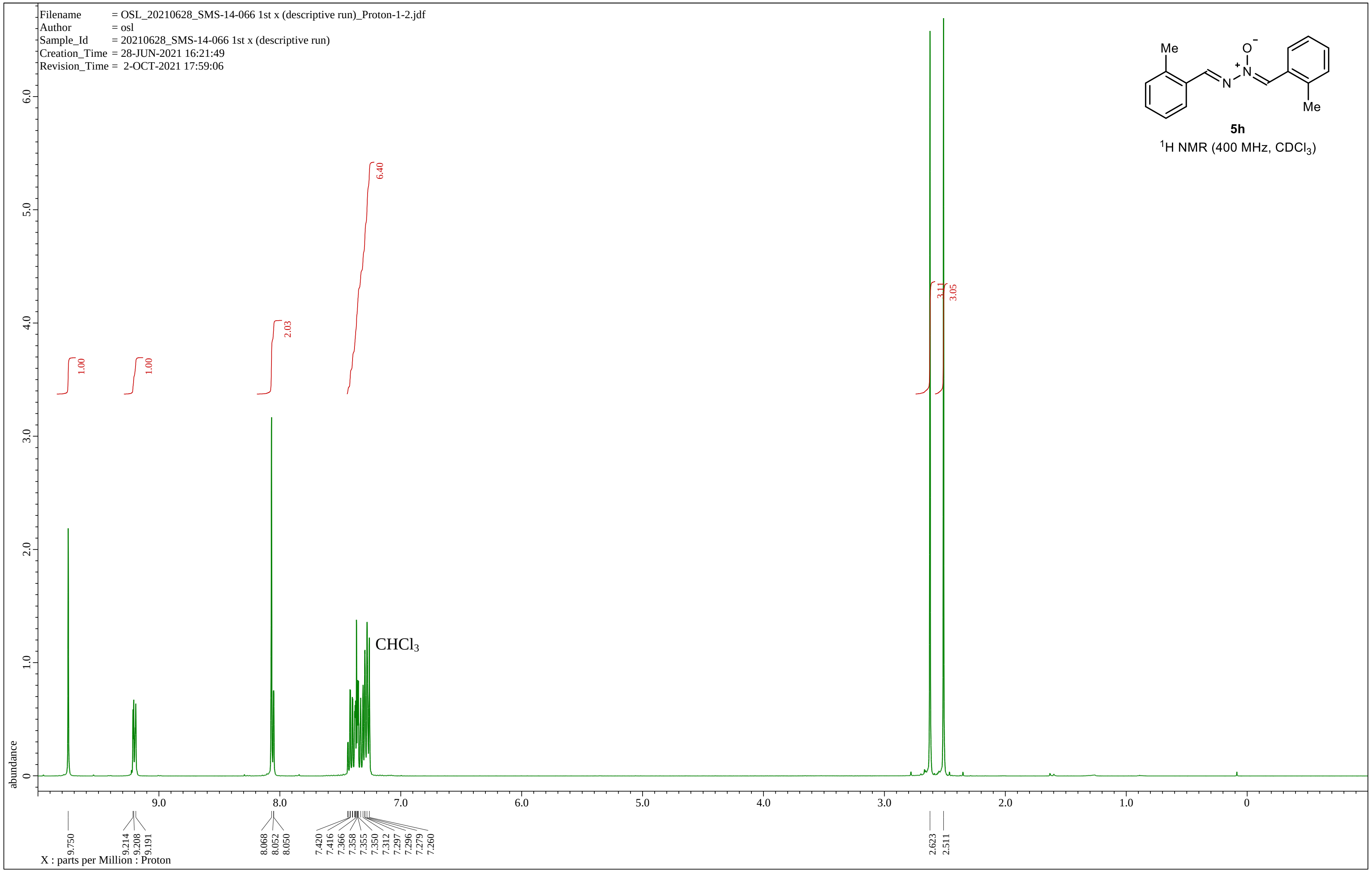

Supplementary Figure 52. ^{1}H NMR spectrum of compound **5h**, recorded at 400 MHz and 298 K in $CDCl_3$.

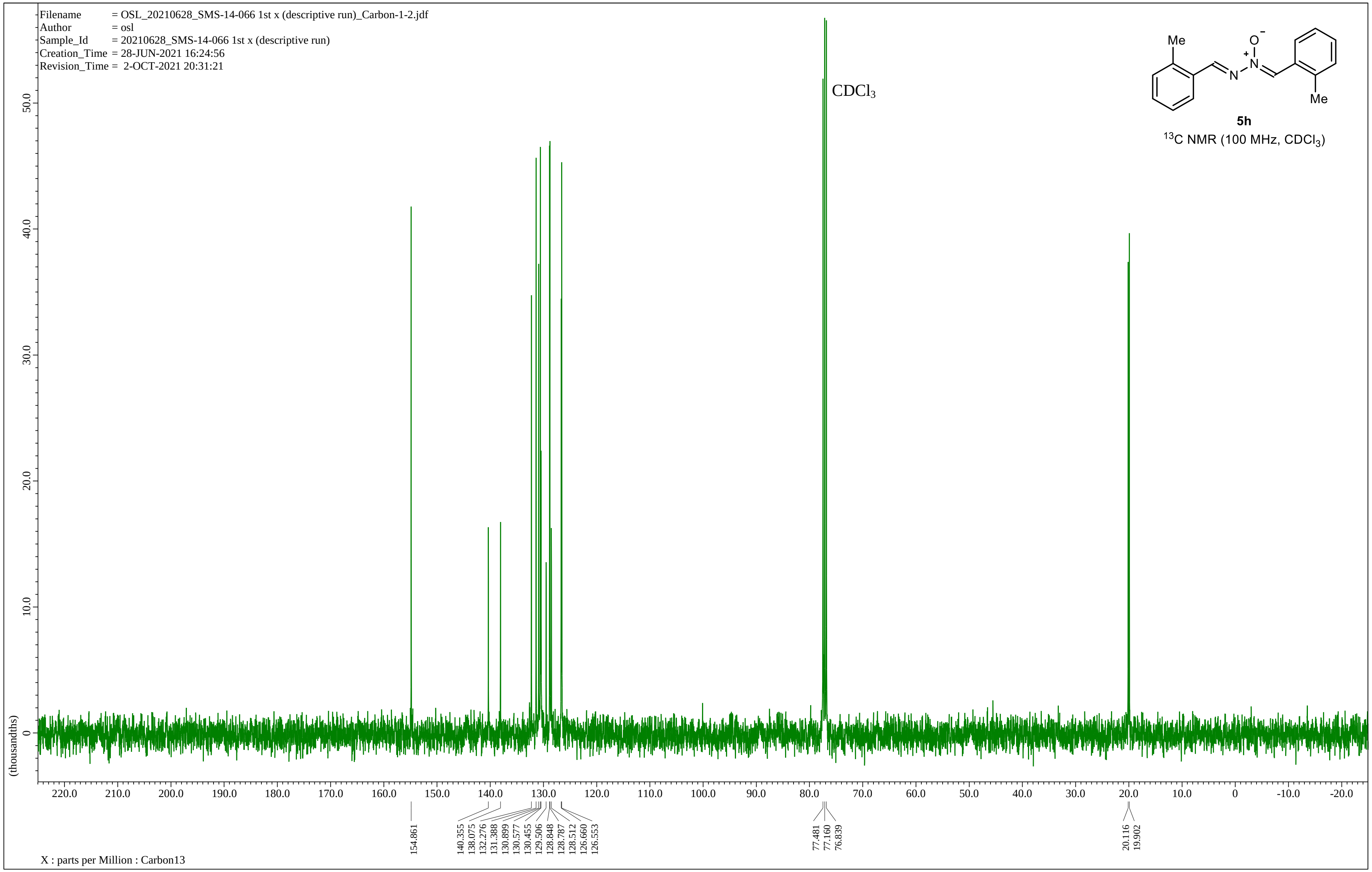

Supplementary Figure 53. ^{13}C NMR spectrum of compound **5h**, recorded at 100 MHz and 298 K in $CDCl_3$.

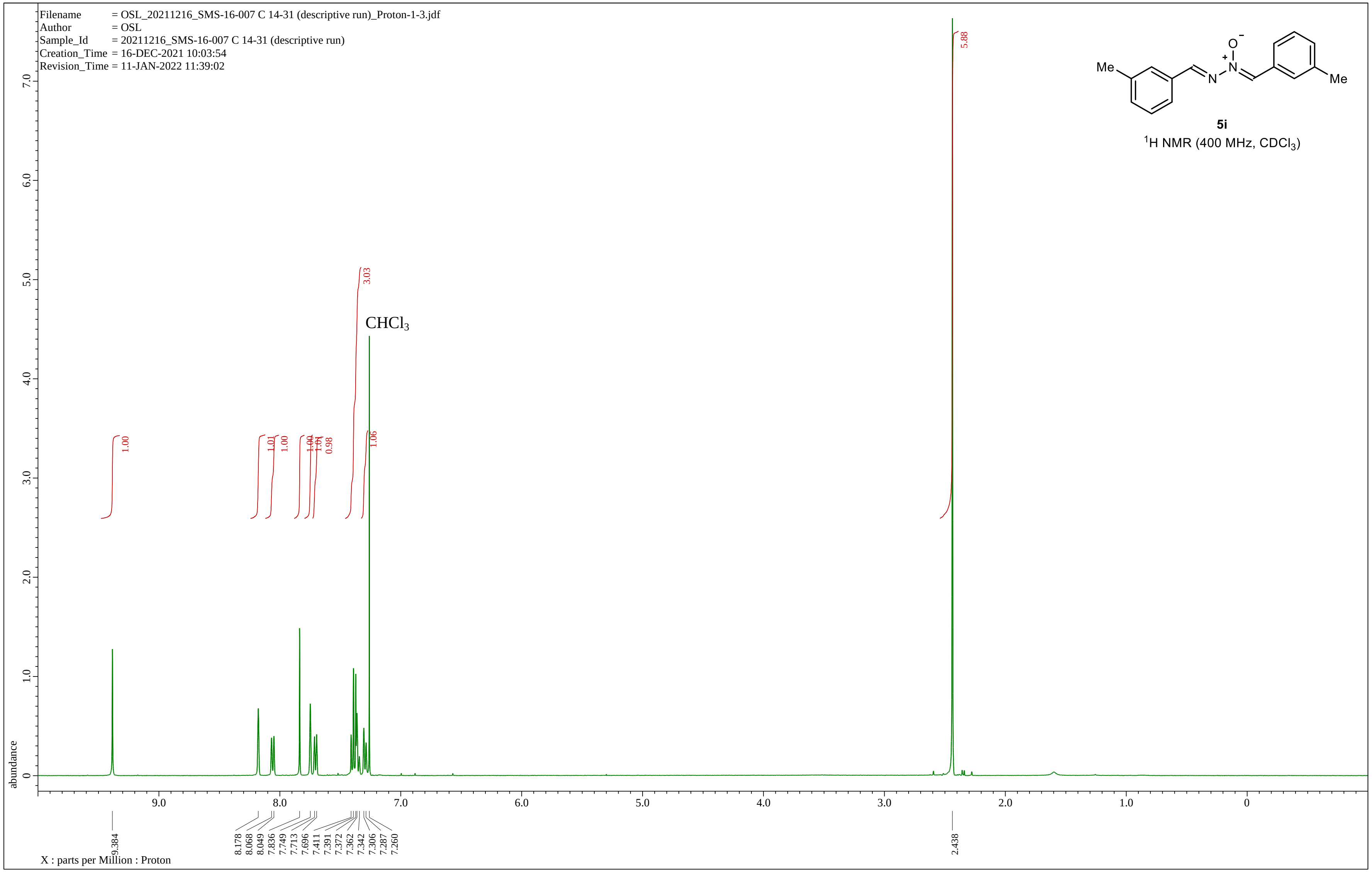

Supplementary Figure 54. ^{1}H NMR spectrum of compound **5i**, recorded at 400 MHz and 298 K in $CDCl_3$.

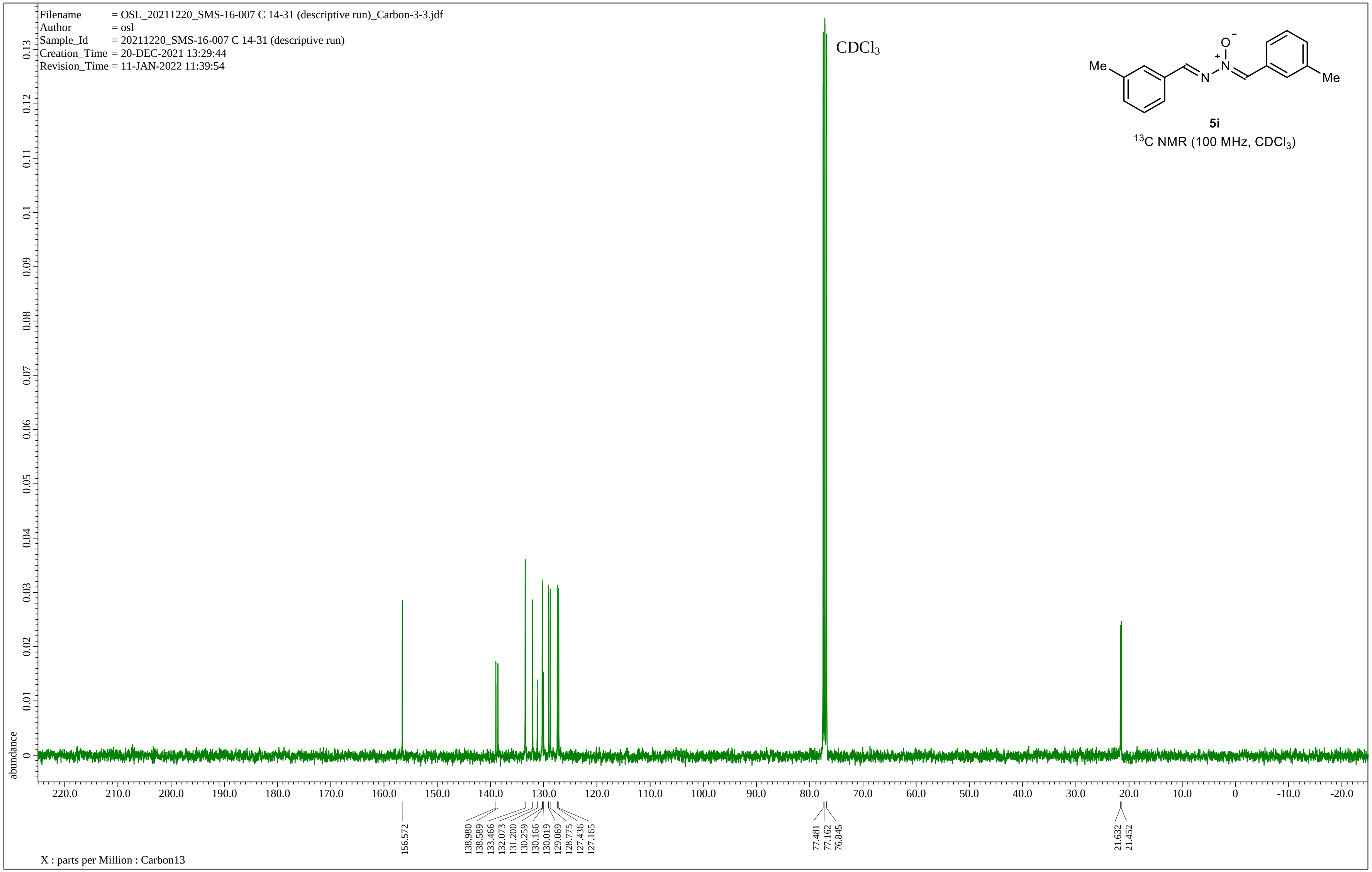

Supplementary Figure 55. ^{13}C NMR spectrum of compound **5i**, recorded at 100 MHz and 298 K in $CDCl_3$.

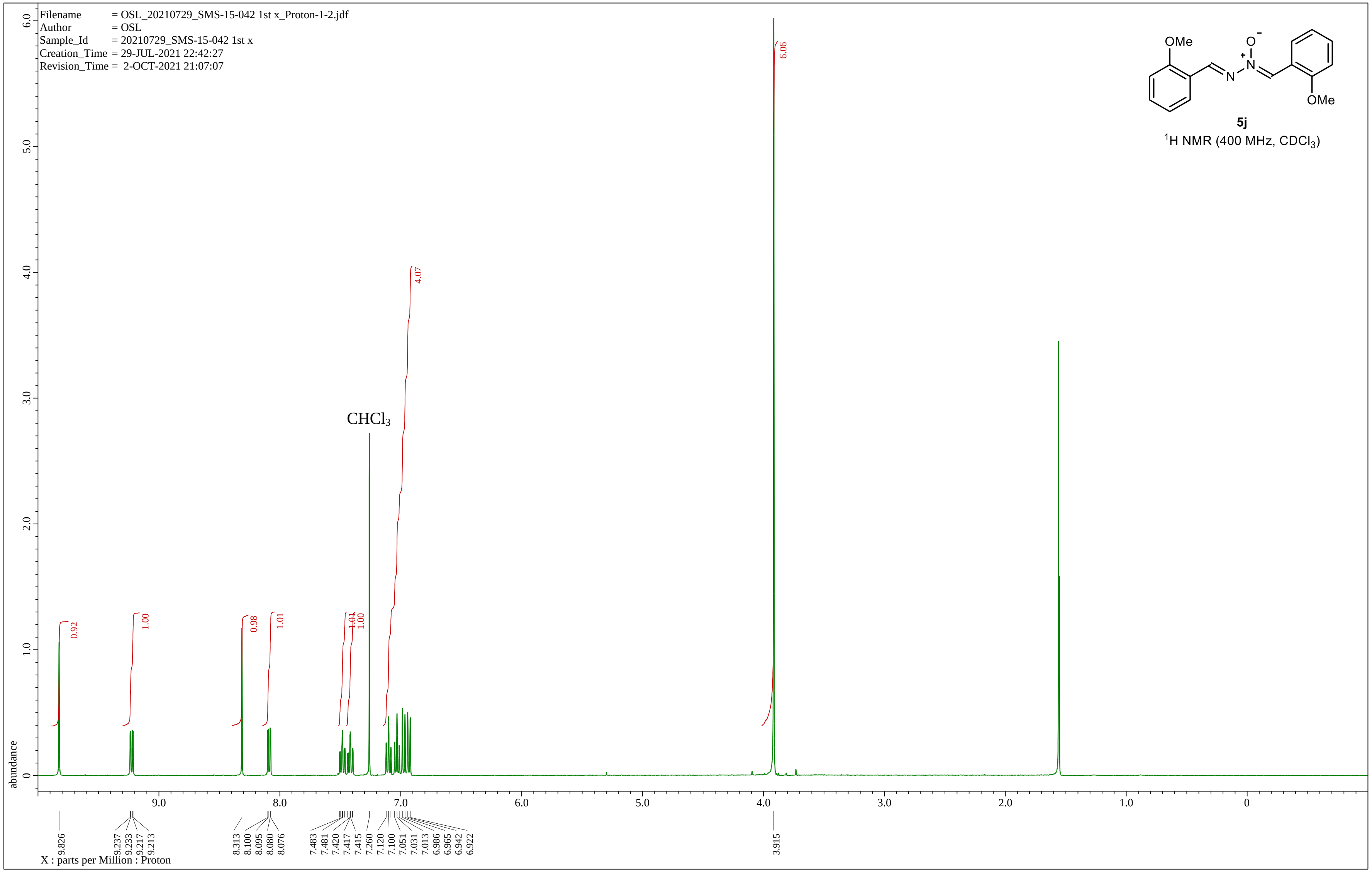

Supplementary Figure 56. ^{1}H NMR spectrum of compound **5j**, recorded at 400 MHz and 298 K in $CDCl_3$.

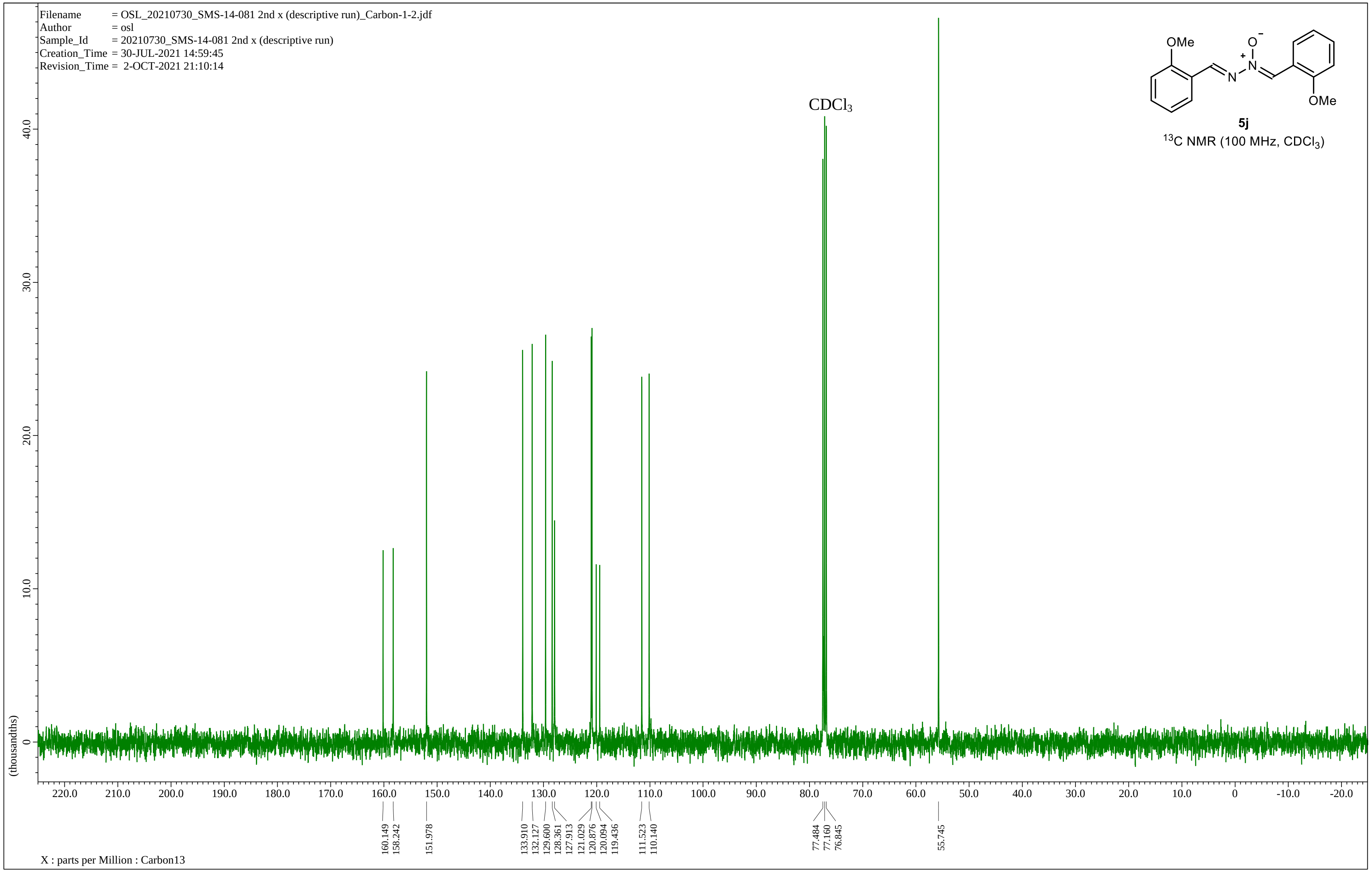

Supplementary Figure 57. ^{13}C NMR spectrum of compound **5j**, recorded at 100 MHz and 298 K in $CDCl_3$.

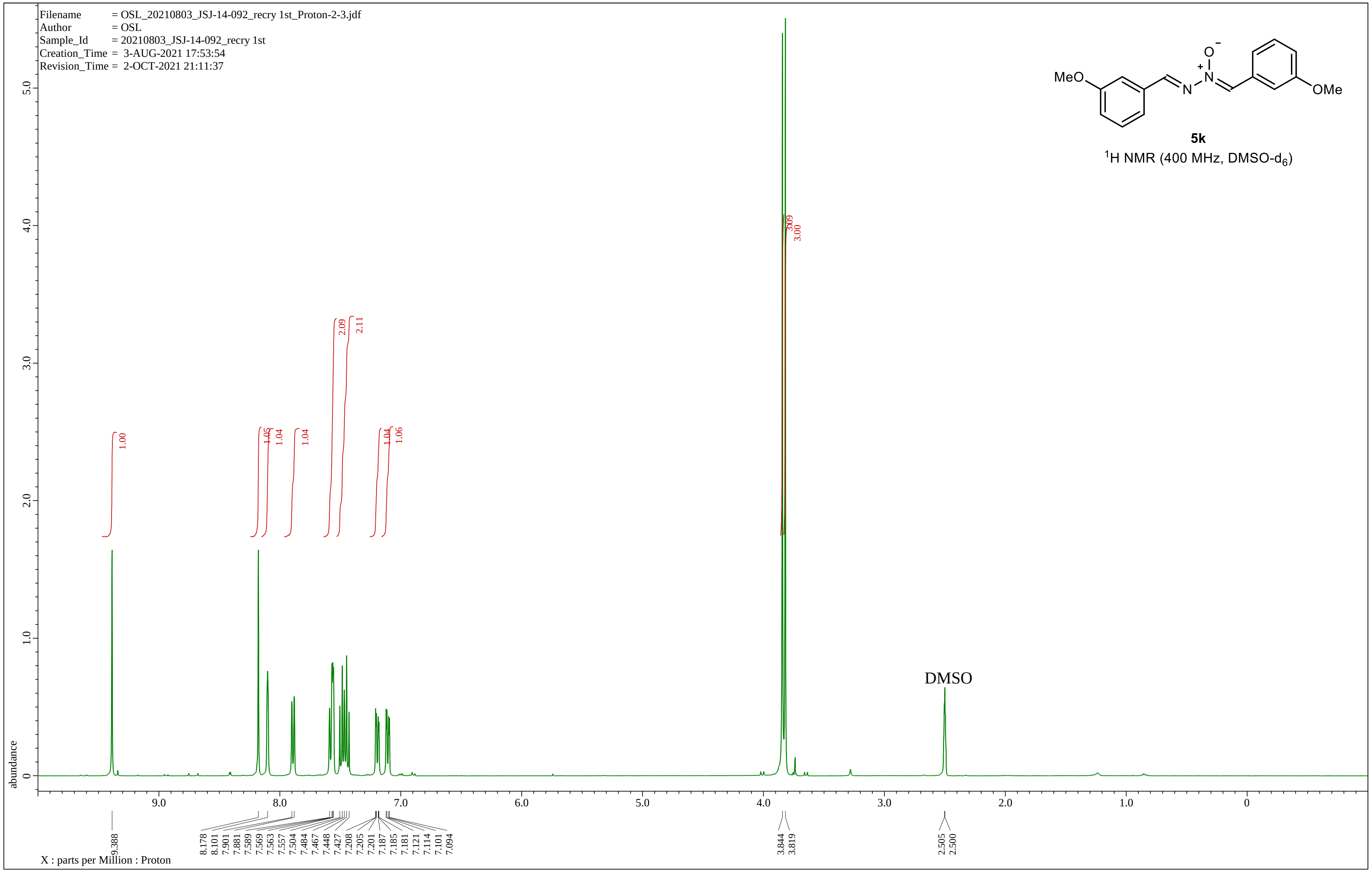

Supplementary Figure 58. ^{1}H NMR spectrum of compound **5k**, recorded at 400 MHz and 298 K in DMSO-d_6.

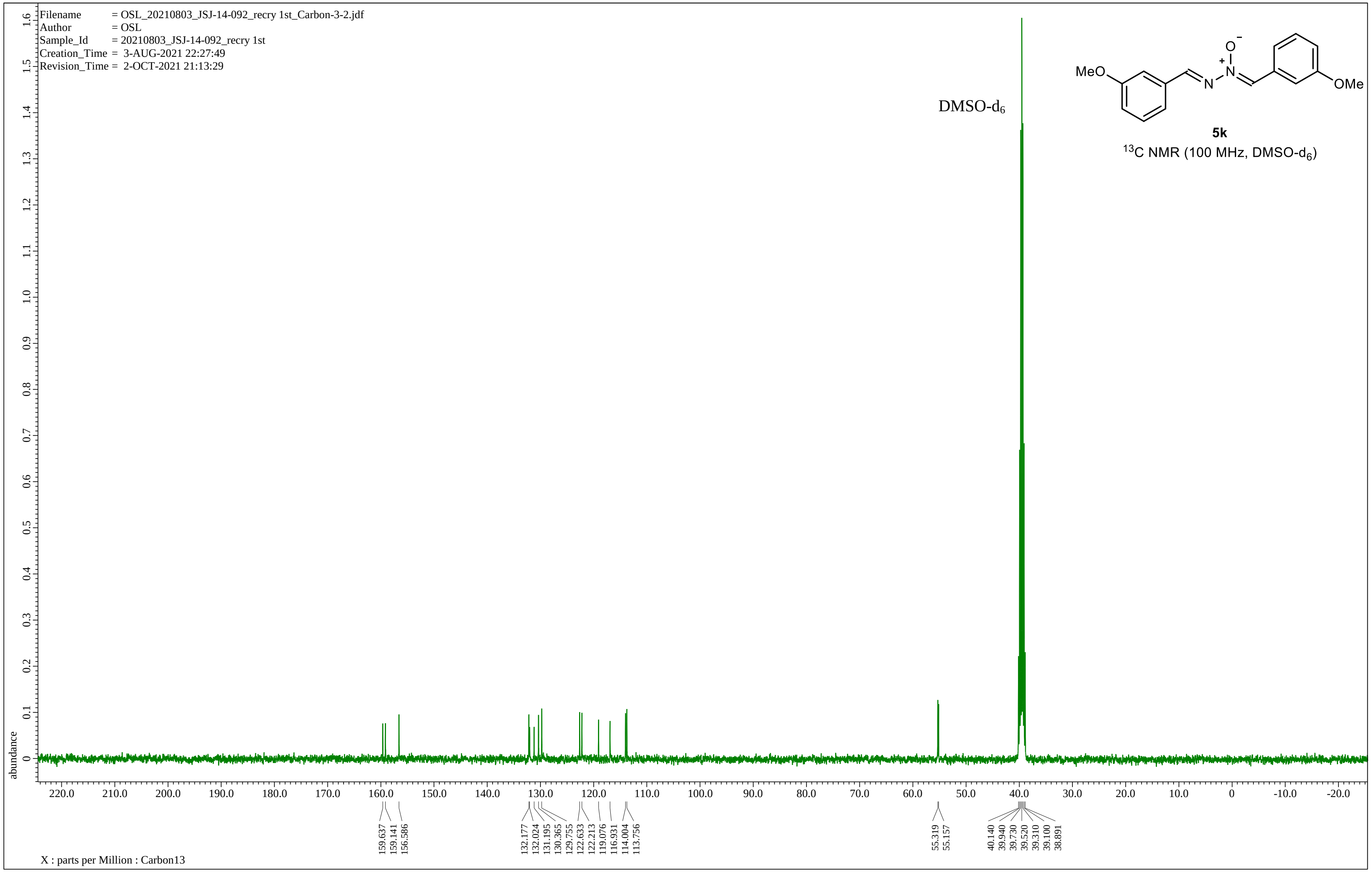

Supplementary Figure 59. ^{13}C NMR spectrum of compound **5k**, recorded at 100 MHz and 298 K in DMSO-d_6.

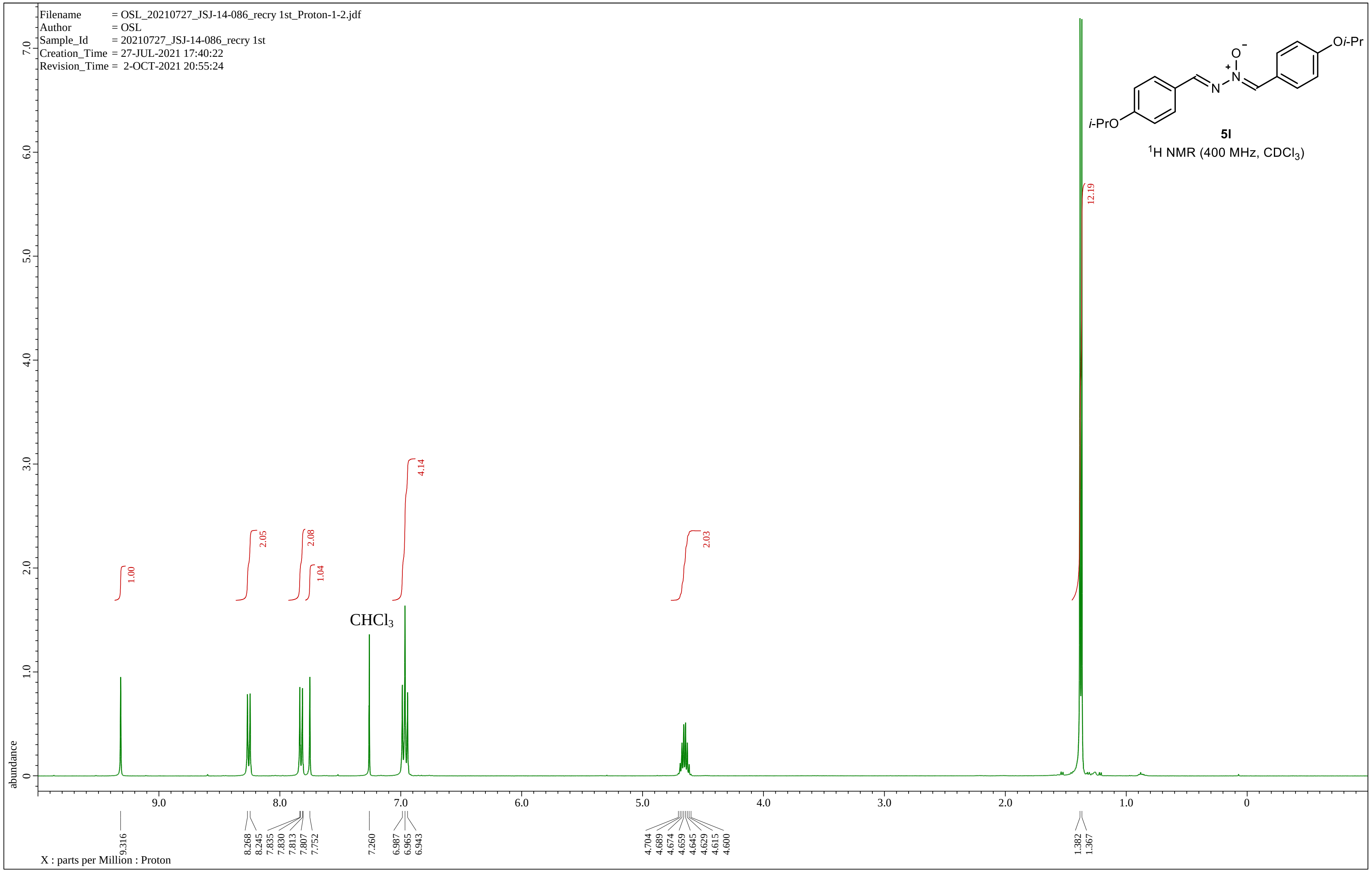

Supplementary Figure 60. ^{1}H NMR spectrum of compound **5l**, recorded at 400 MHz and 298 K in $CDCl_3$.

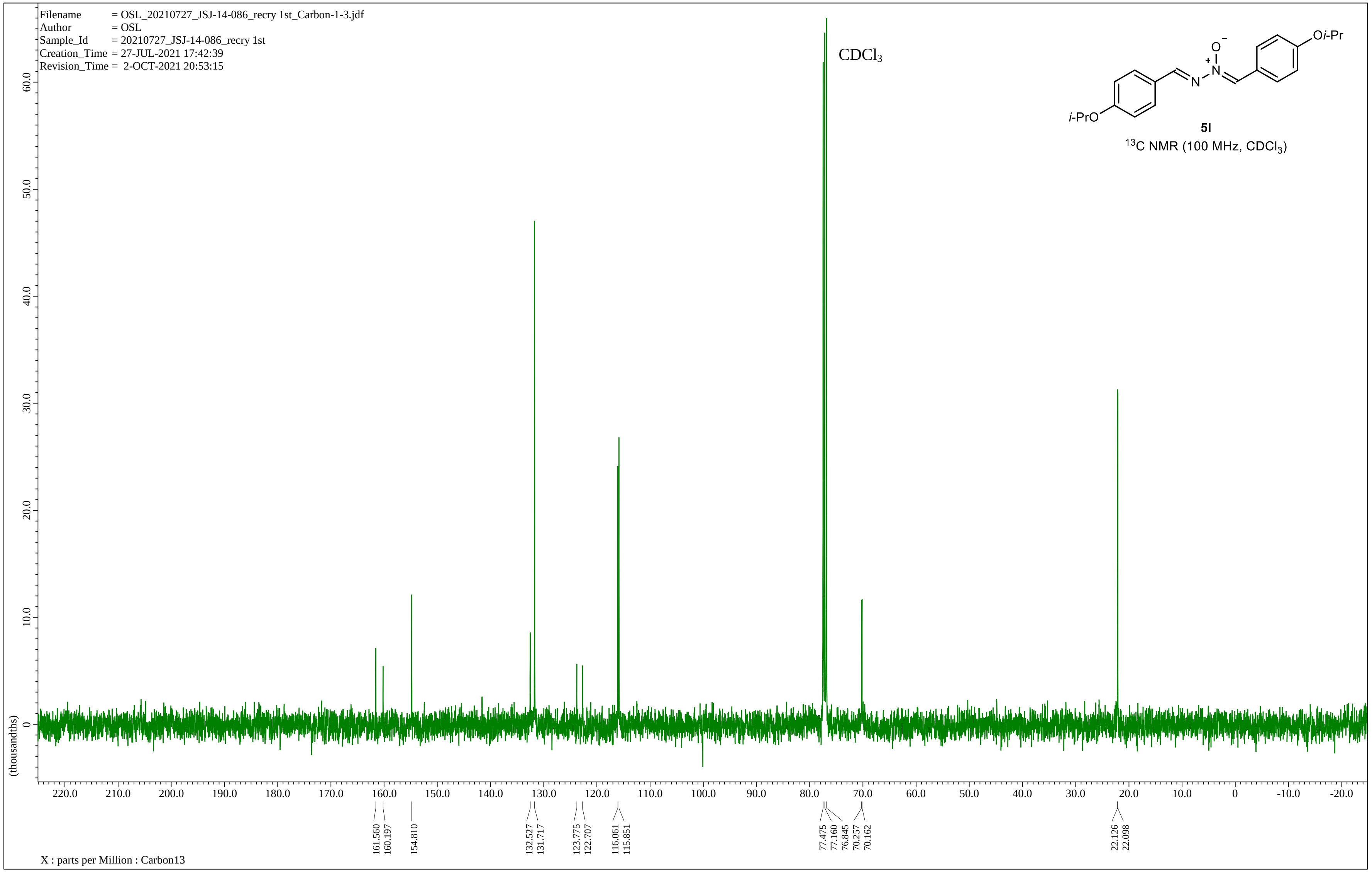

Supplementary Figure 61. ^{13}C NMR spectrum of compound **5l**, recorded at 100 MHz and 298 K in $CDCl_3$.

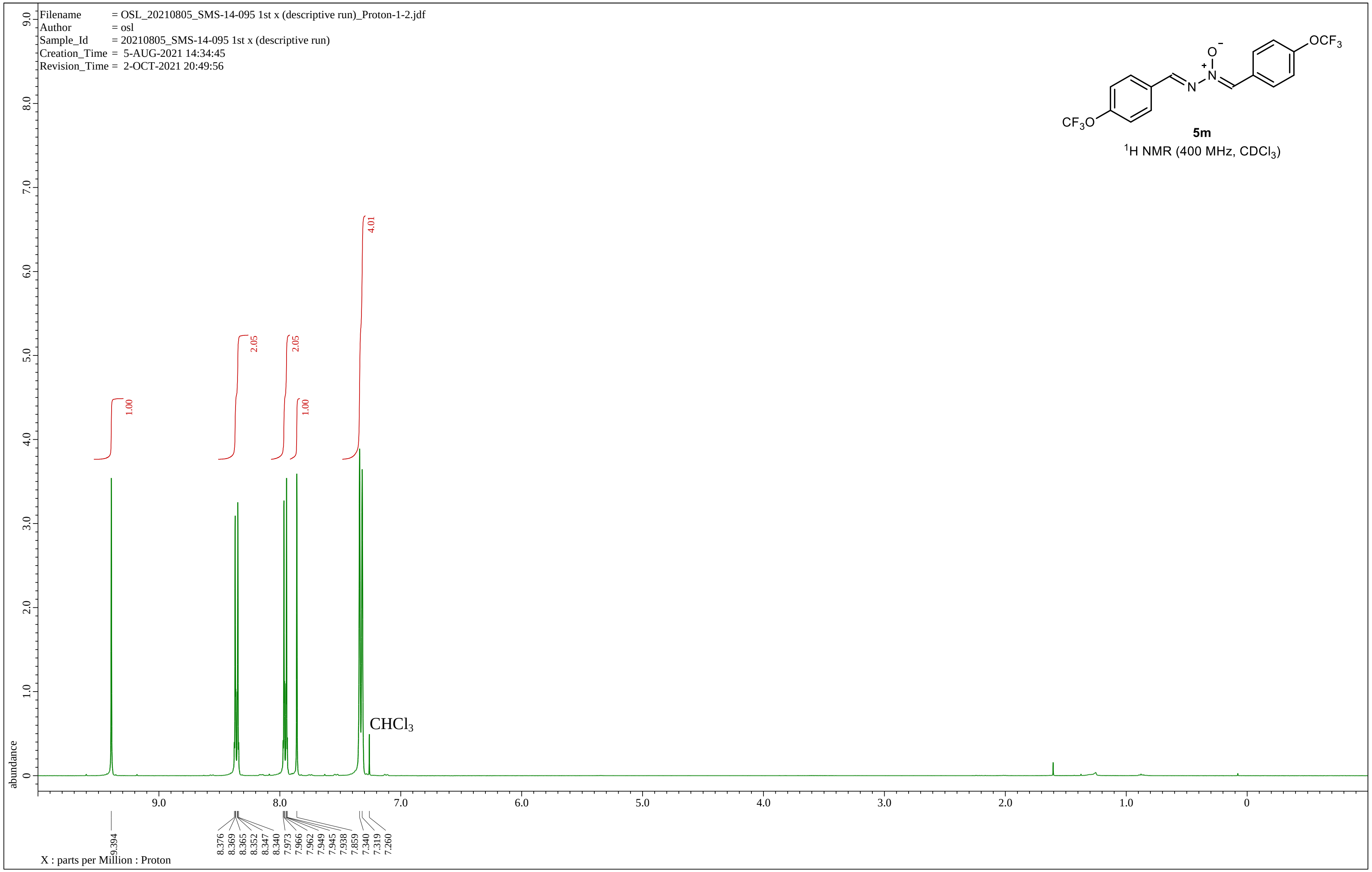

Supplementary Figure 62. ^{1}H NMR spectrum of compound **5m**, recorded at 400 MHz and 298 K in $CDCl_3$.

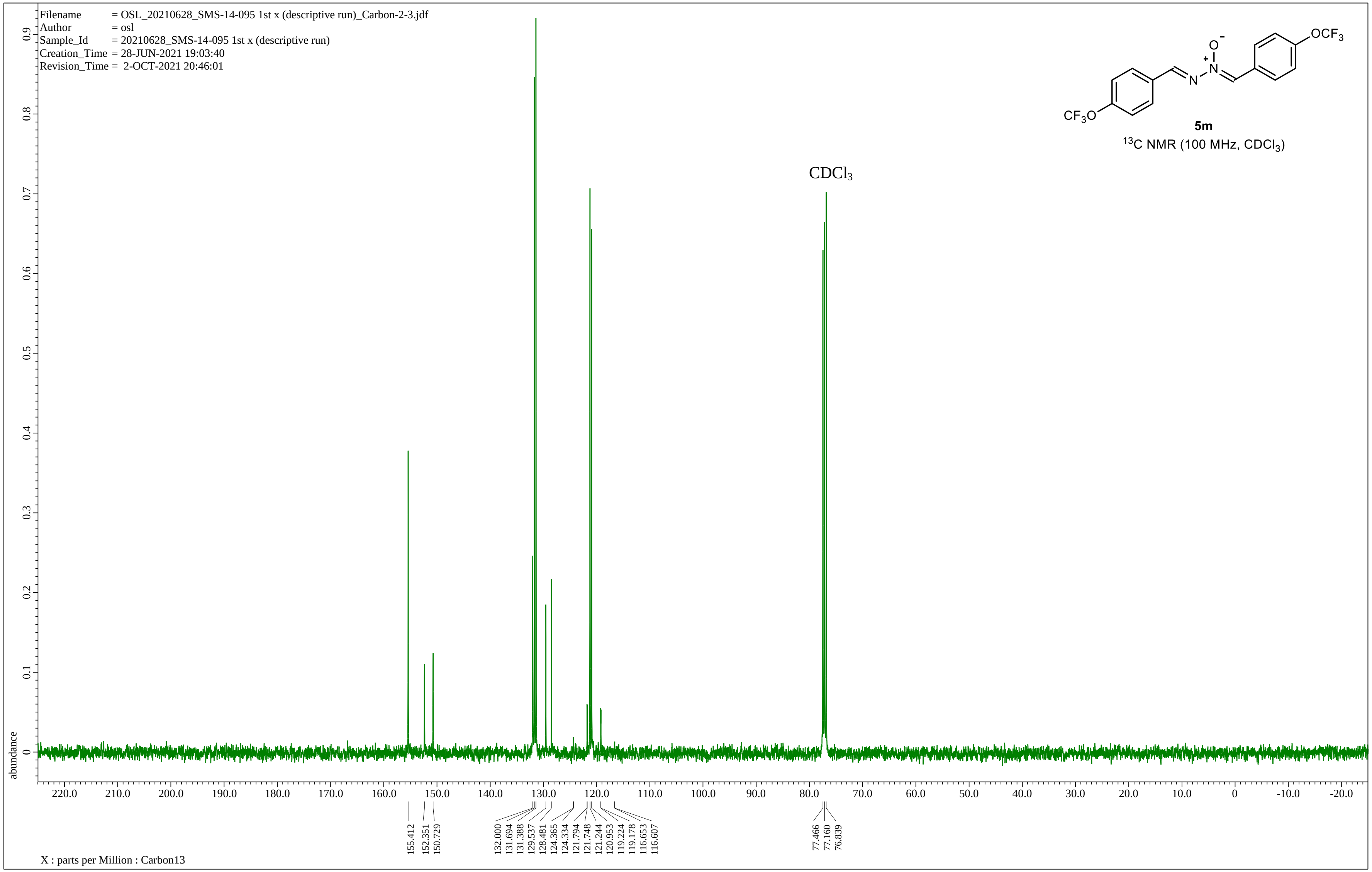

Supplementary Figure 63. ^{13}C NMR spectrum of compound **5m**, recorded at 100 MHz and 298 K in $CDCl_3$.

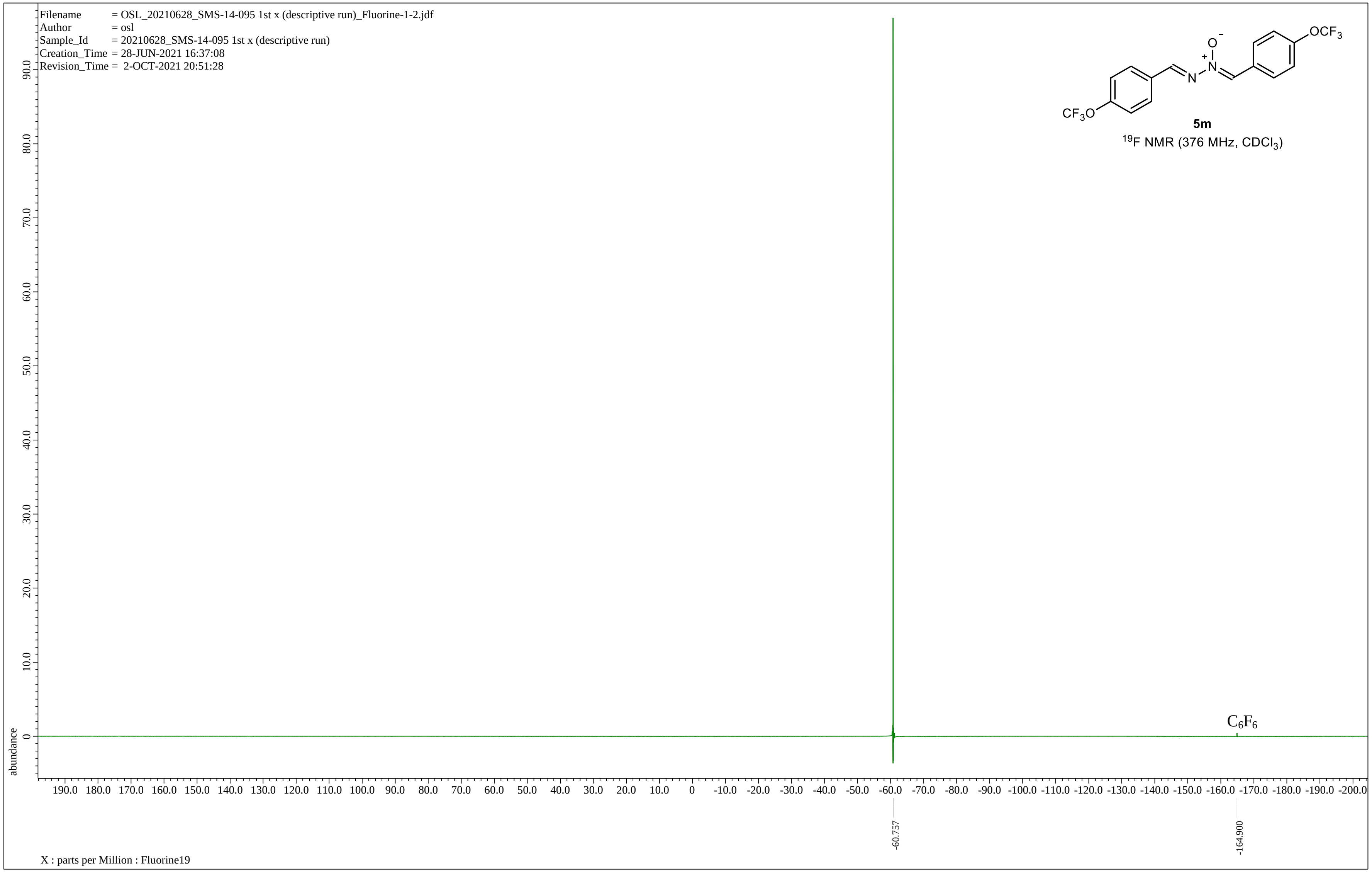

Supplementary Figure 64. ^{19}F NMR spectrum of compound **5m**, recorded at 376 MHz and 298 K in $CDCl_3$.

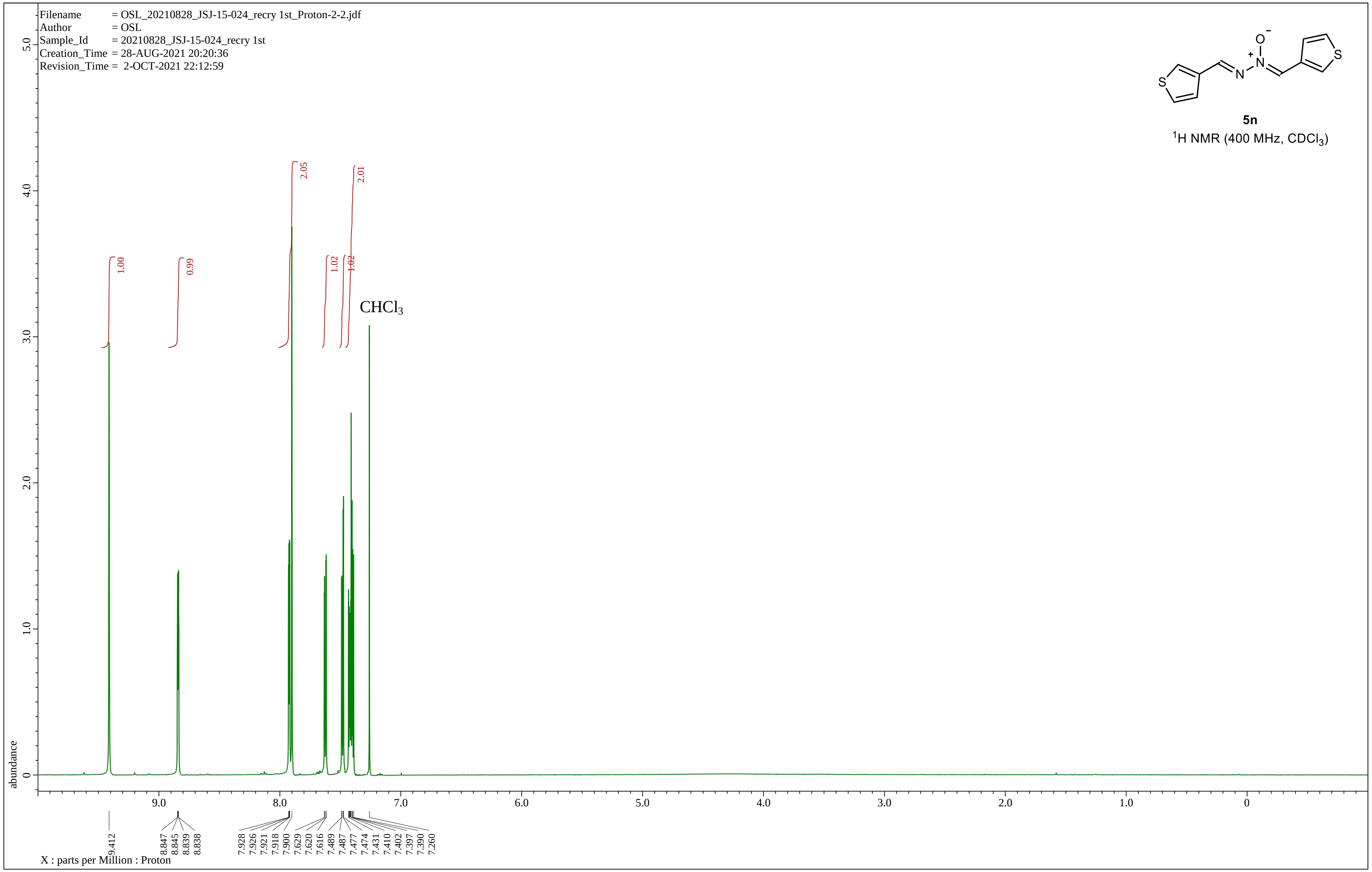

Supplementary Figure 65. ^{1}H NMR spectrum of compound **5n**, recorded at 400 MHz and 298 K in $CDCl_3$.

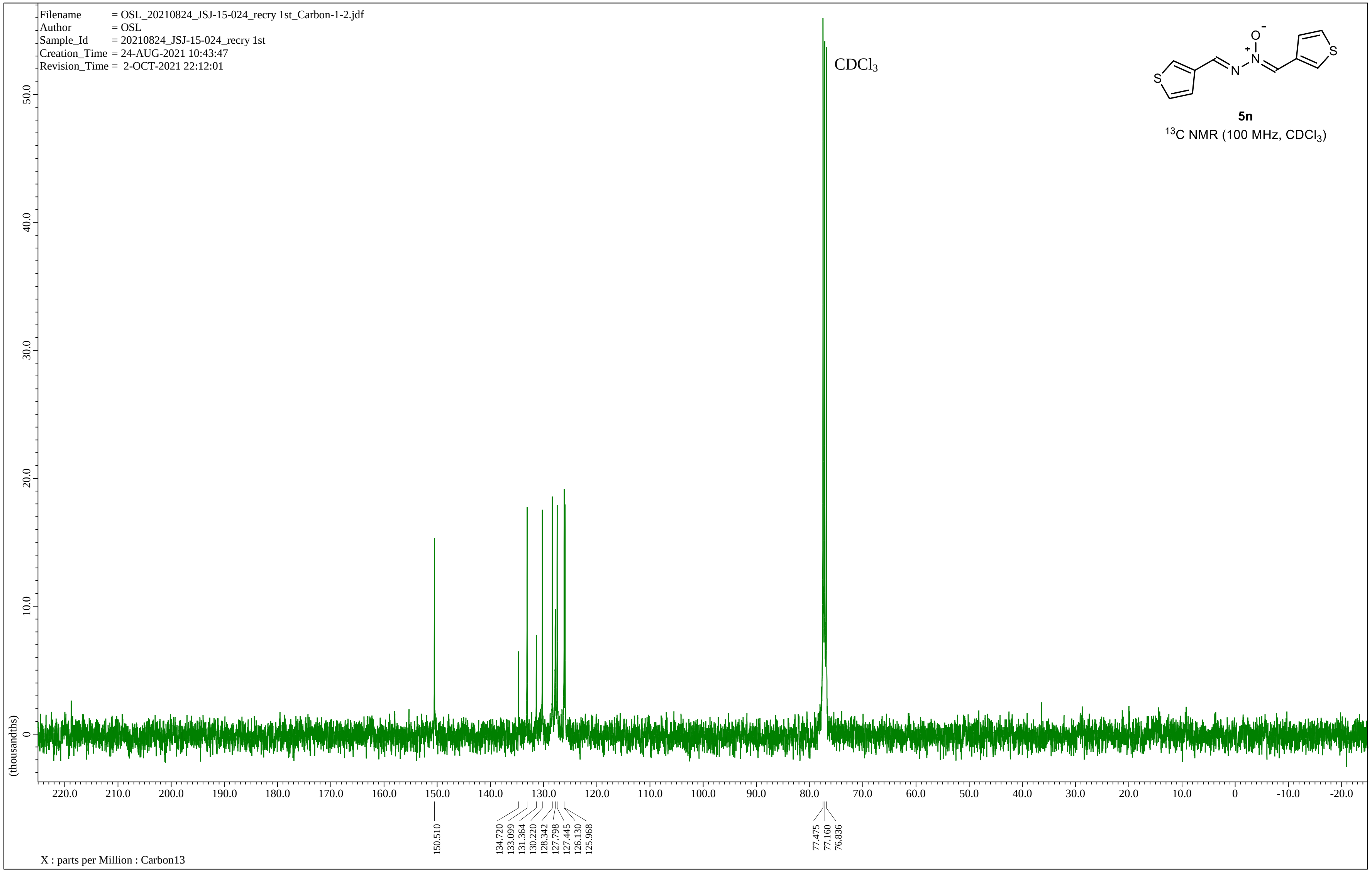

Supplementary Figure 66. ^{13}C NMR spectrum of compound **5n**, recorded at 100 MHz and 298 K in $CDCl_3$.

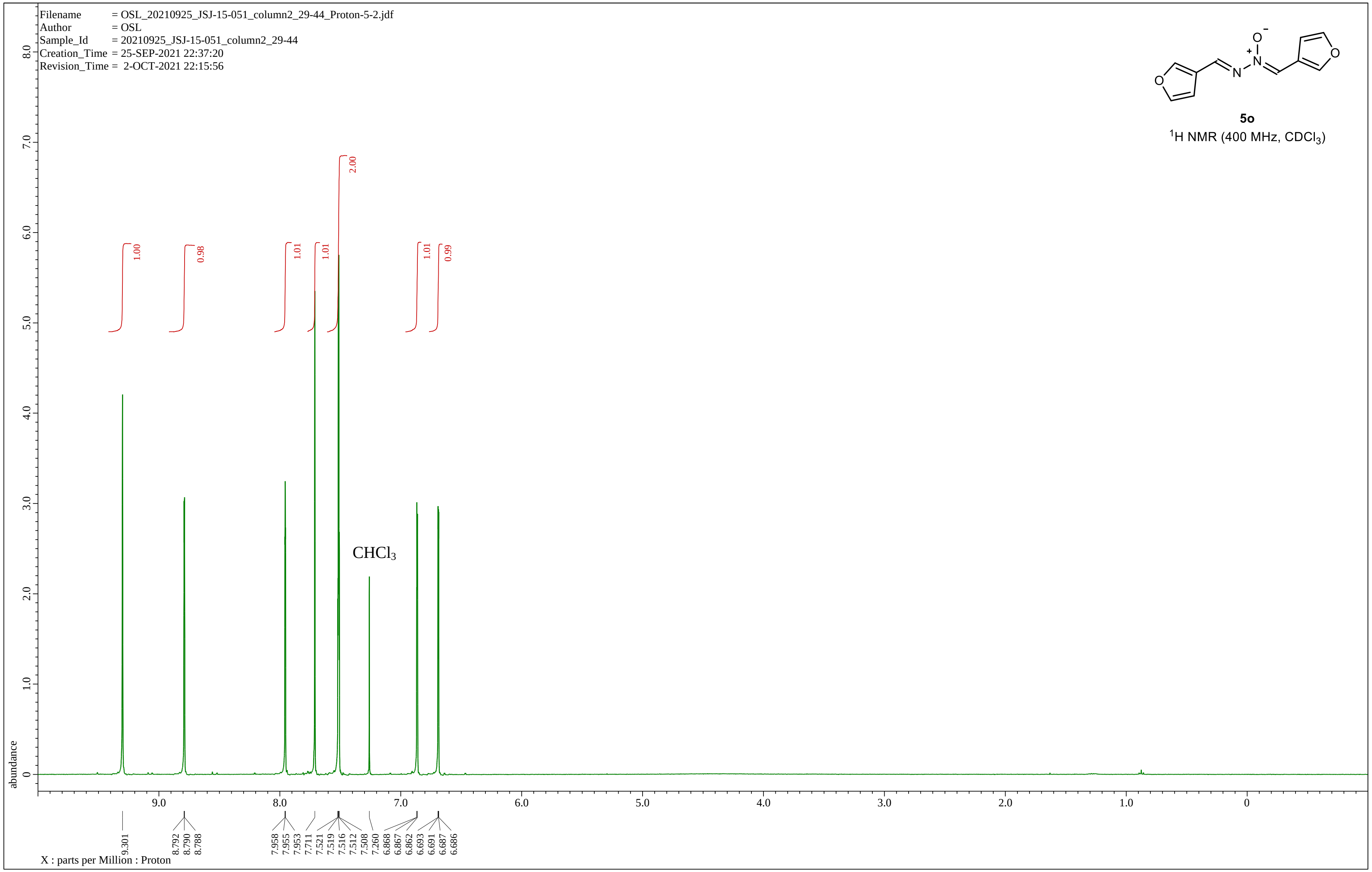

Supplementary Figure 67. ^{1}H NMR spectrum of compound **5o**, recorded at 400 MHz and 298 K in $CDCl_3$.

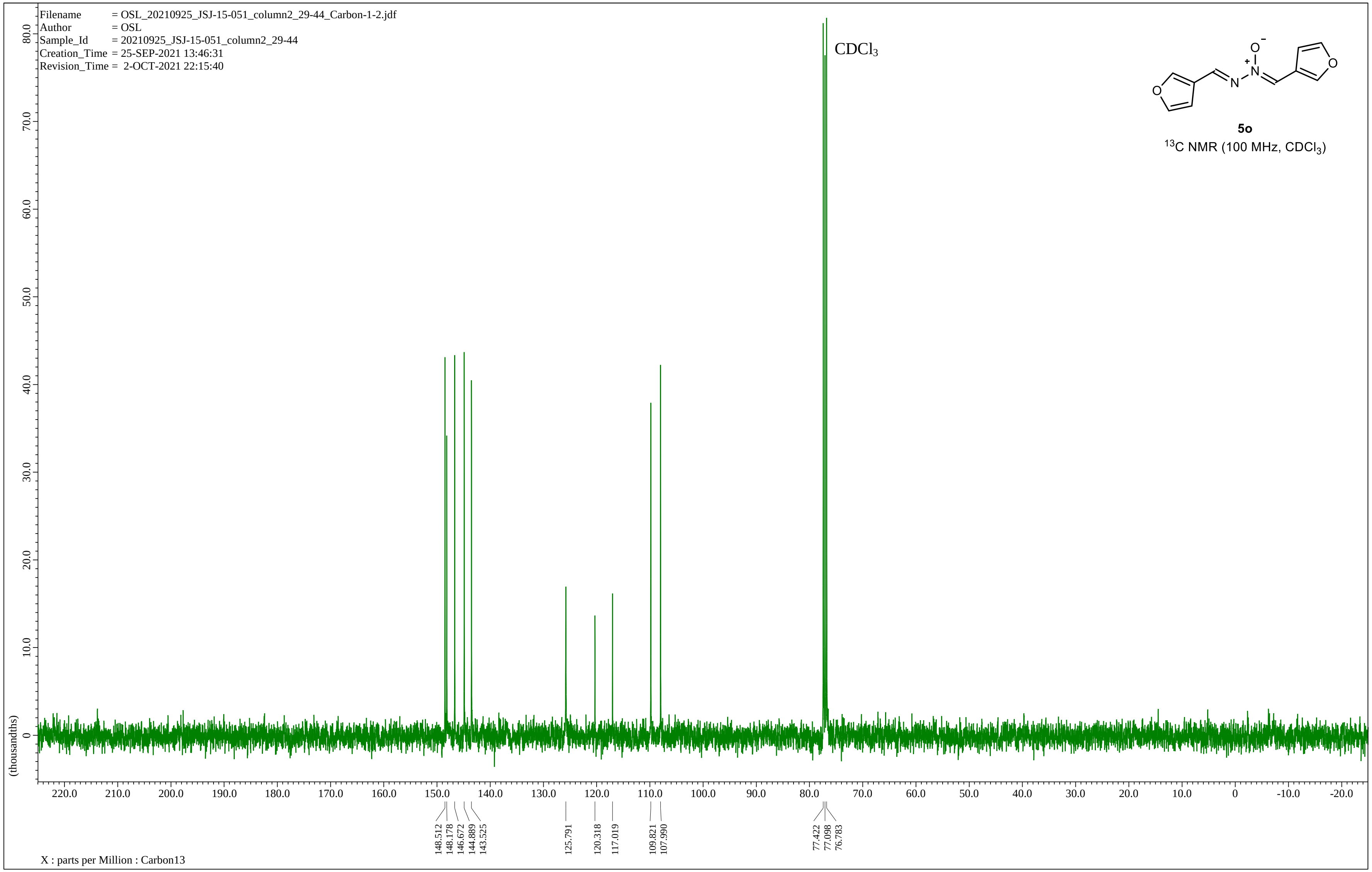

Supplementary Figure 68. ^{13}C NMR spectrum of compound **5o**, recorded at 100 MHz and 298 K in $CDCl_3$.

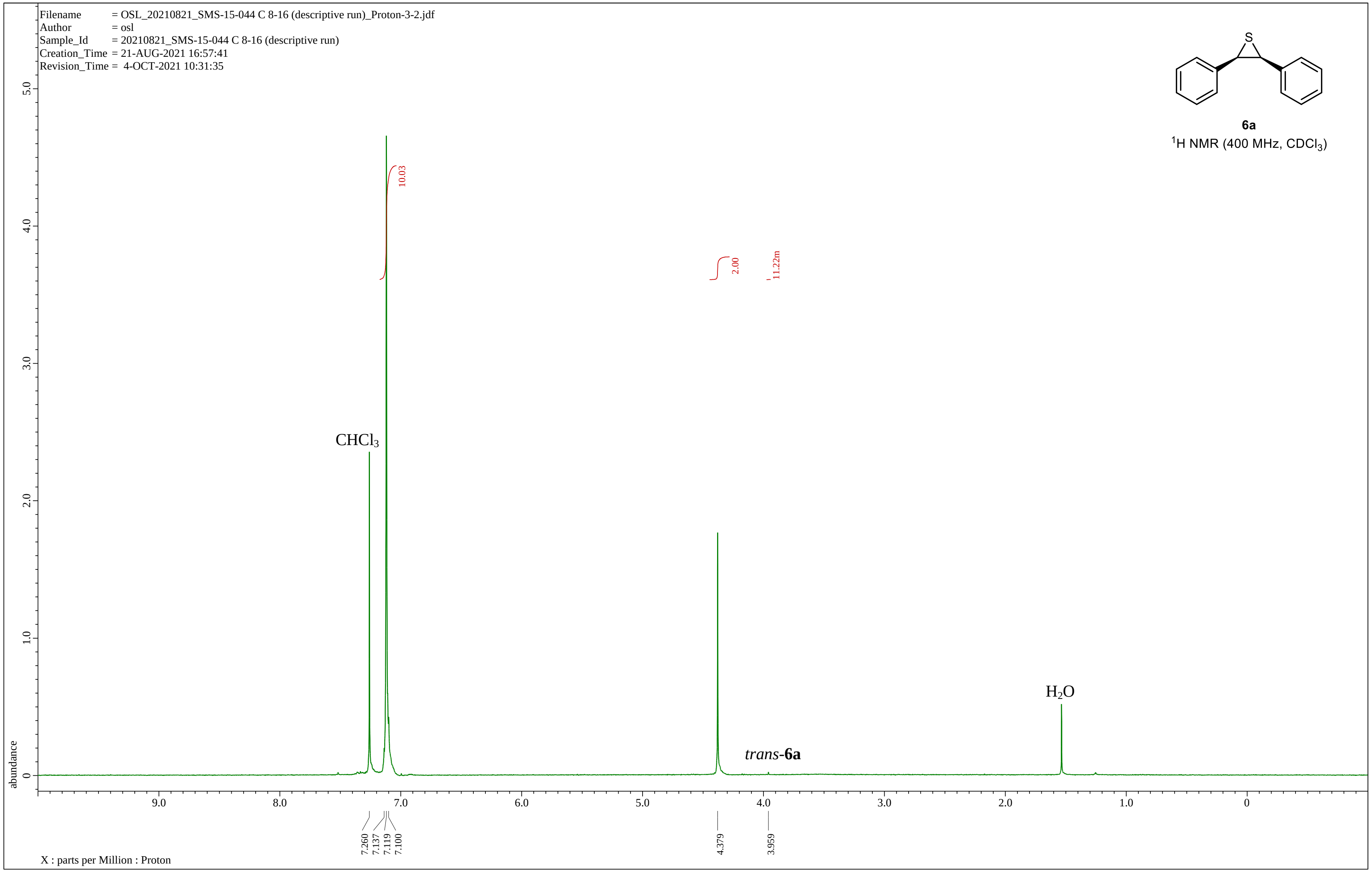

Supplementary Figure 69. ^{1}H NMR spectrum of compound **6a**, recorded at 400 MHz and 298 K in $CDCl_3$.

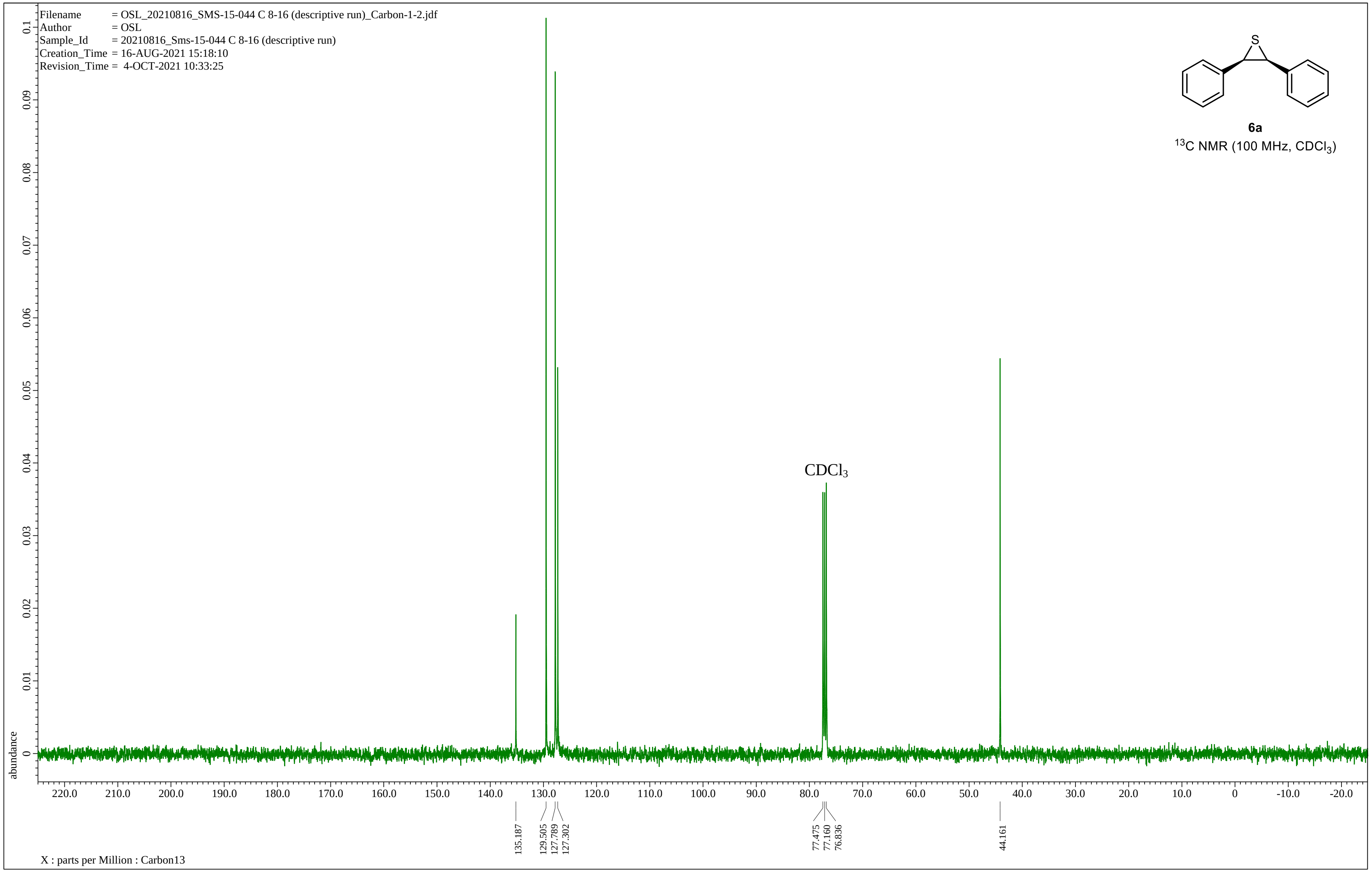

Supplementary Figure 70. ^{13}C NMR spectrum of compound **6a**, recorded at 100 MHz and 298 K in $CDCl_3$.

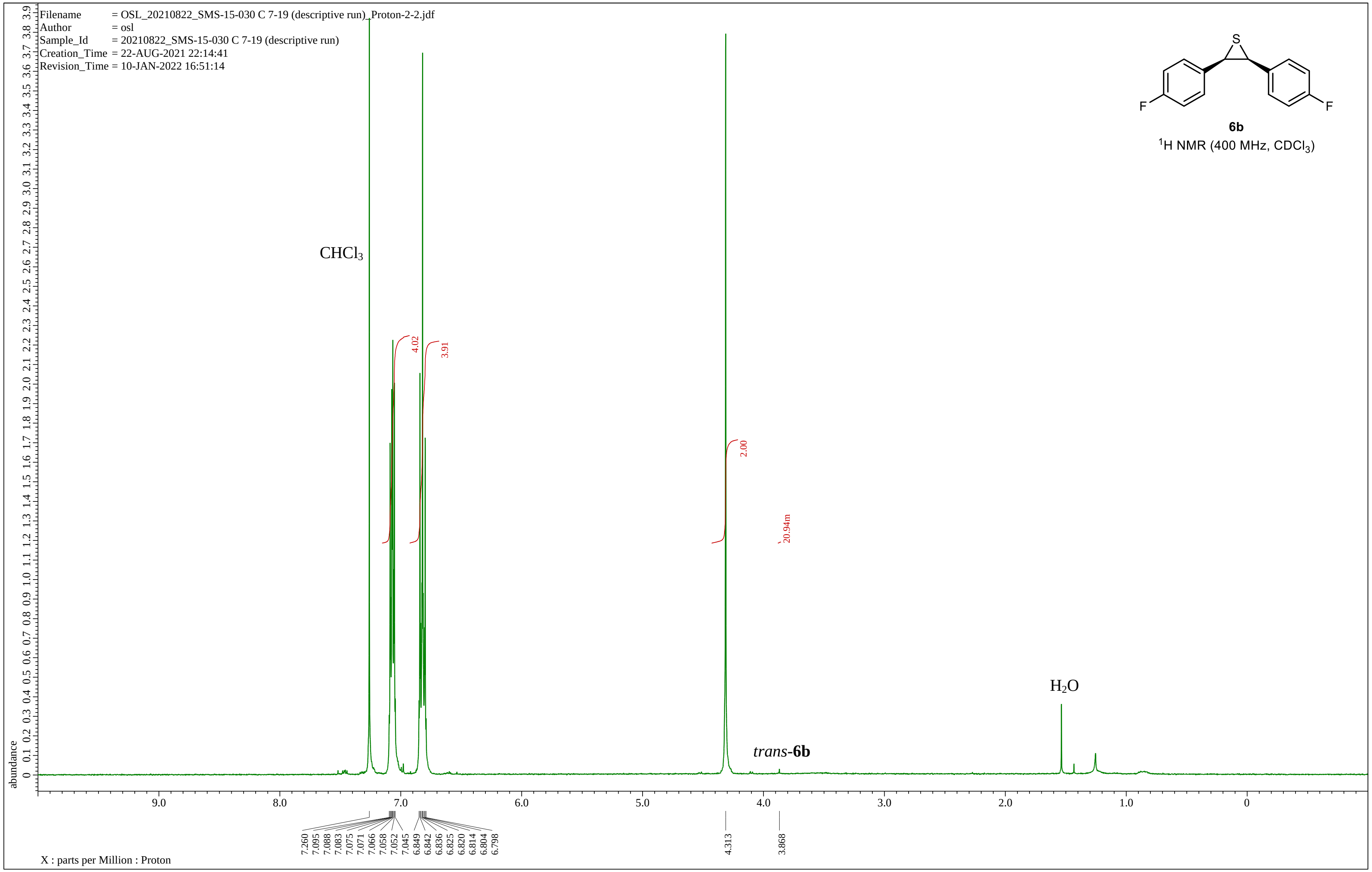

Supplementary Figure 71. ^{1}H NMR spectrum of compound **6b**, recorded at 400 MHz and 298 K in $CDCl_3$.

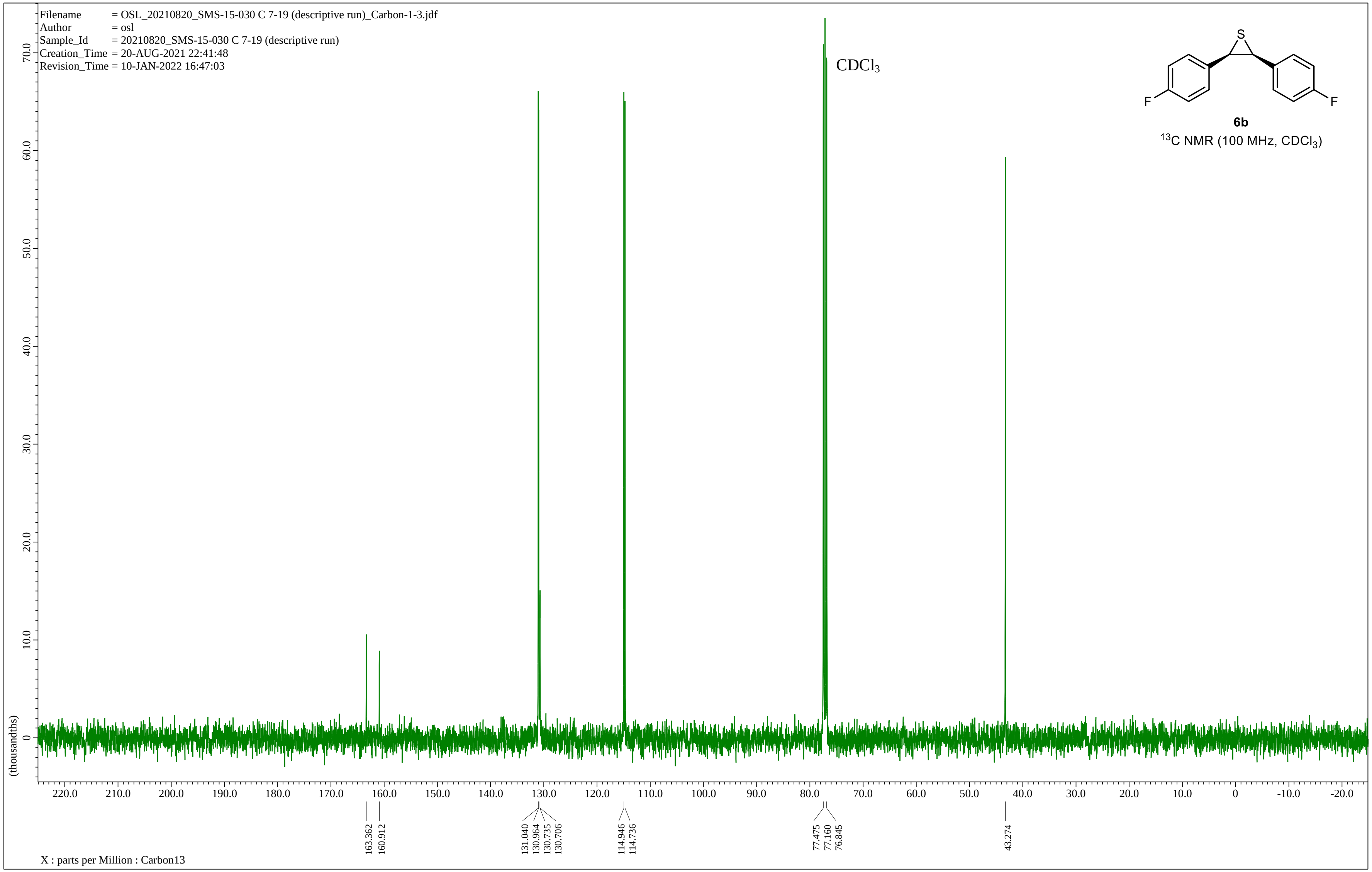

Supplementary Figure 72. ^{13}C NMR spectrum of compound **6b**, recorded at 100 MHz and 298 K in $CDCl_3$.

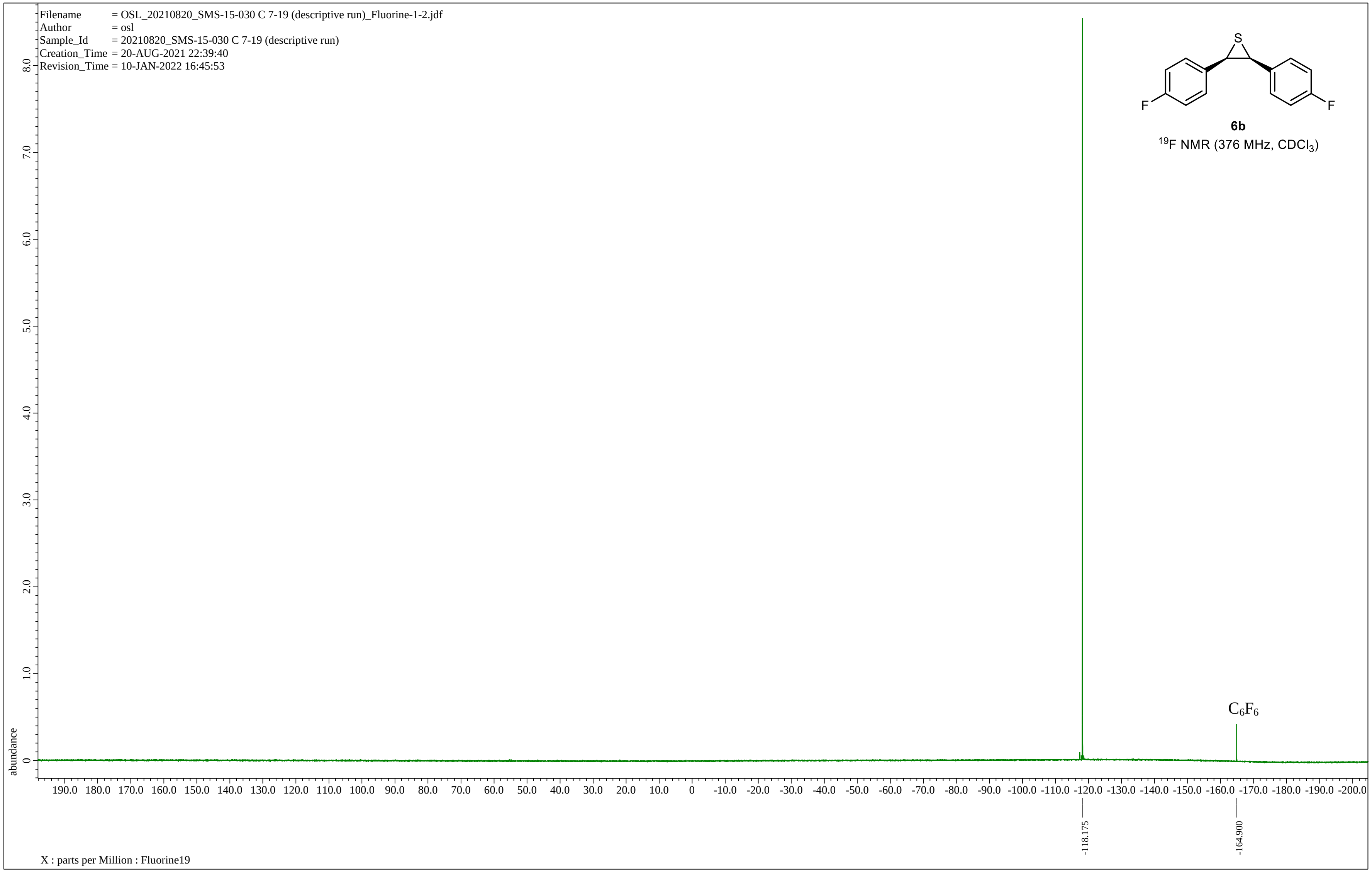

Supplementary Figure 73. ^{19}F NMR spectrum of compound **6b**, recorded at 376 MHz and 298 K in $CDCl_3$.

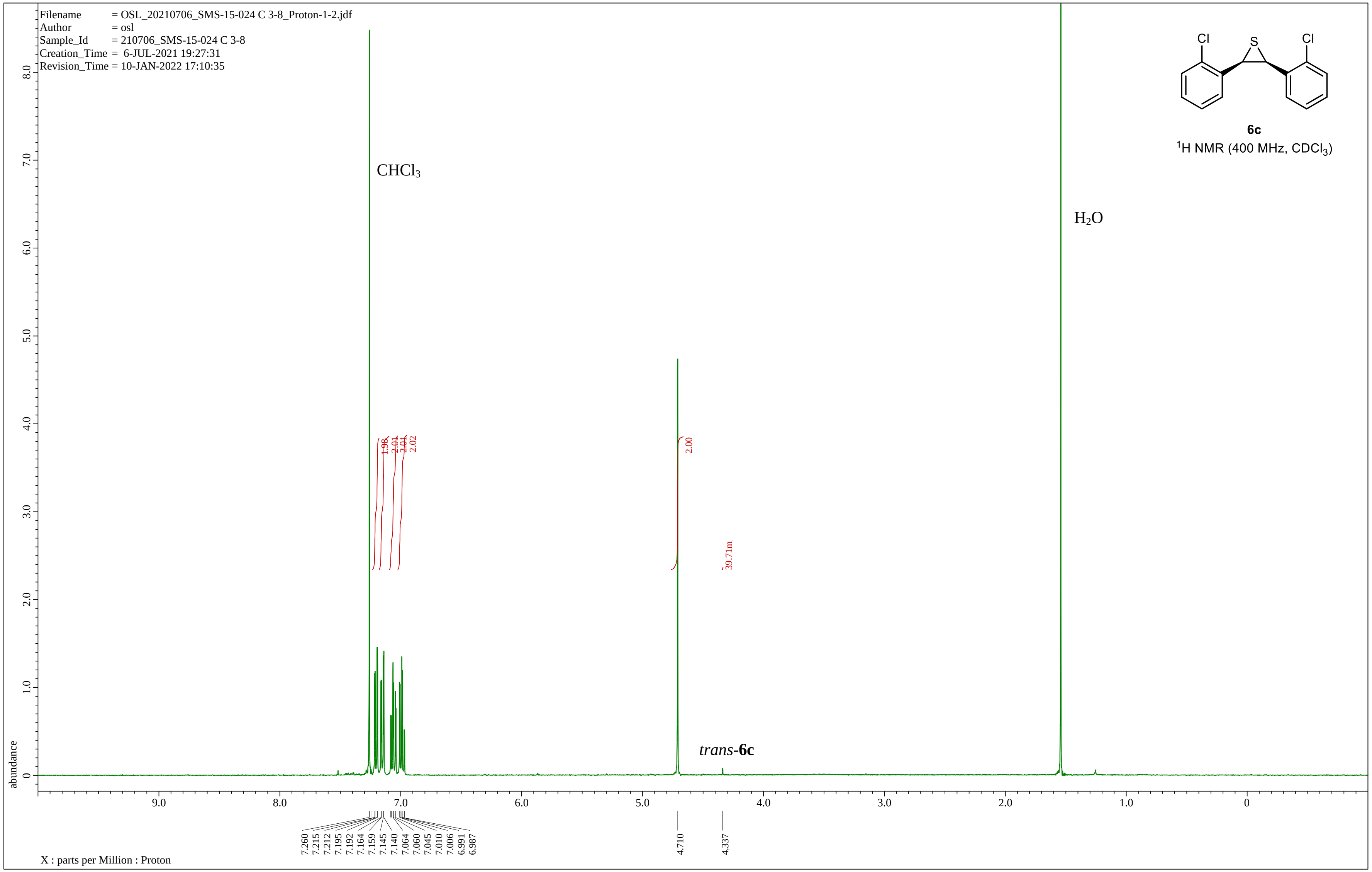

Supplementary Figure 74. ^{1}H NMR spectrum of compound **6c**, recorded at 400 MHz and 298 K in $CDCl_3$.

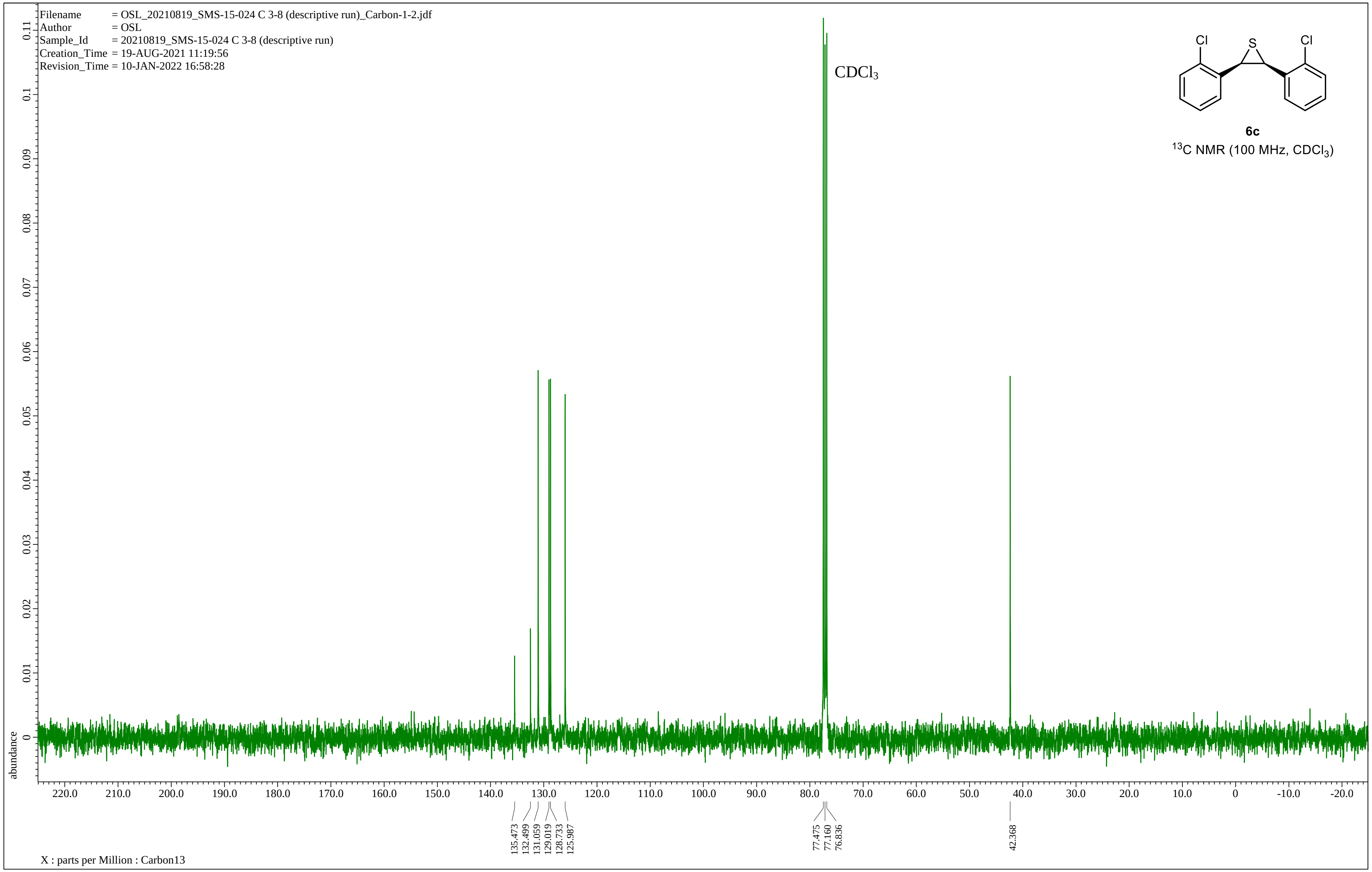

Supplementary Figure 75. ^{13}C NMR spectrum of compound **6c**, recorded at 100 MHz and 298 K in $CDCl_3$.

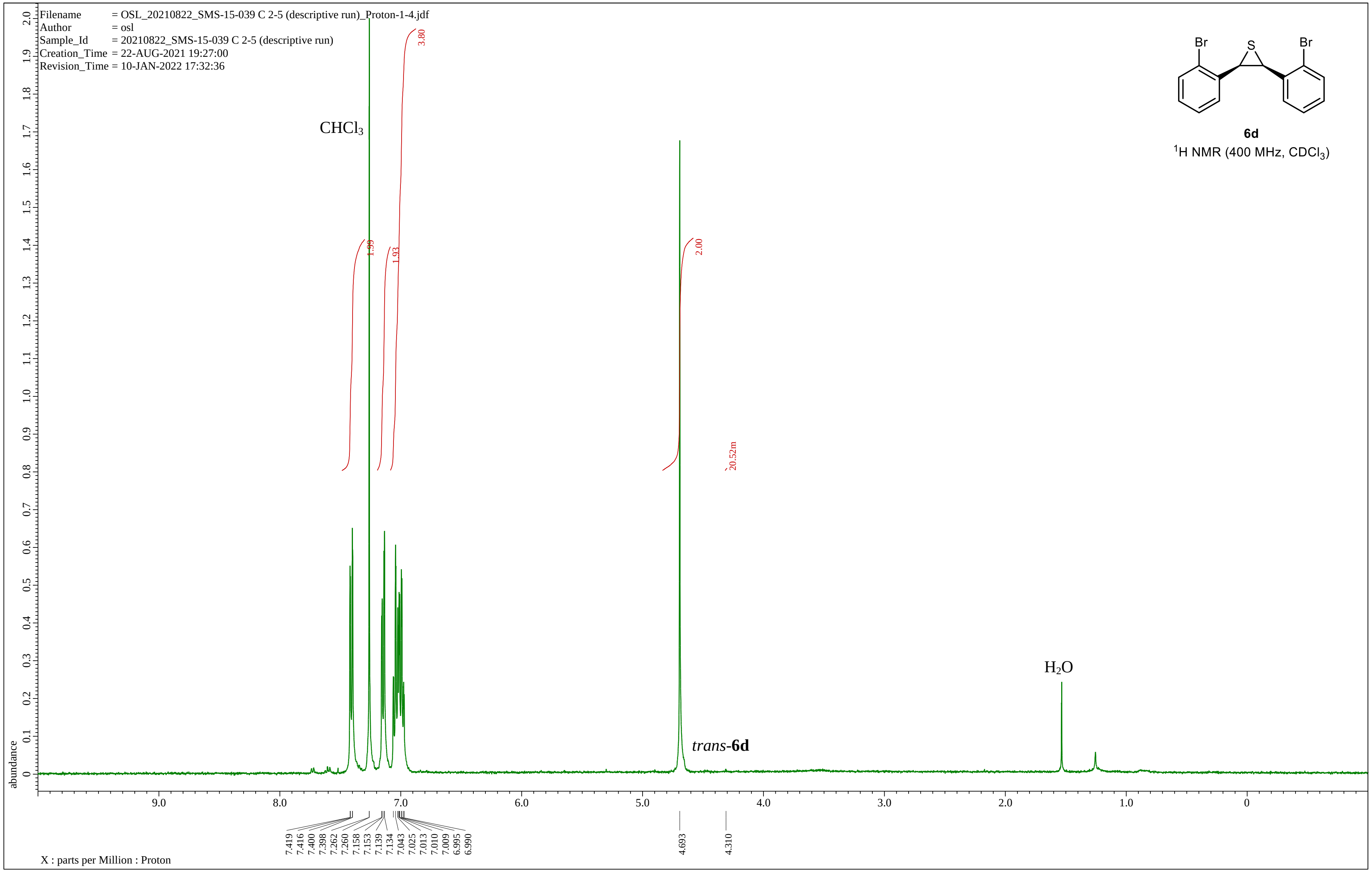

Supplementary Figure 76. ^{1}H NMR spectrum of compound **6d**, recorded at 400 MHz and 298 K in $CDCl_3$.

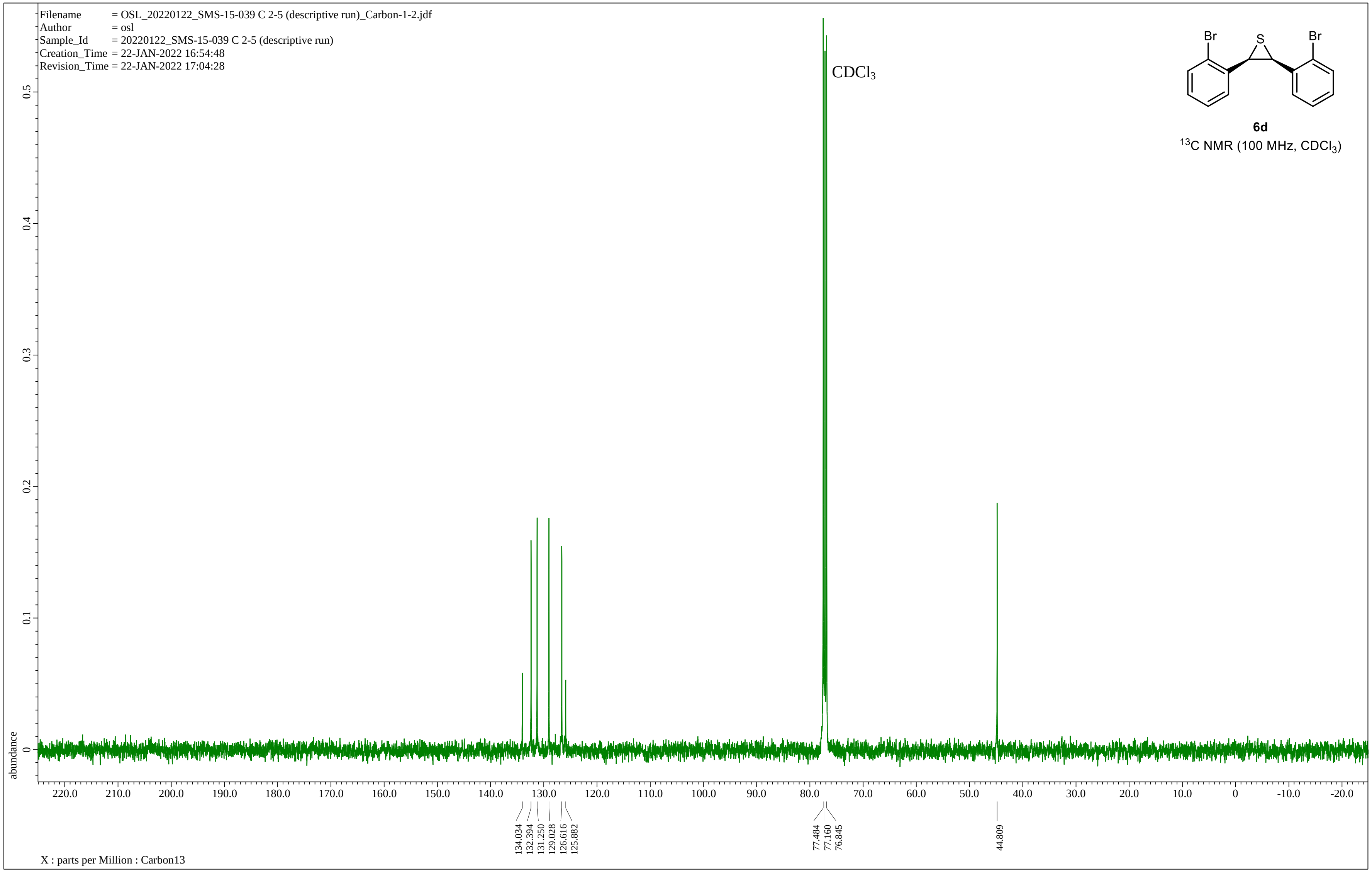

Supplementary Figure 77. ^{13}C NMR spectrum of compound **6d**, recorded at 100 MHz and 298 K in $CDCl_3$.

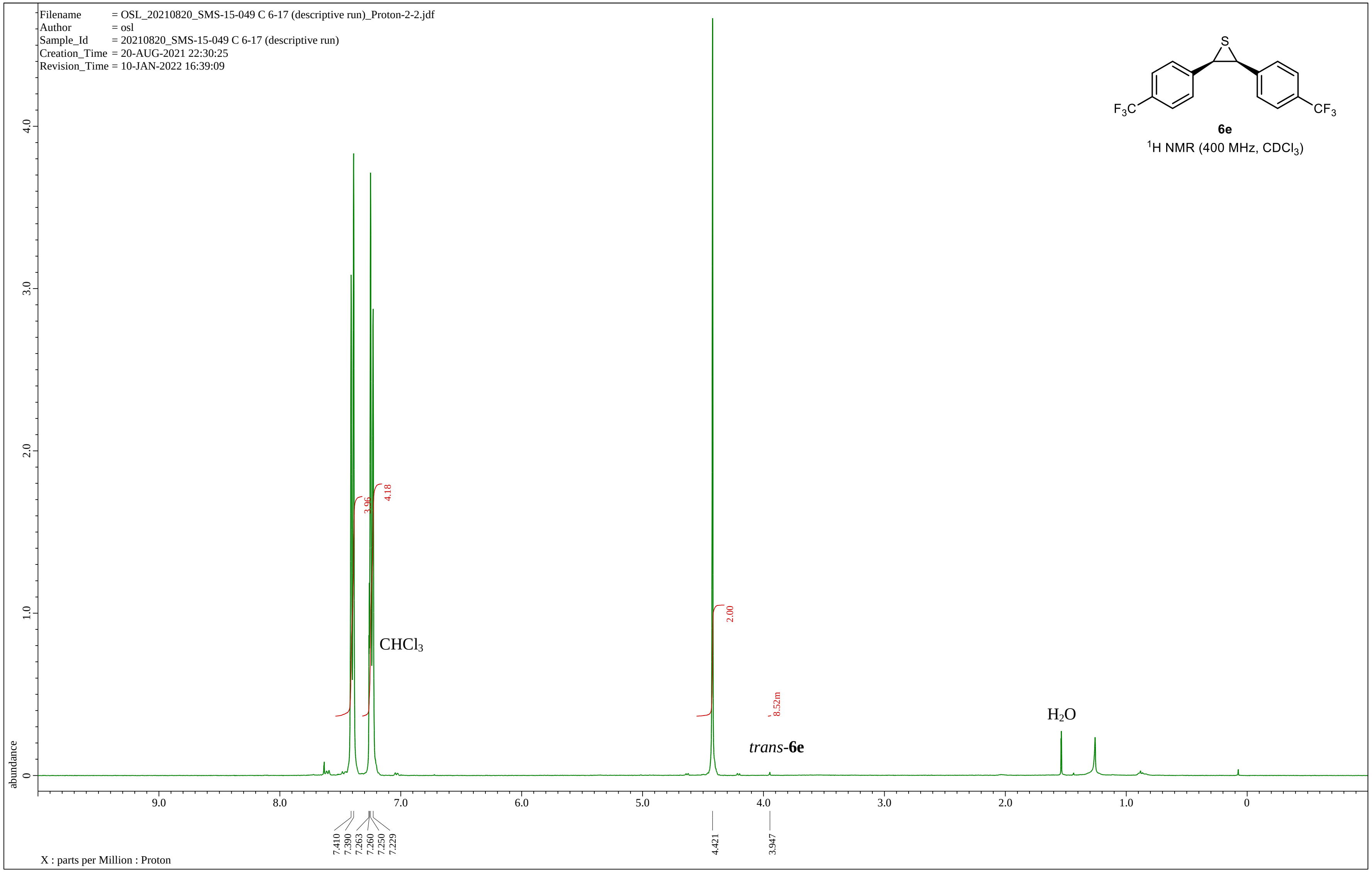

Supplementary Figure 78. ^{1}H NMR spectrum of compound **6e**, recorded at 400 MHz and 298 K in $CDCl_3$.

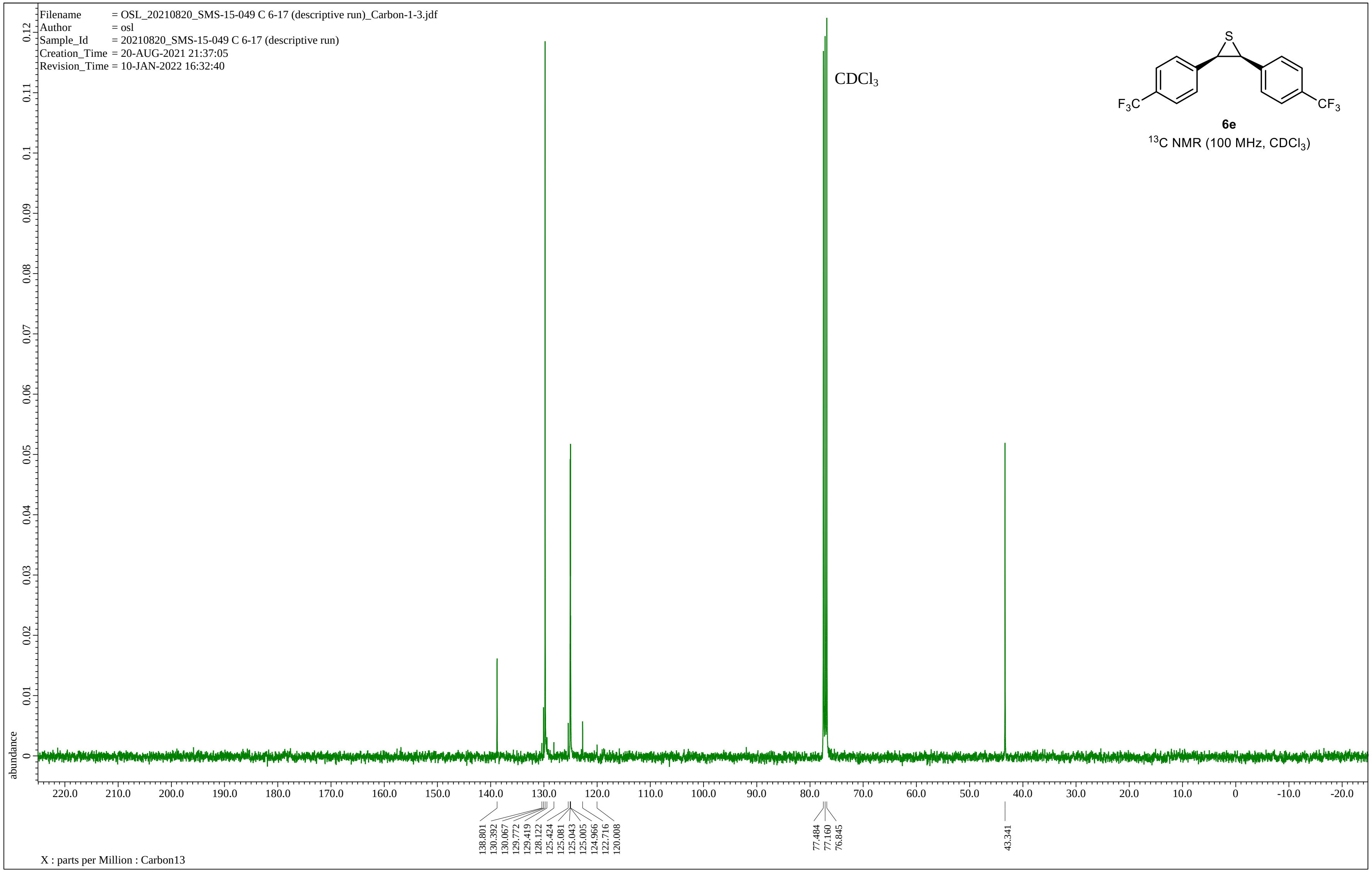

Supplementary Figure 79. ^{13}C NMR spectrum of compound **6e**, recorded at 100 MHz and 298 K in $CDCl_3$.

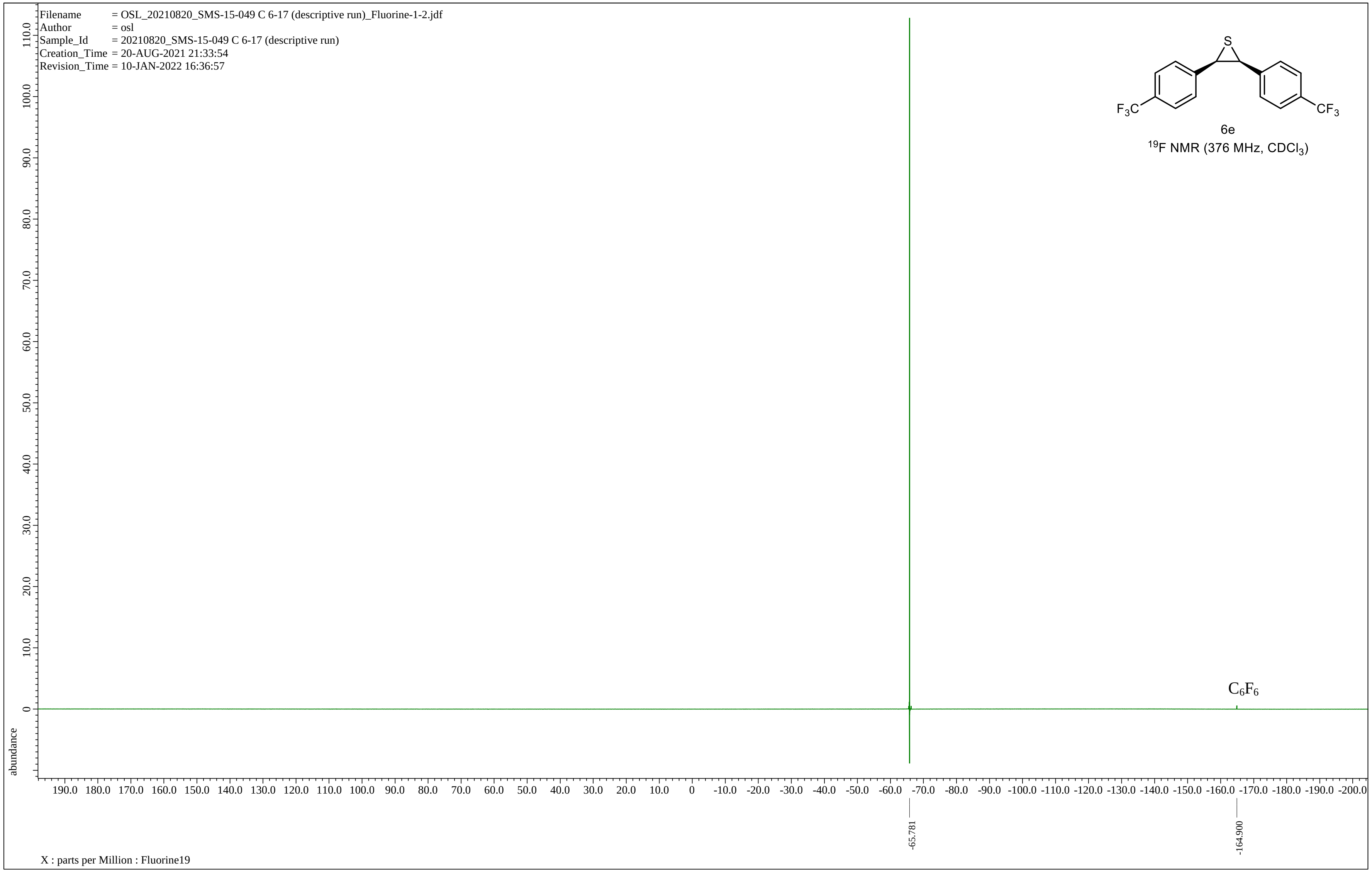

Supplementary Figure 80. ^{19}F NMR spectrum of compound **6e**, recorded at 376 MHz and 298 K in $CDCl_3$.

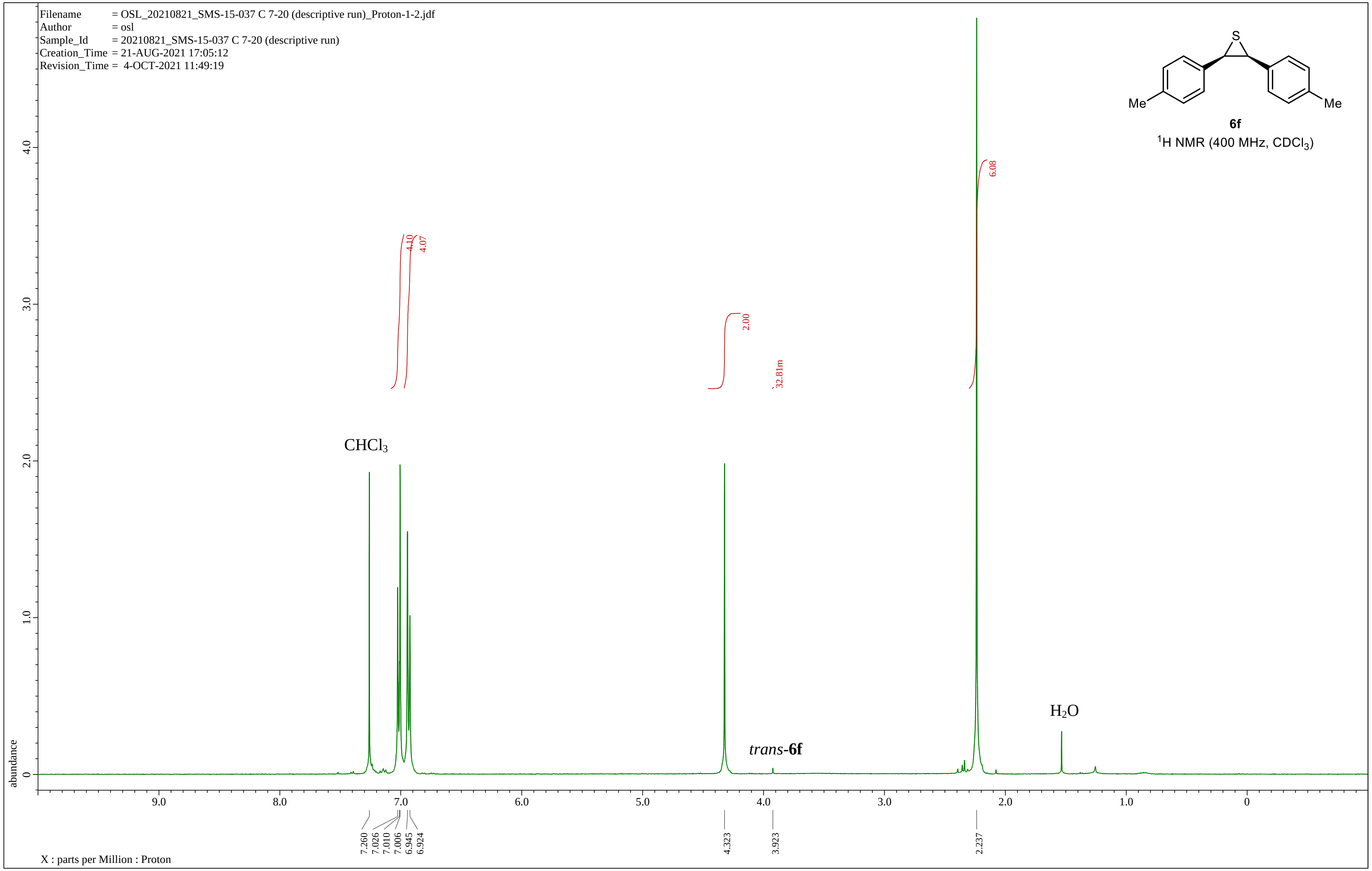

Supplementary Figure 81. ^{1}H NMR spectrum of compound **6f**, recorded at 400 MHz and 298 K in $CDCl_3$.

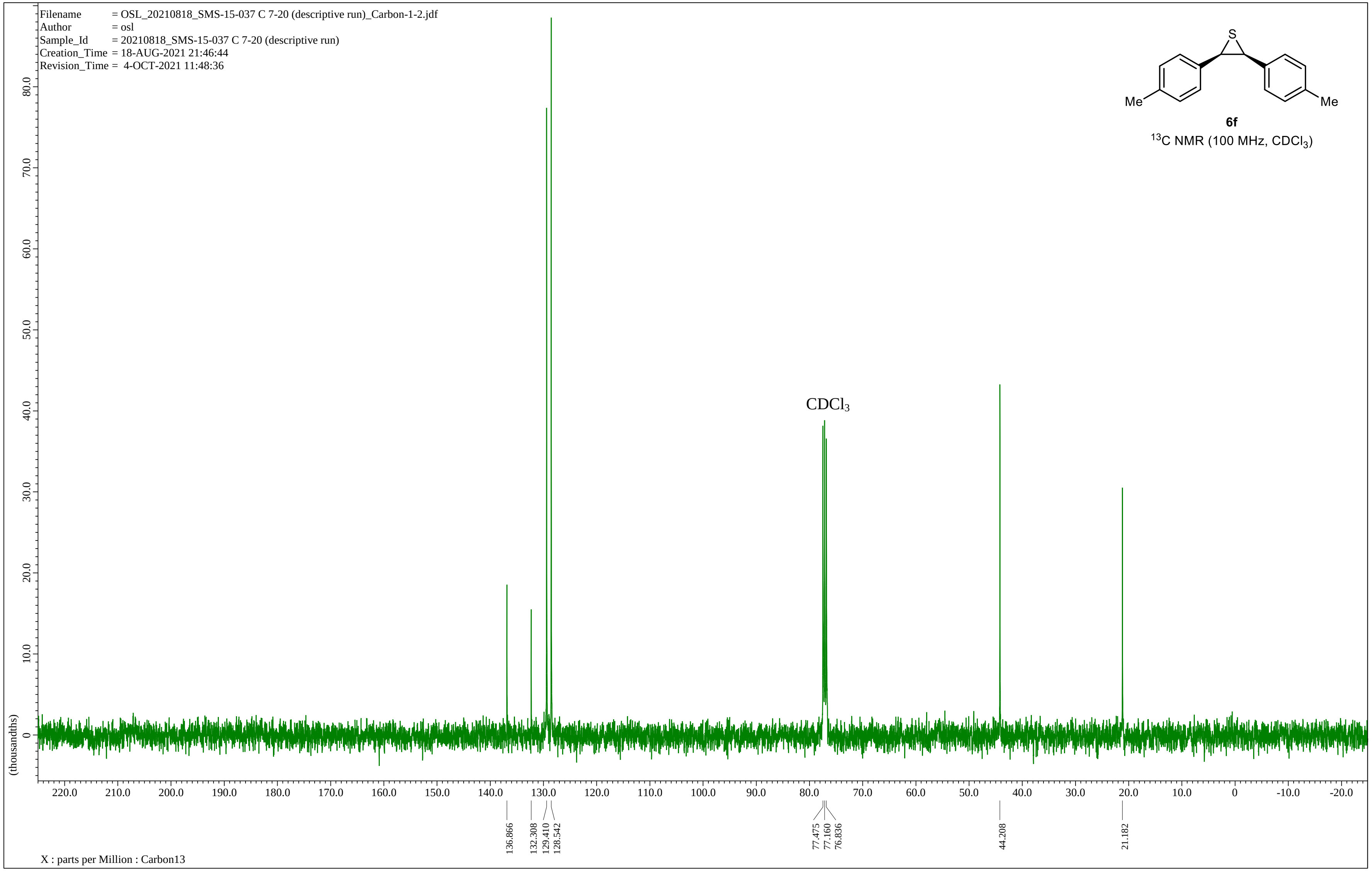

Supplementary Figure 82. ^{13}C NMR spectrum of compound **6f**, recorded at 100 MHz and 298 K in $CDCl_3$.

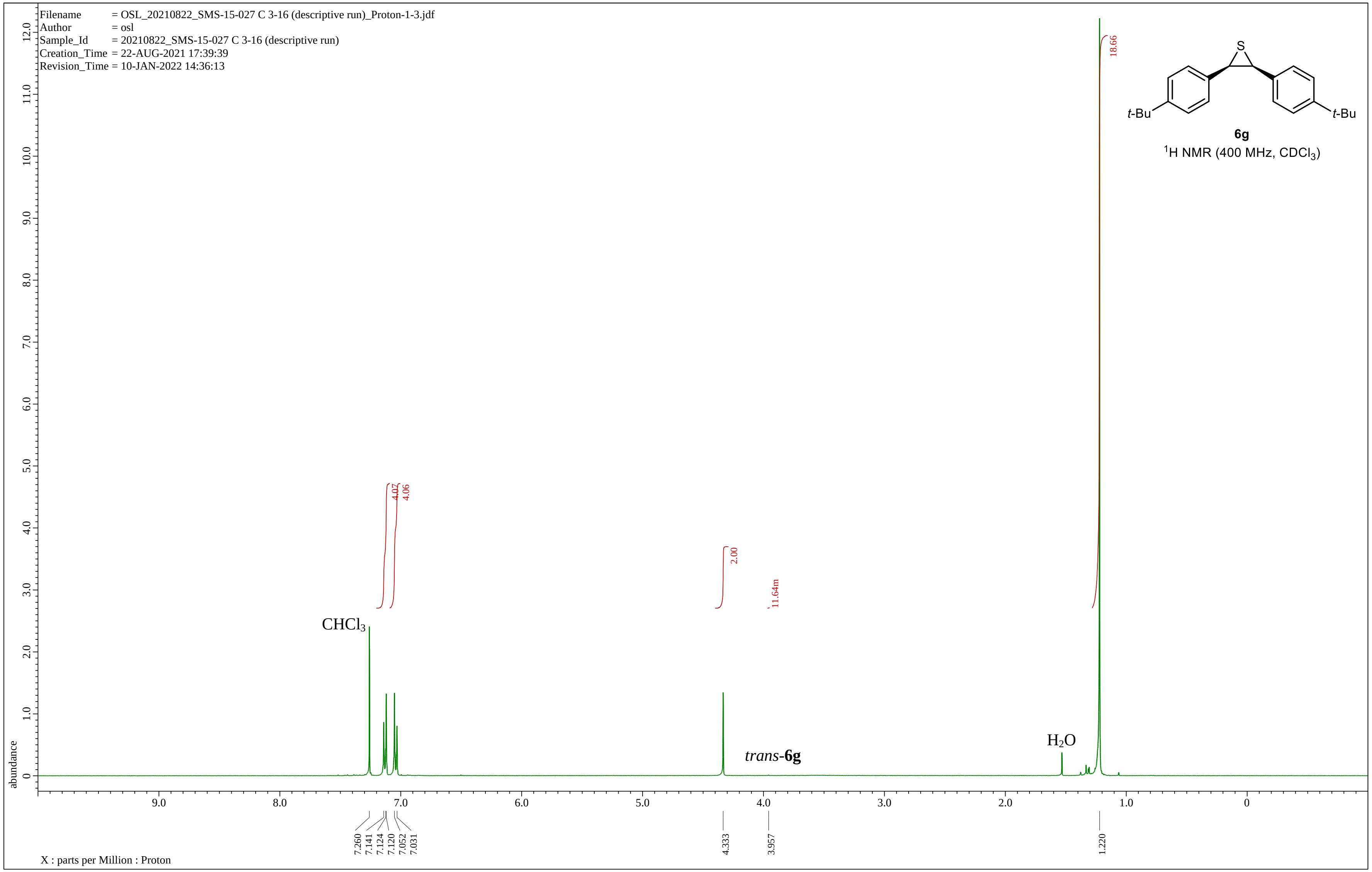

Supplementary Figure 83. ^{1}H NMR spectrum of compound **6g**, recorded at 400 MHz and 298 K in $CDCl_3$.

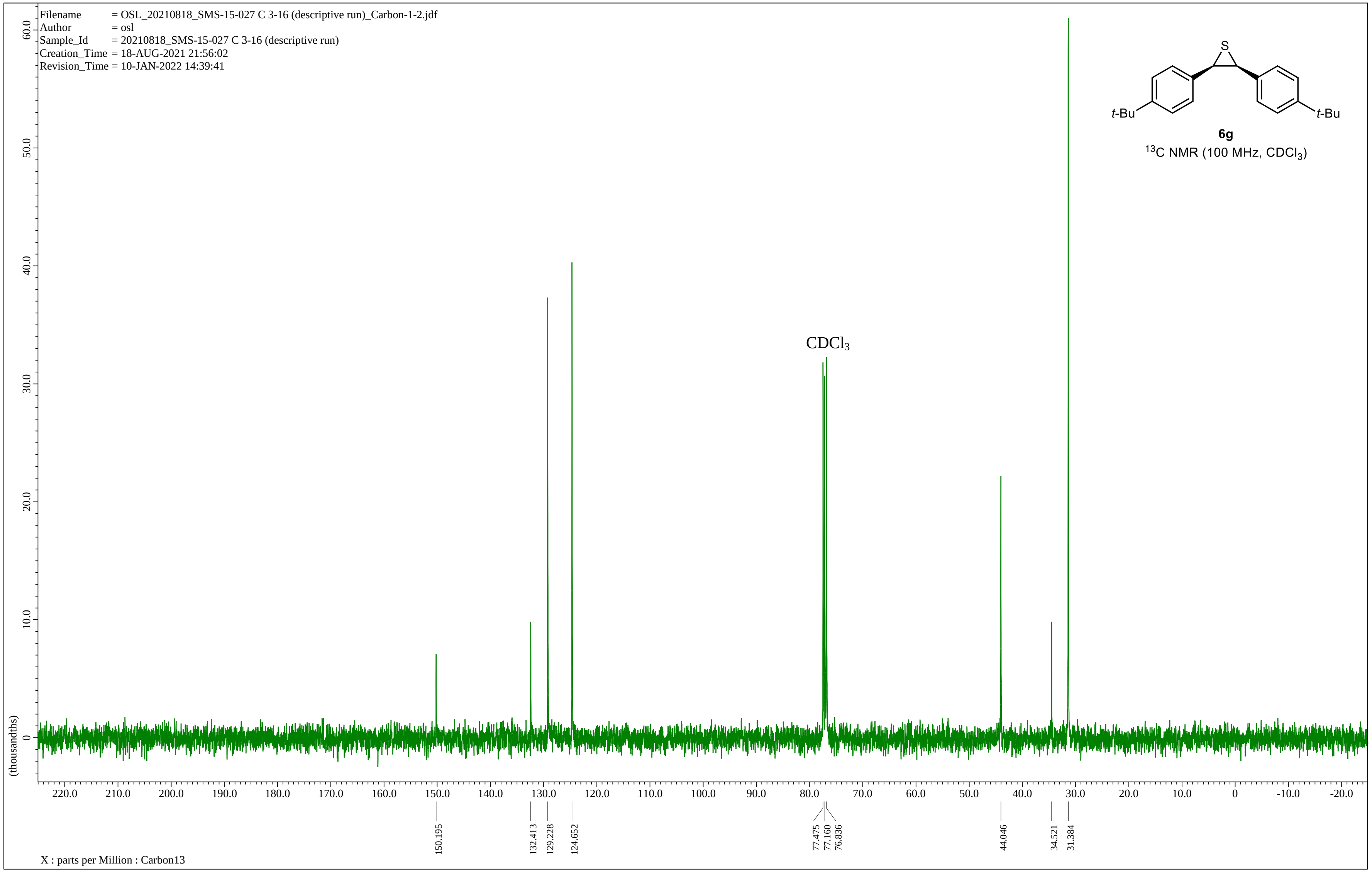

Supplementary Figure 84. ^{13}C NMR spectrum of compound **6g**, recorded at 100 MHz and 298 K in $CDCl_3$.

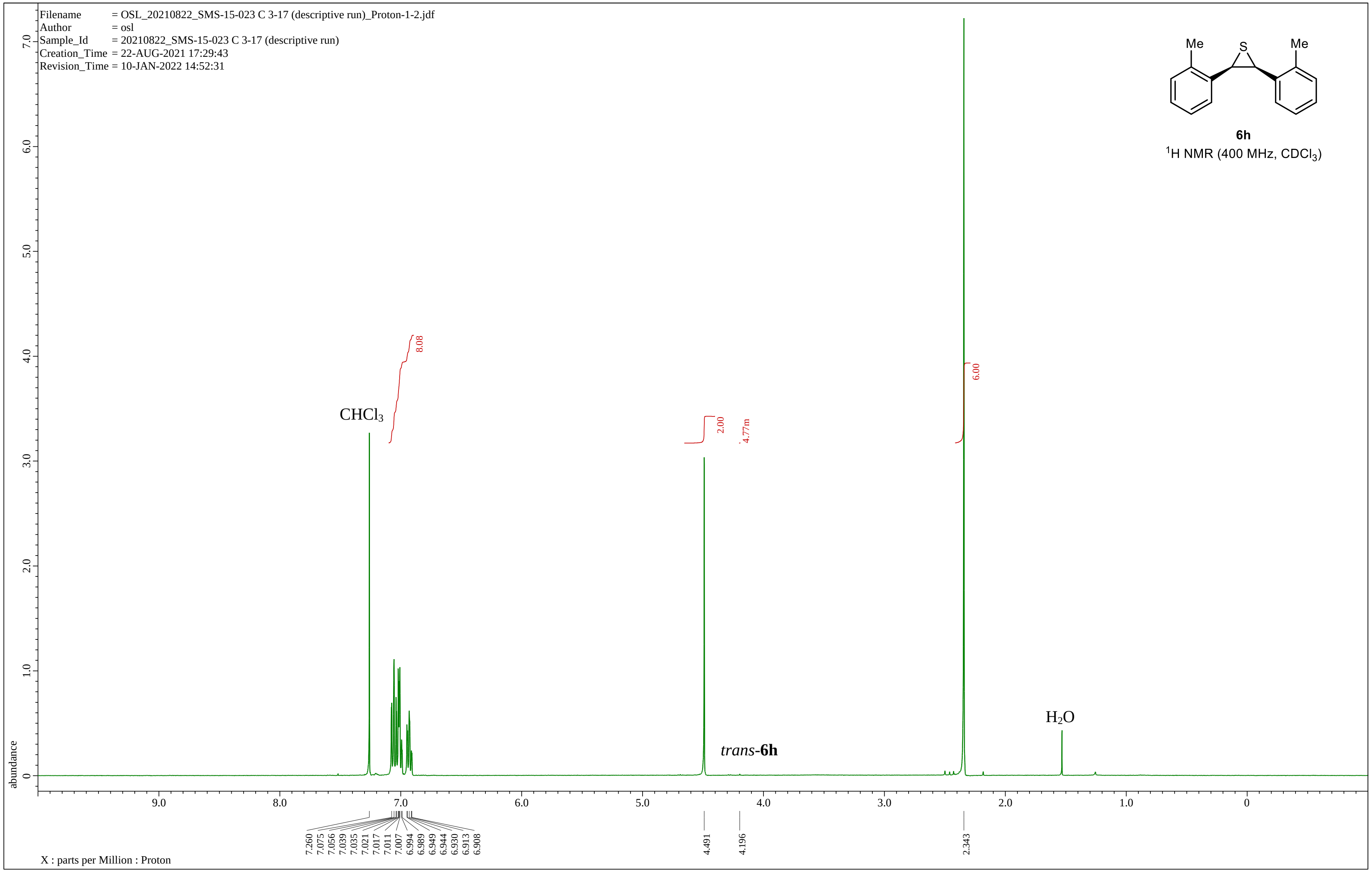

Supplementary Figure 85. ^{1}H NMR spectrum of compound **6h**, recorded at 400 MHz and 298 K in $CDCl_3$.

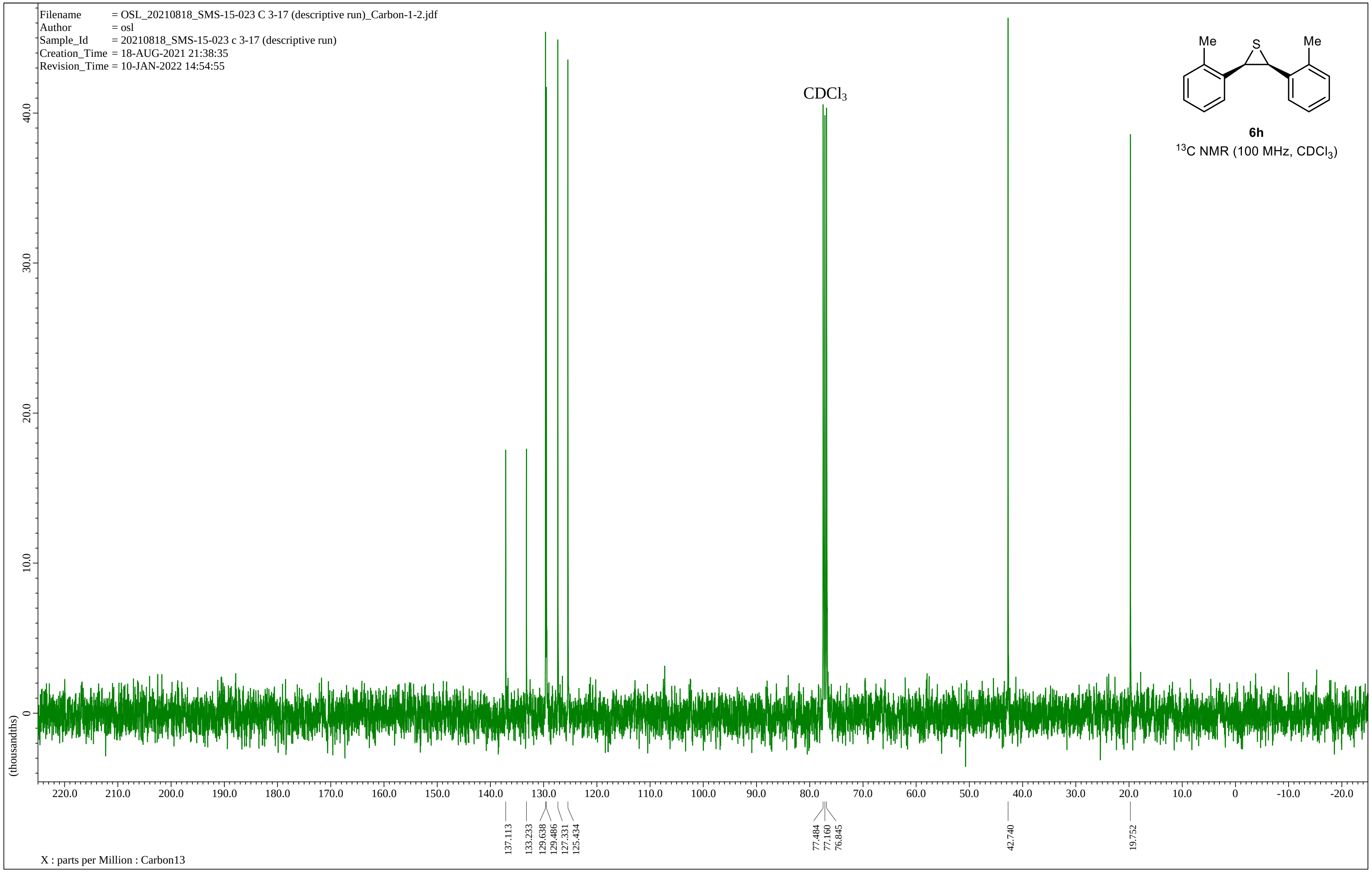

Supplementary Figure 86. ^{13}C NMR spectrum of compound **6h**, recorded at 100 MHz and 298 K in $CDCl_3$.

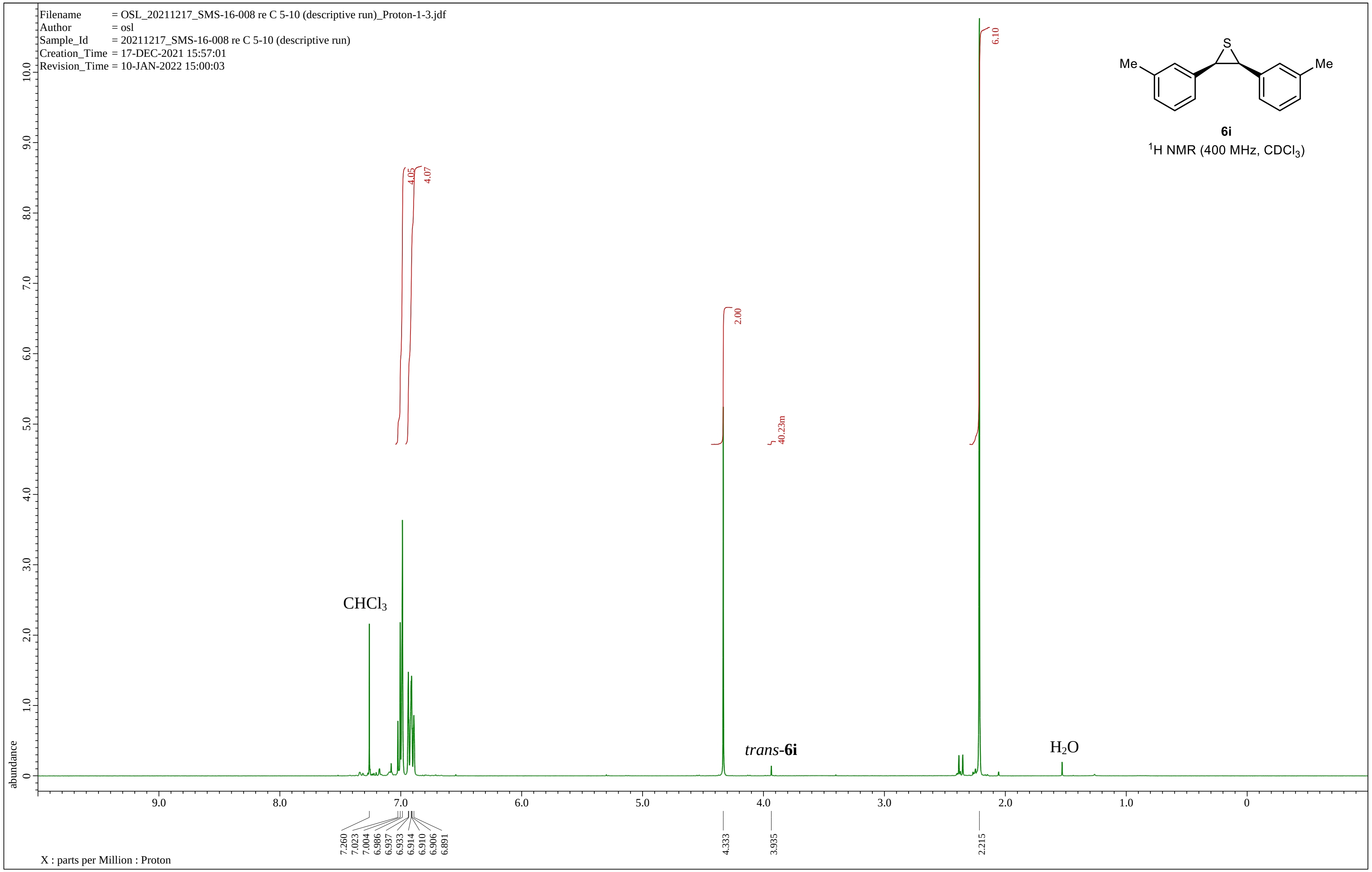

Supplementary Figure 87. ^{1}H NMR spectrum of compound **6i**, recorded at 400 MHz and 298 K in $CDCl_3$.

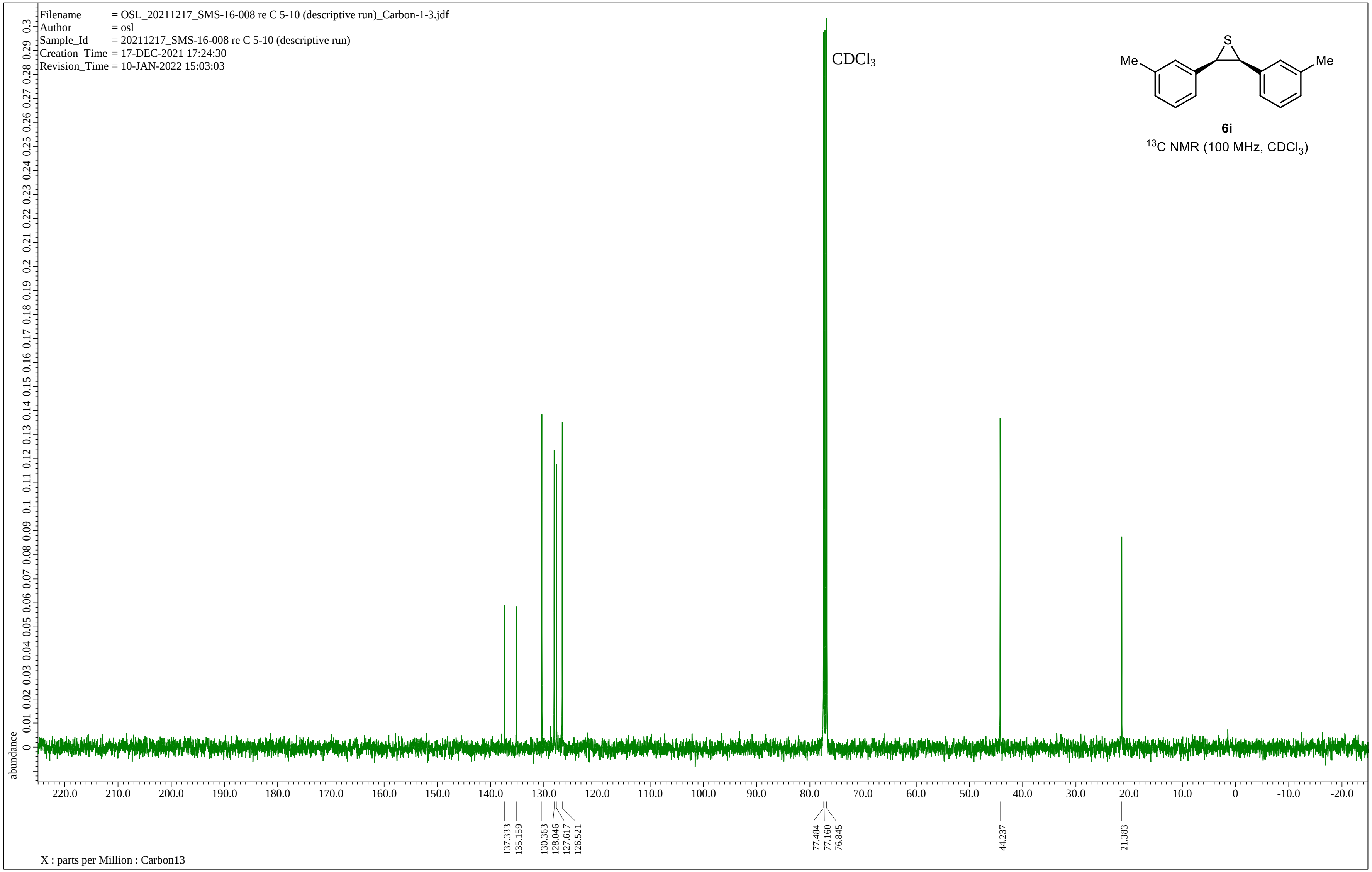

Supplementary Figure 88. ^{13}C NMR spectrum of compound **6i**, recorded at 100 MHz and 298 K in $CDCl_3$.

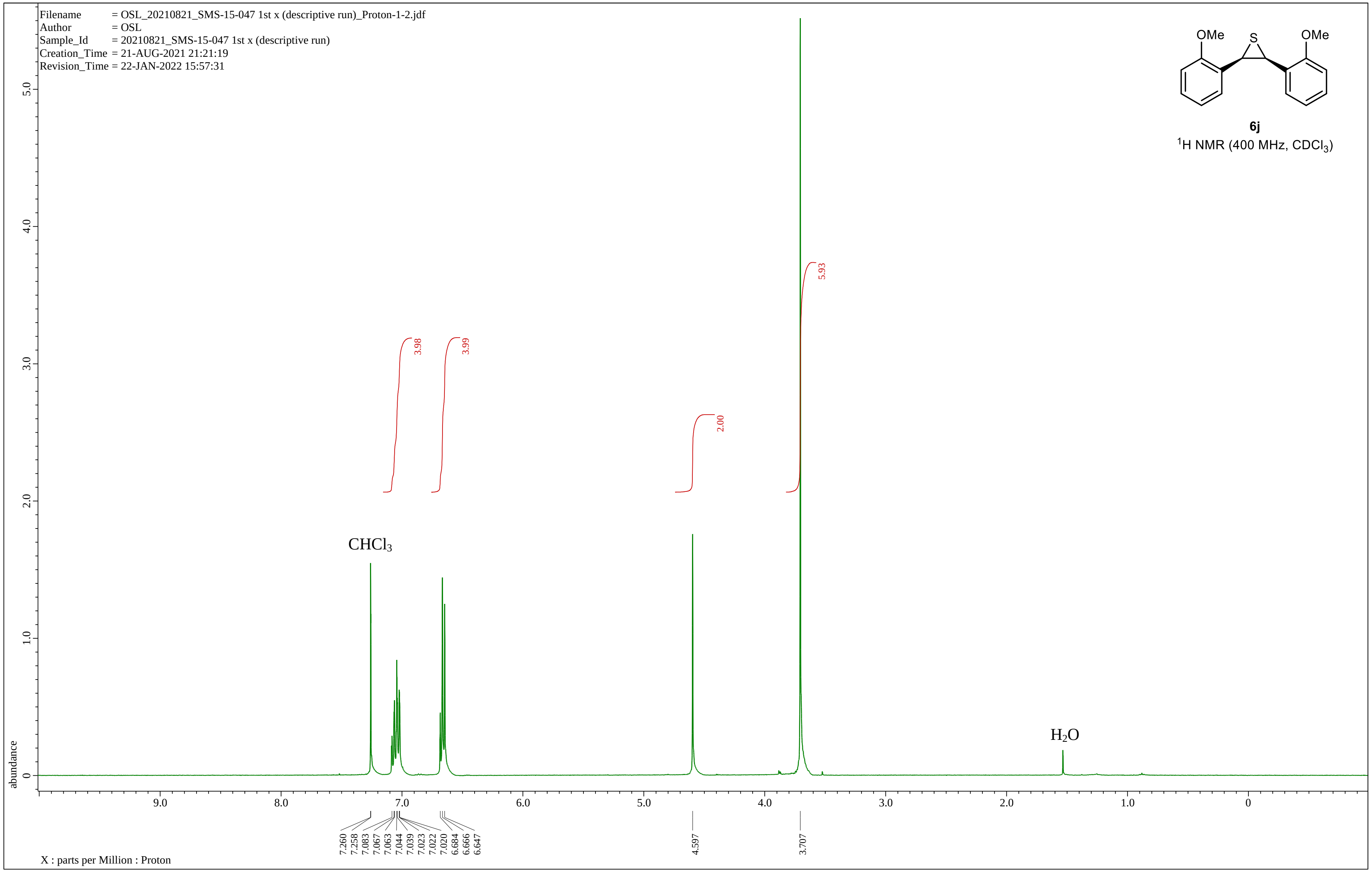

Supplementary Figure 89. ^{1}H NMR spectrum of compound **6j**, recorded at 400 MHz and 298 K in $CDCl_3$.

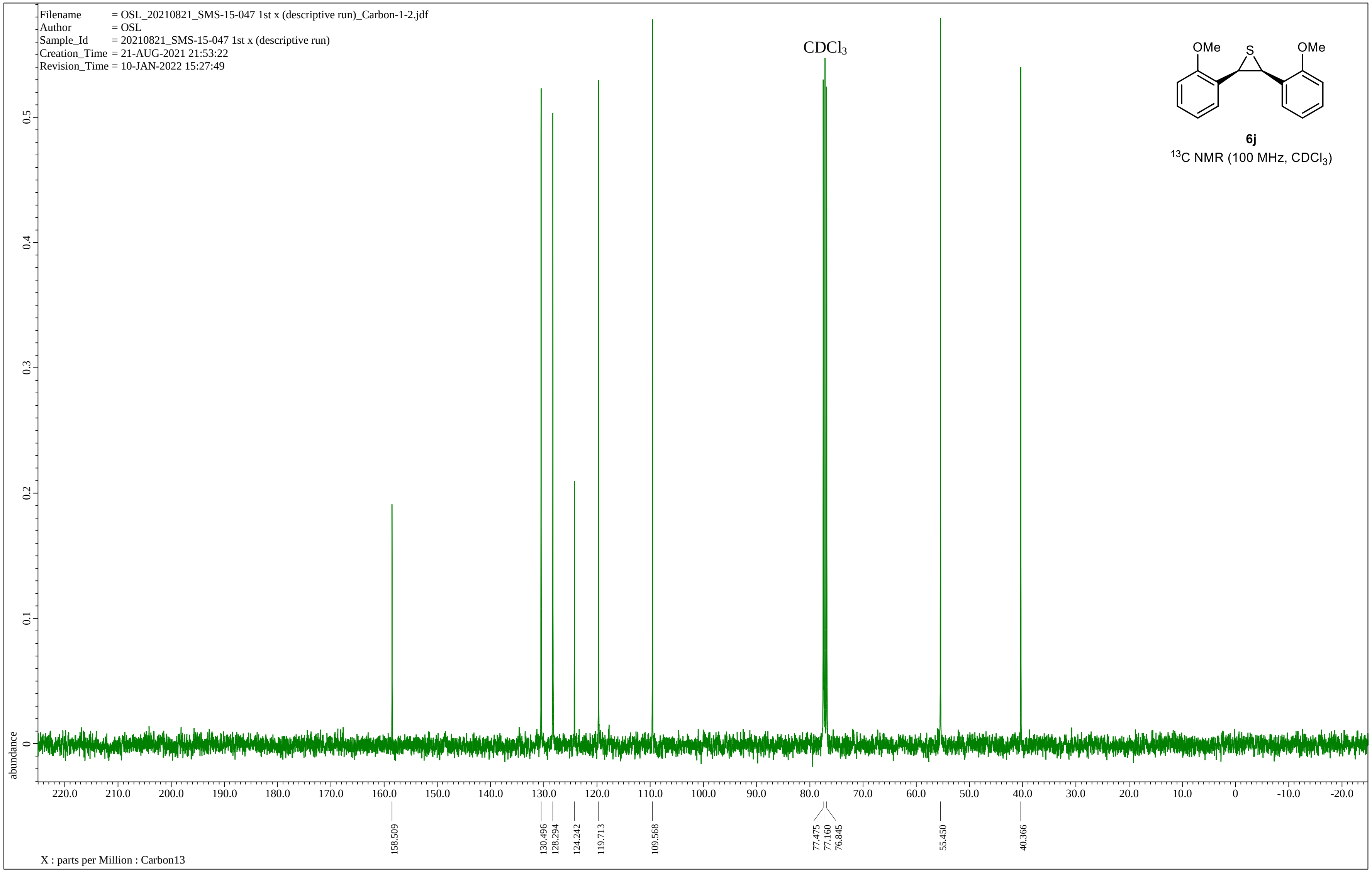

Supplementary Figure 90. ^{13}C NMR spectrum of compound **6j**, recorded at 100 MHz and 298 K in $CDCl_3$.

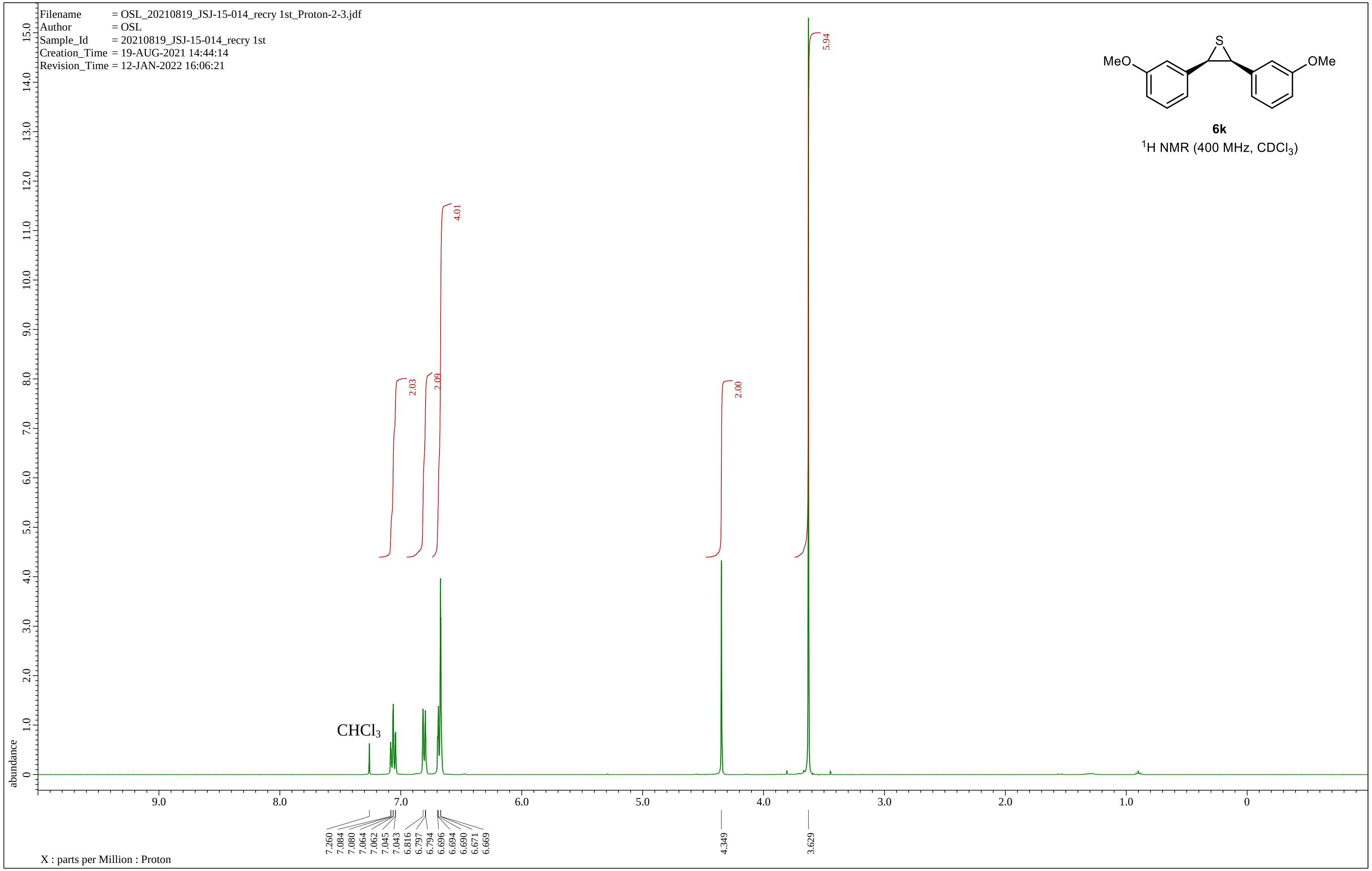

Supplementary Figure 91. ^{1}H NMR spectrum of compound **6k**, recorded at 400 MHz and 298 K in $CDCl_3$.

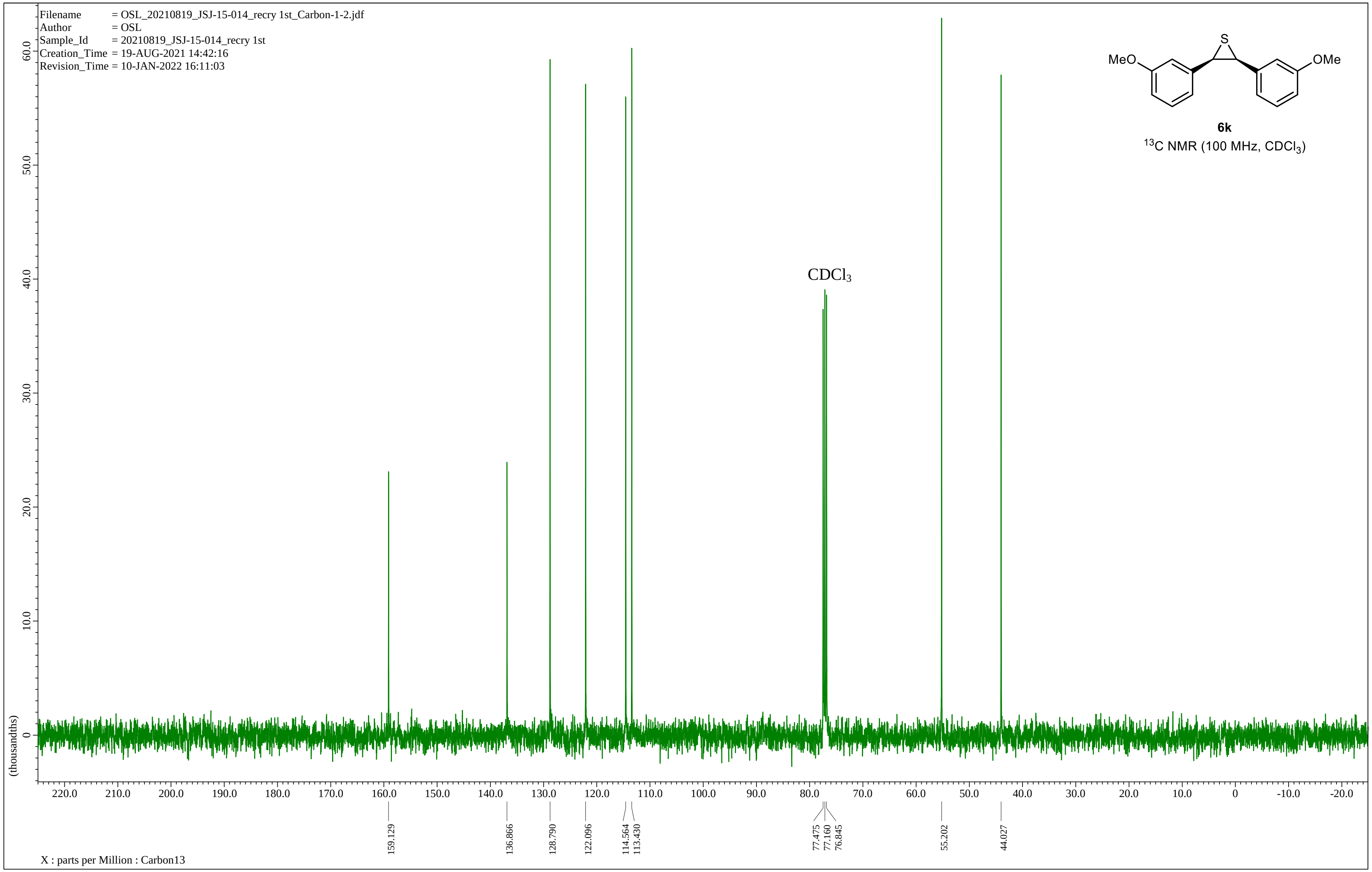

Supplementary Figure 92. ^{13}C NMR spectrum of compound **6k**, recorded at 100 MHz and 298 K in $CDCl_3$.

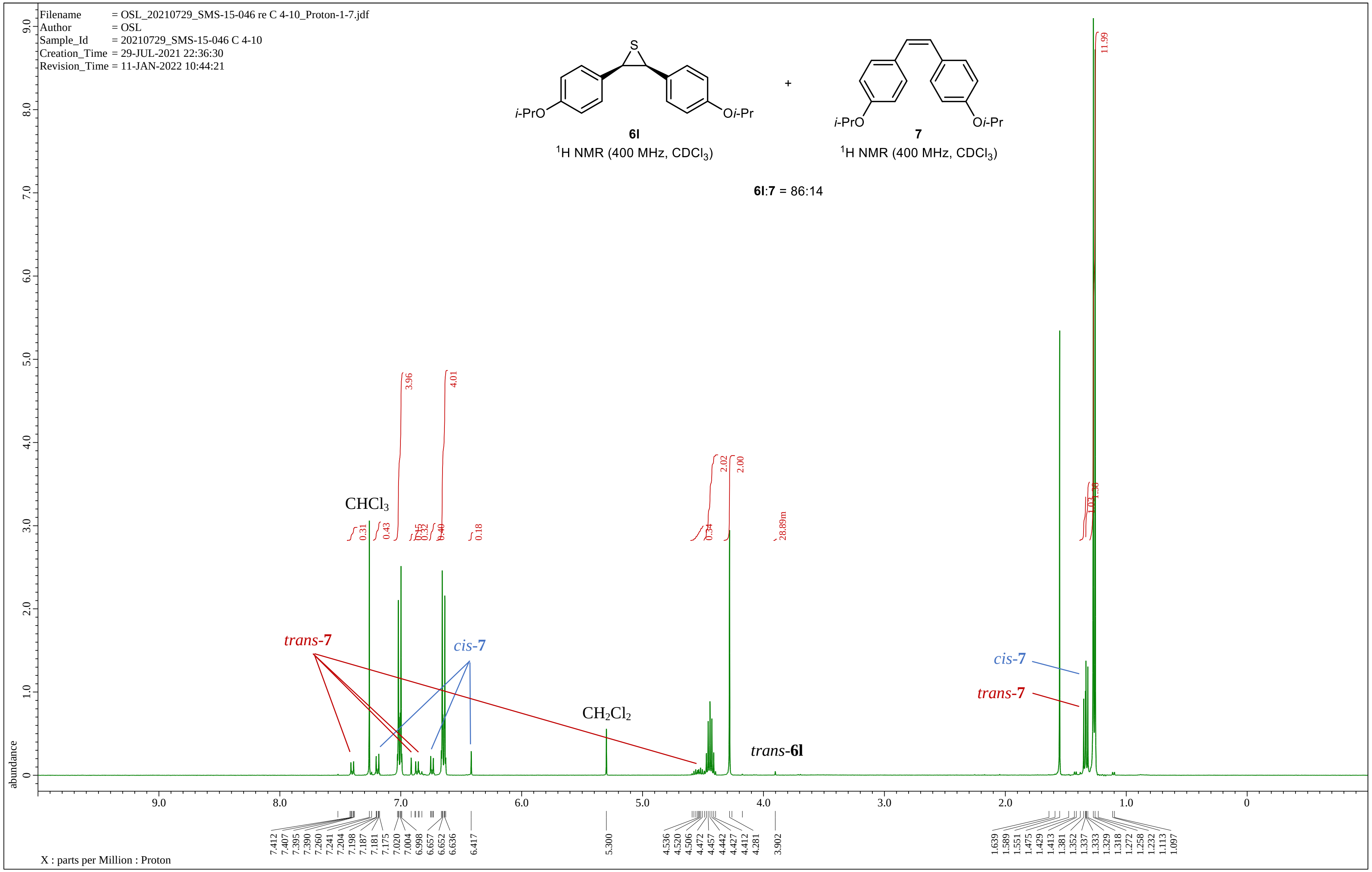

Supplementary Figure 93. ^{1}H NMR spectrum of compounds **6l** and **7**, recorded at 400 MHz and 298 K in $CDCl_3$.

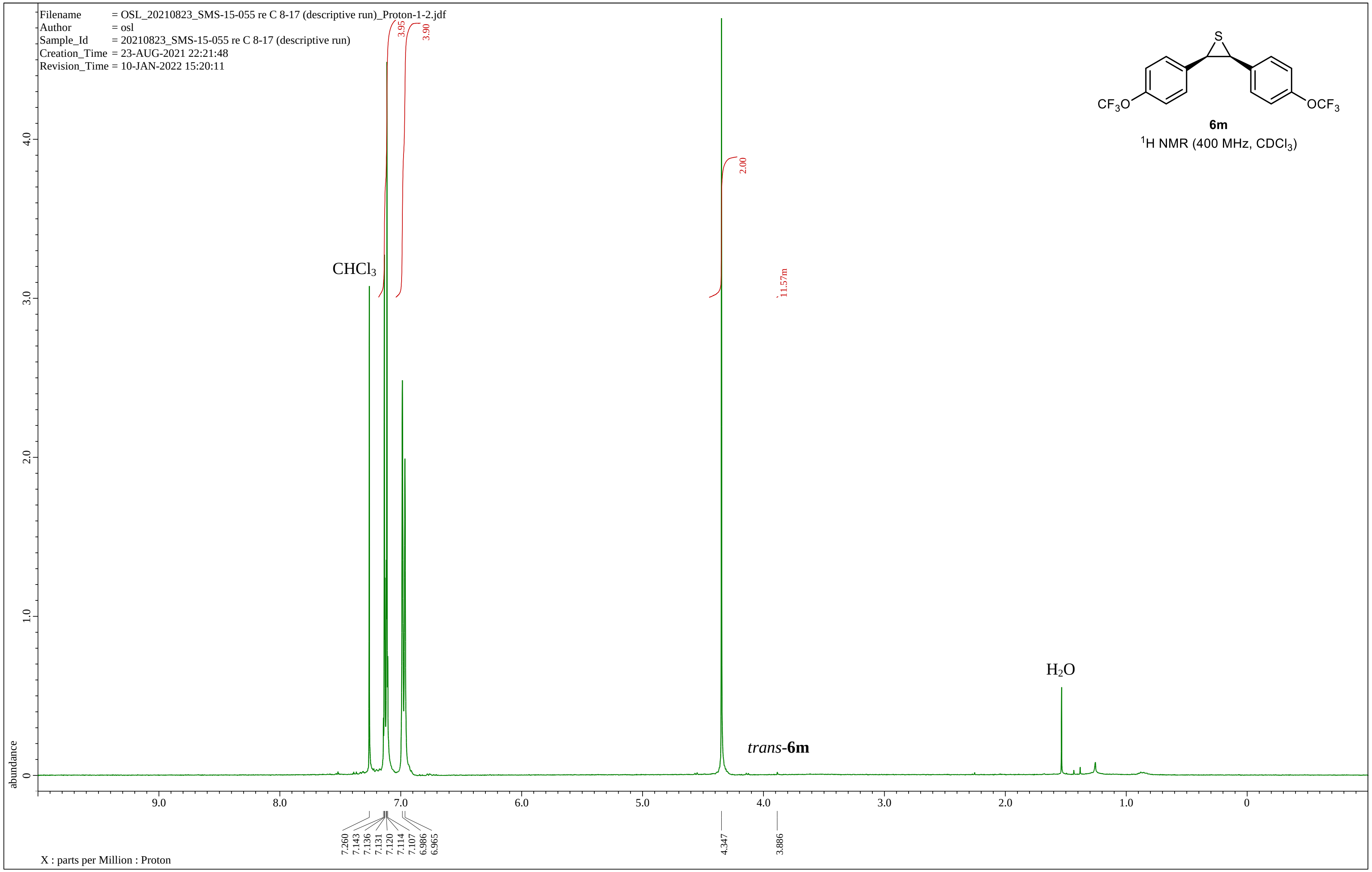

Supplementary Figure 94. ^{1}H NMR spectrum of compound **6m**, recorded at 400 MHz and 298 K in $CDCl_3$.

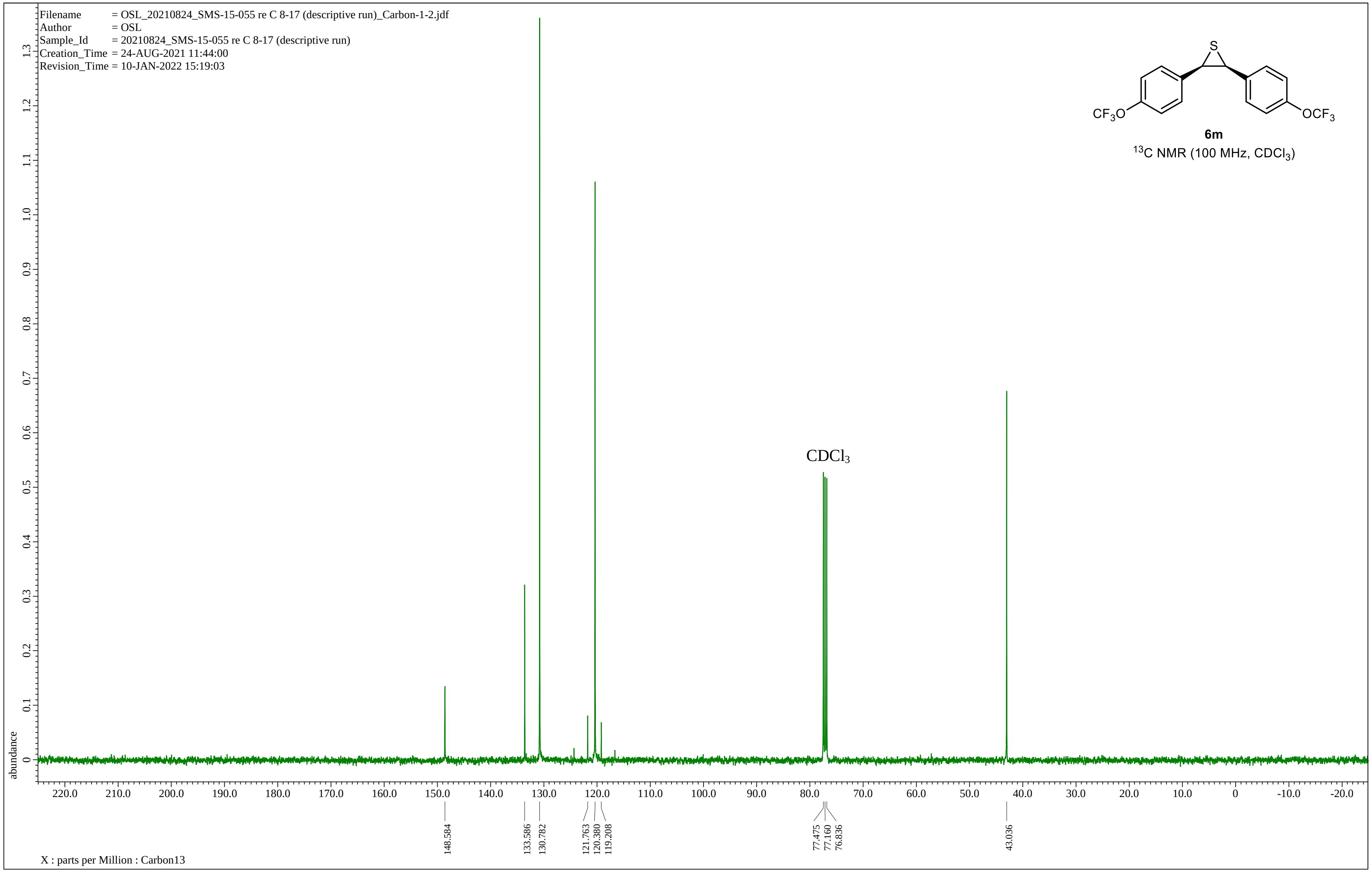

Supplementary Figure 95. ^{13}C NMR spectrum of compound **6m**, recorded at 100 MHz and 298 K in $CDCl_3$.

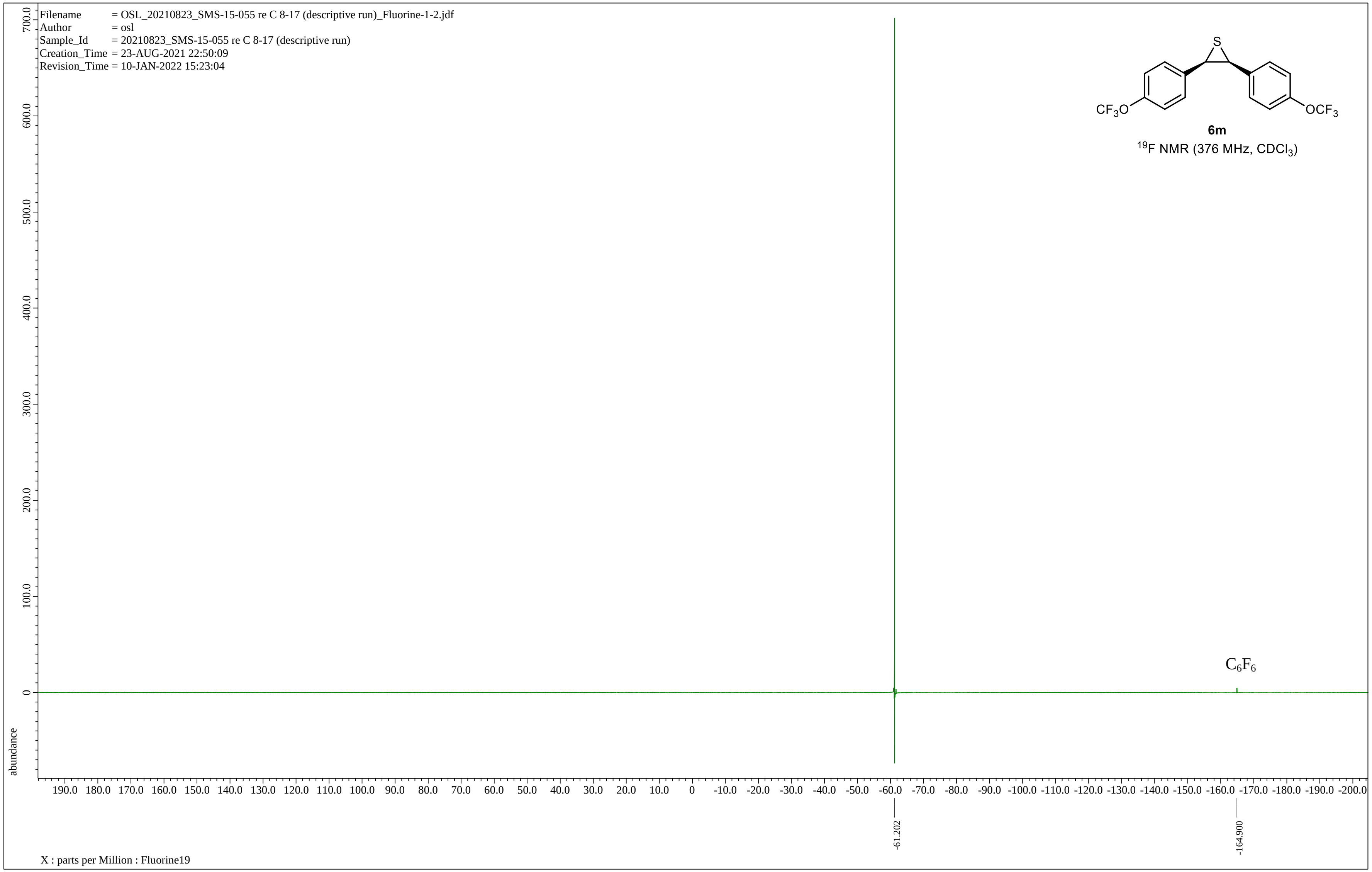

Supplementary Figure 96. ^{19}F NMR spectrum of compound **6m**, recorded at 376 MHz and 298 K in $CDCl_3$.

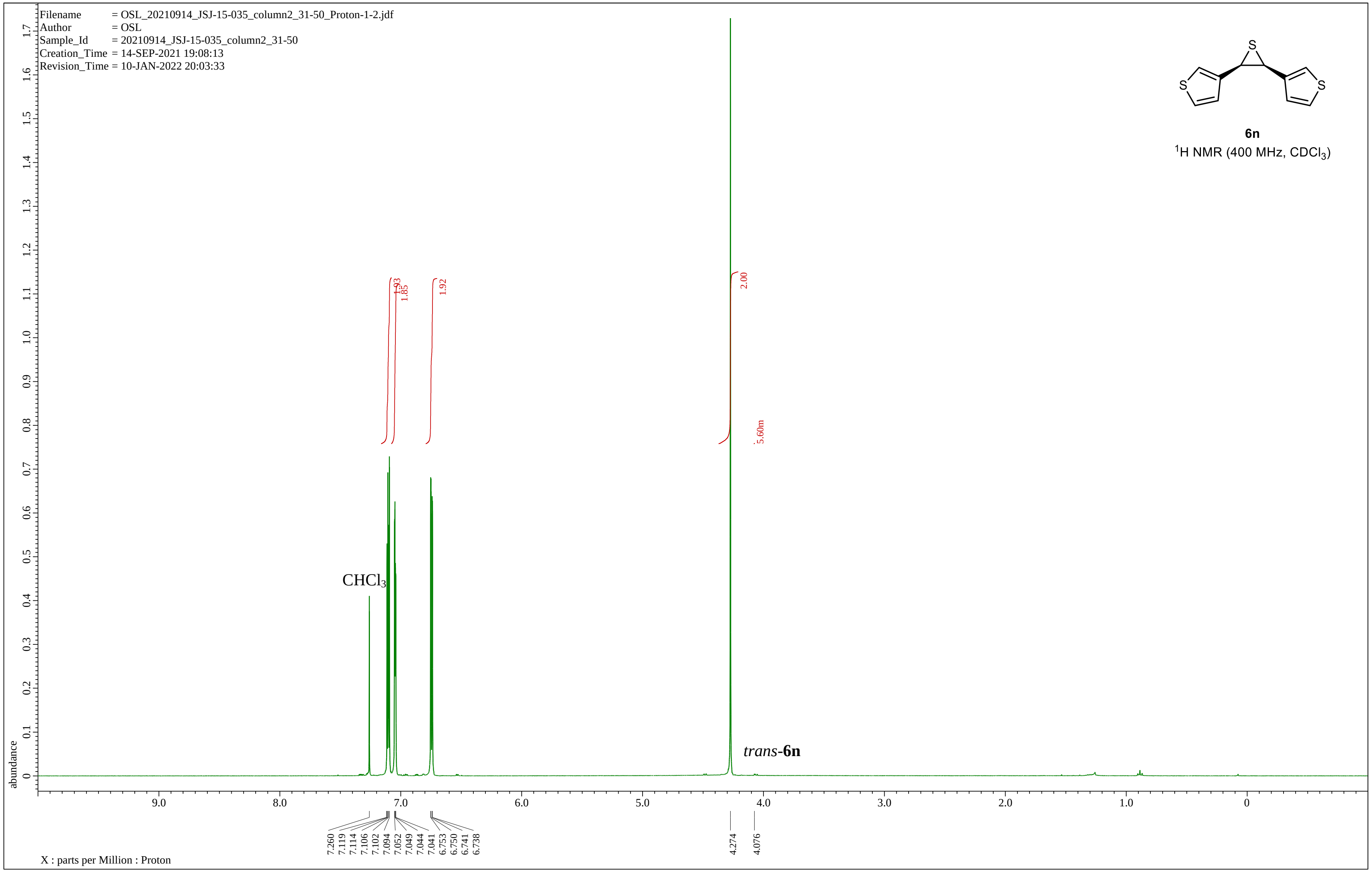

Supplementary Figure 97. ^{1}H NMR spectrum of compound **6n**, recorded at 400 MHz and 298 K in $CDCl_3$.

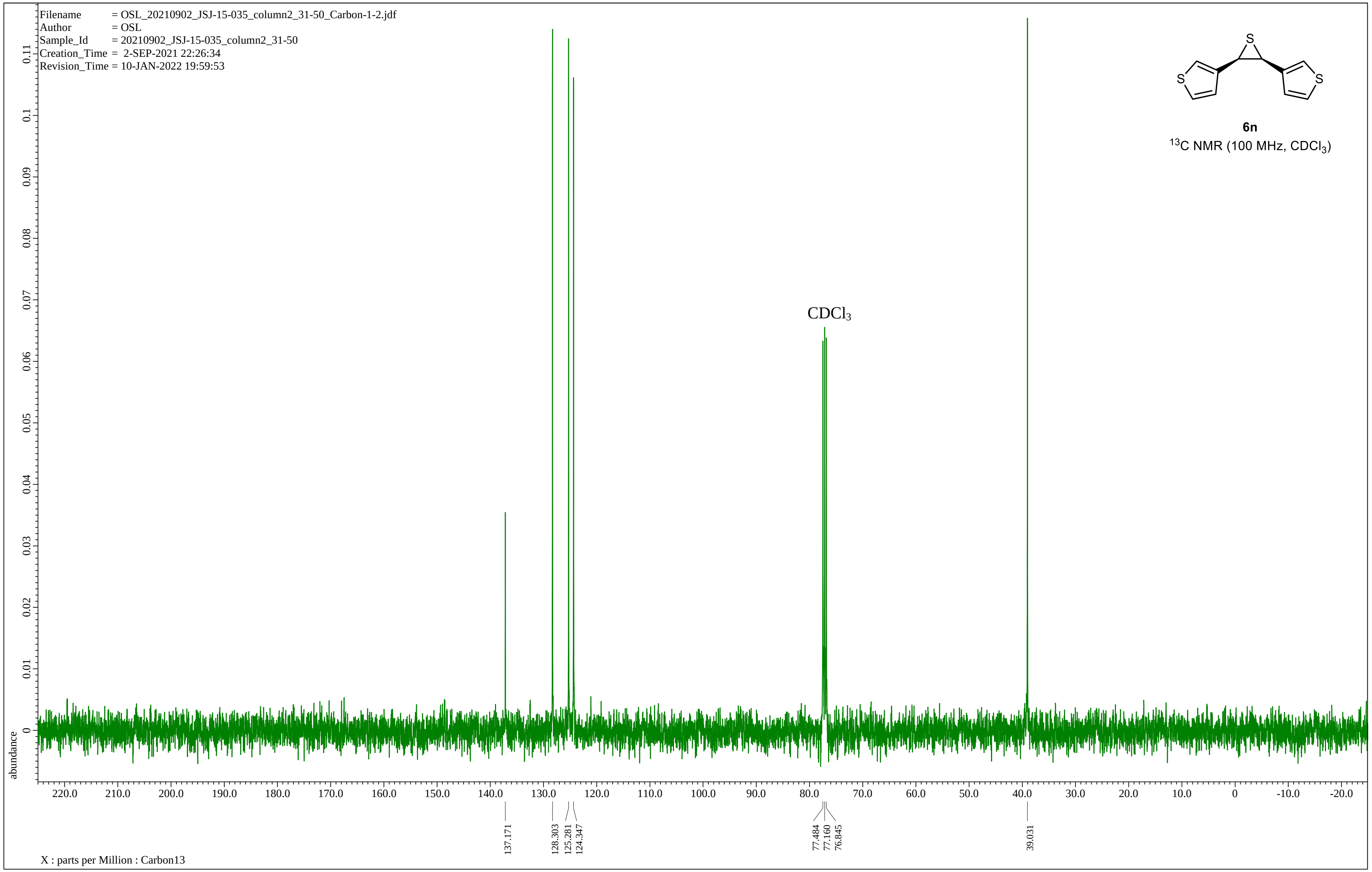

Supplementary Figure 98. ^{13}C NMR spectrum of compound **6n**, recorded at 100 MHz and 298 K in $CDCl_3$.

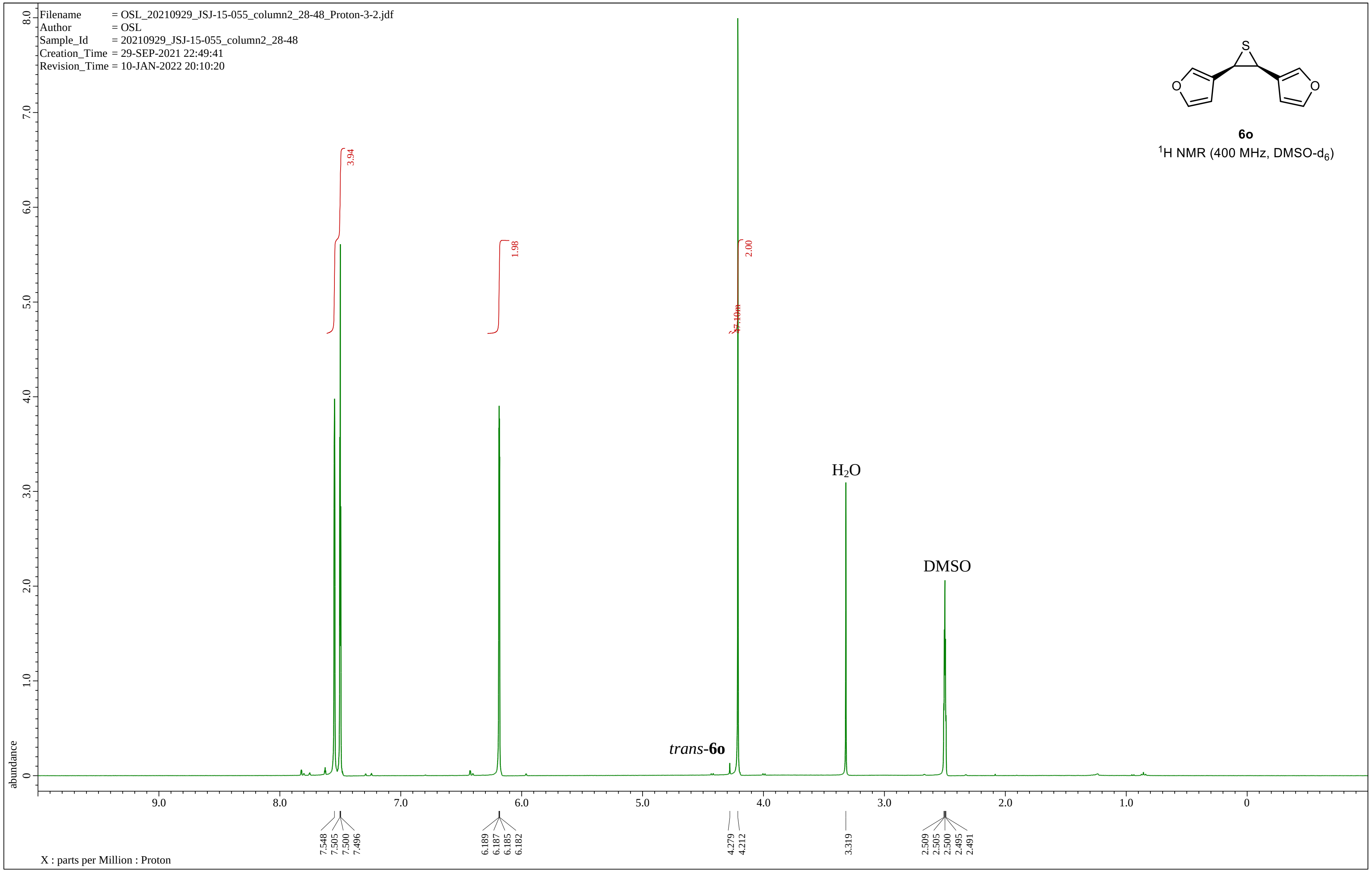

Supplementary Figure 99. ^{1}H NMR spectrum of compound **6o**, recorded at 400 MHz and 298 K in DMSO-d_6.

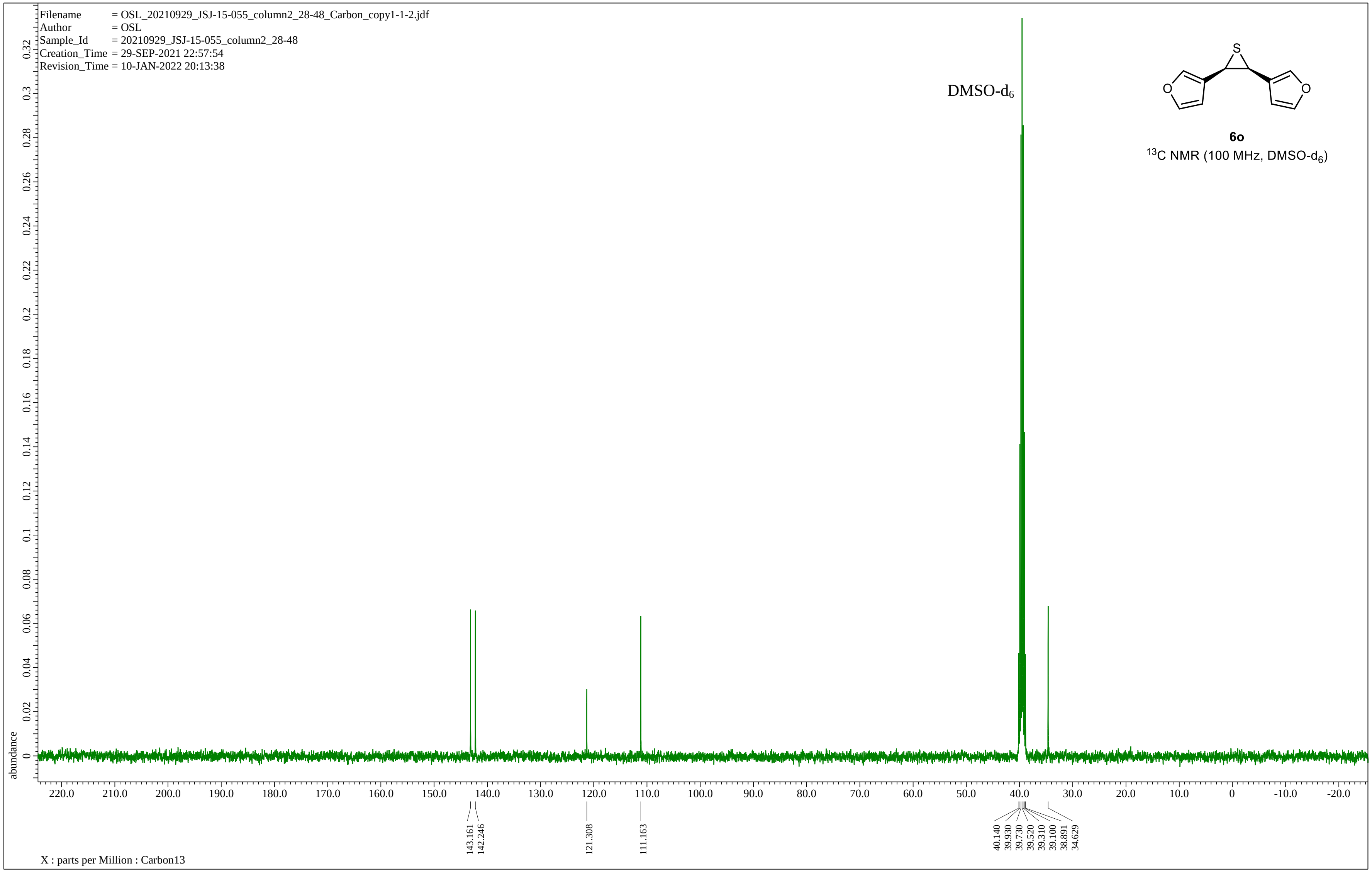

Supplementary Figure 100. ^{13}C NMR spectrum of compound **6o**, recorded at 100 MHz and 298 K in DMSO-d_6.

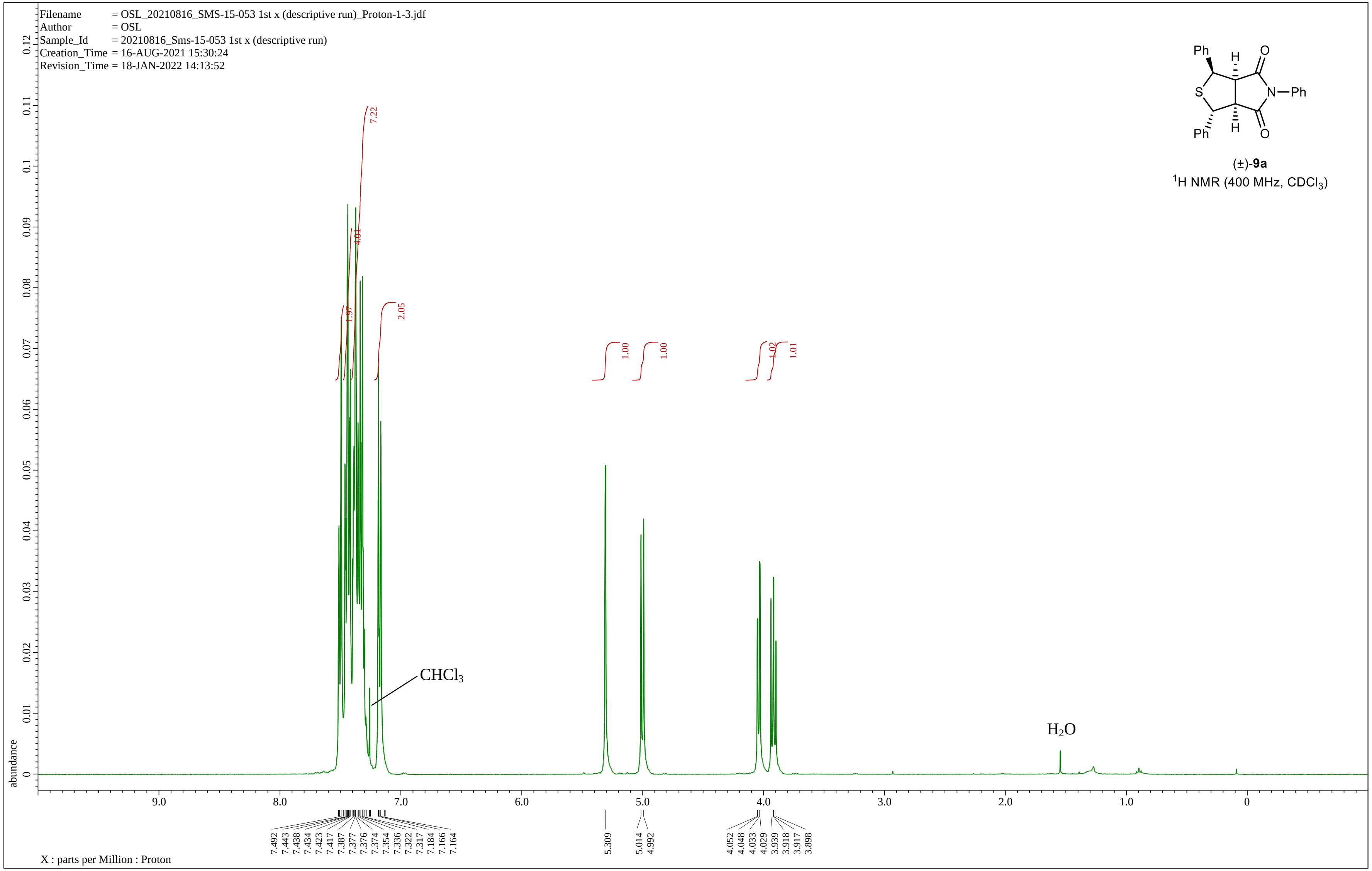

Supplementary Figure 101. ^{1}H NMR spectrum of compound (±)-**9a**, recorded at 400 MHz and 298 K in $CDCl_3$.

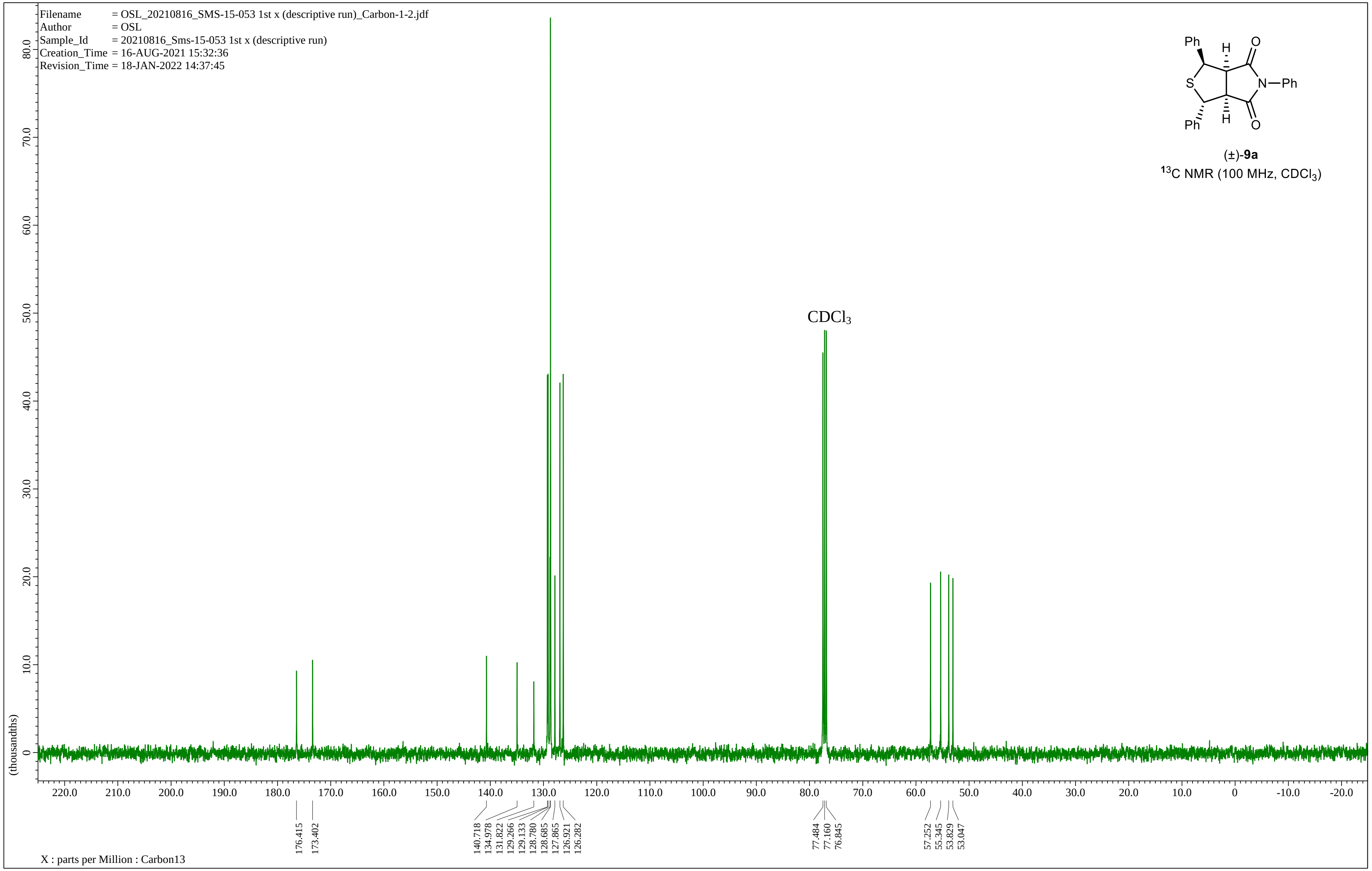

Supplementary Figure 102. ^{13}C NMR spectrum of compound (±)-**9a**, recorded at 100 MHz and 298 K in $CDCl_3$.

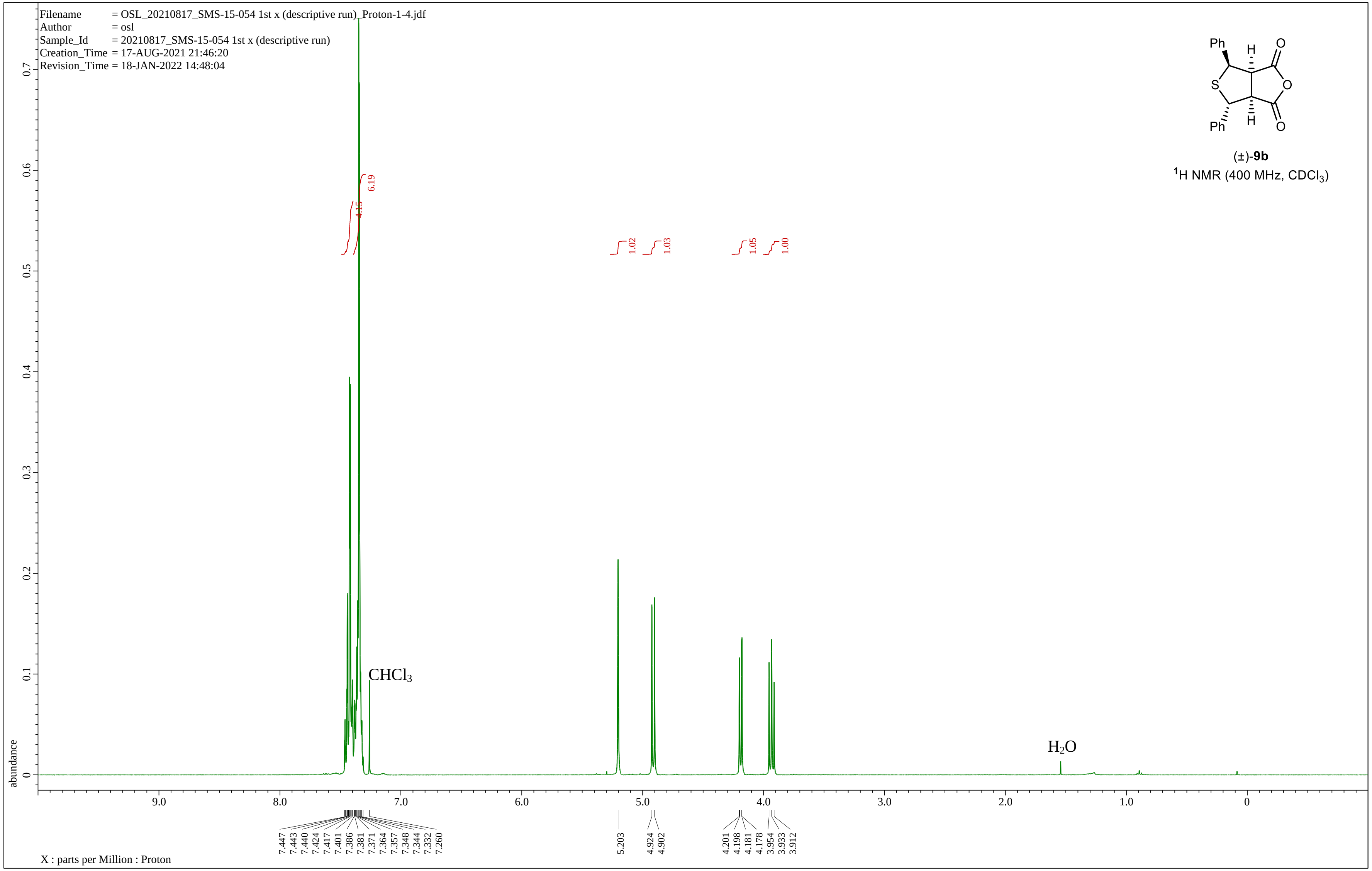

Supplementary Figure 103. ^{1}H NMR spectrum of compound (±)-**9b**, recorded at 400 MHz and 298 K in $CDCl_3$.

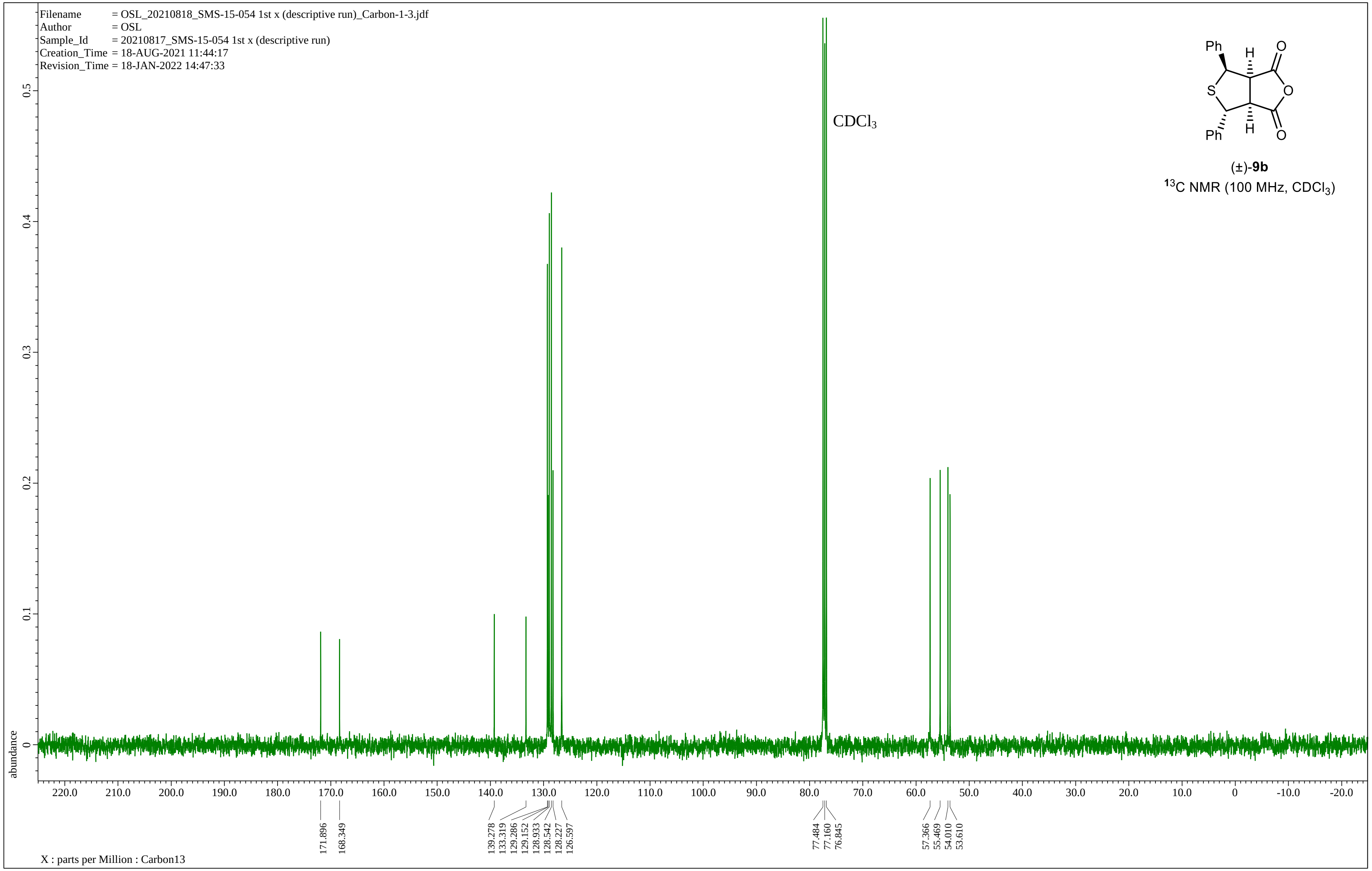

Supplementary Figure 104. ^{13}C NMR spectrum of compound (±)-**9b**, recorded at 100 MHz and 298 K in $CDCl_3$.

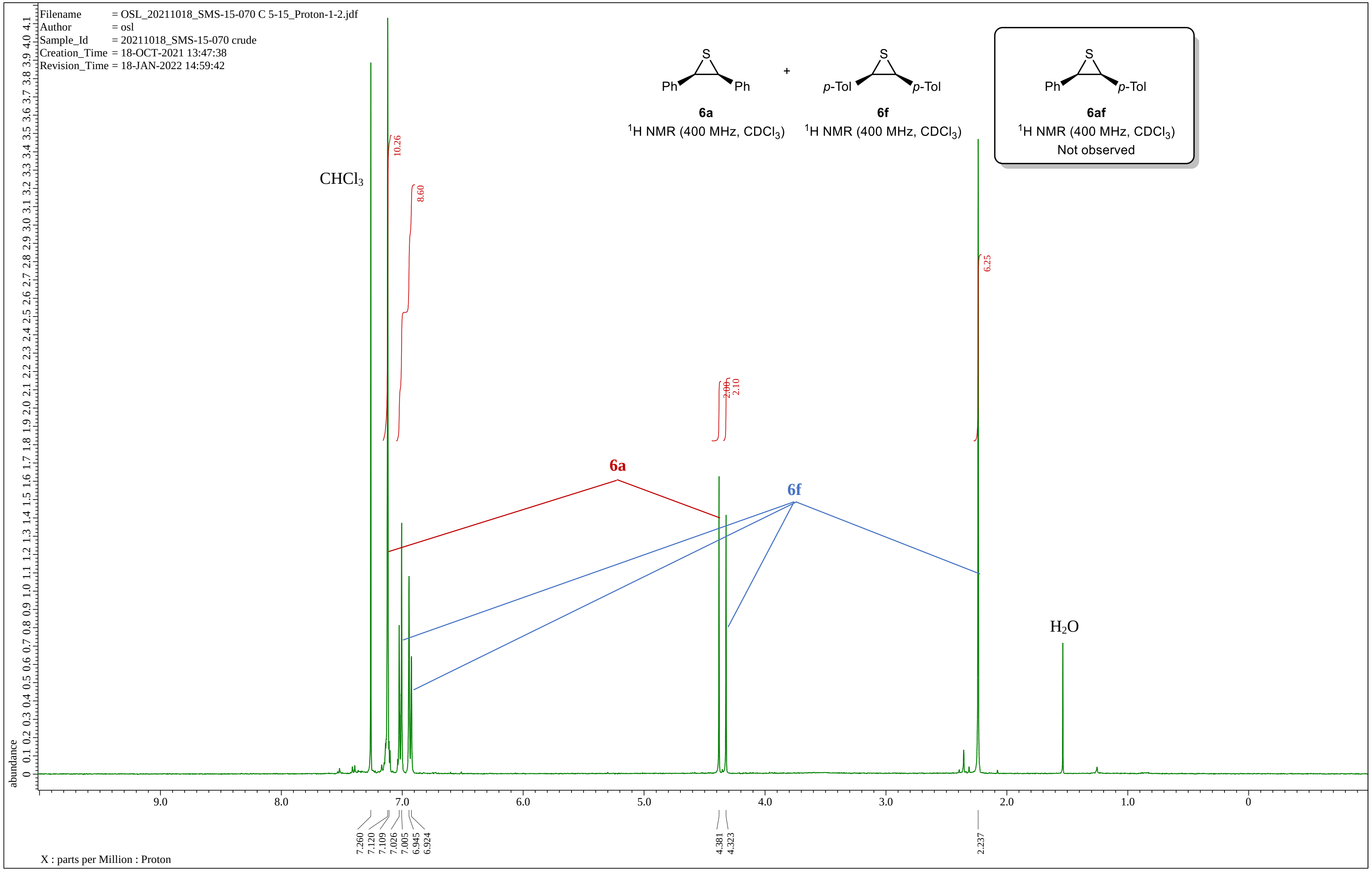

Supplementary Figure 105. ^{1}H NMR spectrum of the crossover experiment with **5a** and **5f**, recorded at 400 MHz and 298 K in $CDCl_3$.

4. Supplementary References

(1) Sek, D., Siwy, M., Bijak, K., Grucela-Zajac, M., Malecki, G., Smolarek, K., Bujak, L., Mackowski, S. & Schab-Balcerzak, E. Comparative Studies of Structural, Thermal, Optical, and Electrochemical Properties of Azines with Different End Groups with Their Azomethine Analogues toward Application in (Opto)Electronics. *J. Phys. Chem. A* **117**, 10320–10332 (2013).

(2) Khan, K. M., Jamil, W., Ambreen, N., Taha, M., Perveen, S. & Morales, G. A. An Expeditious Synthetic Approach Towards the Synthesis of Bis-Schiff bases (Aldazines) Using Ultrasound. *Ultrason. Sonochem.* **21**, 1200–1205 (2014).

(3) Chakraborty, M, Sengupta, D, Saha, T. & Goswami, S. Ligand Redox-Controlled Tandem Synthesis of Azines from Aromatic Alcohols and Hydrazine in Air: One-Pot Synthesis of Phthalazine. *J. Org. Chem.* **83**, 7771–7778 (2018).

(4) Saranya, S., Ramesh, R. & Sémeril, D. Non-Pincer-Type Arene Ru(II) Catalysts for the Direct Synthesis of Azines from Alcohols and Hydrazine under Aerobic Conditions. *Organometallics* **39**, 3194–3201 (2020).

(5) (a) Williams, W. M. & Dolbier, Jr., W. R. Thermal and Photochemical Rearrangements of Azine Oxides I. A Novel Pyrolytic Decomposition to Nitrile. *J. Org. Chem.* **34**, 155–157 (1969). (b) Chuang, K. V., Xu, C. & Reisman, S. E. A 15-Step Synthesis of (+)-Ryanodol. *Science* **353**, 912–915 (2016).

(6) Raredes, R., Bastos, H., Montoya, R. & Chavez, A. L. The Reactions of *N*-Benzoylperoxycarbamic Acid with Azines and Imines. *Tetrahedron* **44**, 6821–6830 (1988).

(7) Soldaini, G., Cardona, F. & Goti, A. Catalytic Oxidation of Imines Based on Methyltrioxorhenium/Urea Hydrogen Peroxide: A Mild and Easy Chemo- and Regioselective Entry to Nitrones. *Org. Lett.* **9**, 473–476 (2007).

(8) Ketcham, R. & Shah, V. P. *cis*- and *trans*-Stilbene Sulfides. *J. Org. Chem.* **28**, 229–230 (1963).

(9) Molecular Orbital PACkage 2016, http://openmopac.net/, Stewart J. J. P., Stewart Computational Chemistry, Colorado Springs, CO, USA.

(10) (a) Zhao, Y. & Truhlar, D. G. The M06 Suite of Density Functionals for Main Group Thermochemistry, Thermochemical Kinetics, Noncovalent Interactions, Excited States, and Transition Elements: Two New Functionals and Systematic Testing of Four M06-Class Functionals and 12 Other Functionals. *Theor. Chem. Acc.* **120**, 215–241 (2008). (b) Zhao Y. & Truhlar, D. G. Density Functionals with Broad Applicability in Chemistry. *Acc. Chem. Res.* **41**, 157–167 (2008).

(11) Gaussian 16, Revision C.01, Frisch, M. J., Trucks, G. W., Schlegel, H. B., Scuseria, G. E., Robb, M. A., Cheeseman, J. R., Scalmani, G., Barone, V., Petersson, G. A., Nakatsuji, H., Li, X., Caricato, M., Marenich, A. V., Bloino, J., Janesko, B. G., Gomperts, R., Mennucci, B., Hratchian, H. P., Ortiz, J. V., Izmaylov, A. F., Sonnenberg, J. L., Williams-Young, D., Ding, F., Lipparini, F., Egidi, F., Goings, J., Peng, B., Petrone, A., Henderson, T., Ranasinghe, D., Zakrzewski, V. G., Gao, J., Rega, N., Zheng, G., Liang, W., Hada, M., Ehara, M., Toyota, K., Fukuda, R., Hasegawa, J., Ishida, M., Nakajima, T., Honda, Y., Kitao, O., Nakai, H., Vreven, T., Throssell, K., Montgomery, J. A., Jr., Peralta, J. E., Ogliaro, F., Bearpark, M. J., Heyd, J. J., Brothers, E. N., Kudin, K. N., Staroverov, V. N., Keith, T. A., Kobayashi, R., Normand, J., Raghavachari, K., Rendell, A. P., Burant, J. C., Iyengar, S. S., Tomasi, J., Cossi, M., Millam, J. M., Klene, M., Adamo, C., Cammi, R., Ochterski, J. W., Martin, R. L., Morokuma, K., Farkas, O., Foresman, J. B. & Fox, D. J. Gaussian, Inc., Wallingford CT, 2016.

(12) Legault, C. Y. *CYLview20*, Université de Sherbrooke, 2020 (http://www.cylview.org).

(13) Bruker-AXS (2014). APEX2. Version 2014.11-0. Madison, Wisconsin, USA.

(14) Krause, L., Herbst-Irmer, R., Sheldrick, G. M. & Stalke, D. Comparison of silver and molybdenum microfocus X-ray sources for single-crystal structure determination. *J. Appl. Crystallogr.* **48**, 3–10 (2015).

(15) Sheldrick, G. Crystal structure refinement with SHELXL. *Acta Cryst. C* **71**, 3–8 (2015).

(16) Dolomanov, O. V., Bourhis, L. J., Gildea, R. J., Howard, J. A. K. & Puschmann, H. OLEX2: a complete structure solution, refinement and analysis program. *J. Appl. Crystallogr.* **42**, 339–341 (2009).
