# Supplementary material for: Synthesis of cis-thiiranes as diastereoselective access to epoxide congeners via 4π-electrocyclization of thiocarbonyl ylides
Source: Nat Commun. 2022 Aug 16;13:4818. doi: 10.1038/s41467-022-32499-3 (PMC9381720; doi:10.1038/s41467-022-32499-3)
Supplement: Supplementary file 2 — Description of Additional Supplementary Files [file 41467_2022_32499_MOESM2_ESM.docx]

**Description of Additional Supplementary Files**

**File Name: Supplementary Data 1
Description:** The Cartesian coordinates for all of the computed structures are provided as a text file.
